# Book of Posters

**DOI:** 10.1080/24740527.2025.2522031

**Published:** 2025-10-06

**Authors:** M. Gabrielle Pagé

## Animal or Non-Human PainAmyloid-beta Delivered by a Spinal Hydrogel Alleviates Neuropathic Pain in a Preclinical Model

Laura Bennett^1^, Hantao Zhang^1^, Timothy Cheung^1^, Nevatha Kingsley^1^, Maham Zain^1^, Quinn Pauli^1^, Maria Haji-Mahmoodzadeh^1^, Molly Shoichet^1^, Robert Bonin^1^

^1^University of Toronto

**Introduction**: Synaptic plasticity that allows for memory in the brain has mechanistic and functional parallels to synaptic plasticity that occurs between neurons in the spinal dorsal horn. The small peptide, amyloid-beta (Ab), is associated with memory loss in Alzheimer’s disease but is present at endogenously low concentrations in brains of healthy individuals. We hypothesize that Ab contributes to synaptic plasticity and sensory processing in the spinal dorsal horn. Our overall aim is to modulate Ab in the spinal dorsal horn to improve hypersensitivity in pain models.

**Methods**: We used Enzyme Linked Immunosorbent Assay (ELISA) to quantify Ab in the spinal cord and we tested mechanical sensitivity using von Frey. We intrathecally delivered synthetic Ab after inducing neuropathic pain by a spared nerve injury (SNI). Ab was delivered by a hydrogel with nanoparticles for spatiotemporal control of the Ab peptide release. Microglia morphology was analyzed by immunohistochemistry and Imaris.

**Results**: We observed that an intrathecal injection of synthetic Ab transiently improved the mechanical sensitivity of female mice but not male mice after SNI. To prolong the improvement in mechanical sensitivity, we delivered Ab via hydrogel, which significantly decreased mechanical sensitivity up to ten days post SNI. Additionally, we found a significant change in microglia morphology in the spinal dorsal horn after hydrogel delivery of Ab in female mice.

**Discussion/Conclusions**: Taken together, our results thus far indicate modulation of Ab may play a role in the mechanical sensitivity attributed to a model of neuropathic pain at the level of the spinal cord.

## Do Prior Pain Experiences Shape Pain Sensitivity? Effects of Repeated Testing and Contextual Conditioning in a Preclinical Chronic Pain Model

Damien Boorman^1^, Loren Martin^1^

^1^University of Toronto Mississauga

**Introduction**: Chronic pain can be amplified by prior experiences and learned associations with specific situations, stimuli, and contexts, a phenomenon known as nocebo hyperalgesia. We recently demonstrated nocebo hyperalgesia effects in rats with chronic pain and aimed to extend these findings to mice to further investigate their neural basis. We examined whether repeated exposure to noxious stimuli amplifies pain behaviors and whether conditioning to high pain in a specific context induces nocebo hyperalgesia in mice.

**Methods**: CD1 mice with a chronic constriction injury (CCI) of the sciatic nerve were assessed for cold allodynia using hind paw withdrawal (HPW) responses on a cold plate (0°C high pain, 20°C low pain) for 5 minutes. Experiment 1 compared repeated testing at high pain (twice daily, days +7–12 post-CCI) with a single test (day +12). Experiment 2 conditioned mice to a novel context to either high- or low-pain for 5 days, followed by low-pain testing in the same context to assess nocebo hyperalgesia.

**Results**: CCI produced cold allodynia, with females exhibiting greater hypersensitivity than males (p = .002). Repeated exposure amplified HPW responses in males (p = .05) and females (p = .02), suggesting prior pain experiences increased future sensitivity. However, conditioning to a high-pain context did not induce nocebo hyperalgesia (p = .56).

**Discussion/Conclusions**: These findings suggest prior pain experiences shape future pain sensitivity, potentially through learning or repeated exposure to noxious stimulation. However, unlike in humans and rats, context alone did not drive nocebo hyperalgesia, suggesting mice may not be ideal for studying these effects.

## The Gut Microbiota Promotes Pain in Fibromyalgia

Weihua Cai^1^, Manon Defaye^2^, Feng Wang^3^, Ji Zhang^1^, Emerson Krock^1^, Jeffrey S. Mogil^1^, Christophe Altier^2^, Yves De Koninck^3^, Nicholas J.B Brereton^4^, Emmanuel Gonzalez^1^, Yoram Shir^1^, Amir Minerbi^5^, Arkady Khoutorsky^1^

^1^McGill University, ^2^University of Calgary, ^3^Laval University, ^4^University College Dublin, ^5^Technion Israel Institute of Technology

**Introduction**: Fibromyalgia is a chronic syndrome characterized by widespread pain without evident tissue injury or pathology, affecting 2–4% of the population, primarily women, its underlying mechanisms remain unclear, and no effective targeted treatments are available. Emerging research suggests that gut microbiota alterations may contribute to various health conditions, including fibromyalgia. This study investigates whether microbiota from fibromyalgia patients can induce fibromyalgia-like symptoms, particularly pain, providing new insights into its potential mechanisms.

**Methods**: Fecal microbiota transplantation (FMT) from fibromyalgia patients to germ-free mice was performed. Pain sensitivity was assessed using von Frey, radiant heat paw-dwithdrawal, hot/cold plate, mouse grimace scales. Additional behavioral and molecular changes were analyzed using various assays, including limb hanging, grip strength, visceromotor response, 16S rRNA gene sequencing, whole-genome sequencing, immunofluorescence, ELISA, real-time PCR, H&E staining, in vivo Ca^2^⁺ imaging, RNA sequencing, metabolomics, and single-cell RNA sequencing.

**Results**: FMT from fibromyalgia individuals, but not from healthy controls, induces persistent pain hypersensitivity, reduced intraepidermal nerve fiber density, changed peripheral immunity, and altered spinal microglia. Notably, pain hypersensitivity reversed after subsequent FMT from healthy controls. An open-label pilot study further showed FMT from healthy individuals alleviated pain in fibromyalgia patients.

**Discussion/Conclusions**: Our findings suggest that gut microbiota alterations in fibromyalgia contribute to pain, potentially playing a causal role. Microbiota influence host physiology through immune modulation and bacterial metabolites. Fibromyalgia patients exhibit systemic changes, including immune dysregulation and metabolic shifts, supporting the role of gut microbiota in disease mechanisms. Further studies are needed to explore microbiota-targeted interventions for fibromyalgia.

## Not So Cool: Cryotherapy Prolongs The Duration Of Inflammatory Pain In Mice

Lucas Vasconcelos Lima^1^, Charlotte Pittman^1^, Boaz Laor^1^, Injy Fouda^1^, Olivia Cargnel^1^, Mélanie Di Maria^1^, Luda Diatchenko^1^, Jeffrey Mogil^1^

^1^Alan Edwards Center for Research on Pain, McGill University, Montreal, QC

**Introduction**: Cryotherapy, or icing, is universally employed and recommended for managing acute inflammation and pain. Recently, published data has suggested that reducing inflammation, despite the short-term benefit of pain relief, has a longer-term risk of delaying the resolution of pain and increasing the risk of developing chronic pain.

**Methods**: Whether cryotherapy would similarly lead to delayed pain resolution was tested in mice given either complete Freund’s adjuvant into the hind paw or subjected to an exercise-enhanced pain assay whereby hypotonic saline was injected into the gastrocnemius muscle before and after wheel running. Mice were tested for mechanical pain thresholds before and at multiple time points after injury.

**Results**: Cryotherapy, applied over three days using different timing protocols, was observed to prolong the duration of pain behavior by approximately two-fold, from ⁓15 days to >30 days. Neutrophil injection into the hind paw was found to prevent the pain chronification caused by cryotherapy. Alternate therapies, including heat, menthol, and contrast therapy (alternating heat and cold) did not affect pain resolution.

**Discussion/Conclusions**: We conclude that, like steroid and non-steroidal anti-inflammatory drugs, the use of cryotherapy should be reconsidered for the management of acute inflammatory injury.

## Pain-Associated Tube Isolation Behaviors of Mice in Nonsocial and Social Contexts

Oakley Morgan^1^, Miranda Roberts Nouel^1^, Junshen Wang^1^, Yifan Han^1^, Jeffrey Mogil^1^

^1^McGill University

**Introduction**: Research from our lab has revealed the influence of the social environment on pain, most notably that social contexts can modulate the expression of pain in humans and mice. Here, we aim to expand on this knowledge by differentiating pain-related isolation (or hiding) behaviors of male and female mice in acute pain, in multiple social settings.

**Methods**: Male and female CD1 mice received an intraplantar formalin injection in the left hind paw and were placed in home cages with one or more red perplex tubes, either alone, or in the presence of two uninjured littermates of the same sex. Isolation (time in tube), pain behaviors (time spent licking/attending the injured paw) and location of expressed pain behaviors (inside/outside tube) were assessed during the second formalin phase.

**Results**: When alone, male and female formalin injected mice spent more time isolating than saline injected mice. There were no differences in the preferred location of pain behavior expression between the sexes, with both male and female mice choosing to express their pain in isolation.

When assessed in a triad social setting amongst littermates, male mice continued to isolate during pain, while female counterparts spent significantly less time in the tubes. The location in which they expressed their pain shifted drastically, with female mice expressing their pain predominantly outside the tube, and males inside.

**Discussion/Conclusions**: Isolation behavior of injured female mice changes in the presence of other uninjured females, while male behavior remains constant, suggesting that females are more adaptive to social dynamics.

## Approaching Another in Pain: Opioid Mediated Preference Toward a Familiar

Crystal Mui^1^, Navdeep Lidhar^1^, Jennet Baumbach^1^, Sana Khan^1^, Zainab Zakaria^1^, Ashley Mutasa^1^, Seyed Asaad Karimi^1^, Loren Martin^1^

^1^University of Toronto

**Introduction**: Social interactions profoundly influence pain perception and consoling behaviors toward distressed individuals. However, the extent to which familiarity with the individual in pain shapes the behavioral responses to a conspecific in pain and the neural mechanisms underlying these behaviors, remain unclear.

**Methods**: To investigate this, we assessed approach behavior toward another mouse in pain using a social affective preference test.

**Results**: Males had no preference interacting toward a familiar in pain or a familiar not in pain, whereas females did. When exposed only to a mouse in pain, both sexes had increased interaction with the familiar in pain compared to familiar not in pain. This preference was mediated by the opioid system, as several opioid antagonists blocked the social approach toward pain in female siblings.

**Discussion/Conclusions**: These findings suggest a critical role for opioid receptors in modulating these behaviors and the importance of familiarity on social responses to pain. By understanding the interplay between social behavior, pain, and relationship dynamics may offer insights into the neural basis of empathy and the social modulation of pain.

## Postictal Changes in Somatosensory and Affective Components of Pain Following Electrical Amygdala Kindling In Rats

Evana Xiao^1^, Kaylea MacDonald^2^, Kerri Mozessohn^2^, Neil M. Fournier^2^

^1^University of Toronto, ^2^Trent University

**Introduction**: Pain is a complex experience, which consists not only of a sensory/discriminative dimension (e.g., the quality, location and intensity) but also an affective affective/motivational dimension (e.g., unpleasantness or aversiveness). Kindling is the process by which daily administration of electrical stimulations to a particular brain region results in the development and intensification of motor seizures. We have previously shown that amygdala kindling produces long-lasting increases in fear and anxiety-related behavior in rats. Interestingly, many of the same neural circuits impacted by kindling are also involved in processing pain information. This led us to hypothesize that seizures might sensitize neural circuits involved in mediating pain responses, which could lead to impairments in the processing of sensory and affective features of pain.

**Methods**: In the present study, rats underwent short-term (30 stim) amygdala kindling and at various points were examined on a battery of pain-related behaviors.

**Results**: We found that kindling was associated with a delayed development of hypersensitivity to mechanical but not thermal pain stimuli. In addition, kindled rats engaged in greater displays of emotional pain behaviors and showed greater activation of the rostral anterior cingulate cortex – a key structure in affective pain perception in humans and animals – in response to inflammatory pain induced by formalin. Interestingly, chemogenetic inhibition of excitatory neurons in the anterior cingulate cortex alleviated kindling-induced impairments in conditioned pain avoidance learning and promoted pain relief.

**Discussion/Conclusions**: These results suggest that chronic seizures can alter pain sensitivity and further highlight the involvement of the anterior cingulate in the modulation of pain-induced emotional behaviors.

## Assessment, Diagnosis and Measurement of PainThe Diagnostic Importance of Chest Pain in the Critical Evaluation of COVID-19 Patients in the Emergency Department: A Retrospective Cohort Study

Maayan Ben Sasson^1, 2^, Shay Perek^3, 4^

^1^Alan Edwards Pain Management Unit, McGill University Health Center, Canada, ^2^Institute for Pain Medicine, Rambam Health Campus, Israel, ^3^Department of Emergency Medicine, Rambam Health Care Campus, Haifa, Israel, ^4^Rappaport Faculty of Medicine, Technion-Institute of Technology, Haifa, Israel

**Introduction**: Acute chest pain is a frequent emergency department (ED) presentation, with causes ranging from benign to life-threatening. Rapid stratification is crucial, especially during the COVID-19 pandemic, where chest pain often indicates potential cardiac injury. This study explores the diagnostic significance of chest pain at ED admission in predicting COVID-19 severity.

**Methods**: We analyzed de-identified data from adult COVID-19 patients admitted to the ED, utilizing the MDclone platform and used Natural Language Processing (NLP) to identify chest pain complaints (Cohen’s kappa 0.92). We examined clinical and laboratory data to correlate chest pain with disease severity.

**Results**: Among 5,504 COVID-19 patients admitted from July 2020 to January 2023, 1,260 (22.9%) reported chest pain. Of these, 125 (9.9%) were admitted to the ICU, and 52 (4.1%) had cardiac complications, yet the 30-day mortality rate was lower in the chest pain group (7.1% vs. 15.4%, p < .0001). For 30-day all-cause mortality, multivariate analysis showed vaccination (AOR 0.250, p < .0001) and oxygen saturation below 90% (AOR 2.186, p < .0001) as stronger predictors whereas chest pain was not one of the predictors. Chest pain remained significant for ICU admission (OR 1.250, p < .0001) and cardiac complications (AOR 5.682, p < .0001).

**Discussion/Conclusions**: Chest pain in COVID-19 patients is linked to lower 30-day mortality but higher ICU admission rates. While it does not independently predict mortality, it serves as a key indicator for critical care needs and potential cardiac events. Vaccination and oxygen saturation are critical for assessing disease severity.

## Body Perception Disturbances Across Pain Conditions: A Scoping Review

Angenie Christy Antony^1^, Arunica Bahl^2^, Eden Bishop^2^, Samantha Hutchins^2^, Bhumi Thakkar^2^, Joycelyn Harris^2^, Tara Packham^2^

^1^ Faculty of Health Sciences, McMaster University, ^2^School of Rehabilitation Sciences, Faculty of Health Sciences, McMaster University

**Introduction**: Body perception disturbances (BPD) include symptoms of neglect, negative emotions, and sensory-perceptual distortions of the affected body parts. Although commonly reported in complex regional pain syndrome (CRPS), this phenomenon may be associated with other chronic pain conditions, but lacks a clear definition. The purpose of this scoping review was to examine how body perception disturbances are defined, described, and measured across several pain conditions in the literature.

**Methods**: We systematically searched CINAHL, PsycINFO, PubMed, MEDLINE, Embase, Cochrane Library, Web of Science, and Pain+ for studies describing and/or measuring BPD in adults with any pain condition. Sources were dual-screened for inclusion.

**Results**: We identified 74 studies reporting descriptions of BPD in different pain conditions: CRPS (n = 29), limb amputation or phantom limb pain (n = 17), low back pain (n = 9), fibromyalgia (n = 4), general chronic pain (n = 4), pregnancy-related lumbopelvic pain (n = 2), and others (n = 8). BPD was described as changes in the body’s mental schema, phantom limb sensations, neglect-like symptoms, altered sense of ownership of the affected part, and distortions of shape, size, length, and heaviness of the affected part. BPD was most commonly measured by the Bath CRPS Body Perception Disturbances Questionnaire, Fremantle Back Awareness Questionnaire, Neglect-Like Symptoms Questionnaire, or subjective drawings and descriptions of the body.

**Discussion/Conclusions**: Despite the recognition of BPD across various pain conditions, it is inconsistently described. Many definitions only capture aspects of BPD, making it difficult to be considered as a distinct phenomenon. Further research is needed to develop a comprehensive definition of this complex symptom constellation.

## A Predictive Model Approach to Thermal Grill Illusion Responsiveness

Matthew Cormie^1^, Pedram Mouseli^1^, Massieh Moayedi^1^

^1^Centre for Multimodal Sensorimotor and Pain Research, Faculty of Dentistry, University of Toronto, Toronto, Canada

**Introduction**: The thermal grill illusion (TGI) is a paradoxical pain phenomenon arising from the spatial alternation of warm and cool, innocuous stimuli. However, only 20–50% of individuals are TGI responders – i.e., they experience pain from interlaced innocuous, cool and warm stimuli. It remains unknown what physiological (e.g., thermal sensitivity) or psychosocial factors (e.g., mood, pain-related cognitions) factors contribute to TGI responsiveness.

Here, we aim to determine whether TGI responsiveness can be predicted using demographic, physiological, and psychosocial measures. We hypothesize that these factors will predict TGI responsiveness with moderate accuracy.

**Methods**: Procedures approved by the University of Toronto’s Human Research Ethics Board.

We used an elastic-net regularization model to determine which factors could predict which individuals are likely to be TGI responders with the following features: age, thermal pain thresholds, Beck’s depression inventory II (BDI), State-trait anxiety inventory, Pain Catastrophizing Scale (PCS) subscales, fear of pain questionnaire, and somatosensory amplification scale. The weight of each class is assigned as inversely proportional to the class frequencies. Model performance was evaluated using a ten-fold cross validation.

**Results**: The model’s overall balanced accuracy was 0.71 (F1 score: 0.7; precision: 0.7), indicating average model performance. When disaggregating the data by sex, the male group’s balanced accuracy was 0.78, while the female groups was 0.68. The features with the highest weights were thermal pain thresholds, BDI, and PCS subscales.

**Discussion/Conclusions**: Our model shows we can predict TGI responsiveness with an average accuracy when factoring in heat and cold pain thresholds, along with psychosocial factors.

## The burden of pain and co-existing symptoms in critically ill adults: A multisite observational study in Quebec

Céline Gélinas^1, 2^, Geneviève Laporte^1, 2^, Emilie Gosselin^3, 4^, Robin Kagie^2^, Caroline Arbour^5, 6^, Francis Bernard^7, 8^, Virginie Williams^9^, David Williamson^8, 10^, Julie Houle^11, 12^, Jean- Nicolas Dubé^7, 12^, Han Ting Wang^7, 13^, Marc Perreault^10, 14^, Andrea Maria Laizner^14, 15^

^1^ Ingram School of Nursing, McGill University, ^2^Centre for Nursing Research and Lady Davis Institute, Jewish General Hospital, ^3^École des sciences infirmières, Université de Sherbrooke, ^4^Centre de recherche du CHUS (Center hospitalier de l’Université de Sherbrooke), ^5^Faculté des sciences infirmières, Université de Montréal, ^6^Hôpital-du- Sacré-Coeur-de-Montréal, ^7^Faculté de médecine, Université de Montréal, ^8^Hôpital du Sacré-Coeur de Montréal, ^9^Équipe de recherche en soins intensifs, Hôpital-du-Sacré- Coeur-de-Montréal, ^10^Faculté de pharmacie, Université de Montréal, ^11^Département des soins infirmiers, Université du Québec à Trois-Rivières, ^12^CIUSSS Mauricie-du Québec, ^13^Centre Hospitalier Université de Montréal (CHUM), ^14^McGill University Health Center, ^15^Research Institute – McGill University Health Center

**Introduction**: Pain is a frequent and distressful symptom in critically ill adults in the intensive care unit (ICU). While multiple symptoms often coexist in the ICU, research on this topic is limited. This study aimed to describe the intensity and distress of pain and co-existing symptoms, and their associations in ICU patients.

**Methods**: In this multisite observational study, patients were recruited from 5 mixed ICUs in the province of Quebec. Eligibility required an ICU stay of over 36 hours, reported pain, and being able to self-report in French or English. Participants completed the Edmonton Assessment Symptom Scale (revised ICU version) which included 11 symptoms, each rated for intensity (0 = no symptom to 10 = worst symptom) and distress (0 = not distressful to 10 = most distressful) in the past 24 hours before ICU discharge.

**Results**: Participants (n = 350) were mostly men (66%), had a mean age of 62 (SD = 14 years) and mainly originated from Canada (71%) and Europe (15%). Total number of co- existing symptoms (>0/10 for intensity) was high (median = 7 symptoms; interquartile range or IQR = 5–9). Most intense symptoms included tiredness (median = 6; IQR = 3–8), pain (median = 5; IQR = 3–8) and lack of sleep (median = 5; IQR = 0–8). These symptoms were mildly distressful (medians = 2 for pain and tiredness, and 3 for lack of sleep). Pain correlated moderately with general discomfort (Spearman’s rho = 0.43; p < .001) and mildly with anxiety and fear (both with Spearman’s rho = 0.29; p < .001).

**Discussion/Conclusions**: Participants reported several co-existing distressful symptoms during their ICU stay. Regular and accurate assessment of pain and co-existing symptoms is essential to optimize treatment and patient outcomes.

## Body perception at the pelvic girdle with the two-point estimation measure: a reliability and validity study

Bradley Halliday^1^, Jennifer Freeman^1^, Sarah Chatfield^1^, Jonathan Marsden^1^

^1^ Faculty of Health, University of Plymouth, England

**Introduction**: Body perception disturbances have been evidenced in low back pain using the two-point estimation (2-PE) measure. The 2-PE has been studied with a method suitable for the assessment unilateral pain, not included a pain-free group, nor examined it at the pelvic girdle (PG).

**Methods**: 2-PE was tested with a digital calliper (two points 120 mm apart), in-person and remotely, using two methods at the PG: a lateral and central measure (two points crossing the mid-line).

Reliability and agreement (in-person versus remote) was assessed with Intraclass Correlation Coefficients (ICC) and Bland Altman plots. Validity of the 2-PE at the PG was evaluated comparing data from two populations (pain-free women and women with PPGP).

**Results**: 22 healthy pain-free participants and 13 participants with chronic (PPGP) were recruited. Intra-rater reliability of the 2-PE (in-person) at the PG was good (central measure – ICC = 0.89 95%CI 0.73–0.95) to excellent (lateral measure – ICC = 0.91 95%CI 0.78–0.96). Interrater reliability was good for all measures (in-person (ICC = 0.79–0.80) and remote (ICC = 0.90). There was satisfactory agreement between in-person and remote 2-PE measure; lateral (mean difference −3.20), central (PG, mean difference 8.09). Women with PPGP were more accurate in their estimation with both methods but did not reach statistical significance: Estimation error difference = 18.22 (95%CI −2.69–39.14), p = .08 (Central – PG); 7.21 (95%CI −34.46–48.89), p = .69 (Lateral).

**Discussion/Conclusions**: The 2-PE is a reliable measure for assessing perceptual disturbances at the PG. The difference in estimation errors may indicate that women with bilateral pain experience greater perceptual disturbances.

## Triage Tools for Surgical Decision-Making in Patients with Shoulder Pain, An Integrative Review

Samah Hassan^1, 2^, Kenneth J. Faber^1, 2, 3, 4^, Joy C MacDermid^1, 2, 4, 5^

^1^The Roth McFarlane Hand and Upper Limb Center, St. Joseph’s Hospital, London, ON, Canada, ^2^Faculty of Health Sciences/School of Physical Therapy, Western Ontario University, ^3^Department of Surgery, Western University, London, ON, Canada, ^4^Clinical Research Lab, Roth McFarlane Hand and Upper Limb Center, St. Joseph’s Health Center, London, Ontario, Canada, ^5^Physical Therapy and Orthopedic Surgery, University of Western Ontario, London, Ontario, Canada

**Introduction**: Shoulder pain is a major cause of work absenteeism, affecting one in three adults. Although nonsurgical treatments like physical therapy are recommended as the first line of management, 40% of patients are referred for surgery before exploring these options. A triage tool is needed to better guide shoulder pain referrals.

This review identified existing triage tools used for shoulder pain referrals, and critically examined how they were developed, their validity, and reliability against existing guidelines for shoulder pain management, indications for surgery, and criteria for surgical referral.

**Methods**: An integrative review approach was used to address the objectives. We conducted a comprehensive search across major databases (Medline, PubMed, Embase, CINAHL plus) and gray literature. Studies focusing on triage tools, referral processes, or surgery candidacy and guidelines for shoulder pain were included.

**Results**: Our preliminary searches retrieved 4252 articles. We selected 378 for full

review and identified 32 articles. Thirteen articles were randomized trials comparing surgical and nonsurgical treatments to determine surgical indicators. Seven articles explored predictor models for surgical outcomes. The remaining articles focused on guidelines and clinical decision-making tools for diagnosing and treating shoulder pain. No specific triage tools or referral processes were found.

**Discussion/Conclusions**: The findings suggest that personalized triage tools that account for patient characteristics, pain phenotypes, functional status, and patient goals – are necessary and may optimize the referral process and guiding appropriate treatment decisions. This review concludes that a structured referral format is needed to prioritize nonsurgical treatment options and minimize unnecessary surgical referrals.

## Redefining Minimal Dataset Requirements for Pain Care Programs: Integrating Operational and Pain Education-Specific Data with a Stepped Care Approach

Rangana Hetti Arachchige^1^, Matt Smith^1^

^1^ Pain Care BC

**Introduction**: Pain management registries traditionally focus on patient-reported outcomes through pain questionnaires, often overlooking critical operational and pain education data. This limited scope can result in missed opportunities to improve pain care and address operational challenges. To enhance pain management, there is a need for an innovative approach that integrates clinical, operational, and educational data.

**Methods**: We introduce a novel framework to expand pain registry datasets by integrating not only patient-reported outcomes but also key operational metrics, such as treatment adherence, resource utilization, and pain education data. This approach aligns with a stepped care model, tailoring interventions based on the severity of pain and patient needs. The expanded dataset aims to provide a more comprehensive understanding of both patient experiences and operational efficiency in pain management.

**Results**: By incorporating these additional data points, the framework supports more effective resource allocation, improves patient care, and enhances overall outcomes. It allows for more personalized, responsive pain management that can better address the diverse needs of patients. This approach bridges the gap between clinical practice, operational data, and pain education, creating a more holistic pain management strategy.

**Discussion/Conclusions**: This innovative framework transforms traditional pain management registries by integrating operational and educational data. It provides a comprehensive view of pain care that supports data-driven decisions, improves patient outcomes, and optimizes resource use. The framework represents a significant advancement in pain care, offering a model for more responsive, personalized, and efficient pain management programs.

## Hemifacial Continuous Pain with Autonomic Symptoms- A Case Report

Sripriya Jayaraman^1^, Fernanda Yanez^2^

^1^ Mount Siani Hospital, ^2^University of Kentucky

**Introduction**: Hemifacial continuous pain with autonomic symptoms is a part of trigeminal autonomic orofacial pains. These have been described as pain attacks occurring in the orofacial region without concomitant headache but with characteristics and features of trigeminal autonomic cephalalgias. This condition presents as constant side-locked pain with associated autonomic features. The following is a case report of hemifacial continuous pain with autonomic symptoms.

**Methods/Results**: 53-year-old male patient reported with continuous pain in the upper left dental quadrant for the last 4 years. The pain was described as aching, and throbbing with an intensity of 5–6/10. Associated symptoms included conjunctival injection (especially on awakening), rhinorrhea(occasional), awakening from sleep. He was unsuccessfully treated by root canal therapy/apicoectomy/extraction on teeth #14-15/ antibiotics and nasal spray. A detailed examination of cranial nerves, cervical, masticatory muscles and intraoral structures was performed; found within normal limits. Brain MRI with and without contrast was unremarkable. Diagnosis of hemifacial continuous pain with autonomic symptoms was given with a trial of indomethacin. On 1-week follow-up, he reported complete pain relief at 25 mg tid. Pain returned upon stopping indomethacin. He reported occipital headache as adverse effect and ceased use.

Sphenopalatine ganglion (SPG) block was performed, resulting in pain resolution for 8 hours. Patient is maintained on a combination of SPG blocks and gabapentin.

**Discussion/Conclusions**: Trigeminal autonomic orofacial pain is rare and facial presentation of hemicrania continua not previously described. Knowledge of the condition is crucial for diagnosis and successful treatment.

## Improving Primary Care Chronic Pain Management through the Development of Team Care Digital Aid

Irina Kudrina^1, 2, 3^, Julia Osborne^4^, Teri Clark^5^, Maxim Yakimenko^6^, Roland Grad^4, 7^

^1^ Family Medicine and Anesthesia Departments, McGill University, ^2^Research Institute, McGill University Health Center, ^3^Research Center, University of Montreal, ^4^Lady Davis Research Institute, ^5^McGill University, ^6^Concordia University, ^7^Department of Family Medicine, McGill University

**Introduction**: Months to years of fragmented documentation significantly hinder primary care-level chronic pain (PCCP) care provision. Our quality improvement team developed the Team Care Digital Aid (TCDA) to assist PCCP coordination, team communication, and promote collaborative decision making. Three objectives were to 1) Structure digital collection of multi domain clinical information in a condensed, practical format; 2) Minimize manual tasks by introducing automated data consolidation, progress tracking, and clinical forms generation; 3) Integrate reminders and resources for patients and care providers.

**Methods**: Three-phase project, participatory methodology, quality improvement lens. A literature review and discussions with family physicians and resident trainees informed project and tool development. 28 participants across multiple sites and career stages include five patient-partners, care providers (family medicine, other specialties, and allied health) and two administrators, who contributed >60 hours of semi-structured feedback interviews. Sessions were recorded and transcribed verbatim. Practical thematic analysis will be conducted after Phase-3 data collection.

**Results**: Participants’ feedback was grouped into two major categories: 1. PCCP and worker compensation (insurance) team care. 2. The potential practical use of the TCDA. Examples of subcategories include administrative burden of PCCP; implementation barriers; team-based care and clinical training; use in collaborative decision making.

Outputs. HTML tool version and its functionalities: (i) Automated generation of clinical summaries, plans and forms; (ii) Automated opioid dose calculations; (iii) Automated multidomain progress tracking; (iv) Resources for patients and care providers.

**Discussion/Conclusions**: The project aims to reduce administrative burdens and assist with PCCP and collaborative decision making. Funding: PBRN, Reseau-1.

## Using Data from the Manage My Pain App to Predict Clinical Outcomes at the Toronto General Hospital Transitional Pain Service

Anna Lomanowska^1, 2^, James Skoric^2, 3, 4^, Tahir Janmohamed^4^, Heather Lumsden-Ruegg^5^, Joel Katz^1, 4, 5, 6^, Hance Clarke^1, 6^, Quazi Abidur Rahman^7^

^1^ Transitional Pain Service, Department of Anesthesia and Pain Management, Toronto General Hospital, University Health Network, Toronto, ON, ^2^These authors contributed equally to this work, ^3^Department of Electrical and Computer Engineering, McGill University, Montreal, QC, ^4^ManagingLife, Toronto, ON, ^5^Department of Psychology, York University, Toronto, ON, ^6^Department of Anesthesiology and Pain Medicine, University of Toronto, Toronto, ON, ^7^Department of Computer Science, Trent University, Peterborough, ON

**Introduction**: Digital health apps such as Manage My Pain (MMP) are popular tools to enhance self-management of pain. Using machine learning, r entered into these apps can be leveraged to predict user outcomes. This study applies machine lear1ning to real- world user data from the MMP app to predict clinically significant pain-related improvements among patients at the Toronto General Hospital Transitional Pain Service (TPS).

**Methods**: REB approval was obtained and users gave consent for use of anonymized data for research. Information entered into MMP user profiles, pain records, daily reflections, and questionnaires by 160 TPS patients over one-month was used to develop a machine learning model. The model utilized logistic regression with recursive feature elimination to predict clinically significant improvements in pain interference, assessed by the PROMIS Pain Interference 8a v1.0 questionnaire. The model was tuned using 10-fold cross-validation and performance was tested using leave-one-out cross-validation.

**Results**: The model predicted patient improvement in pain interference with 79% accuracy and an area under the receiver operating characteristic curve of 0.82. It showed balanced class accuracies between improved and non-improved patients, with 0.76 sensitivity and 0.82 specificity. All MMP app data, not just clinical questionnaire responses, were key to classifying patient improvement.

**Discussion/Conclusions**: When used in a machine learning model, data from a digital health app provides meaningful information alongside clinical questionnaire responses to effectively predict which pain patients will show improvement. The findings emphasize the potential of machine learning in real-world clinical settings to improve personalized treatment plans.

## Development of a Core Outcome Set for Clinical Trials of Extended Reality for Acute and Chronic Pain Across the Lifespan

Giulia Mesaroli^1^, Lauren Harris^1^, Courtney W. Hess^2^, Laura E. Simons^2^, Deirdre Logan^3^, Jennifer N. Stinson^1^

^1^The Hospital for Sick Children, ^2^Stanford University School of Medicine, ^3^Boston Children’s Hospital

**Introduction**: The number of clinical trials of extended reality (XR) for pain has exponentially increased in the past two decades. In order to pool study results and determine the effectiveness of XR for pain, a core outcome set (COS) is needed. Phase 1 systematically reviewed existing literature. Phase 2 used a consensus approach to determine a set of core outcome domains. The aim of this study (Phase 3) was to determine a COS of measures within each domain.

**Methods**: First, a list of measures within each domain were identified from existing literature. Second, a Delphi survey of experts was administered electronically in August 2024 to determine the suitability of each measure, rated on a 11-point numerical rating scale (led by Dr. Logan, Boston Children’s Hospital). Third, a CIHR funded consensus conference is scheduled for January 2025 (led by Dr. Stinson, SickKids Hospital) to determine the final COS of measures.

**Results**: Delphi survey respondents (n = 52) indicated expertise in XR for pain in clinical (75%) and research (89%) settings across pediatric (67%) and adult (47%) populations. Within each domain, 3 measures with the highest rated suitability were selected as candidate measures for the COS. Twenty-one experts including clinicians, researchers, people with lived experience are scheduled to attend the consensus conference, the final COS determined from this meeting will be presented.

**Discussion/Conclusions**: Existing evidence on XR for pain management is challenged with heterogenous approaches to measuring effectiveness outcomes. Implementing a COS is a promising step to advance the field of XR pain research.

## Screening for Neuropathic Pain in Adolescent Cancer Survivors: Sensibility and Validity of the Pediatric PainSCAN©bffv

Giulia Mesaroli^1^, Alex Pizzo^2^, Paul Nathan^1^, Nicole M. Alberts^2^, Jennifer Stinson^1^

^1^ The Hospital for Sick Children, ^2^Concordia University

**Introduction**: Neuropathic pain (NP) in adolescent cancer survivors can result from cancer or its treatments. The Pediatric PainSCAN© is the first NP screening tool developed specifically for children and validated in the pediatric pain clinic setting. However, validity in the pediatric cancer setting is unknown. This study aimed to test the sensibility (ease of use, item relevance, comprehensibility) and convergent validity (relatedness of scores to conceptually similar tools) of the Pediatric PainSCAN© in adolescent cancer survivors.

**Methods**: A cross-sectional survey was administered to cancer survivors aged 13–18 in a pediatric cancer survivorship clinic. The survey included demographic and pain characteristics, NP screening tools (painDETECT and Pediatric PainSCAN©), and a 10-item sensibility questionnaire (4 open-ended questions and 6 questions rated on an 8-point Likert scale where 0 = unacceptable and 7 = excellent). Participant characteristics and questionnaire scores were evaluated using descriptive statistics. Convergent validity was evaluated by comparing participant scores on the two screening tools with Spearman’s correlation coefficient.

**Results**: Participants (n = 33) were 73% female between 13–18 years old (median age 16). Median scores (interquartile range) on the Pediatric PainSCAN© and painDETECT were 31/100 (14, 50) and 11/38 (7, 16). Scores between the two tools were moderately correlated (ρ 0.34, p-value 0.056). Participants rated the Pediatric PainSCAN© highly with respect to clarity of questions (median score 6/7), adequate instructions (median score 6/7), and completion time (median 5/7).

**Discussion/Conclusions**: The Pediatric PainSCAN© is a sensible tool to screen for NP in adolescent cancer survivors. Further testing is needed to determine reliability and criterion validity in this setting.

## Diurnal Pain Rhythmicity in Clinical Trial Participants with Fibromyalgia: A Comparison with Neuropathic Pain

Ryan Navarro^1^, Wilma Hopman^2^, Ian Gilron^3^

^1^Faculty of Medicine, Queen’s University, Kingston, Ontario, Canada, ^2^Department of Public Health Sciences, Queen’s University, Kingston, Ontario, Canada, ^3^Department of Anesthesiology & Perioperative Medicine, Kingston Health Sciences Center, Queen’s University

**Introduction**: Diurnal rhythmicity of chronic pain intensity is well recognized, for example, with neuropathic pain (NP) intensity typically highest in the evening, versus osteoarthritis pain highest much earlier in the day. However, little is known about diurnal pain rhythmicity in patients with fibromyalgia.

**Methods**: We compared pain rhythmicity of fibromyalgia to that of NP by conducting exploratory analyses of data from two recent fibromyalgia clinical trials (68 pooled participants) and one NP clinical trial (55 participants). In these trials, pain intensity (0–10 scale) was rated at 8:00 AM and 8:00 PM during a 7-day pretrial baseline period and throughout each trial. Analyses evaluated morning versus evening pain intensity differences for each condition, as well as possible patient-specific determinants of diurnal variability.

**Results**: Baseline data demonstrated statistically significant diurnal rhythmicity in both conditions. Evening pain was higher than morning pain by approximately 20% in NP and approximately 7% in fibromyalgia. The morning-evening pain intensity difference was significantly greater for NP versus fibromyalgia. In exploratory analyses of fibromyalgia participants, older age, shorter pain duration, and more severe ‘hot-burning’ pain rating were significantly correlated with greater morning-evening differences. In NP participants, higher body weight and higher pain interference with walking and work were significantly correlated with lower morning-evening differences.

**Discussion/Conclusions**: These exploratory analyses suggest that fibromyalgia pain is generally more intense in the evening versus morning. Although this pattern appears less pronounced than with NP, it should be studied further and recognized when investigating and implementing fibromyalgia treatment interventions.

## Exploring the Association Between Adiposity, Pain Intensity and Synovitis in People with Knee Osteoarthritis: A Cross-Sectional Study

Y.V. Raghava Neelapala^1^, Tom Appleton^2^, Luciana Macedo^1^, Steven Hanna^1^, Dylan Kobsar^1^, Trevor Birmingham^2^, Lisa Carlesso^1^

^1^ McMaster University, ^2^Western

**Introduction**: We investigated (i) the association of adiposity with pain intensity and/or effusion-synovitis in people with knee osteoarthritis (OA) and (ii) whether indicators of systemic immune inflammation moderate the above associations.

**Methods**: Individuals with knee OA were sampled from the Western Ontario Registry for Early Osteoarthritis Knee Study. Total body and visceral fat percentages were measured using bioimpedance analysis and synovitis was graded using knee ultrasonography. Systemic immune-inflammation index (SII) and Systemic immune response index (SIRI) were considered markers of systemic immune inflammation. Multiple linear regression and logistic regression with interaction terms were performed to examine the association between fat percentages and pain intensity/synovitis and the interaction effect of fat and SII/SIRI on pain intensity/synovitis. Analyses were adjusted for potential confounders (age, sex, body mass index (BMI), radiographic severity of the opposite knee, and anxio- depressive symptoms).

**Results**: Data from 225 participants (mean age: 61.1 (10.9), 68% female, mean BMI: 31.7 (7.7)) was analyzed. No significant associations were found for adjusted fat and pain intensity models (total body fat: β (adjusted): – 0.03 (−0.49 to 0.55) and (visceral fat: β (adjusted): −0.32 (−1.14 to 0.49). Similarly fat and synovitis, adjusted models were non- significant (total body fat: OR (adjusted): 0.98 (0.92 to 1.05) and visceral fat: OR (adjusted): 1.00 (0.90 to 1.11)). The interaction terms were also not significant.

**Discussion/Conclusions**: Our preliminary results do not support a significant role for adiposity and its interaction with generalized inflammation as factors associated with pain and synovitis when adjusted for potential confounders.

## Investigating Topical Capsaicin-Induced Pain Responses in Young Healthy Adults: Associations with Skin Temperature, Hyperalgesia, and Allodynia

Adrien Nourry^1, 2^, Evelyne Carbonneau^2, 3^, Cécile Smeesters^2, 3^, Matthieu Vincenot^2^, Flore Leblanc^1, 2^, Guillaume Léonard^1, 2^

^1^Université de Sherbrooke, School of Rehabilitation, Faculty of Medicine and Health Sciences, Sherbrooke (Qc), Canada., ^2^Université de Sherbrooke, Research Center on Aging, CIUSSS de l’Estrie-CHUS, Sherbrooke (Qc), Canada, ^3^Université de Sherbrooke, Department of Mechanical Engineering, Faculty of Engineering, Sherbrooke (Qc), Canada

**Introduction**: Topical application of capsaicin has been used in research for many years as an experimental pain paradigm. In addition to pain, capsaicin application is often associated with other signs and symptoms, including increased skin temperature, hyperalgesia and allodynia. The aim of the present study was to explore the relationship between pain intensity and these other manifestations following topical capsaicin application on the lower back and on the knee in young healthy adults.

**Methods**: Capsaicin cream (1%) was applied on the lower back and on the dominant knee of 18 healthy adults (36 ± 16 yrs) in 2 randomized sessions. Pain (0–10 visual analog scale), skin temperature (laser thermometer), hyperalgesia (Von Frey monofilaments), and allodynic area (presence/absence) was measured before and after the application of capsaicin. Nonparametric analyses were used to assess the associations between pain intensity, skin temperature, hyperalgesia and allodynia. Statistical significance was set at p < .05.

**Results**: For the lower back, Spearman’s analyses revealed that pain intensity was correlated with both hyperalgesia (r = −0.68, p = .02) and skin temperature (r = −0.65, p = .04). In contrast, Mann–Whitney tests revealed no significant difference in pain intensity between participants with or without an allodynic zone. No significant results were observed for the knee, either for skin temperature, hyperalgesia or allodynia.

**Discussion/Conclusions**: These results suggest that pain intensity is more strongly associated with skin temperature and hyperalgesia than with allodynia, particularly over the lower back. These site-dependent associations, absent at the knee, suggest that each manifestation reflects a distinct aspect of the response to capsaicin.

## Falling into the Holes: Uncovering Gender Bias and Diagnostic Pitfalls in Fibromyalgia

Tali Sahar^1^, Maria Verner^1^, Adi Shraibman^2^, Alessandra Balleine^1^, Sylvie Toupin^1^, Sabrina Mitrovic^1^, M. Gabrielle Pagé^3^, Vibhu Vibhu^1^, Nuzhat Nipa^1^, Amir Minerbi^4^, Yoram Shir^1^, Maayan Ben-sasson^1^

^1^ The Alan Edwards Pain Management Unit (AEPMU), Montréal General Hospital, Montreal, Quebec, Canada, ^2^Computer Science department at the Tel Aviv-Yaffo Academic College, ^3^Department of Anesthesiology and Pain Medicine, Faculty of Medicine, Université de Montréal, Montreal, Quebec, Canada, ^4^Rappaport Faculty of Medicine, Technion-Israel Institute of Technology, Haifa

**Introduction**: Fibromyalgia is difficult to diagnose due to overlapping symptoms and a lack of specific biomarkers. Leading to frequent misdiagnoses and treatment delays. This study explores factors contributing to diagnostic discrepancies in fibromyalgia.

**Methods**: A prospective cohort of 137 chronic pain patients. Participants underwent medical evaluations and completed the FSDC, CSI, EuroQoL-5D, painDETECT, PROMIS-29, PCS-6, and IPAQ questionnaires. Physician and questionnaire-based diagnoses were compared. Diagnostic discrepancies were analyzed using basic statistics, feature selection, k-means clustering, and learning models.

**Results**: Diagnoses revealed 17 true positives and 87 true negatives for fibromyalgia (FM). There were 24 false negatives – identified by the questionnaire but not by physicians – and 9 false positives – diagnosed by physicians but not by the questionnaire. Significant diagnostic discrepancies included:

- Gender bias: 7 out of 38 men were diagnosed by the questionnaire, but only 1 by physicians. The false negative rate for men (100%), significantly higher than for women (50%) (p = .0295).
- Frequency of widespread pain vs. WPI: Item 9 of the CSI correlated with the questionnaire FM diagnoses. Eleven of the 24 false negatives rated this item as 1–2 on a scale of 0–4, indicating their pain is not often widespread.
- IBS bias: 58.34% of the twelve participants with IBS met FM criteria by the questionnaire, but only 8.3% were diagnosed by physicians.

**Discussion/Conclusions**: This study highlights significant diagnostic challenges in fibromyalgia, including gender bias and IBS-related biases. These findings underscore the need for improved diagnostic criteria (such as including frequency of widespread pain) and greater physician awareness for fibromyalgia.

## The Effects of COVID-19 on Chronic Pain Management for Patients with Use-Disorders: A Qualitative Study

Thomas Samson^1^, Katija Bonin^2^, Alexandra Oprea MacNeil^1^, Karim Mukhida^1, 2^

^1^ Faculty of Medicine, Dalhousie University, Halifax, Nova Scotia, ^2^Department of Anesthesiology, Pain Management and Perioperative Medicine, Halifax, Nova Scotia

**Introduction**: The COVID-19 pandemic significantly disrupted care for individuals managing chronic pain and addictions, populations highly dependent on in-person services. In an effort to limit viral spread, chronic pain clinics across the country transitioned to virtual phone and telehealth consultations and liaised with community pharmacists to continue to provide safe prescription management. This study explores the pandemic’s impact on chronic pain care for patients with comorbid addictions.

**Methods**: A qualitative study using semi-structured interviews was conducted with patients and providers from the Pain Management Unit (PMU) at Victoria General Hospital, Halifax. The interviews were analyzed using thematic analysis, with three researchers reaching consensus on the findings.

**Results**: Thirteen patients with comorbid chronic pain and addictions and seven providers were interviewed. The following themes were identified: 1) effects of pandemic on pain, 2) social supports, 3) street drug use during COVID-19, 4) phone advantages, 5) phone disadvantages, 6) changes to opioid prescribing during the COVID-19 pandemic.

**Discussion/Conclusions**: Patients with chronic pain and addictions adapted to telephone- based care during the pandemic. Although this approach enabled continuity of medication management, it had notable advantages and challenges.

## Trauma History and Mental Health Problems Among Persons With Chronic Pain: Exploration Using a Symptom Network Approach

Gabriella Spiegler^1^, Yilin Zhang^2^, Louis-Phillipe Langlois^3^, Nesrine Mesli^4^, Maria Verner^5^, Sabrina Mitrovic^5^, Mark Ware^5, 6^, M. Gabrielle Pagé^7^, Leon Tourian^5, 8^, Marc Martel^5, 9, 10^

^1^Department of Epidemiology, Biostatistics, and Occupational Health, McGill University, ^2^Faculty of Medicine and Health Sciences, McGill University, ^3^Integrated Program in Neuroscience, McGill University, ^4^Department of Psychology, McGill University, ^5^Alan Edwards Pain Management Unit, McGill University Health Center, ^6^Department of Family Medicine, McGill University, ^7^Department of Anesthesiology and Pain Medicine, Université de Montréal, ^8^Department of Psychiatry, McGill University, ^9^Faculty of Dental Medicine and Oral Health Sciences, McGill University, ^10^Department of Anesthesiology, McGill University

**Introduction**: Trauma histories are known to be prevalent among patients with chronic pain. Trauma and chronic pain both increase vulnerability to mental health disorders, which are common in this population. Anxiety and depression are particularly prevalent among patients with chronic pain, and they are usually manifested by a constellation of neurovegetative, cognitive, and affective symptoms. However, the effects of trauma on patients’ mental health symptoms remains unclear.

The first objective was to examine interrelations between neurovegetative, cognitive, and affective symptoms among patients with chronic pain. We then explored whether the occurrence and relative centrality (i.e., importance) of specific symptoms were influenced by patients’ trauma history.

**Methods**: Patients (n = 786) underwent a structured clinical interview based on the Diagnostic and Statistical Manual of Mental Disorders (DSM-5) that assessed the presence/absence of symptoms associated with Generalized Anxiety Disorder (GAD) and Major Depressive Disorder (MDD). The interview also included questions assessing lifetime trauma history and active (i.e., past year) symptoms of Post Traumatic Stress Disorder (PTSD).

**Results**: Network analyses using the Bootnet package in R indicated that trauma history was most strongly associated with feelings of guilt and restlessness (edge weights: 0.30–0.41). Analyses also revealed that the relative strength centrality of sleep problems was significantly higher among those with a trauma history.

**Discussion/Conclusions**: Our findings provide new insights into the influence of trauma on the occurrence and relative importance of specific cognitive-affective and neurovegetative symptoms among patients with chronic pain.

## Efficacy of the Discretized Analog Scale (DISCAN) for Assessing the Subjective Experience of Pain Intensity Levels During Post-Operative Care in Older Adults

Elizabeth Woodford^1^, Michael Bautista^1, 2^, Roberta M. DiDonato^1, 2^, Geoff Warden^1^, Mark Howells^1^

^1^Faculty of Medicine, Memorial University of Newfoundland, Canada, ^2^Aging Research Center – Newfoundland and Labrador, Grenfell Campus, Memorial University, Canada

**Introduction**: This study investigates whether the Discretized Analog Scale method (DISCAN), a novel clinician-administered scale, is a reliable, accurate and sensitive measure of pain intensity for older adults by correlating it with other established pain intensity scales (the Numeric Rating Scale (NRS) and Visual Analog Scale (VAS).

**Methods**: Patients 40+ years old undergoing postoperative recovery from hip or knee surgeries were recruited and assessed in an outpatient physiotherapy department. We examined the pain scales’ reliability and sensitivity for identifying changes in pain (i.e., effect sizes) by comparing pain-intensity scores before the interventions to pain-intensity after interventions.

**Results**: The findings indicated that there were no effects of age on pain-intensity scores. The reliabilities analyses, intra-class correlation coefficient (ICC), revealed Cronbach alpha values between 0.79–0.95, indicating consistent and reliable pain intensity measurements by DISCAN. Each pain scale (NRS, VAS and DISCAN) demonstrated excellent internal consistency (all p < .001). The sensitivity of the three pain scales revealed similarly significant decreased pain intensity scores when effect sizes were large (r = 0.53–0.55) between postphysiotherapy and the presurgery; and medium (r = 0.41–0.46) between postphysiotherapy and immediately postoperatively (p < .001). However, when effect sizes were smaller (r = 0.23–0.25) comparing 1st −3^rd^ physiotherapy sessions, only the DISCAN pain scale scores identified the decrease in pain intensity across the three physiotherapy treatments. Further, only DISCAN identified two invalid/inconsistent scores.

**Discussion/Conclusions**: The DISCAN measure is a reliable measure of postoperative pain and may be more sensitive than traditionally used measures such as the NRS and VAS.

## EducationMapping Cancer Pain Training Provided to Family Physicians: A Review of Pre-Clerkship Programs Offered By Quebec’s Medical Schools

Charles-Antoine Auger^1, 2, 3^, Maud Bouffard^1, 3^, Marie-Ève Cimon^1, 3, 4^, Marie-Josée Hammond^5^, Maxime Bouchard^5^, Nicole Alberts^6^, Gaëlle Gloaguen^3, 7^, Aline Hajj^1, 3, 8^, Marie-Christine Houde^9, 10, 11^, Hermann Nabi^1, 3, 7^, Jordi Perez^12, 13^, Lucia Gagliese^14, 15, 16^, Lynn Gauthier^1, 3, 7^

^1^L’Équipe de recherche Michel-Sarrazin en oncologie psychosociale et soins palliatifs, ^2^School of Psychology, Université Laval, ^3^CR-CHU de Québec-Université Laval, Oncology Division, ^4^Faculty of Nursing Sciences, Université Laval, ^5^Patient Author, ^6^Department of Psychology, Concordia University, ^7^Faculty of Medicine, Université Laval, ^8^Faculty of Pharmacy, Université Laval, ^9^CSSS de Saint-Jérôme, ^10^Faculty of Medicine, Université de Montréal, ^11^Centre de gestion de la douleur, Center Hospitalier Universitaire de Montréal, ^12^Department of Anesthesiology, McGill University, ^13^Alan Edwards Pain Management Unit, ^14^School of Kinesiology and Health Science, York University, ^15^Department of Anesthesia and Pain Management, Sinai Health Systems, ^16^Department of Anesthesia and Psychiatry, University of Toronto

**Introduction**: Up to 40% of patients experience pain after cancer treatment, often managed by family physicians, who report knowledge gaps. This study examines cancer pain (CP) content in Quebec’s Faculties of Medicine (FACMED) pre-clerkship curricula within a larger training needs assessment.

**Methods**: FACMED were invited to share syllabi/course outlines. Keywords were derived from general pain and CP literature, classification systems, and clinical guidelines. Course objectives were classified as pain-related if they included general pain keywords and CP- related if pain and cancer keywords appeared. CP objectives were mapped onto IASP Interprofessional Pain Curriculum and CanMEDS competencies. Courses with CP content were reviewed using Harden’s Curriculum Framework.

**Results**: Among four FACMED, one declined, one agreed but provided no data, and two participated. FACMED-1 shared a document summarizing preclerkship pain topics, identifying 11/31 (35.5%) as potentially cancer-related, with one (3.2%) focused on CP. Lack of syllabi prevented further analysis. FACMED-2 included pain-related objectives in 16/33 courses (48.5%; 109/5204 [2.1%] of objectives), with CP objectives in two courses (6%; 0.2% of objectives). Objectives addressed IASP’s multidimensional nature of pain (67%), clinical conditions (67%), and assessment (33%), but not management. Most (67%) aligned with CanMEDS’ cognitive domain, 33% with psychomotor, and none with affective. Review of supplementary material from 20/33 courses revealed no CP content in these two courses, but CP content in three others without CP objectives, each containing one slide on CP management for different cancers.

**Discussion/Conclusions**: Findings suggest limited CP integration in Quebec FACMED preclerkship curricula and highlight potential areas for improvement.

## Optimizing Effectiveness and Engagement in Self-Education for Veterans Managing Chronic Pain: A Qualitative Descriptive Study

Ronessa Dass^1^, Stephanie Di Pelino^1^, Lisandra Almeida de Oliveira^1^, Lisa Carlesso^1^, Luciana Macedo^1^, Laura Katz^1^, Tara Packham^1^

^1^McMaster University

**Introduction**: Veterans may have challenges accessing and engaging in pain management. Self-education is an accessible and active form of engagement that can support existing pain management and improve pain outcomes. However, Veterans may experience barriers to self-education (e.g., navigating trustworthy resources). There is a need to explore Veterans’ perception on self-education to support development of future programs.

**Aim**: To describe how Veterans engage with self-education for pain management.

**Methods**: This study uses a qualitative descriptive methodology and constructivist theoretical lens. Participants were recruited from the Chronic Pain Center of Excellence for Canadian Veterans’ network. Data was generated using semistructured individual interviews. Results were analyzed using content analysis informed by the Template for Intervention Description and Replication checklist.

**Results**: A total of 16 Canadian Veterans (M = 13, F = 3) participated in this study.

Veterans described self-education as a continuous process; equipping them to seek innovative strategies to 1) manage their health and 2) be actively involved in their care. Veterans’ military identity may be an asset to self-education, encouraging them to actively find solutions and attain goals. Conversely, that same identity may hinder self-education by creating difficulty seeking support or trusting resources. Veterans believed that self-education programs benefit from the support of healthcare professionals and include aspects of military identity, engage the entire family, and include strategies to identify credible resources.

**Discussion/Conclusions**: This study summarizes Veterans’ description of self-education and insights on how self-education strategies can be tailored for Veterans and their families.

## Sharing Knowledge Through Education: Pain BC’s Initiatives for Healthcare Providers

Alexis Gonzalez^1^, Helena Daudt^1^

^1^Pain BC

**Introduction**: The literature suggests that healthcare providers (HCPs) face challenges in the assessment and management of chronic pain, including inadequate training. To address the training gap, Pain BC developed online asynchronous courses for HCPs as part of a suite of educational programs.

**Methods**: Pain Foundations-Basic is a 5-hour course to improve foundational knowledge of pain science, assessment, and management. Moving through Pain is a 4 -hour course to enable learners to better support people living with pain to move more. To evaluate both programs we surveyed learners before and after the course.

**Results**: Since we launched the current version of Pain Foundations in January 2024, 90 HCPs have completed both assessments. Comparison of pre and post scores through a paired-sample t-test suggests that participants felt more confident about their understanding of pain-related topics after attending the course (presurvey – M = 45.89, SD = 8.95; postsurvey – M = 58.91, SD = 5.98), t(93) = −15.01, p < .001). We conducted a similar analysis for Moving through Pain (launched in October 2024, 132 registrants, 28 completed both assessments) and obtained similar results (presurvey -M = 24.5, SD = 6.44; postsurvey -M = 31.2, SD = 4.76), t(23) = 4.87,p < .001 t(23) = 4.87, p < .001, t(23) = 4.87,p < .001.)

**Discussion/Conclusions**: These preliminary results suggest that structured, evidence-based education may enhance chronic pain understanding among HCPs. Although answering both assessments is a requirement for the completion of the courses, only about one-third of participants completed both. We are currently exploring ways to increase survey response rate.

## The Impact of Project ECHO – A Telementoring Education Program – On Quality of Care: An Exploratory Qualitative Study

Helena Daudt^1^, Karen Ng^2^, Najam Mian^3^, Roland Fletcher^4^, Alexis Gonzalez-Donoso^1^

^1^Pain BC, ^2^Fraser Health, ^3^CPRI, ^4^UBC

**Introduction**: Project ECHO (Extension for Community Healthcare Outcomes) is a telementoring model designed to enhance provider capacity and foster a community of practice. The British Columbia ECHO for Chronic Pain Program (BC ECHOCP) was launched in 2019. However, low response rates to feedback surveys have hindered comprehensive program evaluation. As the first step to developing a new evaluation strategy for the BCECHOCP, we chose to use the British Columbia Health Quality Matrix as a framework to explore the impact of the program on quality of care and identify indicators to measure this impact.

**Methods**: Using a case study approach, we analyzed data from ECHO sessions and conducted semistructured interviews. The data was examined through thematic analysis.

**Results**: We generated three themes through semantic analysis: “I hear you and I will meet you where you are at,” “The gently guided path,” and “Sharing is caring.” A fourth theme, “Balancing wishes and reality is tough,” emerged from latent analysis.

The primary participants in the BC ECHOCP were manual therapists and medical professionals. The program successfully fostered all five dimensions of quality care we studied: respect, safety, accessibility, appropriateness, and effectiveness. We identified eight indicators of ECHO’s impact on quality of care.

**Discussion/Conclusions**: The Matrix provided actionable insights into the BC ECHOCP, which can be used as indicators to measure its impact on quality of care and guide future research directions.

## Why the Zebra? Improving Patient Education and Engagement at the GoodHope Ehlers-Danlos Syndrome Clinic

Tina Do^1^, Anna Lomanowska^1, 2^, Laura McGillis^1^, Nimish Mittal^1, 2, 3^, Hance Clarke^1, 2, 3^

^1^GoodHope Ehlers Danlos Syndrome Clinic, Toronto General Hospital, University Health Network, Toronto, ON, ^2^Department of Anesthesia and Pain Management, University Health Network, Toronto, ON, ^3^Department of Anesthesiology and Pain Medicine, University of Toronto, Toronto, ON

**Introduction**: The GoodHope Ehlers-Danlos Syndrome (EDS) Clinic at Toronto General Hospital is the only clinic of its kind in Canada treating patients with EDS and Generalized Hypermobility Spectrum Disorders (G-HSD). The clinic takes a multidisciplinary approach to the treatment of these complex disorders, which includes disease self-management support and patient education. To enhance patient education and engagement at the clinic, we conducted a quality improvement project to develop educational resources that support patients through their multidisciplinary treatments.

**Methods**: We developed a comprehensive online educational module based on content from EDS literature and information provided by expert clinicians. The interactive module guides patients through the services offered at the GoodHope clinic and provides information about EDS and G-HSD, expectation for care, and self-management approaches. The module was created using Storyline360 software and encompasses interactive features that allow patients to select their learning pathway and access the information that is most relevant to them. This approach supports the unique experience of EDS/G-HSD patients who often have different symptoms, comorbidities, and outcomes (all zebras have unique stripes).

**Results**: The development of the module involved an iterative approach with feedback sought from clinicians and patients throughout the process. Patients and staff reviewed the preliminary versions of the module and provided feedback on the educational content, design, and user experience through questionnaires and think-aloud interviews.

**Discussion/Conclusions**: The module is hosted on the GoodHope clinic website and provides a centralized resource that guides patients through their multidisciplinary care journey while supporting their self-management efforts.

## Enhancing Pain Management Education: Exploring Instructional Design of a Competency- Based, Interprofessional Certificate Program

Lisa Jasper^1^, Kim Dao^2^, Bernadette Martin^1^

^1^University of Alberta, ^2^Tufts University

**Introduction**: Chronic pain affects approximately 20% of Canadians, contributing to significant suffering, economic costs, and healthcare challenges. Despite its prevalence, there is a gap in pain education for healthcare providers, both pre- and post-licensure, limiting access to effective, interprofessional pain management. To address these gaps, the University of Alberta developed the Certificate in Pain Management (Certificate), an online, competency-based, interprofessional continuing professional development (CPD) program. This study aims to explore feedback from Certificate participants in the first 12 years of the program regarding learning activities and instructional design of the courses.

**Methods**: Participants included individuals who enrolled in at least one course within the Certificate from 2010–2022. A comprehensive survey was distributed, capturing demographic details, and feedback on learning activities and instructional design of the three courses in the Certificate program.

**Results**: Out of 238 surveyed participants, 29 (12.2%) responded, representing eleven health professions, both rural and urban locations, and five provinces/countries. Online presentations, individual assignments, and group assignments received positive feedback with 86%/85%/80% agreeing or strongly agreeing that they were effective for learning. Participants rated the instructional quality (93%), guest lecturer’s expertise (93%), and administrative support (89%) highly. Improved interprofessional collaboration (89%) and understanding of professional roles (86%) were also reported.

**Discussion/Conclusions**: The online CPD Pain Certificate provides accessible, postgraduate education to individuals from diverse professions and geographical areas, ensuring evidence and competency-based learning opportunities regardless of location. Survey data highlights effective instructional design and learning activities fostering interprofessional collaboration.

## D’un cercle vicieux à un cercle vertueux ». Comment la motivation à l’activité physique se transforme dans le cadre d’une intervention thérapeutique non médicamenteuse axée sur l’affiliation sociale et l’expérience vicariante auprès de personnes vivant avec la douleur chronique.

Caroline Guay^1^, Sylvie Lafrenaye^1^, Guillaume Léonard^1^, Nathalie Clément^1^, Martine Bordeleau^1^, Jean-Francois Desbiens^1^, Carole Paris^2^

^1^Université de Sherbrooke, ^2^patiente-partenaire

**Introduction**: Les bénéfices d’un mode de vie actif sont reconnus, même auprès d’une population souffrant de douleurs chroniques. Pourtant, il peut être difficile de demeurer actif lorsque la douleur occupe une place importante dans son quotidien. Le projet Versant AKOR 2024 visait à accompagner des personnes sédentaires vivant avec la douleur chronique, suivies au Center d’expertise en gestion de la douleur chronique CHUS (Sherbrooke, Canada) dans la réalisation d’un défi où elles devaient faire individuellement de l’activité physique tout en cumulant ensemble le nombre de kilomètres représente la traversée du Canada complétée parallèlement par les aventuriers AKOR 2024 avec qui elles ont été mises en contact.

**Objectifs**: (1) Comprendre comment se transforme la motivation à l’activité physique des personnes à travers cette intervention où l’activité physique réalisée de façon autonome est proposée sous forme de défi collectif, axée sur l’affiliation sociale et l’expérience vicariante. (2) Observer quelles sont les retombées d’un tel projet sur l’adoption d’un mode de vie plus actif, et l’amélioration de la qualité vie, cinq mois après la fin du projet clinique.

**Méthodologie**: Recherche-intervention à caractère longitudinal et qualitatif pendant une année. Des cueillettes de données répétés ont permis de documenter l’évolution dans le temps du phénomène étudié et l’impact sur le temps actif hebdomadaire. Des questionnaires ont été administrés, des entretiens individuels ont été réalisés et des rencontres de groupe ont été observées.

**Résultats**: Les résultats préliminaires indiquent que 11/12 participants ont amélioré leur motivation à bouger et le temps actif hebdomadaire. Sur le plan scientifique cette étude pourra contribuer à une meilleure compréhension du rôle de l’affiliation sociale et l’expérience vicariante sur la motivation à l’activité physique et sur la qualité de vie.

**Discussion/Conclusion**: Une approche axée sur la connexion sociale pourrait contribuer à l’adoption d’un mode de vie plus actif et durable auprès des personnes vivant avec la douleur chronique.

## Comorbid Chronic Pain and Mental Health: Assessment of Clinical Priorities Among Healthcare Providers in Ontario

Tobi Oyedele^1^, Anna Lomanowska^1^, P. Maxwell Slepian^1, 2, 3^, Brittany N. Rosenbloom^2, 3, 4^, Tania Di Renna^2, 3, 4^, Joel Katz^1, 2, 3, 5^, Jennifer Stinson^3, 6, 7^, Hance Clarke^1, 2, 3^, Jeffrey Wieskopf^1, 3, 8^

^1^Transitional Pain Service, Department of Anesthesia and Pain Management, Toronto General Hospital, University Health Network, Toronto, ON, ^2^Department of Anesthesiology and Pain Medicine, University of Toronto, Toronto, ON, ^3^University of Toronto Center for the Study of Pain, University of Toronto, Toronto, ON, ^4^Toronto Academic Pain Medicine Institute, Women’s College Hospital, Toronto, ON, ^5^Department of Psychology, York University, Toronto, ON, ^6^Child Health Evaluative Services, The Hospital for Sick Children, Toronto, ON, ^7^Lawrence S. Bloomberg Faculty of Nursing, University of Toronto, Toronto, ON, ^8^Department of Psychiatry, Toronto General Hospital, University Health Network, Toronto, ON

**Introduction**: Chronic pain and mental health disorders frequently co-occur, increasing patients’ risk for morbidity and mortality. However, tertiary pain clinics often lack adequate expertise in mental health treatment, while practitioners in psychiatry and psychology have limited expertise in pain management. This practice gap hinders timely and effective intervention for this complex patient population. The current project aims to assess healthcare providers’ understanding and needs regarding comorbid chronic pain and mental health treatment to develop educational resources to enhance their clinical skills and confidence.

**Methods**: In a quality improvement initiative, we disseminated a needs assessment survey to chronic pain clinicians across Ontario via professional organizations and tertiary pain clinics. The survey collected demographic information, assessed clinicians’ experience, confidence, and training needs in treating comorbid chronic pain and mental health conditions, and included open-ended questions on clinical priorities. Findings will inform the development of an e-learning module to support this area of practice.

**Results**: Thus far, 54 clinicians from across healthcare professions and geographical regions of Ontario treating adult and pediatric patients completed the survey. Preliminary findings show that experience and confidence in treating comorbid chronic pain and mental health concerns varied, with lower confidence in managing comorbid pain and trauma. Clinicians highlighted a lack of resources to support mental health treatment in patients with comorbid diagnoses and a need for further training. Data collection is ongoing.

**Discussion/Conclusions**: Addressing the practice gap in comorbid chronic pain and mental health treatment requires enhancing provider confidence and skills through targeted education and clinical resources.

## Insights from Knowledge Translation Initiatives at the Transitional Pain Service for Patients and Providers

Anna Lomanowska^1^, Sabrina Zhu^1^, Christina Choo^1^, Dora Y. Wang^1^, Rabia Tahir^1, 2^, Maxwell Slepian^1, 3^, Joel Katz^1, 3, 4^, Hance Clarke^1, 3^

^1^Transitional Pain Service, Department of Anesthesia and Pain Management, Toronto General Hospital, University Health Network, Toronto, ON, ^2^Michael G. DeGroote School of Medicine, McMaster University, Hamilton, ON, ^3^Department of Anesthesiology and Pain Medicine, University of Toronto, Toronto, ON, ^4^Department of Psychology, York University, Toronto, ON

**Introduction**: Effective chronic pain treatment requires a multidisciplinary approach based on the biopsychosocial model. However, implementing this approach in pain clinics is challenging, as it requires additional resources and expertise. Pain clinics that successfully adopt this model can serve as examples for others. The aim of this quality improvement project is to provide insights from the knowledge translation initiatives at the Transitional Pain Service (TPS) at Toronto General Hospital, a multidisciplinary program for patients at risk of developing chronic postsurgical pain.

**Methods**: Knowledge translation initiatives at the TPS were geared toward both healthcare providers and patients. The initiatives were approved by the institutional quality improvement committee and followed the cyclical Knowledge to Action (KTA) framework, which involves identifying knowledge gaps, adapting knowledge to the local context, and monitoring knowledge use.

**Results**: A needs assessment among TPS patients and healthcare providers identified knowledge gaps and guided the creation of knowledge products. The clinic developed a website (www.transitionalpainservice.ca) to disseminate resources. Initiatives for healthcare providers focused on education about multidisciplinary care and a guide for establishing a TPS. Patient initiatives included information on the website and two online learning modules. Web analytics tracked resource usage and follow-up surveys and interviews assessed engagement.

**Discussion/Conclusions**: Awareness and acceptance of the multidisciplinary pain care model by both providers and patients are critical for its implementation. The TPS’s ongoing knowledge translation efforts are essential in promoting this model, and further evaluation will assess their impact.

## The Alberta Virtual Pain Program: Evaluation of Programming for Healthcare Providers

Elena Lopatina^1, 2^, Susan Sobey-Fawcett^2^, Tina Hoang^1, 2^, Magali Robert^1, 2, 3^

^1^University of Calgary, ^2^Primary Care Alberta, ^3^Alberta Health Services

**Introduction**: The Alberta Virtual Pain Program (AVPP) aims to close existing gaps in care for patients living with chronic pain in Alberta. In November 2024, the AVPP delivered its inaugural webinar for healthcare providers (HCPs). The main objective was to provide HCPs with implementable, pragmatic information to support people with chronic pain by (1) exploring chronic pain as a chronic condition; (2) sharing key messages on chronic pain management tailored for HCPs; and (3) providing an overview of the AVPP.

**Methods**: This evaluation focused on webinar attendance and participant feedback. Registration numbers were obtained via the online registration platform, and attendee numbers were recorded through Zoom. Participant feedback was collected via an anonymous REDCap survey, which included closed- and open-ended questions. Quantitative data were analyzed using descriptive statistics, and qualitative data were thematically analyzed.

**Results**: A total of 224 individuals registered for the webinar, with 122 attending. Among the attendees, 94 participants completed the postwebinar survey. Over 95% of respondents found the webinar content relevant and the delivery format appropriate. Additionally, more than 93% reported they planned to implement learnings from the webinar into their practice. Qualitative feedback underscored the value of the key messages and actionable strategies provided. Participants expressed interest in additional webinars and having more engagement opportunities.

**Discussion/Conclusions**: This evaluation demonstrates that the HCP webinar was a valuable component of AVPP’s programming. Its success confirms the need for and interest in chronic pain management strategies. Participant feedback will inform the development and refinement of future webinars.

## Pain curricula across Canadian prelicensure healthcare professions: Comparison with IASP curriculum-specific guidelines

Hansel Lui^1^, Norman Buckley^2^

^1^Department of Biochemistry and Biomedical Sciences, McMaster University, ^2^Department of Anesthesia, Faculty of Health Sciences, McMaster University

**Introduction**: Previous studies have shown a large deficiency in pain content in undergraduate healthcare education. Revisions to undergraduate healthcare curricula have since been made to incorporate more pain education. The Canadian Pain Care Forum (CPCF) invited speakers from various healthcare disciplines to present pain content within their curricula to assess the degree to which changes have been made. The objective of this study was to compile healthcare curricula pain content from talks given between 2020–2023 and compare to IASP curriculum-specific guidelines and curriculum observations that had been reported between 2005–2013.

**Methods**: Presentations on pain content in healthcare curricula were summarized and the content mapped onto the International Association for the Study of Pain (IASP) curriculum guidelines to determine how closely the education follows the IASP guidelines.

**Results**: The nursing curriculum had the highest correspondence with IASP curriculum guidelines (89.5%), while the physiotherapy curriculum had the lowest concordance with the IASP curriculum guidelines (37.5%). The medicine curriculum touched on 64.5% of the IASP guidelines, the occupational therapy curriculum touched on 84.5% of the IASP guidelines, and the pharmacy curriculum touched on 58.3% of the guidelines.

**Discussion/Conclusions**: Although there has been a significant increase in the amount of pain content covered in each institutions’ curricula, there are still significant gaps. There is active curriculum revision underway [physiotherapy]. Although this is not a systematic curriculum review, it does provide an update to previous observations. More work needs to be done to increase the specificity of pain content in national accrediting bodies.

## Rikki’s Invisible Pain: Collaborative co-design of a children’s book and Grade 3–4 curriculum guide for elementary schools

Karen Juckes^1^, Megan Hewson^2^, Alex Schmidt^3^, Nikki Cooke^4^, Ross McCreery^4^, Erin Beckwell^5^, Krista Baerg^1, 2^, Susan Tupper^1, 2^

^1^University of Saskatchewan, ^2^Saskatchewan Health Authority, ^3^Regina Public School Division, ^4^Person with Lived Experience, ^5^University of Regina

**Introduction**: Youth living with chronic pain are more likely to experience loneliness and social isolation, and report feeling misunderstood by peers due to school and social absences, and lack of understanding about pain[TS1] . A children’s book and curriculum guide for teachers in elementary schools and hospital settings were co-designed to help raise awareness and guide conversations with youth living with pain, their peers, teachers, and family caregivers.

**Methods/Results**: As part of the Improving Pain in Saskatchewan (IPSK) Research project, a six- person working group consisting of multidisciplinary healthcare providers, people with lived experience, elementary school and hospital-based teachers, and researchers co-designed and wrote the resources. A series of five relationship-building meetings were held to establish a common understanding of the story characters, plot, and key issues to include. Iterative writing, feedback gathering, member checking, and revisions took place over 24 months, ending January 2025. The co-design process was informed by a participatory action research paradigm in which experiential knowledge and leadership was prioritized and all team members were considered co-creators of knowledge with equitable contributions to the writing and revising process.

**Discussion/Conclusions**: Rikki’s Invisible Pain is about a young student who lives with complex pain. Rikki is unable to finish a play-day relay race and feels invisible and misunderstood. The teacher supports Rikki to share information about their pain with classmates. Themes of empathy, inclusivity, validation, identity and resiliency are central to Rikki’s story. The children’s book and curriculum guide support children’s education about complex pain.

## Empowering Knee Osteoarthritis Self-Management: Testing Reliability of the Patient Education Materials Assessment Tool Through the Patient Lens

Tarin Moni^1^, Luciana Macedo^1^, Lisa Carlesso^1^, Tara Packham^1^

^1^McMaster University

**Introduction**: Knee osteoarthritis (KOA) is a chronic condition requiring effective self-management practices. People with lived experience (PwLE) often seek additional guidance online, however, are uncertain about resource reliability. The Patient Education Materials Assessment Tool (PEMAT) was developed to help clinicians and the public determine the quality of online educational resources (EdRs) by rating their understandability and actionability, however, reliability has yet to be studied exclusively with PwLEs.

We aimed to assess the overall reliability of the PEMAT-P (print version) when used by PwLE to rate online print EdRs for KOA.

**Methods**: Participants with knee pain (n = 21) rated six pre-selected EdRs using the PEMAT-P. We assessed interrater reliability using percent agreement and intraclass correlation coefficient (ICC), and internal consistency using Cronbach’s alpha. Participants also completed a satisfaction survey to assess the usability of the PEMAT-P.

**Results**: Of 21 respondents, mean age was 31(±11) years, mostly South Asian (48%), and with at least a Bachelor’s degree (52%). There was acceptable reliability for the total PEMAT-P scores (%: 0.73, ICC: 0.73, α: 0.79), good reliability for the understandability scores (%: 0.75, ICC: 0.86, α: 0.65), and poor-moderate reliability for actionability scores (%: 0.68, ICC: 0.49, α: 0.67). Participants found the PEMAT-P to be easy to use with almost no prior knowledge required. They also endorsed face validity and would recommend it to others.

**Discussion/Conclusions**: The PEMAT-P has variable agreement within the domains, specifically actionability. Further revisions would strengthen the tool for PwLE to use confidently to assess online EdRs.

## Sorely Needed: Immersing Undergraduate Biomedical Engineering Students to Drive Needs-Based Innovation in Chronic Pain Management

Aamna Naveed^1^, Mohammed Hasan^1^, Yasameen Ihsan^1^, Jialiang (Kevin) Hu^1^, David Diao^1^, Eugene Maida^1^, Ahilraj Siva^1^, Anna Korol^1^

^1^McMaster University

**Introduction**: Chronic Pain (CP) is a complex challenge requiring innovative biomedical technologies to address clinical needs and improve patient outcomes. The development of such solutions demands interdisciplinary collaboration among clinicians, engineers, and business experts to align technological feasibility with practical applicability. This year-long project examines prototyping in CP care through needs-driven innovation by integrating biomedical engineering students in pain clinics through McMaster University’s Innovators in Scrubs course.

**Methods**: In the clinical immersion phase, the team will attend eight clinical placements (4 hours each) at the Michael G. Degroote National Pain Center. They will observe pain management procedures and collect insights from preceptors, residents, and nurses. In the ideation and prototyping phase, students will analyze their observations and identify a single focused challenge. They will then design and build a prototype to address this challenge. Finally, in the clinical testing and iteration phase, clinicians will trial the prototype in the pain clinic, and provide feedback that will guide iterative refinements. Surveys will be conducted with both clinical preceptors and students to evaluate the perceived benefits of the project. Project will conclude by April 2025.

**Results**: Twelve weeks into the project, students identified 10 unmet clinical needs and developed a 3D-printed proof-of-concept prototype for one of these challenges. Preliminary pilot-testing indicates clinician satisfaction with the design.

**Discussion/Conclusions**: Innovators in Scrubs demonstrates how partnerships between students, clinicians, and faculty can identify critical needs and co-develop solutions for CP care, emphasizing the value of needs-driven innovation.

## Mentorat en douleur chronique, santé mentale et dépendance: Accompagner les professionnels de soins primaires dans leur pratique !

Anne Marie Pinard^1, 2^, Orlane Ballot^1, 2^, Marylie Martel^3^

^1^Cirris, ^2^université laval, ^3^Université de Sherbrooke

**Introduction**: Les problématiques de douleur, de santé mentale et de dépendance sont fréquentes et nécessitent des soins spécialisés et bienveillants. Pour répondre à ce besoin, un projet pilote de mentorat a été lancé au Québec, avec pour objectif de promouvoir des soins de qualité à travers un soutien structuré et collaboratif.

**Méthodes**: Ce programme facilite la discussion de cas complexes, le partage d’expériences et la réflexion sur la pratique clinique. Les échanges se déroulent via une plateforme en ligne, avec des rencontres toutes les six semaines. La satisfaction et les expériences des participants sont documentées au moyen de questionnaires et d’entrevues.

**Résultats**: Depuis janvier 2024, 7 mentors et 21 mentorés du RUISSSUL et RUISSS Estrie- CHUS participent au programme. Tous les groupes ont bénéficié d’une formation initiale, bien accueillie par les participants. Depuis le lancement, tous ont utilisé la plateforme de discussion, bien que leur niveau d’implication varie. Certains rapportent une grande satisfaction vis-à-vis des discussions et des apprentissages, les motivant à utilizer la plateforme régulièrement; d’autres font face à des obstacles, principalement dus à un manque de temps. Les rencontres virtuelles ont été appréciées par la majorité, avec une amélioration entre la première et la cinquième session. Des difficultés concernant la structure, la gestion du temps de parole et le choix des sujets avaient été signalées. Des ajustements ont été apportés, renforçant la cohésion et le bon fonctionnement des groupes. Un climat de confiance et une communauté respectueuse se sont instaurés, favorisant une dynamique positive et une collaboration efficace.

**Discussion/conclusion**: La satisfaction et les impacts du programme sur les mentors et les mentorés continuent d’être documentés. Ce programme vise à améliorer la qualité des soins en douleur chronique, santé mentale et dépendance, en renforçant la confiance des professionnels dans leurs compétences pour aborder ces problématiques.

## Dentistry Students’ Perceptions of Key Learnings and Change in Chronic Pain Beliefs with a Graphic Medicine Reflective Learning Activity

Susan Tupper^1, 2^, Amrinderbir Singh^2^, Erin Beckwell^3^, Dayna Fesciuc^4^

^1^Saskatchewan Health Authority, ^2^University of Saskatchewan, ^3^University of Regina, ^4^Patient Partner

**Introduction**: Chronic pain is misunderstood and undermanaged, particularly when patients lack access to appropriate health services or face intersecting stigma related to substance use, racism, or resource insecurity. Pain management is an essential component of dental practitioner training. We explored changes in chronic pain beliefs from a graphic medicine story and key learnings reported during a reflection activity with third year Doctor of Dental Medicine (DMD) students at the University of Saskatchewan.

**Methods**: This concurrent mixed-methods approach involved a one-group pretest/posttest study to examine change in students’ chronic pain beliefs using subscale-1 of the Chronic Pain Myths Scale (CPMS-1) administered immediately before and after the learning activity. Students read the graphic medicine story that depicts a pregnant person living with chronic pain who presents to the Emergency Department for pain relief but does not receive helpful answers. In small groups, participants discussed the story and generated three key learnings from the reflection activity which were analyzed with structural coding.

**Results**: Small but statistically significant improvement was observed in CPMS-1 scores (n = 39; mean change = 0.95; p = .006; 95% CI = 0.29, 1.61). Small group key learnings were clustered into three structural codes: awareness of gaps in pain management and health services, recognition of the need for patient-centered assessment and approaches to care, and empathy regarding the impacts of pain and limited healthcare.

**Discussion/Conclusions**: Findings suggest that graphic medicine stories may be useful in dental practitioner education to improve chronic pain beliefs, empathy, and awareness of the impacts of chronic pain and limited healthcare services.

## EpidemiologyChronic Pain Among Quebec Nurses During the Early Covid-19 Pandemic: A Cross-Sectional Study

Nabiha Benyamina Douma, Ph.D.^1^, Isabelle Ledoux, Inf., Ph.D.^2^, Didier Mailhot-Bisson, Inf., Ph.D.^2^, Mélanie Marceau, Inf., Ph.D.^2^, Émilie Gosselin, Inf., Ph.D.^2^

^1^Département des sciences de la santé, Université du Québec en Abitibi-Témiscamingue, Rouyn-Noranda, Québec, Canada, ^2^École des sciences infirmières, Faculté de médecine et des sciences de la santé, Université de Sherbrooke, Sherbrooke, Québec, Canada

**Introduction**: Chronic pain (CP) is a major public health problem affecting over 20% of adults in the worldwide general population. The overall prevalence of CP increased during COVID-19 pandemic. Despite the extensive research on CP during the COVID-19 pandemic, few studies explored CP among nurses. The situation of Quebec nurses remains unknown. The objective of this study was to describe CP among Quebec nurses in the context of the early COVID-19 pandemic.

**Methods**: Between July and September 2020, a web-based cross-sectional study was conducted among Quebec nurses (Canada). The survey invitation was e-mailed to 15,000 nurses randomly selected from 28,000 nurses who consented to participate in research projects. The questionnaire included items assessing presence of CP, pain location, and personal and occupational characteristics.

**Results**: A total of 1773 nurses completed the questionnaire. Mean age was 43.8 ± 12.0 years and 91.6% of participants were women. Prevalence of CP was 51.2%. Multisite CP (≥3 sites) was reported by 44.0% of CP group. The back, neck, and shoulders were the most common sites of CP (24.6%, 18.9% and 16.6% respectively).

**Discussion/Conclusions**: CP is a frequent condition among Quebec nurses during COVID-19 pandemic, with a prevalence more than 2.5 times higher than that reported in the general population (worldwide prevalence: 20%). Considering the increase of CP burden during COVID-19 pandemic, our results underline the importance for healthcare organizations to promote CP prevention and to implement workplace CP management programs. This would enhance the management of CP among nurses during similar future crisis.

## Musculoskeletal Disorders Among Quebec Prosthetists/Orthotists: What Changes During Covid-19 Pandemic?

Nabiha Benyamina Douma^1^, François Déry, Ph.D.^2^, Abir El Haouly, Inf., Ph.D.^1^

^1^Département des sciences de la santé, Université du Québec en Abitibi-Témiscamingue, Rouyn-Noranda, Québec, Canada, ^2^Travailleur social en pratique autonome, Magog, Québec, Canada

**Introduction**: Musculoskeletal disorders (MSDs) are frequent among prosthetist/orthotists (OPs) and represent a financial burden for them. The changes of MSDs symptoms and their perceived financial burden in the context of COVID-19 pandemic among Quebec OPs are yet to be studied. The objective of this study was to describe the changes of MSDs symptoms, changes MSDs-related perceived financial burden, and changes of the frequency of doctor visits and use of treatments to relieve MSDs symptoms.

**Methods**: In the context of COVID-19 pandemic, between October and November 2020, a web-based cross-sectional study was conducted among Quebec OPs (Canada). The survey invitation was distributed by the Association des orthésistes/prothésistes du Québec and shared on social media.

**Results**: The convenience sample was composed of 168 OPs. Mean age of participants was 37.0 ± 9.8 years and 75.5% were women. According of the MSDs site, between 11.4% and 39.2% of MSDS group reported a worsening of their symptoms during COVID-19 pandemic, and 87.9% reported that their symptoms were linked to their work. Compared to prepandemic context, 14.4% of MSDs group reported an increase of the frequency of doctor visits, and 24.6% reported an increase of the use of over-the-counter medications to relieve MSDs symptoms. Among MSDs group, 13.1% reported higher perceived MSDs- related financial burden.

**Discussion/Conclusions**: During the COVID-19 pandemic, MSDs have become worse and more burdensome among Quebec OPs, even in a country with a universal health care system. Our findings highlight the increase of need to promote MSDs prevention and implement workplace management programs.

## Long-term Trends in Gabapentinoid, Opioid, and Concurrent Gabapentinoid-Opioid Use in Spinal Cord Injury

Jaycee Farmer^1, 2^, Stephan Schwarz^1^, John Kramer^1, 2^

^1^The University of British Columbia, ^2^International Collaboration on Repair Discoveries

**Introduction**: Neuropathic pain affects up to 70% of individuals with spinal cord injury (SCI). While gabapentinoids are first-line therapies, opioids remain widely prescribed (35–70%) despite risks of impaired recovery, chronic pain, and adverse events, particularly with concurrent gabapentinoid-opioid use. Given evolving prescribing guidelines and safety concerns, understanding trends in gabapentinoid and opioid use in SCI is critical for optimizing pain management. Objectives: To describe overall prescribing rates and temporal trends in gabapentinoid, opioid, and concurrent gabapentinoid-opioid use in SCI from 2000 to 2022.

**Methods**: Using a cohort extracted from Population Data BC and derived by the Praxis Spinal Cord Institute, we conducted a retrospective analysis of gabapentinoid and opioid use from 2000 to 2022. Data included baseline demographics, injury characteristics, comorbidities, and pharmacy dispense records. As a secondary analysis, annual prescribing trends were modeled using year as a linear predictor.

**Results**: Among 4,536 individuals with SCI (median age: 48 years [IQR: 30–66], 74% male), 48% were dispensed both gabapentinoids and opioids, 39% opioids only, 10% neither, and 3% gabapentinoids only. The proportion of receiving both medications increased by 23%, from <1% in 2000 to 24% in 2022. Regression analysis showed a significant association between year and proportion of individuals prescribed both an opioid and gabapentinoid (OR = 1.01, p < .001).

**Discussion/Conclusions**: Gabapentinoid-opioid co-prescription has increased substantially in SCI, raising concerns about the risks of opioid-related adverse events. These findings underscore the need for further research into patient and clinical factors shaping prescribing patterns to inform personalized pain management strategies.

## The Emotional Burden of Pain: Global Pain Index Results from over 18,000 Participants

Peter Gao^1^, Richard Petruschke^1^, Connor Geddis^1^, Carmen Cheung^1^, Thomas Cho^2^

^1^Haleon, ^2^University of Toronto Leslie Dan Faculty of Pharmacy

**Introduction**: Pain is a universal experience, affecting 91% of people globally and impacting daily activities, well-being, and quality of life (QoL). While physical aspects of pain have been extensively studied, there is a gap in understanding the emotional and psychological impacts.

**Methods**: The Haleon ‘Global Pain Index (GPI)’ is a longitudinal social study designed to understand the experiences of people in pain, surveying 18,097 participants across 18 countries, including Canada and the US. Conducted regularly and validated by external experts in pain management, the GPI aims to provide comprehensive insights on pain, with a focus on the emotional and psychological impact.

**Results**: The most recent GPI survey (2023) showed that while the physical aspects of pain have remained relatively stable, the emotional and psychological impact on QoL has increased by 25% since 2014. These findings suggest that pain is now driving a “new epidemic of loneliness,” increasing the gap in awareness and support for emotional and psychological well-being. There is a significant relationship between pain and loneliness, with 31% of people reporting “serious loneliness” due to pain. The GPI provides extensive insight into how pain drives social withdrawal, with many individuals feeling judged, unsupported, and stigmatized, and suggests how these effects can be addressed.

**Discussion/Conclusions**: Emotional and psychological effects of pain are creating a sense of loneliness globally as defined by the GPI. The GPI highlights the need for enhanced training and resources for healthcare professionals to address these aspects of pain, to deliver more comprehensive care for pain sufferers.

## CircaPain: Examining Interindividual Differences in Chronic Pain Through Time-Dependent Variability

Hailey G.M. Gowdy^1^, Doriana Taccardi^1^, Amanda M. Zacharias^1^, Élisabeth Lamoureux^2^, Lesley Norris Singer^3^, Jennifer Daly-Cyr^3^, Manon Choinière^2^, Qingling Duan^1^, Zihang Lu^4^, M. Gabrielle Pagé^2^, Nader Ghasemlou^1, 5, 6^

^1^Department of Biomedical & Molecular Science, Queen’s University, Canada, ^2^Department of Anesthesiology & Pain Medicine, Université de Montréal, Canada, ^3^Chronic Pain Network, McMaster University, Canada, ^4^Department of Public Health Sciences, Queen’s University, Canada, ^5^Department of Anesthesiology & Perioperative Medicine, Queen’s University, Canada, ^6^Centre for Neuroscience Studies, Queen’s University, Canada

**Introduction**: Over 7 million Canadians live with chronic pain, for which current treatments are insufficient. In the pursuit of individualized management strategies, it is crucial to know why and when someone has pain. Time-dependent variability in thermal nociception was found to be regulated primarily by endogenous circadian rhythms; we hypothesize that circadian rhythms also play a role in mechanisms of chronic pain. Our study explores how variability across time – as a proxy for circadian rhythmicity – could be an important factor in understanding inter-individual differences in pain experiences, and if distinct temporal patterns exist.

**Methods**: Following an initial questionnaire, recruited participants (n = 907) completed electronic symptom-tracking diaries, in which they rated their pain intensity, affect, and fatigue on a 0–10 scale daily at 3 timepoints (08:00, 14:00, 20:00) for 1 week. Groups based on time-dependent changes in pain scores were identified using means and standard deviation.

**Results**: 585 participants completed 1 or more full days of compliant diaries (diary entry submitted within 2 hours of timepoint). Five preliminary pain rhythmicity groups were identified: constant low (23.0% of total), constant high (27.0%), increasing (rhythmic↑; 16.0%), decreasing (rhythmic↓; 4.1%), and arrhythmic (mixed; 29.9%). A latent class mixed effects model enabled the identification of alternative groups with a more data- driven approach.

**Discussion/Conclusions**: The interindividual differences in pain variability observed in our sample present a tool to characterize chronic pain, with potential ramifications for our clinical understanding of pain and circadian rhythmicity.

This work is supported by CIHR and the CIHR-SPOR Chronic Pain Network.

## Mapping the Prevalence and Severity of Menopause-related Symptoms in Pre-, Post- and Menopausal Women Attending a Chronic Pain Clinic

Shehnaz Fatima Lakha^1, 2^, Sonia Israilov^2^, Angela Mailis^1, 2^

^1^University of Toronto, ^2^Pain and Wellness Center

**Introduction**: Chronic pain is common among women, often worsened by life transitions like menopause, which brings hormonal changes that can intensify physical, mental, and psychological symptoms, especially in those already experiencing chronic pain. Despite the known effects of menopausal symptoms, comprehensive data lack regarding their prevalence among women seeking treatment at chronic pain clinics. This study aims to map the prevalence and severity of menopause-related symptoms in women seeking treatment for chronic pain.

**Methods**: A retrospective cross-sectional analysis was conducted involving female patients aged 50–60 years, attending a chronic pain clinic. Participants completed a consent form, a structured demographic questionnaire, and the Menopause Rating Scale (MRS) for presence and severity of common menopausal symptoms, as part of usual process of care. Data was analyzed using SPSS. The study protocol has been submitted to the U of T Research Ethics Board.

**Results**: Preliminary analysis shows that women age 53–58 (mean 55 yrs), experience mild to moderate menopause-related symptoms (MRS mean 19) across all domains: somatic (5.25); psychological (mean 6.5) and urogenital domain (mean 7.25), while they report moderate to severe pain. A detailed analysis of additional data will explore the prevalence/severity of menopause-related symptoms in female chronic pain patients.

**Discussion/Conclusions**: Prevalence and intensity of menopause-related symptoms will provide valuable insights for healthcare providers working with female chronic pain populations. Understanding the interplay between menopause-related symptoms and chronic pain can inform treatment strategies, enhance patient care, and contribute to the development of comprehensive management plans addressing both menopausal symptoms and chronic pain.

## The Contribution of Catastrophizing to the Transition from Acute to Chronic Pain-Related TMD: A Prospective Cohort Study

Sherif Elsaraj^1, 2^, Firoozeh Samim^2, 3^, Berlant AlSabbagh^1, 4^, Zovinar der Khatchadourian^2, 5^, Ana Miriam Velly^1, 2, 4^

^1^Department of Dentistry, Jewish General Hospital, Montreal, QC, Canada, ^2^Faculty of Dentistry, McGill University, Montreal, QC, Canada, ^3^Department of Dentistry, Montreal General Hospital, Montreal, QC, Canada, ^4^Lady Davis Institute for Medical Research, Montreal, QC, Canada, ^5^Alan Edwards Pain Management Unit, Montreal General Hospital, Montreal Quebec

**Introduction**: Pain-related Temporomandibular Disorders (PTMD) persist in more than 30% of cases, even after treatment. Catastrophizing has been recognized as a possible risk factor for chronic pain. To the best of our knowledge, this is the first study to explore whether catastrophizing contributes to the progression from acute to chronic PTMD at 3 months and its continuation at 6 months.

**Methods**: In this prospective cohort study, participants from Montreal and Ottawa were recruited, underwent clinical evaluations, and completed the validated Graded Chronic Pain Scale (GCPS) along with the Pain Catastrophizing Scale (PCS) at baseline. Only individuals with pain lasting under 3 months were included. Follow-up GCPS assessments at 3 and 6 months tracked the progression to chronic clinically significant pain (CSP), defined by a score above 50. Logistic regression was employed to identify associated risk factors.

**Results**: Of the 110 PTMD cases, 53.6% transitioned from acute to chronic PTMD by the 3-month follow-up, with 19.1% reporting clinically significant pain (CSP). By 6 months, 43% continued to report pain, with 18.2% experiencing CSP. Helplessness (OR = 1.13, 95% CI: 1.02–1.25) and rumination (OR = 1.18, 95% CI: 1.03–3.56) were associated with an increased likelihood of pain transition, while magnification (OR = 0.79, 95% CI: 0.68–0.92) was linked to a reduced risk of pain transition and persistence.

**Discussion/Conclusions**: The findings highlight catastrophizing as a key factor in the transition to chronic PTMD and its importance in treatment planning.

## Evaluating the Risk of CAP in Relation to Average Daily Dose of Opioids: Insights from a Nested Case-Control Study

Ana Velly^1, 2^, Russell Steele^1^, Eric Morenz^1^, Igor Karp^3^, Dwight E Moulin^3^, Daniel R Morales^4^, Jennifer S Landry^1^, Berlant AlSabbagh^2^, Florina Moldovan^5^

^1^McGill University, Montreal, Canada, ^2^Department of Dentistry, Jewish General Hospital, Montreal, Canada, ^3^University of Western Ontario, London, Canada, ^4^University of Dundee, Dundee, Scotland, ^5^Université de Montréal, Montreal, Canada

**Introduction/Aim**: To investigate the association between opioid use and the increased risk of community-acquired pneumonia (CAP) requiring hospitalization. Furthermore, we assessed the relationship between the average daily dose of opioids and CAP risk.

**Method**: This nested case-control study used the UK Clinical Practice Research Datalink linked to Hospital Episode Statistics. It included new opioid users aged ≥18 years, with up to two controls matched to CAP cases by sex, age, general practice, cohort entry date, and CAP diagnosis date. The primary exposure was the average daily dose, measured in morphine equivalence, prescribed within the 30-day exposure window. The average daily dose is calculated as the total milligrams of morphine equivalence (mgmeq) during the exposure time, divided by the length of the exposure window, which is 30 days. To account for potential confounders, multivariable conditional logistic regression analyses were performed to estimate the odds ratios (ORs) and 95% CIs for CAP. Oral7 prescriptions of opioids that had a missing prescribed daily dose were imputed.

**Results**: Among 932,267 opioid users, 18,445 CAP cases were identified (2.69 per 1,000 person-years) and matched with 30,566 controls. Opioid use significantly increased CAP risk compared to nonuse (OR: 1.83, 95% CI: 1.68–1.99). A median daily dose of 10 mgmeq over 30 days was associated with a twofold increase in CAP risk (OR: 2.05, 95% CI: 1.89–2.22). A 30 mgmeq increase in daily dose resulted in a 1.28-fold higher CAP risk.

**Discussion/Conclusions**: Opioid use increases CAP risk, with higher risk associated with increased dose.

## The Contribution of Health Conditions to the Transition From Acute to Chronic Pain-Related TMD: A Prospective Cohort Study

Ana Miriam Velly^1, 2, 3^, Sherif Elsaraj^1, 2^, Firoozeh Samim^2, 4^, Berlant AlSabbagh^1, 3^, Zovinar der Khatchadourian^2, 5^

^1^Department of Dentistry, Jewish General Hospital, Montreal, QC, Canada, ^2^Faculty of Dentistry, McGill University, Montreal, QC, Canada, ^3^Lady Davis Institute for Medical Research, Montreal, QC, Canada, ^4^Department of Dentistry, Montreal General Hospital, Montreal, QC, Canada, ^5^Alan Edwards Pain Management Unit, Montreal General Hospital, Montreal Quebec

**Introduction**: Pain-related Temporomandibular Disorders (PTMD) persist in over 30% of cases despite treatment. This study is the first to examine the impact of health conditions on the transition from acute to chronic PTMD at 3 months and its persistence at 6 months.

**Methods**: In this prospective cohort study, participants from Montreal and Ottawa underwent examinations and completed the Graded Chronic Pain Scale (GCPS) and health conditions questionnaires at baseline. Only individuals experiencing pain for less than 3 months were included. Follow-up GCPS assessments at 3 and 6 months were conducted to track the transition to chronic clinically significant pain (CSP, characteristic pain intensity > 50) and its persistence. Logistic regression analysis was used to identify associated risk factors.

**Results**: Among the 110 PTMD cases, 53.6% transitioned from acute to chronic PTMD by the 3-month follow-up, with 19.1% reporting chronic clinically significant pain (CSP). At 6 months, 43% continued to report pain, and 18.2% had CSP. Headache (OR = 2.55, 95% CI: 1.21–5.36) and heart disease (OR = 2.65, 95% CI: 1.08–6.52) increased the likelihood of transitioning to chronic PTMD, while asthma (OR = 0.44, 95% CI: 0.22–0.86) reduced the risk of both transition and persistence. Pain in the back, neck, chest, legs, arms, or abdomen, as well as high blood pressure, allergies, thyroid issues, and rheumatic diseases, were not associated with the transition or persistence of PTMD.

**Discussion/Conclusions**: The results suggest that headache and heart disease are associated with the transition to chronic clinically significant PTMD and should be considered when developing treatment plans for PTMD. The reduced risk associated with asthma may be due to the medications used for its treatment.

## Evidence, Systematic Reviews, Guidelines, Implementation ScienceOpioid Analgesics for Chronic Noncancer Pain in Patients Prescribed Opioid Agonist Therapy or with Opioid Use Disorder: A Systematic Review

Vahid Ashoorion^1^, Tushar Sood^2^, Shezel Muneer^2^, Jason Busse^3^, Danielle Rice^3^, Jaris Swidrovich^2^, Umair Majid^2^, James Abesteh^3^, Randi Mao^3^, Abhimanyu Sud^2, 4^

^1^Humber River Health, Toronto, ON, ^2^University of Toronto, Toronto, Ontario, Canada, ^3^McMaster University, Hamilton, Ontario, Canada, ^4^Humber River Health, Toronto, Ontario, Canada

**Introduction**: This study aimed to conduct a systematic review to explore the efficacy, effectiveness, and safety of opioid analgesics alone or in combination with opioid agonist therapy (OAT) to manage chronic non-cancer pain (CNCP) in people with OUD or with a history of OUD.

**Methods**: We searched MEDLINE, Embase, PsycINFO, CINAHL and AMED from inception to July 2023 for randomized studies and up to January 2025 for nonrandomized studies. We assessed the risk of bias in included studies, evaluated the quality of evidence using the GRADE approach, and provided a narrative summary of treatment effects.

**Results**: Our search identified 15,988 unique citations, of which six observational studies were deemed eligible to inform safety outcomes for review, while no observational studies or RCTs met the eligibility criteria for efficacy or effectiveness outcomes. The likelihood of suicidality was twice as high in CNCP patients with OUD receiving long-term opioid therapy (LTOT) compared to those without OUD (absolute risk increase: 127; 95% CI: 36 to 249 more participants with suicidality in 1,000 participants; moderate certainty evidence). Compared to opioid analgesics alone, the risk of fatal opioid-related overdose may decrease in patients with CNCP and OUD who receive both opioid analgesics and OAT (absolute risk reduction: 60; 95%CI: 18 to 94 fewer deaths in 1,000 participants; low certainty evidence).

**Discussion/Conclusions**: There is limited evidence to guide opioid prescribing for individuals with OUD. Further studies are needed to better understand the benefits and risks of opioid analgesics for chronic non-cancer pain (CNCP) patients with OUD.

## Caring for Clinicians Managing Chronic Pain: A Systematic Review of Validated Questionnaires for the Measurement of Clinicians’ Well-Being

Claudie Audet^1^, Andréanne Bernier^1^, Marimée Godbout-Parent^1^, Hermine Lore Nguena Nguefack^1^, Lise Ferland^1^, Paula L. Bush^2^, Tracy A. Barnett^2^, Sylvie Lambert^2^, Anaïs Lacasse^1^

^1^Université du Québec en Abitibi-Témiscamingue, ^2^Université McGill

**Introduction**: The management of chronic pain (CP) can be complex and poses multiple challenges for clinicians. Measuring CP clinicians’ experience of care, and more broadly their well-being, provides valuable insights into the impact of the practice environment, with the potential to improve workforce retention, patient safety, and care quality.

**Objectives**: This systematic review aimed to appraise validated self-reported measurement instruments designed to measure the well-being of clinicians.

**Methods**: Validation studies published in English or French on measurement instruments covering domains of clinicians’ well-being, as proposed by the National Academy of Sciences, were included. Studies were retrieved in December 2023, by searching the following databases with a 10-year filter: CINAHL, Embase, HaPI, MEDLINE, PsycINFO, Mental Measurements Yearbook, and APA PsycTests. The study selection process was completed by two independent reviewers.

**Results**: Out of 10,441 records identified 136 validation studies were included. Most came from the USA (27.2%), Spain (11.0%), Canada (5.9%), and Australia (5.9%). Among profession-specific instruments (44.1%), the most targeted professionals were nurses and physicians. Issues such as fatigue and compassion fatigue, trauma, burnout, and stress were covered by 17.6% of the instruments. Evidence-based practice, multidisciplinary practice and collaboration were covered by 22.1% of the instruments. Most covered measurement properties were internal consistency (89.7% of studies) and structural validity (72.8%).

**Discussion/Conclusions**: Many instruments for measuring clinicians’ well-being are discussed in the scientific literature, but few have been fully tested for all measurement properties. However, our study provides a solid foundation for teams to advance psychometric evaluation and cross-cultural adaptation.

## Chronic Pain Primary Care Clinics: Different Contexts but Similar Facilitators and Barriers

Tania Augière^1, 2^, Gabriella Lavoie-Dias^1, 2^, Yves Couturier^3, 4^, Manon Choinière^1, 2^, Gabrielle Pagé^1, 2^

^1^Centre de recherche du Center hospitalier de l’Université de Montréal, ^2^Université de Montréal, ^3^Centre recherche sur le vieillissement, ^4^Université de Sherbrooke

**Introduction**: In 2021, a Chronic Pain Action Plan was put in place by the Quebec Ministry of Health and Social Services. Following this plan, five interprofessional teams were established to promote a primary care approach for chronic pain management. This study examined the heterogeneity of implementation strategies between the teams and identify facilitators and barriers from the perspective of the team managers.

**Methods**: All participating clinics’ managers (n = 5) completed a self-report questionnaire on implementation practices using REDCap within the first 6 months of setting up these new services.

**Results**: Even though the five clinics had some common characteristics (e.g., a goal of providing an interprofessional assessment of patients, the intention of prioritizing the patients of primary care facilities), results show that they differed significantly in terms of the clinic’s location, target population, and services offered. Four out of 5 managers indicated that an important facilitator was the availability of adequate training in chronic pain management for their professionals. Three mentioned the strong motivation of the hired healthcare professionals. The hardest aspects (reported by 3/5 managers) in setting up these new services were handling a project with lack of long-term funding, finding the necessary material resources (e.g., office space), managing the additional responsibilities of this new service, and collecting data to measure the impact of the new service.

**Discussion/Conclusions**: Despite supervising different teams, the experience of the chronic pain team managers suggests similar facilitators and barriers which will be important to consider when scaling up these initiatives throughout the province.

## Interprofessional Primary Care Clinics for Chronic Pain Management: Assessment of Perceived Competence, Consideration of Psychosocial Factors, and Use of External Resources

Tania Augière^1, 2^, Gabriella Lavoie-Dias^1, 2^, Yves Couturier^3, 4^, Manon Choinière^1, 2^, Gabrielle Pagé^1, 2^

^1^Centre de recherche du Center hospitalier de l’Université de Montréal, ^2^Université de Montréal, ^3^Centre recherche sur le vieillissement, ^4^Université de Sherbrooke

**Introduction**: In response to the Chronic Pain Action Plan developed by the Quebec Ministry of Health and Social Services, five interprofessional primary care clinics were recently set up to offer optimal chronic pain management and reduce the pressure on specialized pain services. This study aimed to examine factors that could impact the implementation of this new service, including the professionals’ perceived competence in working with chronic pain populations, their perspectives on team work approaches including the consideration and incorporation of psychosocial factors in pain management, and their use of external resources to guide their practice.

**Methods**: Healthcare providers (n = 27) working in these interprofessional clinics completed a self-report questionnaire using REDCap within the first 6 months of setting up these new services.

**Results**: Of the 27 respondents, 23 were clinicians, with a high proportion of rehabilitation professionals (61%). Psychosocial factors were considered as highly important (mean ± SD = 8.04/10 ± 0.27). Healthcare providers reported feeling competent in their new role (mean ± SD = 7.19/10 ± 0.23) when treating patients with chronic pain. Eighty percent of the professionals indicated they use validated pain assessment tools routinely. The main reasons for not using these tools were a lack of training (67%) and a perceived lack of expertise (43%).

**Discussion/Conclusions**: Psychosocial factors are perceived as important in the development of pain management plans by professionals working in interprofessional primary care settings. However, a perceived lack of training may hinder the use of external resources, such as validated tools.

## A Novel Systems-Based High-Impact Implementation Strategy to Improve Procedural Pain for Infants in Canadian NICU: Clinical and Implementation Effectiveness

Mariana Bueno^1^, Bonnie Stevens^2^, Melanie Barwick^2^, Marsha Campbell-Yeo^3^, Christine Chambers^3^, Carole Estabrooks^4^, Rachel Flynn^5^, Sharyn Gibbins^6^, Denise Harrison^7^, Wanrudee Isaranuwatchai^8^, Sylvie LeMay^9^, Melanie Noel^10^, Jennifer Stinson^2^, Anne Synnes^11^, Charles Victor^1^, Janet Yamada^12^

^1^University of Toronto, ^2^The Hospital for Sick Children, ^3^Dalhousie University, ^4^University of Alberta, ^5^University College Cork, ^6^Trillium Health Partners, ^7^University of Melbourne, ^8^St. Michael’s Hospital, ^9^Université de Montréal, ^10^University of Calgary, ^11^BC Children’s Hospital Research Institute, ^12^Toronto Metropolitan University

**Introduction**: The Implementation of Infant Pain Practice Change (ImPaC) Resource is a web-based high-impact implementation strategy developed to improve NICU pain practices. We aimed to evaluate clinical and implementation effectiveness of ImPaC in Canadian NICUs.

**Methods**: A hybrid type 1 effectiveness-implementation design (cluster randomized controlled trial and longitudinal descriptive study) was utilized. Eligible NICUs with >15 beds were randomized to intervention (INT) or wait-listed to usual care (UC) for 6 months. NICUs in the UC group then were offered ImPaC in an equivalent manner to INT group. The number of painful procedures/infant/day and the proportion of infants with assessment and management associated with procedures were the primary outcomes. Secondary outcomes were implementation feasibility and fidelity.

**Results**: Data from ~30 infants/site (INT = 678, UC = 325) were collected from 23 NICUs (INT = 12, UC = 11). The average number of procedures/infant/day was less in the INT group (p = .003). The proportion of procedures associated with pain assessment (p = .001) and pain management (p = .001) was greater in the INT group. NICUs spent an average of 10.18 (±4.36) hours implementing ImPaC and 14/23 (60.9%) NICUs implemented ImPaC with fidelity (as intended). NICUs who spent more time using ImPaC and with fidelity achieved fewer painful procedures/infant/day and improved use of pain assessment and treatment strategies.

**Discussion/Conclusions**: Clinical and implementation effectiveness of the ImPaC Resource resulted in improved pain practices. Further exploration of implementation strategies needs to be undertaken to effectively introduce ImPaC in NICUs beyond the Canadian context.

## Skin-to-skin Interventions for Painful Procedures in Very and Extremely Preterm Infants: A Systematic Review and Meta-Analysis

Estreya Cohen^1^, Haleh Hashemi^1^, Nichaela Garvey^1^, Carol Cheng^2^, Vibhuti Shah^2^, Rebecca Pillai Riddell^3^

^1^York University – Faculty of Graduate Studies, ^2^Mount Sinai Hospital, ^3^York University – Faculty of Health

**Introduction**: Very and extremely preterm infants are subjected to increased number of painful procedures. They are less physiologically stable than older preterm infants, parents are less able to do skin-to-skin contact (SSC) due to infant physiological instability, and there is a higher prevalence of infant support devices that makes SSC for pain more challenging. Thus, the effectiveness of SSC for painful procedures in this population must be systematically examined. The aim of the present study is to synthesize studies that examine the effectiveness of SSC for procedural pain in very and extremely preterm infants.

**Methods**: We searched MEDLINE, Embase, CINAHL, Cochrane, and APA PsycInfo for studies examining the effectiveness of SSC during painful procedures for very and extremely preterm infants (GA < 32 weeks, 6 days). Reviewers independently screened titles and abstracts and reviewed the full texts.

**Results**: Of the 1657 identified, 24 studies fulfilled the inclusion criteria. All studies examined procedural pain, with heel stick being the most common painful procedure (n = 20), followed by retinopathy of prematurity screening (n = 2), tape removal (n = 1) and venepuncture (n = 1). Skin-to-skin contact was usually evaluated with the infant’s mother (n = 20), and in 2 studies an alternative female performed SSC. Quality of the studies was mixed with the minority being of high quality. Effect sizes varied dependent on outcome measures.

**Discussion/Conclusions**: This review provides important and relevant insights into premature infant pain management. There is very little research examining this population and these preliminary findings highlight how SSC is implemented in preterm infant pain research.

## Consolidation and Systematic Appraisal of Guideline Recommendations Regarding Management of Chronic Pain: A Protocol for a Digital Chronic Pain Recommendation Map

Andrea Darzi^1^, Kian Torabiardakani^1^, Gonzalo Bravo-Soto^1^, Daniela Montalva-Romero^1^, Rachel Couban^1^, Lynn Cooper^1^, Stacey Ritz^1^, Jaris Swidrovich^2^, Vivian Welch^3^, Gordon Guyatt^1^, Holger Schünemann^1, 4^, Jason Busse^1^

^1^McMaster University, ^2^University of Toronto, ^3^University of Ottawa, ^4^Humanitas University

**Introduction**: Chronic pain affects 1 in 5 people; however, management remains suboptimal. There is an urgent need to improve concordance between evidence and practice. Thus, our aim is to develop and mobilize a digital chronic pain recommendation map (RecMap) in three priority areas: opioids, cannabis for medical purposes, and spine- related interventional procedures.

**Methods**: The project comprises three main phases including the: (1) planning phase: where we engaged with diverse interest-holders and co-designed a team structure to ensure an efficient and effective workflow; (2) development phase: First, we systematically and comprehensively identified relevant guidelines in any language since 2019 through a search of electronic databases and international websites. Teams of reviewers were trained to screen titles and abstracts and full texts to identify eligible guidelines. Once this step is complete, we will appraise the reporting quality of the guidelines and recommendations using the AGREE-II tool and AGREE-REX respectively. Third, we will extract data including relevant equity information using infrastructure in GRADEPro. Fourth, we will explore divergence and compare recommendations answering the same guideline question using a specific set of criteria. Fifth, we will develop plain language summaries/recommendations (PLSs and PLRs) in narrative and infographic formats and decision aids to support patient-physician decision making. Finally, we will translate the platform to French, Spanish, and Mandarin; (3) Mobilization phase: we will co-create strategies with our interest holders to disseminate the RecMap to relevant target users including people inequitably impacted by chronic pain.

**Discussion/Conclusions**: The Chronic Pain RecMap will enhance (1) transparency, recognition, and promotion of trustworthy evidence for people living with chronic pain, and (2) accountability among decision makers. Also, features of the RecMap such as decision aids will support shared decision making and encourage consideration of patient’s values and preferences where needed. Work is currently underway and we anticipate having findings by May 2025.

## Prevalence of Symptom Exaggeration Among North American Independent Medical Evaluation Examinees: A Systematic Review of Observational Studies

Andrea Darzi^1^, Li Wang^1^, John Riva^1^, Rami Morsi^2^, Rana Charide^1^, Rachel Couban^1^, Samer Karam^1^, Kian Torabiardakani^1^, Annie Lok^1^, Shanil Ibrahim^1^, Sheena Bance^3^, Regina Kunz^4^, Gordon Guyatt^1^, Jason Busse^1^

^1^Mcmaster University, ^2^University of Chicago, ^3^Centre for Addiction and Mental Health, ^4^University Hospital Basel

**Introduction**: Independent medical evaluations (IMEs) are commonly acquired to provide an assessment of impairment; however, these assessments show poor interrater reliability. One potential contributor is symptom exaggeration by patients, who may feel pressure to emphasize their level of impairment to qualify for incentives. This study explored the prevalence of symptom exaggeration among IME examinees in North America, which if common may represent an important consideration for improving the reliability of IMEs.

**Methods**: We searched CINAHL, EMBASE, MEDLINE and PsycINFO to July 08, 2024 for observational studies that used a known-group design or multimodal determination method. Paired reviewers independently assessed risk of bias and extracted data. We performed a random-effects model meta-analysis to estimate the overall prevalence of symptom exaggeration and explored potential subgroup effects for sex, age, education, clinical condition, and confidence in the reference standard. We used the GRADE approach to assess the certainty of evidence.

**Results**: We included 44 studies with 46 cohorts and 9,794 patients. The median of the mean age was 40 (IQR 38 to 42). Most cohorts included patients with traumatic brain injuries (67%) or chronic pain (24%). Prevalence of symptom exaggeration across studies ranged from 17% to 67%. We found low certainty evidence suggesting that studies with a greater proportion of women (≥40%) may be associated with higher rates of exaggeration (47%, 95%CI 36 to 58) vs. studies with a lower proportion of women (<40%) (31%, 95%CI 28 to 35; test of interaction p = .02). We found no significant subgroup effects for type of clinical condition, confidence in the reference standard, age, or education.

**Discussion/Conclusions**: Symptom exaggeration may occur in almost 50% of women and in around a third of men undergoing IMEs. Future research should establish the reliability and validity of evaluation criteria for symptom exaggeration and develop a structured IME assessment approach.

## Peer Support in Chronic Pain Management: A Patient Co-Led Systematic Review

Jennifer Donnan^1^, Joshua Rash^1^, Drakes Dalainey^2^, Swab Michelle^1^, Queen Jacques^1^, Alesha King^1^, Linkiwich Delane^3^, Whelan Kati^1^, Mcintyre Virginia^4^

^1^Memorial University, ^2^University of Ottawa, ^3^University of Guelph, ^4^People in Pain Network

**Introduction**: Chronic pain affects 20% of Canadians, and these patients often struggle to access care leaving them feeling isolated. Care typically involves a multi-modal biopsychosocial treatment approach. Peer support is one option that is broadly described as individuals with lived experience offering empathy, validation, and support to others facing similar challenges. A systematic review was conducted to understand the effectiveness and role of peer support for adults with chronic pain.

**Methods**: Ten databases were searched between inception and May 29, 2024. Studies were eligible if they were a randomized-controlled trial or observational study that examined peer support among community-dwelling adults with chronic non-cancer pain and evaluated: physical and emotional functioning, pain, quality of life, self-efficacy, self-management knowledge, and/or healthcare utilization.

**Results**: In all, 21 studies met inclusion criteria. There was heterogeneity across definitions of peer support and outcome measures. Pain interference improved in most studies, with peer-led interventions often yielding better outcomes than usual care. However, neither within group significance and clinical meaningfulness was achieved. Eight of 15 studies showed within-group improvements in symptoms of depression, but few found significant differences between peer programs and heterogeneous controls. Peer-support only resulted in a significant improvement in self-efficacy relative to control in one trial.

**Discussion/Conclusions**: The effect of peer-led interventions were difficult to discern given heterogeneity among trials conducted. Evidence suggests improvement in pain- related interference and emotional well-being are likely outcomes. Further research is needed to clearly define and standardize peer support interventions and assess their long-term effectiveness across diverse populations.

## The Impact of Chiropractic Care on Prescription Opioid Use for Non-Cancer Spine Pain: A Systematic Review and Meta-Analysis

Peter Emary^1^, Kelsey Corcoran^2^, Brian Coleman^2^, Amy Brown^3^, Carla Ciraco^3^, Jenna DiDonato^3^, Li Wamg^1^, Rachel Couban^1^, Abhimanyu Sud^4^, Jason Busse^1^

^1^McMaster University, ^2^Yale University, ^3^Private Practice, ^4^University of Toronto

**Introduction**: Chiropractic care for spine-related pain may reduce opioid prescribing and long-term opioid use; however, the overall magnitude and certainty of these effects are uncertain. We conducted a systematic review and meta-analysis to assess the impact of chiropractic care on new or continued prescription opioid use among adults with non-cancer spine pain.

**Methods**: We searched for eligible randomized controlled trials (RCTs) and observational studies indexed in MEDLINE, Embase, AMED, CINAHL, Web of Science, and the Index to Chiropractic Literature up to June 27, 2024. Paired reviewers independently assessed risk-of-bias and extracted data. We performed random- and fixed-effects meta-analyses and used GRADE to assess the certainty of evidence.

**Results**: Two RCTs (838 participants) and 18 cohort studies (5,292,620 participants) were eligible for review. We found very low-certainty evidence that, compared to standard medical care alone, receipt of chiropractic care was associated with between 34% and 65% lower odds of receiving prescription opioids (OR = 0.66; 95% CI, 0.50–0.86; 2 RCTs; and OR = 0.35; 95% CI, 0.27–0.45; 18 cohort studies) and 73% lower odds of initiating long-term opioid use (OR = 0.27; 95% CI, 0.15–0.47; 6 cohort studies) for non-cancer spine pain. Absolute risk reductions among chiropractic recipients ranged from 10% less to 16% less initiating prescription opioids for spine-related pain, and 3% less initiating long-term opioid use.

**Discussion/Conclusions**: Our findings suggest that access to chiropractic services may reduce reliance on opioids for non-cancer spine pain; however, the evidence is very low-certainty. Rigorously designed RCTs are needed to confirm these findings.

## Measurement Properties of the Numerical Pain Rating Scale in People with Shoulder Disorders

Rochelle Furtado^1^, Romi Haas^2^, Hana Marmura^3^, Samuel Whittle^4^, Sofia Ramiro^5^, Dorcus Beaton^6^, Beverley Shea^7^, Pamela Richards^8^, Arianne Verhagen^9^, Joel Gagnier^10^, Rachelle Buchbinder^2^

^1^Health and Rehabilitation Sciences, Western University, ^2^School of Public Health and Preventive Medicine, Monash University, ^3^University of North Carolina, North Carolina, United States, ^4^The Queen Elizabeth Hospital, Adelaide, Australia, ^5^Leiden University Medical Center, Leiden, The Netherlands, ^6^Institute of Health & Work and the University of Toronto, Toronto, Ontario, Canada, ^7^Ottawa Hospital Research Institute, University of Ottawa, ^8^Bristol, United Kingdom, ^9^Graduate School of Health, University of Technology Sydney, ^10^Departments of Epidemiology & Biostatistics and Surgery, Western University, Ontario, Canada

**Introduction**: We aimed to synthesize the available evidence on the measurement properties of the Numeric Pain Rating Scale (NPRS), to determine its suitability to measure shoulder pain within a core outcome set.

**Methods**: MEDLINE, EMBASE and CINAHL were searched until June 2023. Studies assessing any psychometric property of the NPRS, that was used in a population of patients with shoulder disorders was included. Two reviewers independently screened articles, assessed risk of bias using the Good Methods checklist. Data extraction was completed by the reviewers. The available evidence across all measurement properties was synthesized using the OMERACT Filter 2.2 Instrument Selection Algorithm. For each measurement property, evidence quality was rated as green (good evidence supporting this property), amber (some caution but good enough to move forward) or red (evidence against this property or only poor quality evidence).

**Results**: A total of 12 studies, that examined 5 different measurement properties of the NPRS were retrieved. Three studies examined construct validity, 2 studies for test-retest reliability, 4 studies for responsiveness, 4 studies for clinical trial discrimination and 3 studies for thresholds of meaning. Based on the algorithm, 4/5 measurement properties were rated amber. Only the property of thresholds of meaning was rated green, indicating good methods and adequate performance.

**Discussion/Conclusions**: Despite the popularity of the NPRS, additional high-quality research is required to confirm construct validity, test-retest reliability, responsiveness and clinical trial discrimination of the NPRS before it can be endorsed as part of a core outcome set.

## Stress triggers and Chronic Low Back Pain: How Flexibility Shapes Pain Experiences

Mael Gagnon Mailhot^1, 2^, Karen Ghoussoub^1, 2^, Lise Dassieu^3, 4^, Élise Develay^1^, Mathieu Roy^5^, Étienne Vachon-Presseau^6^, Pierre Rainville^7^, Sonia Lupien^8^, M. Gabrielle Pagé^1, 2, 9^

^1^Research Center of the Center hospitalier de l’Université de Montréal, Montréal, Canada, ^2^Department of psychology, University of Montreal, Montreal, Canada, ^3^Research Center of the CIUSSS-du-Nord-de-l’Île-de-Montréal, Montreal, Canada, ^4^School of Social Work, Université du Québec à Montréal (UQAM), Montreal, Canada, ^5^Department of psychology, McGill University, Montreal, Canada, ^6^Faculty of Dental Medicine, McGill University, Montreal, Canada, ^7^Research Center of the Institut Universitaire de Gériatrie de Montréal, Montreal, Canada, ^8^Department of Psychiatry, University of Montreal, Montreal, Canada, ^9^Department of Anesthesiology and Pain Medicine, University of Montréal, Montreal, Canada

**Introduction**: The STUN model identifies four key triggers of the physiological stress response: lacking a perceived Sense of control, social-evaluative Threat, Unpredictability and Novelty. This study explores how individuals with chronic low back pain (CLBP) experience daily subjective stress and its relationship with pain.

**Methods**: This qualitative study is part of a larger mixed-methods longitudinal project investigating stress-pain associations among individuals with CLBP. Semistructured interviews were conducted with participants who were selected based on their responses to an electronic diary measuring daily stress and pain. Transcripts were analyzed inductively with a reflexive thematic approach to identify key themes related to the interactions between STUN characteristics and pain perception.

**Results**: We recruited 29 participants (15 men, 14 women) with a mean age of 49 years (range: 23–71 years). The study highlights the significant role of social-evaluative threats and compounding stress triggers in shaping chronic pain experiences. This effect is particularly pronounced when multiple STUN factors interact, undermining participants’ coping strategies. However, cognitive and behavioral flexibility, such as adapting activities, being mindful of limitations, and reframing pain perception, can restore agency, reduce subjective stress, and improve pain self-management. Yet, the lack of flexible approaches fosters learned helplessness through avoidance, self-blame, and a perceived loss of agency.

**Discussion/Conclusions**: These findings highlight how triggers of physiological stress influence lived pain experiences. Results underscore the importance of considering subjective psychosocial stressors in chronic pain experiences. Future research could explore how promoting flexibility and adaptive coping strategies may enhance stress resilience and improve pain outcomes.

## A Scoping Review of the Literature to Understand the Perceptions, Attitudes, and Preferences Toward Exercise/Physical Activity Interventions for Individuals Living with Pain

Nicholas Held^1^, Joline Attalla^2^, Robin Campbell Bromhead^2^, Joy C. MacDermid^2^, Jordan Miller^3^, Temitope Osifeso^2^, Allison Mizzi^1^, Lauren Roberts^3^

^1^McMaster University, ^2^Western University, ^3^Queens University

**Introduction**: Living with pain can make physical activity/exercise (PA/E) challenging. Extensive research has been completed to understand the barriers and facilitators of PA/E for various populations; however, less is known about this and perceptions, attitudes, and preferences toward PA/E among individuals living with pain. The aim of this scoping review was to explore the literature on the attitudes, perspectives, and preferences toward PA/E for individuals with pain.

**Methods**: Databases MEDLINE, EMBASE, SPORTDiscus, Web of Science, Emcare, and Psychinfo were searched from inception to June 19, 2023. The study followed the Preferred Reporting Items for Systematic Reviews and Meta-Analyses extension for Scoping Reviews (PRISMA-ScR) Checklist 9 and methodological standards for scoping reviews.

**Results**: 14,374 articles and abstracts were screened for eligibility, 254 underwent full screening, and 73 met inclusion criteria. A preliminary synthesis of data extracted from 59 full-text articles was conducted. Five themes emerged: Physical (pain severity, pain condition, comorbidities, physical benefits); Psychological (motivations, knowledge of PA/E, fear of pain/injury, psychological benefits), Social (camaraderie, group settings, social and professional support), Environmental (weather, facilities, location, time, transportation, cost), and Cultural (cultural attitudes and beliefs, gender, and societal expectations).

**Discussion/Conclusions**: These results provide a comprehensive understanding of the complexity of contributing factors toward PA/E engagement for individuals living with pain. This information will help inform the specifications for a decision aid to support CAF Veterans living with pain to engage in exercise.

## Patient-Reported Outcomes in Randomized Clinical Trials: A Repeated Cross-Sectional Study

Ala Heravi^1^, Dmitry Gryaznov^1^, Jason Busse^2^, Christof Schönenberger^1^, Belinda von Niederhäusern^3^, Lena Hausheer^1^, Manuela Covino^1^, Johannes Schwenke^1^, Selina Epp^1^, Alexandra Griessbach^1^, Malena Chiaborelli^1^, Arnav Agarwal^2^, Szimonetta Lohner^4^, Julian Hirt^1^, Stefan Schandelmaier^1^, Simon Egli^1^, Moshao Makhele^1^, Alain Amstutz^1^, Dominik Mertz^2^, Anette Blümle^5^, Erik von Elm^6^, Ramon Saccilotto^1^, Ayodele Odutayo^7^, Sally Hopewell^7^, Benjamin Speich^1^, Matthias Briel^1^

^1^University of Basel, ^2^McMaster University, ^3^Roche Pharma AG, ^4^University of Pécs, ^5^University of Freiburg, ^6^University of Lausanne, ^7^University of Oxford

**Introduction**: We examined the prevalence and characteristics of patient-reported outcomes (PROs) in randomized clinical trial (RCT) protocols across medical fields, their reporting quality, and the consistency between PROs specified in trial protocols and subsequent reporting in trial publications.

**Methods**: We included 237 RCT protocols approved in 2012, and 251 approved in 2016, by ethics committees in Switzerland, Germany, and Canada. Pairs of reviewers independently extracted characteristics of RCT protocols, PROs specified in protocols and reported in corresponding results publications and assessed the reporting quality of RCTs with a PRO as the primary outcome.

**Results**: Out of 488 included RCT protocols, 147 (30%) did not report use of a PRO; 97 (20%) specified a PRO as the primary outcome and an additional 244 (50%) as a secondary outcome. The prevalence of PROs varied substantially across medical fields, ranging from 100% in rheumatology and psychiatry to about one third in cardiology and anesthesiology. At 8–10 years after RCT approval, 40% of published trials (115/264) reported all PROs as defined in the protocol, 21% (55/264) did not report any pre- specified PROs, and 36% (94/264) reported more, less, or different PROs than pre- specified in the protocol. Among 63 RCT publications that reported a PRO as their primary outcome, reporting quality was often inadequate with 7 of 13 CONSORT-PRO items met by less than half of trials.

**Discussion/Conclusions**: Less than half of RCT protocols with planned PROs reported them as specified in corresponding published results, suggesting outcome reporting bias.

## Transforming Chronic Pain Care: Co-Designing the Alberta Virtual Pain Program with People with Lived Experience

Elena Lopatina^1, 2^, Susan Sobey-Fawcett^2^, Tina Hoang^1, 2^, Magali Robert^1, 2, 3^

^1^University of Calgary, ^2^Primary Care Alberta, ^3^Alberta Health Services

**Introduction**: The Alberta Virtual Pain Program (AVPP) was developed to address gaps in chronic pain care in Alberta.

**Methods**: Engagement and co-design with people with lived experience (PWLE) has been a cornerstone of the AVPP’s development and implementation. Key examples include:

- The proposed model of care for the AVPP was directly informed by patient and community engagement research, which produced key recommendations for the health delivery system to improve care and support for individuals living with chronic pain (https://doi.org/10.1136/bmjopen-2023-072048),which served as the foundation for the AVPP’s design.
- PWLE provided letters of support, which accompanied the AVPP proposal submitted to the government, and participated in meetings with government leaders to advocate for enhanced chronic pain resources in Alberta.
- PWLE actively contributed to the co-design of the AVPP as members of the advisory committee and working groups, determining the structure and content of services, reviewing materials, and shaping program delivery.
- A feedback process was established to capture and act on participant experiences through anonymous surveys and open dialogue during Peer Support Worker-led group sessions. This feedback is reviewed by the team to enable timely improvements.

**Results**: The AVPP officially launched in April 2024, and within less than 6 months, over 200 patients have already engaged with its services. Participant feedback has been overwhelmingly positive.

**Discussion/Conclusions**: The success of the AVPP underscores the critical role of PWLE in every phase of health system innovation.

## An Ecosystem of Accepting Life with Chronic Pain: A Meta-ethnography

Cassandra Macgregor^1^, David N Blane^2^, Emmanuelle Tulle^1^, Claire L Campbell^3^, Ruth J Barber^4^, Clementine Hill O-Connor^2^, Christopher Seenan^1^

^1^Glasgow Caledonian University, ^2^University of Glasgow, ^3^NHS Fife, ^4^No Affiliation

**Introduction**: Chronic pain is a heterogeneous category of conditions and experiences. For people living with long-term pain, “acceptance” can be associated with improved quality of life, mood and function, but is typically framed as an individual behavior. Qualitative studies of lived experiences challenge this framing, demonstrating complexity related to sociocultural and healthcare experiences, and difficulties with language, meaning, and measurement.

**Methods**: We conducted a systematic database, and gray literature, search, including qualitative studies using adults with chronic pain as the primary condition. We conducted meta-ethnographic synthesis with researchers of differing backgrounds.

**Results**: we included 10 studies. Our “lines of argument” include:

- Accepting life with chronic pain involves a fluid and continuous journey with fluctuating states of acceptance, a turning point, and iterative steps.
- “Acceptance” is a contested concept, related to the underpinning nature of chronic pain, and cultural models of health and illness.
- The individual’s journey can include a challenge to identity, negatively influenced by capitalist and ableist ideology and structures, with the associated individualism limiting the accepting process for some with chronic pain, particularly those ascribed lower socioeconomic status.

We found it helpful for individuals to experience a caring, supportive and coherent system that includes healthcare, workplaces and political discourse being adaptive to the realities of chronic pain. We combined these into a conceptual framework; “an ecosystem.”

**Discussion/Conclusions**: Our findings broaden conceptualization of “acceptance of chronic pain” beyond an individualized psychological construct, to a fluid and continuous journey, interconnected with our sociocultural-political worlds; an ecosystem.

## Promoting Evidence-Informed and Equitable Primary Care for People Living with Chronic Pain and Illness: A Scoping Review

Megan MacNeil^1, 2, 3^, Jenna Jessa^2^, Lesley Singer^1^, Alex Haaggaard^1^, Justin Bonhomme^2^, Kate Storey^3^, Kathryn A. Birnie^1, 2^

^1^Chronic Pain Network, ^2^University of Calgary, ^3^University of Alberta

**Introduction**: Primary healthcare is essential for ensuring equitable access to care and managing chronic conditions like chronic pain in Canada. Primary care providers face diverse and competing demands, which can create barriers to evidence-informed clinical decision making. Knowledge mobilization (KM) strategies can help bridge this gap by facilitating access to evidence-informed research. To inform the Chronic Pain Network’s activities, this scoping review consolidates evidence on KM strategies that engage primary care in implementing evidence-based interventions to support people with chronic illness.

**Methods**: Medline, Scopus, Embase, CINAHL, and Web of Science were searched from January 2004 to May 27, 2024. Three reviewers screened abstracts, and two reviewers screened full-text articles. Eligible studies: 1) focused on evidence-based interventions for chronic illness care, 2) reported on KM strategies to promote evidence use in care, and 3) took place in primary/community care settings in Canada. KM strategies were coded using the ERIC taxonomy and AIMD framework. Descriptive summaries and narrative synthesis were used to analyze the data.

**Results**: The search strategy generated 2,679 unique references. Of these, 161 met inclusion criteria and were reviewed in greater detail for analysis. Studies included a wide range of primary care providers (e.g., family physicians, allied health professionals) and varied KM strategies across the ERIC taxonomy (e.g., supporting providers, developing partner interrelationships, providing interactive assistance).

**Discussion/Conclusions**: This review offers insight into how KM strategies are applied in primary care and the components of their delivery. Findings will inform effective KM for chronic pain in Canada.

## Nociplastic Pain Criteria Must Expand to Include Patients with Unclassifiable Pain

Angela Mailis^1^, Demetry Assimakopoulos^1^, Jaqueline Clark^2^

^1^Pain and Wellness Center, ^2^Pain In Motion Research Group

**Introduction**: The IASP term “Nociplastic pain” is a third mechanistic descriptor for chronic pain patients with pain perception primarily mediated by CNS mechanisms. Specific grading nociplastic pain criteria require clinical pain hypersensitivity signss, leaving many individuals with non-nociceptive, non-neuropathic pain as “unclassifiable.” Here we propose criteria modification to include additional sensory alterations to avoid the problem of “unclassifiable pain.”

**Methods**: Comprehensive literature review gathered evidence for the prevalence and types of non-nociceptive/non-neuropathic chronic pain patients with sensory deficits or no sensory alterations, as well neurophysiological evidence as to the hyposensitivity substrate in these populations.

**Results**: Clinical and QST studies show that 19.8–34% of fibromyalgia patients (prototype of nociplastic pain syndromes) fail to qualify as nociplastic pain based on current nociplastic criteria, while 20%-40% of non-nociceptive/non-neuropathic pain patients display sensory deficits. QST confirms sensory deficits in nonspecific neck pain, chronic low back pain, cold CRPS etc. Brain imaging studies show that widespread hypoesthesiae are associated with brain function, metabolism, and structural changes. Mechanisms associated with sensory hyposensitivity reportedly include premorbid sensory processing abnormalities, attentional processes causing diversion away from painful areas, and psychological factors, resulting in CNS-mediated inhibition of somatic sensations.

**Discussion/Conclusions**: We propose that the IASP Taxonomy Task force consider updating the algorithm and allowing 3 nociplastic pain subgroups (with sensory loss/deficit; mixed deficits/gains; no sensory abnormality; criterion 3). These subtypes would be classified as possible nociplastic pain in the absence co-morbidities and probable in the presence of co-morbidities (criterion 4), while the label of “unclassifiable pain” is removed.

## Harms of Interventional Procedures for Chronic Noncancer Spine Pain: A Systematic Review and Meta-Analysis of Non-Randomized Studies

Faheem Malam^1^, Saad Asif^1^, Muhammad Khalid^1^, Cameron Leafloor^1^, Patrick Hong^2^, Tal Levit^1^, Dena Zeraatkar^3^, Li Wang^3^, Rachel Couban^3^, Arnav Agarwal^3^, Thomas Agoritsas^4^, Jason Busse^3^

^1^University of Ottawa, ^2^University of Toronto, ^3^McMaster University, ^4^University Hospitals of Geneva

**Introduction**: Clinicians frequently offer patients living with chronic spine pain interventional procedures, particularly in North America, such as joint or epidural injections with corticosteroids or anesthetics, medial branch blocks or radiofrequency ablation. We summarized the evidence on long-term and infrequent harms following interventional procedures for chronic non-cancer spine pain.

**Methods**: We searched MEDLINE, EMBASE and CINAHL from inception to February 2023 for non-randomized studies reporting on harms of interventional procedures administered to adults living with chronic axial or radicular non-cancer spine pain with ≥4 weeks of follow-up. We used random-effects models for all meta-analyses and the GRADE approach to evaluate the certainty of evidence.

**Results**: We included 60 longitudinal studies that enrolled 4,966 patients with chronic spine-related pain. Thirty-one studies investigated radiofrequency ablation or denervation, 22 epidural injections, and 11 joint injections or nerve blocks. Low certainty evidence suggests that interventional procedures for chronic spine pain may result in an increased prevalence of temporary altered level of consciousness (prevalence: 2.1%; 95%CI 0.7 to 4.1), deep infection (prevalence: 0.4%; 95%CI 0 to 1.5) and dural puncture (prevalence: 1.6%; 95%CI 0.2 to 3.7). Interventional procedures may increase prevalence of metabolic complications, and prolonged sensory deficits, pain or stiffness (prevalence ranged from 8.2% to 16.3%), but the supporting evidence was only very low certainty.

**Discussion/Conclusions**: Low certainty evidence suggests that interventional procedures for chronic spine pain may increase the prevalence of temporary altered level of consciousness, deep infection, and dural puncture. Other harms are uncertain due to very low certainty evidence.

## Pain Assessment in Dementia: A Systematic Review on the Measurement Properties of Observational Pain Tools

Andrew McLennan^1^, Emily Winters^1^, Michelle Gagnon^2^, Thomas Hadjistavropoulos^1^

^1^University of Regina, ^2^University of Saskatchewan

**Introduction**: Systematic reviews have synthesized the psychometric evidence on observational pain assessment tools for dementia populations. These reviews, however, are outdated and have not evaluated key psychometric properties (e.g., structural and content validity). Additionally, some reviews have not used standardized criteria, such as the COnsensus-based Standards for the selection of health Measurement INstruments (COSMIN; Mokkink et al., 2018). To address these gaps, we conducted a systematic review focused on assessing the psychometric properties and methodological quality of studies that have examined observational pain tools in dementia populations.

**Methods**: Search terms (e.g., assessment tool, pain, older adults) were used to systematically search four databases (i.e., Medline, PsycInfo, CINAHL, Web of Science) for all existing records up to December 2023, which yielded a total of 111 records after screening. The COSMIN criteria were used to evaluate the psychometric quality of evidence for each observational tool.

**Results**: We identified 26 observational tools, and most psychometric studies were of low quality. Compared to other measurement properties, evidence on content and structural validity was lacking for all observational tools. The Pain Assessment Checklist for Seniors with Limited Ability to Communicate-II (PACSLAC-II), Doloplus-2 and Pain Assessment in Advanced Dementia (PAINAD), have been studied more extensively across COSMIN-recommended measurement properties than other tools.

**Discussion/ Conclusions**: This study presents the state of psychometric evidence for observational pain tools used for people with dementia. Our findings will help clinicians and researchers identify tools for use, while also pointing out tools (and their specific measurement properties) that require further examination.

## Brain-Computer Interfaces as a Therapeutic Tool for Chronic Pain: A Mini-Review

Ariel Motsenyat^1^, Jiyeon Park^2^, Stevie D. Foglia^3^, Aimee J. Nelson^2, 3^

^1^Integrated Biomedical Engineering and Health Sciences, McMaster University, Hamilton, ON, Canada, ^2^Department of Kinesiology, McMaster University, Hamilton, ON, Canada, ^3^School of Biomedical Engineering, McMaster University, Hamilton, ON, Canada

**Introduction**: One in five Canadians live with neuropathic pain, with current treatments offering only temporary relief. Patients need a solution that targets the underlying neurological causes. Brain-Computer Interfaces (BCIs), particularly EEG-based systems, enable real-time neurofeedback to modulate neural activity involved in pain perception. The aim of our minireview was to identify the EEG-BCI protocols currently utilized, and their effectiveness in reducing pain symptoms in Fibromyalgia, Central Neuropathic Pain (CNP), and Complex Regional Pain Syndrome (CRPS).

**Methods**: A systematic literature review on PubMed identified eight studies that met inclusion criteria, two authors independently reviewed each article at each stage.

**Results**: Our review identified two EEG-BCI protocols for the treatment of pain symptoms: Sensorimotor Rhythm (SMR) training and Pain Frequency Band training. SMR training enhances the power of the SMR band, to reduce pain and associated symptoms. The Pain Frequency protocol increases alpha power and reduces theta and beta bands to normalize cortical activity. The protocols used visual or auditory feedback to promote self-regulation without explicit instructions. Both protocols showed significant reduction in pain-related symptoms, with SMR reducing fibromyalgia-related symptoms by 40%–71%, while the Pain Frequency Protocol reduced CNP-related symptoms by 22%–42%. Combining these protocols yielded promising results for Fibromyalgia.

**Discussion/Conclusions**: Although specific mechanisms remain vague, BCI-induced cortical changes likely enhance pain inhibitions and neuroplasticity. Future research should focus on standardizing EEG-BCI protocols for chronic pain, exploring combining pain treatment modalities (e.g., BCI + brain stimulation), and extending BCI applications to other chronic pain conditions such as CRPS.

## Pharmacologic Management of Acute Pain in Children: A Systematic Review and Network Meta-analysis

Laura Olejnik^1^, João Pedro Lima^1^, Behnam Sadeghirad^1^, Jason Busse^1^, Ivan Florez^2^, Samina Ali^3^, James Bunker^4^, Danny Jomaa^5^, Adam Bleik^6^, Mohamed Eltorki^6^

^1^McMaster University, ^2^University of Antioquia, ^3^University of Alberta, ^4^Western University, ^5^University of British Colombia, ^6^University of Calgary

**Introduction**: Acute pain affects 60% of children in pediatric emergency departments, with multimodal therapy recommended for management. This systematic review and network meta-analysis (NMA) evaluated the safety and efficacy of pharmacologic treatments for acute pediatric pain, providing evidence to inform clinical practice and guidelines.

**Methods**: We followed PRISMA guidelines and searched multiple databases up to October 2023 for randomized controlled trials involving children with acute pain, randomized to pharmacologic agents or placebo. Data were extracted and the risk of bias assessed by independent reviewers. We performed pairwise meta-analyses and NMA using random-effects models, with subgroup analyses by medication route and pain type. The GRADE approach was used to assess the certainty of evidence, categorizing interventions based on effectiveness and harm.

**Results**: We included 41 trials with 4,935 children. High to moderate certainty evidence showed that compared to placebo, nonsteroidal anti-inflammatory drugs (NSAIDs) (weighted mean difference [WMD], −1.29; risk difference [RD] for achieving the minimally important difference, 16%), ketamine (WMD, −1.12; RD, 14%), and mid-to-high potency opioids (WMD, −1.19; RD, 15%) effectively reduced pain. NSAIDs also reduced the need for rescue medication (RR, 0.31; RD, 16% fewer patients). NSAIDs and acetaminophen did not increase gastrointestinal adverse events. Other comparisons showed moderate-to-low certainty evidence of little to no difference from placebo.

**Discussion and Conclusions**: Compared to placebo, NSAIDs, ketamine, and mid-to high- potency opioids are effective in reducing acute pediatric pain. NSAIDs provide the greatest benefits and least harm, suggesting their use as first-line therapy.

## Effectiveness and Safety of Intra-Articular Interventions for Knee and Hip Osteoarthritis Based on Large Randomized Trials: A Systematic Review and Network Meta-Analysis

Tiago Pereira^1^, Pakeezah Saadat^2^, Pavlos Bobos^3^, Samir Iskander^3^, Nicolas Bodmer^2^, Martina Rudnicki^4^, Henry Kiyomoto^5^, Thais Montezuma^3^, Matheus Almeida^3^, Rishi Bansal^1^, Pai-Shan Cheng^2^, Jason Busse^6^, Alex Sutton^7^, Peter Tugwell^8^, Gillian Hawker^2^, Peter Juni^1^, Bruno da Costa^1^

^1^University of Oxford, ^2^University of Toronto, ^3^Western University, ^4^University College London, ^5^Faculty of the Americas, ^6^McMaster, ^7^University of Leicester, ^8^University of Ottawa

**Introduction**: We explored the effectiveness and safety of intra-articular interventions for knee and hip osteoarthritis (OA) through a systematic review and Bayesian random-effects network meta-analysis.

**Methods**: We searched CENTRAL and regulatory agency websites (inception-2023) for large, English-language, randomized controlled trials (RCTs) (≥100 patients/group) examining any intra-articular intervention. Pain and function outcomes were presented as standardized mean differences (SMDs) (95% credible intervals, 95% CrI). The prespecified minimal clinically important between-group difference (MID) was −0.37 SMD.

**Results**: Among 57 RCTs (22,795 participants) examining 18 intra-articular interventions, usual care or placebo, treatment effects were larger in 35 high-risk-of-bias trials than in 22 low/unclear-risk-of-bias trials. In the main analysis (excluding high-risk-of-bias trials), triamcinolone had the highest probabilities of reaching the MID for pain relief at weeks 2 and 6 (75% and 90%, respectively) with corresponding SMDs of −0.48 (95% CrI,-0.85 to −0.10) and −0.53 (95% CrI,-0.79 to −0.27) compared to placebo. The complex homeopathic products Tr14/Ze14 showed therapeutic potential at week 6 compared to placebo (SMD:-0.42, 95% CrI,-0.71 to −0.11, 64% probability of reaching the MID). Hyaluronic acid, platelet rich plasma, LMWF serum albumin, sprifermin, Fasitibant, Atelocollagen, and polyacrylamide hydrogel, had no effect on pain greater than placebo.

**Discussion/Conclusions**: Large RCTs with lower risk of bias indicate that the effects of commonly administered intra-articular interventions for knee or hip OA are consistent with placebo effects.

## Cultural Variations in the Affective Responses to Physical Pain: Clinical Implications and Future Research Directions

Marie-Pier Plouffe Demers^1^, Stéphanie Cormier^2^, Camille Saumure^3^, Daniel Fiset^2^, Caroline Blais^2^

^1^University of Quebec in Montreal, ^2^University of Quebec in Outaouais, ^3^University of Fribourg

**Introduction**: This systematic review, conducted in accordance with PRISMA guidelines, provides a comprehensive overview of current knowledge on how cultural environments shape pain communication. The review examines each stage of the pain communication process – from the origin of pain to its expression – including conceptualization, experience, regulation, and expressivity.

**Methods**: From an initial pool of approximately 700 articles, 59 studies were selected for detailed analysis and quality assessment. To facilitate interpretation, the review classified cultural groups examined in these studies according to Schwartz’s seven transnational cultural groupings (2004; i.e., West Euro, East Euro, English-speaking, Latin American, South Asian, Confucian and sub-Saharan African nations) with an additional category for Middle Eastern nations, as per Gupta and Hanges (2004).

**Results**: The findings reveal significant gaps in the literature, notably the absence of standardized tools for assessing the affective dimension of pain across cultures, with nearly 40 different instruments identified. Furthermore, the heavy reliance on self-reported measures, primarily questionnaires, which were developed in specific language and culture complicates the identification of genuine cultural differences. The review also highlights an underrepresentation of certain world regions, particularly Sub-Saharan Africa and Latin America.

**Discussion/Conclusions**: Based on these insights, recommendations were developed and will be reviewed by a panel of expert clinicians and researchers using the Delphi method. This structured communication technique facilitates consensus-building among experts on complex topics. In conclusion, this review underscores the need for a systematic, culturally sensitive approach to pain communication research to improve validity and inform clinical practices that reflect diverse cultural contexts.

## Effectiveness and Safety of Dry Needling for Upper-Quarter Myofascial Pain: A Systematic Review and Meta-Analysis

Jeremy P Steen^1^, Karen Mao^2^, Shane T Claffey^2^, Deepa Dhillon^2^, Cecilia Massis^3^, Kishore Jaiswal^3^, Cynthia Chui^4^, Dinesh Kumbhare^1^

^1^Institute of Health Policy, Management and Evaluation, Dalla Lana School of Public Health, University of Toronto, Toronto, ON, Canada, ^2^University of Illinois College of Medicine, Chicago, IL, USA, ^3^Faculty of Health Sciences, Queen’s University, Kingston, ON, Canada, ^4^Library and Information Services, University Health Network, Toronto, ON, Canada

**Introduction**: Myofascial pain syndrome (MPS) is a common source of musculoskeletal pain and is often resistant to conventional treatments such as exercise and medication. Dry needling has emerged as a promising treatment option for upper-quarter MPS. Existing systematic reviews have limitations, leaving uncertainty about its effectiveness and safety. This systematic review and meta-analysis evaluated the effectiveness and safety of dry needling for upper-quarter MPS.

**Method**: We searched seven databases and trial registries through June 26, 2024, for randomized trials comparing dry needling to sham or no intervention in adults (≥18 years) with upper-quarter MPS. We conducted random-effects meta-analyses for patient-important outcomes at short-term follow-up (0 to 24 hours after end of the intervention), medium-term follow-up (7 to 21 days), and long-term follow-up (>21 days). We performed a priori subgroup analyses to explore heterogeneity and assessed the certainty of evidence using GRADE.

**Results**: We included 10 trials (384 participants). The frequency and duration of dry needling interventions ranged from a single session to 6 sessions over 4 weeks. Compared to sham, moderate-certainty evidence shows that dry needling probably reduces pain at short-term follow-up by 2.41 points on a 10-point Visual Analogue Scale (VAS) (95% CI −3.20 to −1.63). A greater reduction was observed at medium-term follow- up (MD −2.67, CI −3.27 to −2.07; moderate-certainty). Results were robust to worst plausible assumptions regarding pain relief in studies with missing outcome data. No data were available at long-term follow-up. Reported adverse events were mild and transient.

**Discussion/Conclusions**: Dry needling likely reduces pain in adults with upper-quarter MPS at short- and medium-term follow-up.

## Shedding Light On Alternative Strategies for More Effective Pain Management: A Translational Systematic Review of Light Therapy for Chronic Pain

Doriana Taccardi^1^, Hailey GM Gowdy^1^, Emily Sharp^1^, Prisha Adya^1^, Tracy Cupido^2^, M. Gabrielle Pagé^3^, Nader Ghasemlou^1^

^1^Queen’s University, ^2^Kingston Health Sciences Center, ^3^Université de Montréal

**Introduction**: People experiencing chronic pain often describe rhythmic fluctuations of pain intensity throughout the day. Recent evidence suggests circadian rhythmicity is an important factor in the biopsychosocial profiles of people with chronic pain. Treatment options for chronic pain are lacking, often relying on pharmacology; circadian rhythmicity presents a novel target for alternative therapies. Light therapy has been explored as one such treatment, but there is little consensus on its use in both clinical and pre-clinical settings.

**Methods**: We conducted a translational systematic review of both preclinical and clinical studies, registered with PROSPERO (CRD42023429231), to address the effects of light therapy on chronic pain. Only studies evaluating the effect of light-/phototherapy, using any type of light acting on photoreceptors, were included; while those applying light to other sites (e.g., the skin) were excluded from this review. Risk of bias was assessed using the Cochrane tool for clinical studies and the SYRCLE tool for preclinical.

**Results**: Of 6802 records screened, 16 studies were extracted, including 11 clinical and 5 preclinical studies. Overall, light therapy was found to improve chronic pain outcomes in most preclinical and clinical studies, prompting the need for further studies. Specific findings for clinical and preclinical studies are discussed in detail.

**Discussion/Conclusions**: Findings from this review will help guide the setup of our new CIHR-funded innovative clinical trial (iCT) to evaluate the effectiveness of light therapy as an individualized strategy to treat chronic pain. This will further inform the individual responsiveness to specific therapies through modulation of circadian rhythms.

## Defining Advice to Stay Active in Low Back Pain or Back-Related Leg Pain—A Scoping Review

Pedro Victor Tavares Gregorio^1^, Tatiana Grasser^2^, Javier Muñoz Laguna^3^, Felipe José Jandre dos Reis^4^, Leticia Rangel^1^, Leandro Alberto Calazans Nogueira^5^, Ney Meziat-Filho^6^

^1^Postgraduate Program in Rehabilitation Sciences, Augusto Motta University Center, Rio de Janeiro, Brazil, ^2^Instituto Federal do Paraná, Paraná, Brazil, ^3^Epidemiology, Biostatistics and Prevention Institute (EBPI), University of Zurich, Zurich, Switzerland, ^4^Instituto Federal do Rio de Janeiro, Rio de Janeiro, Brazil; School of Physical and Occupational Therapy, McGill University, ^5^Postgraduate Program in Rehabilitation Science, Centro Universitário Augusto Motta, Rio de Janeiro, Brazil, ^6^School of Rehabilitation Science, Faculty of Health Sciences, Institute for Applied Health Sciences, McMaster University, Hamilton, Ontario, Canada; Postgraduate Program in Rehabilitation Sciences, Augusto Motta University Center – UNISUAM, Rio de Janeiro, Brazil.

**Introduction**: Low back pain with or without back-related leg pain is a major cause of disability. “Advice to stay active” is a guideline-recommended intervention but is often vaguely defined, creating a knowledge gap in musculoskeletal health. The aim of this study was to map, characterize, and define “advice to stay active” interventions for low back pain or back-related leg pain.

**Methods**: A scoping review was conducted using JBI model searching Cochrane, PEDro, PubMed, Scopus, and Web of Science. No language restrictions were applied. Eligible study designs were randomized clinical trials, study protocols, systematic reviews, and guidelines. Data extraction included author, year, study design, aims, definition of “advice to stay active,” arms of intervention and control description, where was “advice to stay active” in arm of intervention, sample and country.

**Results**: Fifty-two articles were included, revealing variability in how “advice to stay active” was presented. The most frequent components were grouped into four categories: good prognosis (n = 18; 34.62%), performing daily activities (n = 17; 32.69%), coping despite pain (n = 11; 21.25%), and gradual return (n = 9; 17.31%).

**Discussion/Conclusions**: Most studies provided incomplete definitions or omitted key components identified in this research. The four main components: good prognosis, daily activity performance, coping with pain, and gradual return can facilitate behavior change, promote self-efficacy, and support recovery. The most common elements of ‘advice to stay active’ in academic literature are good prognosis, performing daily activities, moving despite pain, and gradual return.

## Co-design of Visualization and Stories for Digital Literacy: An Integrated Digitally-Enabled Care Trajectory for Chronic Pain

Regina Visca^1^, Caroline Dumouchel-Hudon^2^

^1^McGill University Health Center, ^2^CHUM

**Introduction**: In Quebec, chronic pain management poses significant challenges for individuals in remote areas and an aging population with limited mobility, who face long wait times and geographic barriers. To improve accessibility, coordination, continuity and patient engagement, Quebec has integrated innovative digital technologies – virtual care coordination and telemonitoring – into chronic pain management pathways. However, a key barrier to its adoption is digital literacy. The aim of this study is to understand gaps in digital health literacy, define design principles to conceptualize and visualize digital health as narratives and understand its benefits.

**Methods**: Semi-structured focus groups were conducted with patients, clinicians, designers, telehealth experts, and decision-makers who were purposively recruited from pain and telehealth networks between June to November 2024. A descriptive qualitative research design was used to identify gaps in digital literacy and design principles to address these gaps.

**Results**: Participants (N = 12) identified gaps in digital literacy, mainly understanding: 1) new technologies and how they can be integrated in clinical processes; 2) value proposition of the technology including care improvement, workflow efficiencies; 3) importance of data and how it will be used. Design principles included the use of: 1) empathy to understand/express emotions; 2) visual aesthetics to share meaningful/compelling information and stories; 3) storyboards to share a patient-centered vision that clarifies patient journeys, providers’ roles and technology integration.

**Discussion/Conclusions**: The co-creation of storyboards not only helps clarify the value of technology in care pathways but also ensures equity by increasing digital literacy. It helps patients to better understand how digital health enables their care by visualizing the steps involved, thus reinforcing engagement in the care process. Patients are also empowered by using visual aids to explain their journey to their loved ones. Finally, by making roles more visible, storyboards enhance collaboration/partnership between patients and providers, facilitating informed decisions.

## Predictors of Chronic Postsurgical Pain after Cardiac Surgery: A Systematic Review and Meta-Analysis of Observational Studies

Li Wang^1^, Henry Kwon^2^, Gwendolyn Lovsted^1^, Sara Ghazizadeh^3^, Alireza Malektojari^3^, Shaunattonie Henry^4^, Rachel J. Couban^5^, Jason W. Busse^1^, Michael McGillion^4^

^1^Department of Anesthesia, McMaster University, Hamilton, ON, Canada, ^2^Wayne State University School of Medicine, Detroit, Michigan, United States, ^3^Hormozgan University of Medical Sciences, Bandarabbas, Iran, ^4^School of Nursing, McMaster University, ^5^Michael G. DeGroote Institute for Pain Research and Care, McMaster University.

**Introduction**: We aimed to conduct a systematic review and meta-analysis of observational studies to identify predictors of CPSP after cardiac surgery.

**Methods**: We searched MEDLINE, EMBASE, PsycInfo, and CINAHL to identify cohort and case-control studies, in any language, that explored predictors of CPSP after cardiac surgery. Paired reviewers screened literature, assessed risk of bias, and extracted data. We conducted random-effects meta-analysis.

**Results**: Twenty-nine observational studies with 14,344 cardiac surgery patients were eligible. The pooled prevalence of CPSP at any severity is 23% (95%CI 17% to 30%), and 8% (95%CI 6% to 12%) for moderate-to-severe CPSP. Moderate- to high-certainty evidence shows that CPSP after cardiac surgery is associated with older age (adjusted odds ratio[OR] 1.27 on every 10-year increase, 95%CI 1.11 to 1.44), female sex (OR 1.42, 95%CI 1.10 to 1.83), greater BMI (OR 1.13 on every 1-point increase, 95% CI 1.01 to 1.27), acute postoperative pain (OR 1.33 on every 1-point increase on a 0–10 NRS,95% CI 1.07 to 1.66), opioid consumption (OR 1.15 on every 10-mg increase, 95%CI 1.01 to 1.30), previous cardiac surgery (OR 2.07, 95% CI 1.19 to 3.59), baseline anxiety (OR 3.12, 95% CI 2.24 to 4.35) and depression (OR 1.97, 95% CI 1.43 to 2.70).

**Discussion/Conclusions**: Baseline anxiety, depression, history of cardiac surgery, older age, female, greater BMI, more severe acute postoperative pain, and greater opioid consumption are associated with CPSP after cardiac surgery. Early identification of high-risk population and early intervention is important to reduce the risk of CPSP after cardiac surgery.

## The Efficacy and Safety of Ketorolac as an Adjuvant for Regional Anesthesia Techniques – A Systematic Review of Randomized Controlled Clinical Trials

Alexander Xiang^1^, Sarah Lopes Sadafi^2^, Vivek Patil^1^, Jenny Wang^2^, Anushka Patel^2^, Ella Ying^2^, Li Wang^3, 4^

^1^Department of Medicine, McMaster University, Hamilton, Ontario, Canada, ^2^Faculty of Health Sciences, McMaster University, Hamilton, Ontario, Canada, ^3^Michael G. DeGroote Institute of Pain Research and Care, McMaster University, Hamilton, Ontario, Canada, ^4^Department of Health Research Methods, Evidence, and Impact, McMaster University, Hamilton, Ontario, Canada

**Introduction**: Adjuvants to local anesthetics in regional anesthesia have been used to improve block duration and postoperative pain control; however, their effects remain controversial. This systematic review evaluated the efficacy and safety of ketorolac as an adjuvant in regional anesthetic techniques.

**Methods**: We searched Medline, EMBASE, CINAHL, and CENTRAL to identify randomized controlled trials (RCTs) examining ketorolac added to local anesthetics versus local anesthetic alone in regional anesthesia (RA). We conducted a random-effects meta-analysis and a priori subgroup analyses.

**Results**: Twenty-six RCTs and 1,547 patients were included. Ten RCTs examined peripheral nerve blocks, 14 examined IV regional anesthesia, and two examined neuraxial anesthesia. Moderate certainty evidence shows ketorolac added to local anesthetics, compared to local anesthetics alone, probably reduces postoperative pain at 1 hour (weighted mean difference [WMD] −1.96 on a 0–10 numerical rating scale, 95% CI −3.09 to −0.83), at 2 hours (WMD −1.88, 95% CI −2.36 to −1.40), prolongs time to first analgesia (WMD 2.21 hours, 95% CI 1.05 to 3.38) and block duration (WMD 125.76 minutes, 95% CI 11.92 to 239.61), without significant increases in adverse events (relative risk 0.84, 95% CI 0.54 to 1.30). Low certainty evidence shows no significant reduction in overall opioid consumption (WMD −4.5 mg, 95% CI −9.66 to 0.65).

**Discussion/Conclusions**: Ketorolac as an adjuvant in regional anesthesia probably improves postoperative pain control, prolongs time to first analgesic request and block duration, without significant increases in adverse events. Future research on the comparative utility of different adjuvants for regional anesthesia is needed.

## Interventions for the Management of Long COVID (Post-COVID condition): Living Systematic Review

Dena Zeraatkar^1^, Michael Ling^1^, Sarah Kirsh^1^, Tanvir Jassal^1^, Mahnoor Shahab^1^, Hamed Mohaved^1^, Jhalok Ronjan Talukdar^1^, Alecia Walch^1^, Samantha Chakraborty^2^, Tari Turner^2^, Lyn Turkstra^2^, Roger S. McIntyre^3^, Ariel Izcovitch^4^, Lawrence Mbuagbaw^1^, Thomas Agoritsas^1^, Signe A. Flottorp^5^, Paul Garner^6^, Tyler Pitre^3^, Rachel Couban^1^, Jason W. Busse^1^

^1^McMaster University, ^2^Monash University, ^3^University of Toronto, ^4^Universidad del Salvador, ^5^Norwegian Institute of Public Health, ^6^Liverpool School of Tropical Medicine

**Introduction**: Although most patients recover from COVID-19, up to 15% experience long-term health effects, including fatigue and impaired cognitive function, called Long COVID. Considerable resources have been invested to study Long COVID, resulting in several published clinical trials and many more planned or ongoing. We are establishing and maintaining a living systematic review to provide trustworthy summaries of evidence on the effectiveness of interventions for managing Long COVID.

**Methods**: We searched research databases, up to December 2023, for trials that randomized adults with Long COVID to pharmacologic or nonpharmacologic interventions, placebo, or usual care. Reviewers worked independently and in duplicate to screen search records, extract data, and assess risk of bias. We summarized the findings of these trials and assessed the certainty of evidence using the GRADE approach.

**Results**: 24 trials with 3,695 patients proved eligible. Moderate certainty evidence suggested that, compared with usual care, an online program of cognitive behavioral therapy (CBT) probably reduces fatigue and improves concentration. Moderate certainty evidence also suggested that a physical and mental health rehabilitation program likely leads to improvements in overall health and quality of life and reduces symptoms of depression.

We did not find compelling evidence supporting the effectiveness of other interventions, including drug or dietary interventions and medical devices and technologies.

**Discussion/Conclusions**: Our findings suggest that offering patients CBT or physical and mental health rehabilitation will likely improve symptoms. Current guidance on managing Long COVID is limited and largely consensus-based. We trust that this review will inform future guideline recommendations.

## Fundamental SciencesWhen Trust in Physicians Increases Helplessness: The Role of Illness Uncertainty in Chronic Pain Management

Shokouh Abolhosseini^1^, Madelaine Gravelle^1^, Sophie McNamee^1^, Etienne Bisson^2, 3^, Tim Salomons^1^

^1^Department of Psychology, Queens University, Kingston, ON, Canada, ^2^Department of Anesthesiology and Perioperative Medicine, Queen’s University, Kingston, ON, Canada, ^3^Chronic Pain Clinic, Kingston Health Sciences Center, Hotel Dieu site, Kingston, ON, Canada

**Introduction**: People with chronic pain often experience feelings of helplessness due to the uncertainty surrounding their condition and treatment options. However, trust in one’s physician may be a protective factor, as greater trust can mitigate the negative aspects of an uncertain diagnosis. We hypothesized that illness uncertainty would play a key role in the relationship between pain severity and helplessness in adults with chronic pain, and that higher trust in physicians (TIP) would reduce the impact of pain severity on uncertainty.

**Methods**: We conducted a cross-sectional study between October 2023-June 2024. Adults with chronic pain were recruited from an interdisciplinary chronic pain clinic. Participants (N = 199) completed an online survey via Qualtrics. Data was analyzed using moderated mediation in SPSS.

**Results**: The significant mediation model showed that pain predicted illness uncertainty (R^2^ = .30, F(4,194) = 20.77, p < .001), which predicted helplessness (R^2^ = .18, F(3,195) = 14.13, p < .001). The index of moderated mediation was also significant (b = 0.03, 95%CI[0.00, 0.07]); high TIP was associated with a stronger relationship between pain and uncertainty (b = 0.47, 95%CI [0.06, 0.98]), while low and moderate TIP did not significantly affect this relationship.

**Discussion/Conclusions**: Contrary to our hypothesis, higher TIP may inadvertently increase feelings of illness uncertainty and helplessness in individuals with chronic pain. People with high TIP may rely more on medical guidance and experience greater uncertainty when their pain remains unmanaged. Thus, increasing trust without addressing underlying uncertainties may unintentionally increase distress. Future research should explore strategies to manage expectations and enhance patient-provider communication to reduce uncertainty-driven helplessness in people with chronic pain.

## Perineuronal Nets and Their Impact on Pain Modulation in Chemotherapy-Induced Peripheral Neuropathy

Allutas Alhamwi^1, 2^, Hannah Cho^1, 2^, Nicole Scher^1, 3^, Emerson Krock^1, 2^

^1^Alan Edwards Center for Research on Pain, ^2^Faculty of Dental Medicine and Oral Health Sciences McGill University, Montreal, Quebec, ^3^Department of Biochemistry, McGill University, Montreal, Quebec

**Introduction**: Chemotherapy-induced peripheral neuropathy (CIPN) is a prevalent side effect of chemotherapeutics, like cisplatin, and affects up to 30% of patients long after treatment completion. Cisplatin accumulates in the dorsal root ganglia (DRG) and causes neurotoxic effects, which lead to persistent pain. Despite its prevalence, the mechanisms behind cisplatin-induced pain are not fully understood. Perineuronal nets (PNNs), a specialized extracellular matrix network known to regulate neuronal activity in the brain and spinal cord, may play a role in this context. However, PNNs’ role in primary nociceptor function remains unclear. In this study, we hypothesize that cisplatin stimulates immune cells to degrade DRG PNNs, leading to long-lasting neuronal hypersensitivity and pain. Objective: Determine how cisplatin induces pain-like behavior through immune cell- mediated DRG PNN degradation.

**Methods**: Male and female mice received 2 mg/kg cisplatin for 3 consecutive days. Pain-like behavior was assessed using von Frey and Hargreaves tests. TIMP1 was administered intrathecally to inhibit PNNs degrading protease activity and neutrophils were depleted with an anti-Ly6G antibody. DRG immune cells and PNNs were analyzed using immunofluorescence microscopy.

**Results**: Cisplatin-induced mechanical and thermal hypersensitivity, and a loss of PNNs and collagen I in the DRG. Cisplatin also increased macrophage proliferation and neutrophil infiltration. Blocking proteases with TIMP1 or depleting neutrophils reduced pain-like behavior and preserved PNN integrity.

**Discussion/Conclusions**: Determining the role of PNNs in the DRG will clarify how cisplatin-driven PNNs remodeling links to CIPN and pain. Our findings suggest that preventing PNNs degradation in the DRG reduces pain-like behavior, likely through immune cell involvement.

## Investigating the function of APOE polymorphisms in chronic pain

Nicole Brown^1^, Noe Francois-Saint-Cyr^1^, Volodya Hovhannisyan^1^, Arkady Khoutorsky^1^

^1^Department of Anesthesia and Faculty of Dentistry, McGill University, Canada.

**Introduction**: Single-cell RNA sequencing of microglia identified Apolipoprotein E (Apoe) as the top upregulated gene in spinal cord microglia at chronic stages following peripheral nerve injury in mice. APOE regulates neuroimmune functions, synaptic activity, and aging. Humans express three APOE isoforms: APOE-ε2, APOE-ε3 and APOE- ε4. APOE-ε4 is the strongest genetic risk factor for Alzheimer’s disease. Our previous work demonstrated that APOE-ε2 carriers have significantly higher risk to develop chronic pain, whereas carriers of APOE-ε4 have lower risk to develop chronic pain.

**Methods**: To test the role of ApoE polymorphisms in chronic pain, we used humanized mice expressing APOE-ε2, APOE-ε3 or APOE-ε4 (KI). For cell-type specific effects in neuropathic pain, these knock-in (KI) mice were crossed to a Cre inducible microglial cell line (TMEM119) to create a conditional knock out (cKO) model. We implemented spared nerve injury (SNI), a model for neuropathic pain, on both KI and cKO mice.

**Results**: Following SNI, APOE-ε4 mice showed less nerve-injury induced cold hypersensitivity, while APOE-ε2 mice showed increased spontaneous pain. APOE-ε2 TMEM119 cKO mice demonstrated less mechanical hypersensitivity than APOE-ε2 KI mice. APOE-ε2 microglia displayed fewer branch number and lower process length, suggesting a reactive state. Furthermore, APOE-ε2 mice showed higher percentage of CD68 in microglia.

**Discussion/Conclusions**: These results reinforce our hypothesis that APOE-ε2 increases risk for neuropathic pain while APOE-ε4 may be protective. APOE-ε2 microglia appear to be more reactive and phagocytic which may be contributing to chronic pain, whereas APOE-ε4 microglia are homeostatic and may suggest a protective phenotype.

## Pain and Autonomic Function: The Relationship Between Conditioned Pain Modulation and Cardiovagal Baroreflex Sensitivity

Laila Chaudhry^1^, Charlotte Usselman^1^, Jeffrey Mogil^1^

^1^McGill University

**Introduction**: Conditioned pain modulation (CPM) is considered to measure the “capacity” of human endogenous pain inhibitory mechanisms and is elicited using a “pain inhibits pain” paradigm. Alternatively, stress can also inhibit pain. Evidence suggests that blood pressure (BP)-regulating baroreceptors mediate stress-induced analgesia: naturally or experimentally increased BP stimulates baroreceptors, producing descending pain inhibition. Given this evidence, we can assume that baroreceptor mechanisms (i.e., baroreflex) are involved in pain modulation, yet we do not know if it is involved in CPM specifically. Therefore, the purpose of this study was to determine the relationship between the baroreflex and CPM.

**Methods**: Participants (n = 5) were instrumented to measure heart rate (HR), BP, and respiration, underwent 10 min of baseline quiet rest, then lastly a CPM protocol consisting of: two suprathreshold heat stimulations, followed by a 30-s cold pressor test, then a final heat stimulation. We used baseline BP and HR values to calculate cardiovagal baroreflex sensitivity (BRS) via the sequence method. Up-sequences, a marker for parasympathetic activation, are 3+ concurrent increases in systolic BP (SBP) and decreases in HR; down-sequences, or 3+ concurrent decreases in SBP and increases in HR, are considered a marker for parasympathetic withdrawal. BRS was then stratified by up- and down-sequences and regressed against absolute values of CPM hypoalgesia.

**Results**: CPM displayed a significant negative correlation with down-sequences (r = −0.93, p = .02).

**Discussion/Conclusions**: This implies that prolonged parasympathetic activity, via less efficient withdrawal, is associated with greater CPM hypoalgesia and may serve as a valuable autonomic target for chronic pain therapies.

## Ca^2+^ Activity Dynamics in the Spinal Cord Are Altered in Acute and Chronic Pain Models

Samuel W. Fung^1, 2^, Erika K. Harding^1, 2, 3^, Jo Anne Stratton^4^, Stephanie M. Norlock^5^, Jenny K. Cheung^1^, Hantao Zhang^1^, Julieanne Dalsgaard^1^, Michael E. Hildebrand^5^, Robert P. Bonin^1,6,7,8^

^1^Department of Pharmaceutical Sciences, Leslie Dan Faculty of Pharmacy, University of Toronto, Toronto, Ontario, Canada, ^2^These authors contributed equally., ^3^Department of Clinical Neurosciences, University of Calgary, Calgary, Alberta, Canada, ^4^Department of Neurology and Neurosurgery, Montreal Neurological Institute-Hospital, McGill University, Montreal, Quebec, Canada, ^5^Department of Neuroscience, Carleton University, Ottawa, Ontario, Canada, ^6^Department of Cell and Systems Biology, Faculty of Arts and Science, University of Toronto, Toronto, Ontario, Canada, ^7^University of Toronto Center for the Study of Pain, University of Toronto, Toronto, Ontario, Canada, ^8^Corresponding Author

**Introduction**: Understanding the development and maintenance of pathological pain is critical for the discovery of therapeutic targets. Different forms of pathological pain alter nociceptive processing in the superficial dorsal horn (SDH) of the spinal cord through distinct mechanisms, but these differences are poorly understood. The purpose of this project was to develop and apply an ex-vivo SDH Ca^2+^ imaging pipeline to screen different forms of pathological pain.

**Methods**: We employed several models of pathological pain: acute inflammatory (Capsaicin), protracted inflammatory (Complete Freud’s Adjuvant, CFA), neuropathic (Spared Nerve Injury, SNI), and osteoarthritic (ACL transection, OA). We captured network wide SDH dynamics of spontaneous and evoked cellular activity, under these pain conditions, using epifluorescent Ca^2+^ imaging in an ex-vivo approach. To confirm this approach could elucidate alterations in cellular Ca^2+^ responses, we applied blockers of GABAergic and glycinergic receptor mediated inhibition (bicuculline and strychnine), a model of disinhibition.

**Results**: When glutamate was applied to stimulate activity, cells in slices treated with bicuculline and strychnine experienced a heightened Ca^2+^ response compared to control. Under glutamate evoked conditions, we found that the SDH neurons from Capsaicin, CFA, and SNI models similarly exhibited a higher amplitude of Ca^2+^ response compared to controls, while the OA model did not elicit a difference in response. Unexpectedly, the number of spontaneously active neurons, in the absence of glutamate, was significantly decreased in the OA and CFA models. Since spinal astrocytic activation has been previously implicated in OA and CFA, we turned to paclitaxel, which has been known to selectively activate astrocytes. An intrathecal injection of paclitaxel-induced spinal astrocytic expression and peripheral hypersensitivity, which was reversed with the astrocytic gap junction inhibitor, carbenoxolone.

**Discussion/Conclusions**: Collectively, our findings provide further insight into the diverse manifestations of inflammatory, neuropathic, and osteoarthritic pain across different time scales in the spinal cord.

## Exploring Avoidance and Approach Learning in Uncertain Environments: A Computational Study of Pain and Reward Learning

Omar Khalil^1, 2, 3^, Clizia Martini^4^, Massieh Moayedi^1, 2, 3, 5^, Andreea O Diaconescu^4, 6, 7, 8^

^1^Centre for Multimodal Sensorimotor and Pain Research, Faculty of Dentistry, University of Toronto, Toronto, ON, Canada, ^2^University of Toronto Center for the Study of Pain, University of Toronto, Toronto, ON, Canada, ^3^Division of Clinical and Computational Neuroscience, Krembil Brain Institute, Toronto Western Hospital, University Health Network, Toronto, ON, Canada, ^4^Department of Psychology, University of Toronto, ^5^Department of Dentistry, Mount Sinai Hospital, Toronto, ON, Canada, ^6^Institute of Medical Sciences, University of Toronto, Toronto, ON, Canada, ^7^Krembil Center for Neuroinformatics, Center for Addiction and Mental Health (CAMH), Toronto, ON, Canada, ^8^Department of Psychiatry, University of Toronto, Toronto, ON, Canada

**Introduction**: Decision making in uncertain environments requires individuals to adjust behavior based on outcomes, a process guided by reinforcement learning. Although previous studies have explored appetitive and aversive learning, few have done so in uncertain contexts, often relying on reinforcement learning models with fixed learning rates. We use the Hierarchical Gaussian Filter (HGF) to model adaptive learning and compare appetitive, aversive, and interactive learning behaviors.

**Methods**: A total of 128 participants (67 F) completed a probabilistic reinforcement learning task, assigned to one of three groups: reward-learning (monetary gain), pain-avoidance learning (thermal pain), or approach-avoidance learning (reward and pain outcomes). We modeled learning using a mean-reverting HGF and analyzed group- and sex-level differences. Additionally, we examined associations between learning parameters and psychological traits [BAS-Drive, Fear of Pain (FPQ), Pain Catastrophizing Scale (PCS)].

**Results**: A significant difference in omega 2 (ω2), a proxy for vigilance, was observed between groups, with pain-avoidance showing higher ω2 (p = .042). A sex difference in ω2 suggested that this group effect was driven by males (p = .018). No significant associations emerged between ω2 and psychological traits in the reward-learning and approach-avoidance groups, but ω2 was significantly positively correlated with FPQ in the pain-avoidance group (p = .01).

**Discussion/Conclusions**: Our findings suggest that the presence of pain alone leads to the highest vigilance, possibly driven by males, while the presence of monetary rewards may shift value and attention away from pain, reducing vigilance. Within the pain-avoidance group, a positive correlation between FPQ and ω2 suggests that fear of pain may underlie increased vigilance.

## Dissecting Neuroimmune Interactions: Deciphering Cellular Crosstalk and the Role of Neuropeptides in Modulating Immune and Host Cell Responses

Nandita Menon^1^, Dr. Anil Kishen^1, 2^

^1^University of Toronto ^2^Mount Sinai Hospital

**Introduction**: Neuropeptides Substance P (SP), Calcitonin Gene-Related Peptide (CGRP) are known to regulate inflammation and pain. However, their context-dependent effects and downstream signaling pathways remain unclear. This study investigates how neuron-derived neuropeptides shape immune responses and explore the contributions of host tissue to inflammation and pain.

**Methods**: We simulated inflammation in vitro by exposing neurons to lipopolysaccharide (LPS) and analyzed the effects of their conditioned media on macrophages and periodontal ligament (PDL) cells. Neuropeptide receptor inhibitors were used to assess the contributions of SP and CGRP. Additionally, neurons were exposed to conditioned media from macrophages and PDL cells to study reciprocal interactions. Changes in cytokine expressions, macrophage markers, and TRP channel regulation were assessed.

**Results**: Neurons released CGRP in response to LPS which exhibited anti-inflammatory properties but triggered a proinflammatory response in macrophages. Blocking SP and CGRP signaling revealed that these upregulated CD68 expression in macrophages, leading to increased IL-1β and TGF-β1 secretion. Conversely, exposure of neurons to conditioned media from inflammatory macrophages and PDL cells induced a proinflammatory shift, upregulating TRPV1 but downregulating TRPA1, proposing immune-driven pain signaling. SP, CGRP specifically promoted TRPV1 expression while IL-10 regulated TRPA1 in neurons.

**Discussion/Conclusions**: Our study dissects neuroimmune interactions by specifically investigating the role of neuropeptides on inflammation and pain signaling. Neuronal CGRP mediates immune cell activation, while immune-derived signals affect neuronal excitability and pain pathways. We also highlight the role of host tissue in shaping neuroimmune interactions, providing a comprehensive understanding of the cellular crosstalk that drives inflammation and pain.

## Trajectories and Predictors of the Long-Term Course of Chronic Low Back Pain: A Longitudinal Cohort Study of the Quebec Back Pain Consortium

Pouya Rabiei^1, 2^, Michel-Pierre Coll^2, 3^, Pierre Langevin^4^, Marc-Olivier Dubé Dubé^2, 5^, Jean- Sébastien Roy^2, 5^, Hugo Masse-Alarie^2, 5^

^1^Faculty of Medicine, Université Laval, Québec, QC, Canada, ^2^Centre Interdisciplinaire de Recherche en Réadaptation Et Intégration Sociale (Cirris), Québec, QC, Canada, ^3^École de Psychologie, Université Laval, Canada, ^4^Faculty of Medicine, School of Rehabilitation Sciences, ^5^School of Rehabilitation Sciences, Faculty of Medicine, Université Laval, Quebec City, Quebec, Canada

**Introduction**: Chronic low back pain (LBP) poses a significant healthcare burden. It is crucial to explore whether distinct pain trajectories exist and what factors influence their progression once pain becomes chronic. This study aims to describe chronic LBP trajectories over 2 years and identify key predictors of their evolution.

**Methods**: We analyzed data from 1,490 chronic LBP patients from the Quebec Low Back Pain Consortium, using variables from the Canadian minimal dataset collected at baseline. Pain intensity was measured at 3, 6, 12, and 24 months. Latent class growth analysis (LCGA) was utilized to identify pain trajectories. Machine learning classifiers were employed to subgroup individuals based on these trajectories and personal characteristics.

**Results**: LCGA identified four trajectories: persistent high pain (50%), moderate pain with slow decline (33%), mild stable pain (13%), and progressive recovery (3%). Cluster membership probabilities were high (>0.90) except for Progressive Recovery (0.70). Logistic regression was the most effective classifier (AUC = 0.74, p = .009). Pain duration and sex were predictors for persistent high pain; physical function and comorbidity influenced moderate pain with slow decline; depression was linked to mild stable pain. No significant predictors emerged for progressive recovery.

**Discussion/Conclusions**: Findings indicate that while most individuals with chronic LBP remain stable, a minority show significant improvement. Interestingly, the literature has not yet identified a pain trajectory considered as recovery in chronic pain. However, no clear characteristics explained their recovery, likely due to sample size limitations. Targeting pain-related and psychological factors may help prevent long-term pain progression.

## Pain Perception in the Context of the Exploration-Exploitation Dilemm

Nicolas Roy^1, 2^, Michel-Pierre Coll^1, 2^

^1^Université Laval, ^2^Cirris

**Introduction**: The present research project aims to test the hypothesis that subjective pain perception is modulated by learning. One of the main functions of pain is to help us learn about our environment and guide our behaviors to minimize pain. However, minimizing pain does not always mean avoiding it. Often, pain allows us to acquire new information, giving it an informational value. Thus, pain perception should be modulated based on its informational value.

**Methods**: Participants (n = 42) completed a 200-trial learning and exploration task using a four-armed restless bandit model. They had to choose between several options associated with electric pain stimulations, with intensity varying on each trial according to a Gaussian random walk. Their instruction was to reduce their perceived pain, and they were aware that the intensities changed gradually on each trial.

**Results**: A Q-Learning reinforcement learning model was used to model participants’ learning and allowed for the categorization of each decision as either an exploration or exploitation decision. The perceived intensity of pain was analyzed by comparing the pain resulting from exploration and exploitation decisions using a linear model, while controlling for the physical intensity of the stimuli with a polynomial regression. The results indicate an increased perception of pain during exploratory decisions.

**Discussion/Conclusions**: These results suggest that pain perception appears to be modulated by its informational value depending on the exploration or exploitation context. Exploration, while crucial for learning, amplifies perceived pain, thereby impacting decision making.

## Correlations Among Endogenous Pain Modulation Mechanisms

Rossi Tomin^1^, Wanda Gao^2^, Omar Khalil^1^, Christine Sexton^1^, Kevin Murray^3^, James Khan^4^, Lauren Atlas^5, 6, 7^, David Finn^3, 8, 9^, Massieh Moayedi^1, 10, 11, 12^

^1^Centre for Multimodal Sensorimotor and Pain Research, Faculty of Dentistry, University of Toronto, Toronto, ON, Canada, ^2^University of Toronto, Faculty of Arts and Science, ^3^Pharmacology and Therapeutics, School of Medicine, University of Galway, Galway, Ireland, ^4^Department of Anesthesiology & Pain Medicine, University of Toronto, ^5^National Center for Complementary and Integrative Health, National Institutes of Health, Baltimore, Maryland, ^6^National Institute of Mental Health, National Institutes of Health, Baltimore, Maryland, ^7^National Institute on Drug Abuse, National Institutes of Health, Baltimore, Maryland, ^8^Galway Neuroscience Center, University of Galway, Galway, Ireland, ^9^Centre for Pain Research, University of Galway, Galway, Ireland, ^10^University of Toronto Center for the Study of Pain, University of Toronto, Toronto, ON, Canada, ^11^Department of Dentistry, Mount Sinai Hospital, Toronto, ON, Canada, ^12^Division of Clinical and Computational Neuroscience, Krembil Brain Institute, Toronto Western Hospital, University Health Network, Toronto, ON, Canada

**Introduction**: Descending pain modulatory systems can be engaged by various paradigms, including placebo analgesia (PA), offset analgesia (OA), and conditioned pain modulation (CPM), each initiated through different procedures. It remains unclear whether these paradigms share common neurobiological mechanisms or engage the same modulatory circuits. One approach to assess overlapping mechanisms is to determine if analgesia levels correlate within individuals across paradigms. This study aims to examine correlations in analgesia produced by PA, OA, and CPM and explore potential sex differences in these relationships.

**Methods**: Forty-nine healthy participants (24 female) underwent calibration (Atlas et al., 2022) and a PA paradigm with classical conditioning and placebo/control conditions (Eippert et al., 2009). They then completed an OA paradigm, with a noxious stimulus at 46°C (T1), raised to 48°C (T2), and returned to 46C° (King et al., 2014). Finally, they underwent a CPM paradigm, using a water bath as a conditioning stimulus and pressure pain thresholds as a test stimulus (O’Brien et al., 2018). Pain reduction ratings across paradigms were correlated using appropriate tests within sexes and at the group level (p < .05).

**Results**: No significant correlations were found across paradigms when analyzing the entire population. Additionally, no significant sex differences emerged in pain modulation patterns.

**Discussion/Conclusions**: The results indicate that PA, OA, and CPM likely operate through distinct, independent mechanisms, with no clear overlap in their modulatory pathways.

## Neuropathic Pain Driven Loss in the Appetitive Value of Affective Touch in Mice

Maham Zain^1^, Laura Bennett^1^, Quinn Pauli^1^, Shajenth Premachandran^2^, Juliet Arsenault^1^, Dylan Terstege^3^, Nevatha Kingsley^1^, Jonathon Epp^3^, Reza Sharif-Naeini^2^, Yves De Koninck^4^, Robert Bonin^1^

^1^University of Toronto, ^2^McGill, ^3^University of Calgary, ^4^University of Laval

**Introduction**: Sensory neurons expressing MrgprB4 detect gentle stroking in mice and their activation is known to be rewarding. Here we investigated whether activation of Channelrhodopsin (ChR2) expressing MrgprB4-lineage afferents is appetitive, whether this is altered in chronic pain and whether this is reflected in the downstream circuits recruited.

**Methods**: We used a light paired real-time place preference (RTPP) paradigm to assess the appetitive quality of blue light stimulation in MrgprB4-ChR2 mice. We also assessed whether this behavior was sensitive to neuropathic pain induced via nerve injury or acute pain induced via an intraplantar capsaicin injection. These mice also underwent a final stimulation after which the spinal cords and brains were dissected out for immunohistochemical analysis of c-Fos.

**Results**: The appetitive nature of optogenetic stimulation was maintained in the presence of capsaicin sensitization but was abated in states of neuropathic pain. This nerve injury induced loss in appetitive value was accompanied by alterations in the pattern of local activity within the spinal dorsal horn, altered recruitment of superficial dorsal horn neurons expressing neurochemical markers of projection neurons, and altered recruitment of the parabrachial nucleus that receives the bulk of the input from these projection neurons. These changes were also accompanied by changes in the higher order global encoding of these stimuli across various limbic and cortical structures.

**Discussion/Conclusions**: In conclusion, the appetitive value associated with affective touch is plastic and this plasticity is accompanied by altered encoding of this stimuli at both spinal and supraspinal levels with variations seen across both cortical and subcortical structures in the brain.

## Gender/Sex DifferencesAt the Intersection of Pain: How Sex and Gender Influence Frequent Medical Care Use

Marimée Godbout-Parent^1^, Nancy Julien^1^, Hermine Lore Nguena Nguefack^1^, Gabrielle Pagé^2^, Line Guenette^3^, Lucie Blais^2^, Anaïs Lacasse^1^

^1^Université du Québec en Abitibi-Témiscamingue, ^2^Université de Montréal, ^3^Université Laval

**Introduction**: Chronic pain (CP) disproportionately affects women, sexual and gender minorities, and other marginalized groups, highlighting the need for an intersectional approach to care. Sex, gender, and social factors influence healthcare utilization and must be considered to improve care trajectories. This study aimed to explore the associations between sex, gender, and frequent medical care use among individuals with CP.

**Methods**: The COPE Cohort, formed by linking a web-based survey with Quebec health administrative databases was used. Frequent medical care users were defined as the top 10% of our sample with the highest number of medical visits in the year following the completion of the questionnaire in 2019. Sex (male, female), gender identity (men, women, gender-diverse), and gender-stereotyped personality traits (masculine, feminine, androgynous, undifferentiated) were analyzed, and cluster analysis was used to create intersectional subgroups (incorporating sex, gender identity, gender-stereotyped personality traits, living in a remote region, country of birth, education, employment, and age).

**Results**: Out of 895 people, 10.6% (n = 95) were frequent medical care users with (top 10% cutoff: ≥13 visits/year). Frequent medical care users proportion varied across sex (male: 3.9% vs. female: 12.0%, p = .002), gender identity (men: 4.0% vs. women: 12.1% vs. gender-diverse: 0%, p = .002) but not between gender-stereotyped personality traits subgroups. Multivariable results showed that the intersectional cluster labeled ‘unemployed women’ (compared to men) had increased odds of being frequent medical care users (OR: 4.05, 95%CI: 1.48–11.10).

**Discussion/Conclusions**: Certain profiles are at higher risk of frequent medical care usage, highlighting the need for tailored care strategies.

**Understanding the Role of Dorsal Horn Genetics in Chronic Pain Between Males and Females**

Xindi Wang^1^, John Kramer^1^

^1^University of British Columbia

**Introduction**: Females report higher chronic pain prevalence than males, partially due to physiological differences. The dorsal horn (DH) modulates noxious input through excitatory and inhibitory neurons. In chronic pain, strengthened excitatory neurons and weakened inhibitory neurons may increase pain responses. Although rodent studies have revealed sex-biased spinal cord (SC) genetics, human data remain limited. This study explored sex-biased gene expression within human DH and identified pathways that may underlie chronic pain.

**Methods**: We integrated data from two sources: (1) an R Shiny app (Yadav et al.) providing single-nucleus RNA sequencing of lumbar (L3-L5) SC from 5 donors. (2) VoyAGEr, a bulk RNA-seq dataset of 159 cervical (C1) SC donors (Male:102, Female:57). Using source 1, 50 genes that are most localized to excitatory& inhibitory DH respectively were identified. The 100 genes were further assessed for sex bias using VoyAGEr (two-sample t-test, Benjamini-Hochberg<0.05). Additionally, all sex-biased C1 DH genes were identified using the R package LIMMA-voom (p < .05, log fold change >1.5). Pathway enrichment was analyzed via GO database.

**Results**: Out of the 100 DH-localized genes, 55 were upregulated in females (excitatory:25; inhibitory:30), while only one gene was upregulated in males (inhibitory). Cervical 1 DH genes showed significant sex bias, with 580 female-biased and 16 male-biased. Pathway analysis revealed enrichment in chemical synaptic transmission (CST) pathway in females.

**Discussion/Conclusions**: In females, both excitatory and inhibitory DH neurons were upregulated, further emphasizing sex differences in DH genetics. Findings highlight the need for sex-inclusive research in chronic pain treatment development.

## Imaging: Pain Imaging and NeuroimagingTemporal Trends in Spinal Imaging in Ontario (2002–2019) and Manitoba (2001–2011), Canada

Rayeh Al-Ghetaa^1^, Mostafa Alabousi^2^, John You^3^, Peter Emary^2^, John Riva^2^, John Dufton^4^, Yoan Kagoma^2^, Raja Rampersaud^1^, Michael Goytan^5^, Thomas Feasby^6^, Martin Reed^5^, Jason Busse^2^

^1^University of Toronto, ^2^McMaster, ^3^Trillium Health Partners, ^4^University Hospital of Northern British Columbia, ^5^University of Manitoba, ^6^University of Calgary

**Introduction**: Several studies have reported the overuse of spinal imaging. We assessed temporal trends in spine imaging in two Canadian provinces.

**Methods**: We explored the use of X-ray, computed tomography (CT), and magnetic resonance imaging (MRI) examinations of the cervical, thoracic, and lumbar spine regions among adults in Ontario (April 1, 2002, to March 31, 2019) and in Manitoba, Canada (April 1, 2001, to March 31, 2011) using linked Ontario Health Insurance Plan administrative databases and data from Manitoba Health. We calculated the age- and sex-adjusted rates of spinal X-ray, CT, and MRI examinations by dividing the number of imaging studies by the population of each province for each year and estimated the use of each imaging modality per 100,000 persons.

**Results**: The total cost of spine imaging in Ontario increased from $45.8 million in 2002/03 to $70.3 million in 2018/19 (a 54% increase), and in Manitoba from $2.2 million in 2001/02 to $5 million in 2010/11 (a 127% increase). In Ontario, rates of spine X-rays decreased by 12% and spine CT scans decreased by 28% over this time period, while in Manitoba, rates of spine X-rays and CT scans remained constant. Age- and sex-adjusted utilization of spinal MRI scans per 100,000 persons markedly increased over time in both Ontario (277%) and Manitoba (350%).

**Discussion/Conclusions**: Despite efforts to reduce the use of inappropriate spinal imaging, both Ontario and Manitoba have greatly increased utilization of spine MRI in the past two decades.

## Exploring the Role of Peak Alpha Frequency in Pain Sensitivity and Discomfort

Alyson Champagne^1, 2^, Mathieu Roy^3^, Michel-Pierre Coll^1, 2^

^1^Université Laval, ^2^Centre interdisciplinaire de recherche en réadaptation et intégration sociale, ^3^Université McGill

**Introduction**: Large individual variability in pain perception has led to the exploration of biomarkers that could predict pain and complement subjective evaluations. One promising candidate is the individual peak alpha frequency (PAF), an EEG-based biomarker potentially linked to pain sensitivity. While some studies suggest that resting-state PAF may predict pain sensibility and be pain-specific, findings remain inconsistent. This study aims to further validate PAF as a pain biomarker in a tonic pain paradigm and assess its specificity to nonpainful aversive experiences.

**Methods**: EEG data were collected from 77 healthy individuals during resting-state, tonic thermal pain (with/without intensity ratings), and an unpleasant auditory stimulus (with/without unpleasantness ratings). Power spectral density was computed using a Fourier transform on raw EEG signals. PAF was estimated using the center of gravity (CoG) method within the alpha band (8–13 Hz) over sensorimotor cortices during rest and pain/aversive tasks. Correlations between PAF and pain/discomfort mean ratings were computed.

**Results**: Preliminary results show that PAF was highly stable across tasks (ICC = 0.93, p < .01) and did not significantly differ between conditions (F(4, 72) = 1.11, p = .35). A negative correlation was found between resting-state PAF and tonic pain intensity (ρ = – 0.20, BF10 = 3.80), but not with auditory unpleasantness (ρ = −0.02, BF10 = 1.01). No associations were found with psychological factors.

**Discussion/Conclusions**: These results suggest that PAF is negatively associated with pain intensity but not with auditory unpleasantness, supporting its potential as a biomarker of individual pain sensitivity.

## Alpha Oscillations and Conditioned Pain Modulation Predict Pain Relief from Spinal Cord Stimulation

Rima El-Sayed^1, 2^, Vaidhehi Sanmugananthan^1, 2^, Ariana Besik^2^, Natalie Osborne^1, 2^, Emily Mills^2^, Camille Fauchon^2^, Anuj Bhatia^2, 3^, Benjamin Dunkley^4, 5, 6^, Karen Davis^1, 2, 7^

^1^Institute of Medical Science, University of Toronto, Toronto, ON, Canada, ^2^Division of Brain, Imaging, and Behavior, Krembil Brain Institute, University Health Network, Toronto, ON, Canada, ^3^Department of Anesthesia and Pain Medicine, Toronto Western Hospital, and University of Toronto, Toronto, ON, Canada, ^4^Diagnostic Imaging, The Hospital for Sick Children, Toronto, ON, Canada, ^5^Neurosciences & Mental Health Program, The Hospital for Sick Children Research Institute, Toronto, ON, Canada, ^6^Department of Medical Imaging, University of Toronto, Toronto, ON, Canada, ^7^Department of Surgery, University of Toronto, Toronto, ON, Canada

**Introduction**: Neuropathic pain (NP) is a severe form of chronic pain associated with significantly reduced quality of life. Spinal cord stimulation (SCS) can provide significant pain relief but is ineffective in half of those treated, highlighting the importance of predictive markers of treatment outcome. Given previous studies showing abnormal alpha oscillations and altered conditioned pain modulation (CPM) in chronic pain, our aim was to determine whether alpha oscillations and CPM can predict pain relief from SCS.

**Methods**: Patients with NP were evaluated before a 12-day SCS trial using pain self-reports, CPM, a magnetic resonance imaging (MRI) scan and a 5-minute resting state magnetoencephalography (MEG) scan to evaluate alpha oscillations (8–13 Hz) in the dynamic pain connectome. Those with ≥30% pain reduction in a post SCS evaluation were considered treatment responders. Age- and sex-matched healthy controls (HC) also underwent CPM testing and MEG.

**Results**: The analyses included 40 patients with NP (22 F, 18 M) and 29 HCs (17 F, 12 M). Approximately half of the patients were nonresponders and pre-SCS testing of CPM revealed this group (but not the responders) to exhibit significantly diminished inhibitory CPM compared to HCs. Pre-SCS MEG revealed that lower peak alpha power throughout the ascending nociceptive pathway and salience network was correlated with greater pain relief.

**Discussion/Conclusions**: These data highlight abnormalities in alpha oscillations in the ascending nociceptive pathway and the health of the descending inhibitory control system in patients with NP that could provide predictive value when considering personalized pain management with SCS treatment.

## Increasing the Peak Alpha Frequency (PAF) Using Auditory Stimuli

Amélie Grandjean^1^, Massieh Moayedi^2^, David Seminowicz^3^, Ali Mazaheri^1^

^1^University of Birmingham, ^2^University of Toronto, ^3^University of Western Ontario

**Introduction**: The Peak Alpha Frequency (PAF) is the frequency with the greatest power within the alpha band (8–14 Hz), measured using EEG. PAF is a stable and heritable trait linked to pain sensitivity. Slower PAF is associated with heightened experimental acute pain sensitivity and chronic pain conditions. However, it remains unknown whether PAF can be modified by external stimuli and whether this would impact pain sensitivity. Here, we address the first question by determining whether auditory stimuli in the form of broadband noise bursts can increase PAF and assessing the duration of the increase.

**Methods**: Eight participants consented to procedures approved by the local ethics board. Participants had their PAF measured while resting with eyes closed for 3 minutes. Next, they passively listened to either 8 Hz or 12 Hz noise bursts, followed by a second resting-state recording. They completed a standardized cognitive task before a third resting-state recording. Three hours later, participants returned to repeat auditory stimulation.

**Results**: We show that both 8 Hz and 12 Hz sounds increased PAF at parietal electrodes compared to baseline in 7 of 8 participants. The increase was more pronounced and appeared to compound with repeated auditory stimulation in individuals with a slower baseline PAF. The increase was not sustained after the 3-hour interval.

**Discussion/Conclusions**: These pilot findings have led to developing of a follow-up study aimed at optimizing the auditory stimulus’ impact, measuring its duration, and exploring potential mechanisms using time-frequency analyses of EEG recordings taken while listening to the sounds.

## Investigating the Effect of Heat Stimulus Parameters on Pain-Related fMRI BOLD Activity

Somayeh Mashatan^1^, Sarah Margerison^2^, Brent Stewart^2^, Michael Keaser^2^, David Seminowicz^1^

^1^Medical Biophysics, The University of Western Ontario, ^2^Department of Neural and Pain Sciences, University of Maryland School of Dentistry, Baltimore, MD, USA

**Introduction**: Although most pain neuroimaging studies have examined the effects of stimulus intensity on pain-related brain activity, the research on stimulus duration is limited. This study employs simultaneous EEG-fMRI collected from healthy participants to determine the effects of thermal stimulus intensity and duration on brain activity. Here, we focus only on the fMRI findings.

**Methods**: Fifty healthy participants underwent a heat pain paradigm with four intensities (warm, slight, moderate, intense) and four durations (4, 8, 21, 36 seconds) while EEG and fMRI data were recorded simultaneously. First-level fMRI analysis created contrasts for 16 conditions (4 duration x 4 intensity), followed by group-level analysis using a flexible factorial design to assess the main effects of intensity and duration.

**Results**: A significant main effect of intensity was observed in the right and left anterior insula, left thalamus, and left dorsolateral prefrontal cortex (DLPFC). The main effect of duration was observed in the left DLPFC, right and left anterior prefrontal cortex, and right and left caudate.

**Discussion/Conclusions**: Distinct brain regions are associated with processing heat pain intensity and duration, highlighting the unique contributions of these factors to pain perception.

## Exploratory Analysis of White Matter Microstructure and Placebo and Nocebo Effects in Youth

Si Chen Pan^1^, Lucy Stuckey^2^, Adam Kirton^1^, Helen Carlson^1^, Melanie Noel^1^, Serena Orr^1^, Nivez Rasic^1^, Frank MacMaster^3^, Kathryn Birnie^1^, Lindsay Craddock^1^, Jillian Miller^1^

^1^University of Calgary, ^2^University of Alberta, ^3^Dalhousie University

**Introduction**: Harnessing placebo, and/or reducing nocebo (negative expectations of a treatment) effects has significant implications for medical practice. In adults, greater microstructure of the right dorsolateral prefrontal cortex and left rostral anterior cingulate cortex is associated with stronger placebo effects. However, this may not be applicable to adolescents due to cortical immaturity.

**Methods**: Eight healthy youth (14–18 years) underwent 3T MRI scanning and a validated pain-related placebo/nocebo protocol. Participants’ heat pain thresholds (low, moderate, high) were measured. One inert cream dyed three different colors was applied to each row of a 3 × 3 grid drawn on the forearm. Participants were told that the pink cream increased heat sensitivity, the blue cream decreased sensitivity, and the yellow cream had no effect. The first grid column served as a primer, and moderate heat pain was applied to the remaining squares of the grid. Pain intensity was self-reported using a numerical rating scale (0 “no pain” to 10 “worst pain imaginable”). Mean average diffusivity was obtained from diffusion tensor images.

**Results**: Youth with greater white matter microstructure of the left cingulum were more responsive to nocebo hyperalgesia (b = −0.772, p = .013, R^2^ = 0.770) and appeared less responsive to placebo analgesia (b = 0.776, p = .085, R^2^ = 0.275).

**Discussion/Conclusions**: Exploratory analyses revealed that greater white matter microstructure of the left cingulum was associated with placebo/nocebo effects. Further investigation into these relationships is required, so that we can better understand the neurobiology of placebo/nocebo in youth.

## Theta-Gamma Phase-Amplitude Coupling in the Dynamic Pain Connectome in Healthy Individuals and Abnormalities in People with Chronic Pain

Ariana Seyed Makki^1, 2^, Kasey Hemington^1, 2^, Anton Rogachov^1, 2^, Joshua Cheng^1, 2^, Rachael Bosma^1, 2^, Natalie Osborne^1, 2^, Rima El-Sayed^1, 2^, Benjamin Dunkley^2, 3, 4, 5, 6^, Robert Inman^2,7^, Karen Davis^1, 2, 8^

^1^Division of Brain, Imaging and Behavior, Krembil Brain Institute, Krembil Research Institute, University Health Network, Toronto, Ontario, Canada, ^2^Institute of Medical Science, University of Toronto, Toronto, Ontario, Canada., ^3^Department of Diagnostic Imaging, Hospital for Sick Children, Toronto, Ontario, Canada., ^4^Neurosciences & Mental Health, SickKids Research Institute, Toronto, Ontario, Canada., ^5^Department of Medical Imaging, University of Toronto, Toronto, Ontario, Canada., ^6^Department of Psychology, University of Nottingham, Nottingham, UK., ^7^Department of Immunology, University of Toronto, Toronto, Ontario, Canada, ^8^Department of Surgery, University of Toronto, Toronto, Ontario, Canada

**Introduction**: How does the brain process nociceptive stimuli and contribute to chronic pain? Theta-gamma phase-amplitude coupling (PAC) could serve to gate nociceptive processing and modulation at different points of interaction within the dynamic pain connectome (DPC). This study investigated whether there is normally PAC of intrinsic activity within the DPC and whether it is disrupted in people with chronic pain.

**Methods**: Resting-state magnetoencephalography was used to measure theta-gamma PAC in 38 healthy individuals (20 M, 18 F) and 37 individuals with chronic pain associated with ankylosing spondylitis (20 M, 17 F). The magnitude and incidence of PAC was assessed in nodes of the ascending nociceptive and descending antinociceptive pathways, default mode and salience networks. We also examined whether there were associations between PAC and each patient’s chronic pain intensity, disease severity, and functional limitations.

**Results**: Most or all individuals in the healthy and chronic pain groups exhibited PAC in all DPC regions tested, except the subgenual anterior cingulate cortex of the descending antinociceptive pathway (37% and 45%, respectively). Individuals with chronic pain exhibited PAC abnormalities in the right midcingulate cortex of the salience network, which also had moderate associations with disease severity and functional limitations. Compared to males, females with chronic pain showed more widespread PAC abnormalities across the DPC.

**Discussion/Conclusions**: This study provides novel data to implicate theta-gamma PAC as a means to shape the outcome of noxious input to the brain. These findings also point to PAC failures as a possible abnormality that could contribute to chronic pain.

## Revealing Trigeminal System Microstructural Alterations, Hyperexcitability, and Inflammation in Episodic Migraine: A Combined 7 T DTI/fMRI and PET Study

Sarasa Tohyama^1^, Michael Datko^1^, Ludovica Brusaferri^2^, Lillian Kinder^1^, Jack Schnieders^2^, Mackenzie Hyman^2^, Alison Goldstein^1^, Melaina Gilbert^1^, Hope Housman^2^, Vi Le^2^, Kassandra Round^1^, Frances Marin^3^, Megan Heffernan^2^, Ronald Garcia^2^, Randy Gollub^2^, Robert Edwards^4^, Bruce Rosen^2^, Nouchine Hadjikhani^2^, Hsinlin Cheng^5^, Zev Schuman- Olivier^6^, Marco Loggia^2^, Vitaly Napadow^1^

^1^Department of Physical Medicine and Rehabilitation, Spaulding Rehabilitation Hospital, Harvard Medical School, ^2^Athinoula A. Martinos Center for Biomedical Imaging, Department of Radiology, Massachusetts General Hospital, Harvard Medical School, ^3^Center for Mindfulness and Compassion, Department of Psychiatry, Cambridge Health Alliance, Harvard Medical School, ^4^Department of Anesthesiology, Perioperative and Pain Medicine, Brigham and Women’s Hospital, Harvard Medical School, ^5^Department of Neurology, Massachusetts General Hospital, Harvard Medical School, ^6^Center for Mindfulness and Compassion, Cambridge Health Alliance

**Introduction**: Migraine, a highly prevalent and disabling chronic pain condition, involves sensitization of the trigeminal system. The trigeminal system has been historically difficult to image using neuroimaging techniques because of its complex, small-scale structure. No studies to date have examined this system using an advanced multimodal neuroimaging approach.

**Methods**: We employed 7 T diffusion tensor imaging (DTI)/fMRI and PET, to investigate the structure-function associations of the trigeminal system in migraine patients. 60 patients and 20 healthy controls underwent 7 T DTI/fMRI and PET. The DTI measure of fractional anisotropy (FA) and the PET signal were extracted from both trigeminal nerves. Innocuous forehead stimulation was applied to investigate brainstem fMRI response to trigeminal sensory afference. FA was compared between patients and controls. FA was also correlated with the PET signal and brainstem fMRI response in the migraine cohort.

**Results**: Migraine patients showed decreased FA in both trigeminal nerves compared to controls. Patients also demonstrated fMRI activation in the right spinal trigeminal nucleus (SpV). A negative association was observed between trigeminal FA and the PET signal. A positive association was observed between trigeminal FA and SpV fMRI activation.

**Discussion/Conclusions**: In conclusion, migraine patients demonstrate altered trigeminal nerve white matter microstructure and SpV fMRI response to trigeminal sensory afference. Furthermore, the greater the microstructural alteration of the nerve, the greater the neuroinflammation in the same anatomical region and less functional communication present in its associated brainstem nuclei. Trigeminal system remodeling may be an important aspect of the dynamics underlying migraine pathophysiology.

## Brain Microstructure and Pain Interference Following Idiopathic Scoliosis Repair in Youth

Maddison Tory^1^, Jillian Miller^1^, Catherine Lebel^1^, Melanie Noel^1^

^1^University of Calgary

**Introduction**: Approximately 20% of youth undergoing major surgeries grapple with chronic post-surgical pain (CPSP). Recent data indicates that exaggerated memories of pain surrounding the surgical experience predict higher levels of postsurgical acute and chronic pain. Although psychological influences of CPSP have been identified, the biological mechanisms are less understood. This study examined brain microstructural changes and postsurgical pain in youth who underwent surgical repair for idiopathic scoliosis.

**Methods**: In all, 50 youth aged 10–18 years requiring surgical correction for idiopathic scoliosis, were recruited. Youth underwent diffusion tensor imaging (DTI) and filled out pain interference questionnaires approximately 1 week before their surgery. Moreover, they will repeat the same DTI and questionnaire at 4-months postsurgery. Fractional anisotropy (FA) values, a DTI measure of white matter microstructure, were obtained from the fornix, cingulum and uncinate fasciculus. Paired t-test compared pain interference and FA values pre- to postsurgery. Linear mixed models and linear regression were used to examine whether greater white matter microstructure was associated with greater pain interference 4-months postsurgery.

**Results**: Pain interference significantly decreased between 1-week presurgery to 4-months postsurgery (mean 66 to 50, P < .001), however no significant FA changes were observed. Lower fornixFA at baseline was associated with less pain interference at 4-months postsurgery (b = 0.43, P = .03, R2 = 0.59).

**Discussion/ Conclusions**: For those with greater fornix microstructure at baseline, pain interference was higher at 4-months postsurgery. The fornix is involved in memory formation and consolidation. Greater connectivity of the fornix may lead to greater reconsolidation of pain memories.

## Exploration of Brain Efficiency and Placebo and Nocebo Responses in Youth

Yi An Wang^1^, Adam Kirton^1^, Helen Carlson^1^, Melanie Noel^1^, Serena Orr^1^, Nivez Rasic^1^, Frank MacMaster^2^, Kathryn Birnie^1^, Lindsay Craddock^1^, Jillian Miller^1^

^1^University of Calgary, ^2^Dalhousie University

**Introduction**: Expectations surrounding treatments can significantly enhance (nocebo hyperalgesia) or decrease (placebo analgesia) response to pain. Whole-brain global efficiency measures how efficiently information is transferred between nodes across the brain and local efficiency measures how efficiently information is transferred with the immediate neighbors of a node. Little is known about how whole-brain efficiency influences placebo and nocebo responses.

**Methods**: 10 healthy youth (ages 14–18) were included in this exploratory analysis. Using a thermal device, participants’ thresholds for low, moderate, and high heat pain were determined. A 3 × 3 grid was drawn onto their forearm. One inert cream dyed pink, blue, and yellow was applied one color per row. Participants were made to believe that the pink cream induced hyperalgesia, the blue induced analgesia, and the yellow had no effect. Moderate heat pain was then applied to the grid and pain intensity was self-reported. Youth subsequently underwent resting-state functional MRI scanning from which graph theory metrics were obtained. Linear regressions were used to explore the relationships between global and local brain efficiency and pain intensity, controlling for age and gender.

**Results**: Lower whole-brain global (R^2^ = .782, F(3,6) = 7.182, p = .02) and local efficiency (R^2^ = .815, F(3,6) = 8.790, p = .03) were significantly associated with greater nocebo responses after controlling for age and gender. In contrast, neither global nor local efficiency were associated with placebo responses in youth.

**Discussion/Conclusions**: Exploratory analyses revealed that youth with lower whole- brain global and local efficiency may be more susceptible to nocebo effects.

## Neural Mechanisms of Adaptive Control in the Anterior Mid-Cingulate Cortex Across Negative Emotion, Cognition, and Somatic Pain

Yuan Yao^1^, Chun Yin Liu^1^, Andrew Furman^2^, Michael Keaser^2^, Alexander Shackman^3^, David Seminowicz^1, 2^

^1^University of Western Ontario, ^2^University of Maryland, Baltimore, ^3^University of Maryland, College Park

**Introduction**: Theoretical frameworks suggest that adaptive control is a key mechanism underlying the role of the anterior mid-cingulate cortex (aMCC; also known as the dorsal anterior cingulate cortex, dACC) across psychological domains, including emotion, cognition, and pain. However, empirical evidence remains limited. Adaptive control refers to the process by which individuals evaluate choices and voluntarily act to best respond when faced with current, or foreseeable physical harm or abstract errors.

**Methods**: This study analyzed task-based connectivity patterns, defined as the statistical dependencies between brain structures that are anatomically apart, of the aMCC using data from 23 healthy adults (11 F, M = 20.8 yrs, SD = 3.79) who underwent functional magnetic resonance imaging (fMRI) while performing tasks probing negative affect (threat-of-shock task), cognitive control (multisource interference task), and somatic pain (thermal stimulation).

**Results**: Results from univariate seed-based connectivity analyses with both classical and Bayesian inference, along with multivariate component analysis, corroborated adaptive control as the candidate underlying process for aMCC. Furthermore, cross-fitted three- layer dynamic causal modeling (DCM) revealed a forward-flow of information from domain-specific inputs (Layer 1) to the aMCC and higher-order processing regions (Layer 2) across tasks.

**Discussion/Conclusions**: These findings support the role of adaptive control as a unifying process governing aMCC function and highlight the need for future research to further validate this framework.

## Pain in Specific PopulationsLiving with Phantom Limb Pain: Insights into the Impact of Pain on Limb Loss

Andrea Aternali^1^, Nicole Dimitrova^1^, Heather Lumsden-Ruegg^1^, Sander L. Hitzig^2^, Amanda L. Mayo^3^, Joel Katz^1^

^1^York University, ^2^St. John’s Rehab Research Program, ^3^Sunnybrook Health Sciences Center

**Introduction**: Phantom limb pain (PLP) can be both physically painful and emotionally distressing. For many, PLP persists long after amputation, affecting daily life, emotional well-being, and relationships. Our understanding of the subjective experience of PLP remains limited, as much of the existing literature focuses on clinical aspects rather than lived experience.

**Methods**: To address this gap, semistructured interviews were conducted with individuals who had undergone unilateral limb amputation to better understand the impact of PLP. The interviews were transcribed, and an inductive thematic and content analysis was performed.

**Results**: Ten individuals (9 male, Mage = 50.4 ± 12.3 years) participated in the study. All participants endorsed PLP over the past week, where the average pain intensity was 5.3 ± 3.3 on a 0–10 numerical rating scale. From the interviews, three key themes were identified: the frustration of being unable to manage their pain, challenges associated with coping with their new reality and no longer being able to engage in the activities they used to enjoy. Moreover, the participants also reported strategies that help them move toward acceptance; letting go of what life used to be like, acknowledging that they cannot get their limb back, and sharing their experiences with other individuals with limb loss.

**Discussion/Conclusions**: The findings highlight the profound impact of PLP on individuals’ lives and underscore the importance of addressing both physical and emotional dimensions in pain management. This research contributes to a deeper understanding of PLP and suggests that holistic, peer-supported approaches may help individuals adapt to life after amputation.

## International Environmental Scan for Pediatric Complex Regional Pain Syndrome Treatment Protocols: Preliminary Results

Krista Baerg^1^, Amanjot Kaur^1^, Nicole Cooke^2^, Susan Tupper^1^, Allen Finley^3^

^1^University of Saskatchewan, ^2^Patient Partner, ^3^Dalhousie University

**Introduction**: There is a lack of consensus on best practice for treatment of pediatric Complex Regional Pain Syndrome (pCRPS). We sought to summarize unpublished pCRPS clinical treatment protocols.

**Methods**: An anonymous survey was distributed to members of the International Pediatric Pain list-serve (approximately 1200 members from over 40 countries) who engage in informal discussion about any topic related to pediatric pain. The 9-question survey collected country of residence, practice setting, use of a treatment protocol, policy, and/or clinical standard, type (e.g. triage, medication/physical therapy/psychology protocol), how it is used (standardized/individualized) to manage pCRPS and two open- ended questions describing protocol use and development; participants had the option to upload up to 5 documents. The survey was exempt from Ethics Board review.

**Results**: 30 participants (USA, n = 9, 30%; Canada, n = 7, 23.33%; other, n = 14, 46.7%) practicing in outpatient/ambulatory (n = 26, 86.7%), inpatient (n = 15, 50%), intensive rehabilitation (n = 5, 16.7%) and other (n = 2, 6.7%) settings (multiple responses allowed) participated. Most participants (n = 22, 73.3%) reported their clinic or organization did not have a pCRPS protocol, policy or clinical standard. When available, none were applied in all cases (n = 8, 26.7%); care was individualized either using standard protocols (n = 4) or with standard protocols available (n = 4). Physical therapy (n = 8), psychology (n = 8), triage (n = 6), medication/infusion (n = 6) and other (n = 1) protocols were reported (multiple responses allowed; all pediatric). Three documents were uploaded.

**Discussion/Conclusions**: Our findings suggest the need for clinical guidance with consensus guidelines to improve standardization and quality of care in the treatment of pCRPS.

## Public Opinion and the Undertreatment of Pain in Dementia: A Social Media Study

Louise I.R. Castillo^1^, Laney Yarycky^1^, Thomas Hadjistavropoulos^1^

^1^University of Regina

**Introduction**: Pain in people with dementia is frequently underassessed and undertreated related to the sufferers’ limited ability to communicate. Effective pain assessment strategies are available but not widely used. Public opinion is often influential in changing policies including policies to overcome the underassessment of pain in long- term care (LTC). To evaluate public opinion, we examined social media discussions.

**Methods**: Keyhole social media monitoring was used to extract Twitter posts. Searches for tweets containing the hashtags #pain #dementia AND/OR key words “pain dementia” were conducted for between 1/10/18 to 28/2/19. Identified tweets were subjected to content analysis.

**Results**: A total of 1,992 tweets were identified. Over 30% focused on pain assessment/management in dementia but these tweets mainly originated from scientists/clinicians. There was little discussion from the general public who frequently used the word “pain” as a metaphor for grief/suffering (21.39%). Most of the remaining tweets were either unrelated to pain in dementia or it was unclear whether the discussion of pain was related to dementia.

**Discussion/Conclusions**: Our results suggest that, while scientists are disseminating information about the underassessment of pain in dementia, there is little evidence that the general public is pre-occupied with this issue. In contrast, previous research has demonstrated that the public is well aware of other challenges (e.g., infection control) faced by older adults in LTC. There is a need for greater dissemination efforts to inform the public of the widespread problem of underassessed pain in dementia and of available solutions.

## Exploration de l’effet de la thérapie ostéopathique viscérale (TMOv) sur la douleur et l’anxiété des patients atteints de lombalgies chroniques non-spécifiques: Une étude pilote.

Anais Beaupré^1^, Émilie Paul-Savoie^1^, Christian Rochefort^1, 2, 3^

^1^Université de Sherbrooke, ^2^Centre de recherche de l’Université de Sherbrooke, ^3^Centre de recherche de l’Hôpital Charles Le Moyne

**Introduction**: Les lombalgies sont des troubles musculosquelettiques présentant un problème de santé publique en croissance. La prévalence à vie de souffrir de lombalgies chroniques

non-spécifiques (LCNS) dans la population générale peut atteindre 23%. Les traitements usuels ne sont pas totalement curatifs. L’ostéopathie viscérale, objet de débats et controverses, sont souvent pratiquées au Québec pour cette condition. Cette étude explore la faisabilité de la prise en charge ostéopathique viscérales (TMOv) pour les LCNS.

**Méthodologie**: Une étude pilote de faisabilité a été réalisée. 20 individus de plus de 18 ans vivant avec des LCNS ont été recrutés suite à une évaluation ostéopathique. Ces participants ont été randomisés en deux groupes recevant ensuite à deux reprises des TMOv ou un traitement simulé. L’intensité de la douleur lombaire, l’anxiété situationnelle, la capacité fonctionnelle, la satisfaction du soin et la perception du changement ont été évalués avant les interventions et après chacune des interventions et lors d’un appel de suivi trois semaines après la dernière intervention.

**Résultats**: Aucune différence significative entre les groupes pour les composantes motivo-affectives de la douleur et l’anxiété situationnelle (∂ = 0,497, ∂ = 0,907) et l’intensité de la douleur (p = 0,058, β − 0,19) n’a été observé. Une différence significative a été observée pour la capacité fonctionnelle (p = 0,017, β − 0,34), la perception de changement (p = 0,035, β = 0,48) et la satisfaction du traitement (2,97 ± 0,69). Le taux d’acceptabilité du traitement était de 73,7% [51,2%-88,19%], le taux de recrutement de 33,3% [22.7% 45.9%] et le taux de rétention de 95% [76,39%-99,11%]. L’effet indésirable le plus documenté a été la fatigue (30%).

**Discussion/Conclusions**: Les TMOv standardisées semble montrer une amélioration de la capacité fonctionnelle et sont bien acceptées comme option de soin pour les LCNS, avec peu d’effets secondaires rapportés.

## Altered Metabolite Profile in Individuals with Complex Regional Pain Syndrome (CRPS)

Maayan Ben Sasson^1, 2^, Vibhu Kumar^1, 3^, Emmanuel Gonzalez^3, 4^, Tali Sahar^1^, May Haddad^2^, Nicholas Brereton^5^, Sabrina Mitrovic^1^, Maria Verner^1^, Sylvie Toupin^1^, Yoram Shir^1^, Amir Minerbi^1, 2^

^1^Alan Edwards Pain Management Unit, McGill University Health Center, Canada, ^2^Institute for Pain Medicine, Rambam Health Campus, Israel, ^3^McGill University, Canada, ^4^Canadian Center for Computational Genomics, Canada, ^5^Université de Montréal, Canada

**Introduction**: CRPS causes severe limb pain with autonomic and inflammatory symptoms of unclear etiology. Gut-bacterial metabolites may influence chronic pain. This study investigates the gut metabolome in CRPS patients to explore its potential role in the condition.

**Methods**: Plasma (62 CRPS,40 controls) and fecal (40 CRPS,39 controls) samples (age-gender- and ethnicity matched) were analyzed using automated sample preparation and UPLC-MS/MS profiling. Data were processed via the Metabolon LIMS system.

**Results**: Our findings in CRPS patients reveal:

1. Metabolite Disruptions: Altered fecal and plasma metabolites, including short-chain fatty acids and benzoates, align with gut microbiome changes.
2. Tryptophan & Serotonin Reduction: Lower plasma levels may enhance pain sensitivity and are also linked to anxiety and depression which are common in chronic pain.
3. Kynurenine Pathway Imbalance: Elevated KYN/TRP ratio and reduced kynurenate suggest inflammation, neurotoxicity, and central sensitization.
4. IDO-1 Activity: Increased IDO-1 and a 10-fold rise in fecal xanthurenate highlight inflammation, oxidative stress, and neurotoxicity.
5. Gut Dysbiosis: High fecal skatole levels (3.15-fold rise) suggest DNA damage through oxidative stress and inflammation. Elevated plasma fibrinopeptides indicate poor microcirculation as an inflammation process.
6. Phospholipid Metabolism Alterations: Disruptions in energy metabolism and antioxidant mechanisms, with reduced plasmalogens and sphingolipids, may contribute to heightened pain sensitivity.

**Discussion/Conclusions**: Our findings highlight disruptions in metabolism, inflammation, and gut-brain interactions in CRPS, including altered tryptophan and lipid pathways, oxidative stress, and gut dysbiosis. These mechanisms may contribute to heightened pain sensitivity and chronic inflammation. Whether these changes are a cause or effect remains unclear.

## The Pain Experiences of Adolescent Survivors of Childhood Cancer: A Qualitative Exploration

Jada Benedictson^1^, Alex Pizzo^1^, Giulia Mesaroli^2^, Paul C. Nathan^2^, Jennifer N. Stinson^2^, Nicole M. Alberts^1^

^1^Concordia University, ^2^The Hospital for Sick Children Research Institute

**Introduction**: Clinically significant pain during and after childhood cancer treatment is common and distressing. Our recent work has shown that this pain persists into adulthood, with 41% of adult survivors experiencing chronic pain decades after their diagnosis. However, little is known about the pain experiences of adolescent survivors (ages 13–18).

**Methods**: Adolescent survivors of childhood cancer (N = 142, consent rate = 91.6%) were recruited from the AfterCare Clinic at The Hospital for Sick Children. Participants who endorsed pain were directed to an open-ended survey question: “Please describe the pain you are experiencing. If possible, give an example or explain how pain impacts (or doesn’t impact) your day-to-day life.” Responses were analyzed using descriptive content analysis.

**Results**: Of the participants, 23% (N = 33, mean age [SD] 15.6 [1.4], 73% female, mean time off treatment [SD] = 8.4 years [4.3]) reported having pain in the past month. Regarding pain experiences, 3 major themes were identified: 1) factors exacerbating pain (physical and psychological) and its impact on daily activities; 2) sources of pain including cancer-specific (i.e., location and treatment) and non-cancer-specific; and 3) characteristics and quality descriptors of pain experiences (e.g., “sharp” and “tingling”).

**Discussion/Conclusions**: Improved characterization of adolescent survivors’ pain is needed, as this carries implications for pain assessment and treatment. Findings show that almost a quarter of adolescent survivors are experiencing pain and provide insight into how they experience, describe, and are impacted by this pain. Future research with larger samples is needed to further characterize adolescent survivors’ pain, including chronic pain, and associated outcomes.

## Specifications to Inform Best Practice Recommendations and a Decision Aid Framework To Support Canadian Armed Forces Veterans Living with Pain to Optimize Their Physical Activity and Exercise Engagement

Robin Campbell Bromhead^1^, Joy C. MacDermid^1^, Nicholas Held^2^, Jordan Miller^3^, Heidi Cramm^3^, Joline Attalla^1^, Shannon Killip^1^

^1^Western University, ^2^McMaster University, ^3^Queens University

**Introduction**: Canadian Armed Forces (CAF) Veterans are twice as likely to experience chronic pain compared to the Canadian population. Evidence suggests physical activity/exercise can help improve health for people with pain. Given the higher prevalence of pain and unique military factors, Veterans may have specific needs that are different from the general public. This study aimed to understand the unique barriers, facilitators, and preferences for physical activity/exercise for CAF Veterans living with chronic pain to provide specifications to inform best practice recommendations and a decision-aid support framework.

**Methods**: A qualitative interpretive description was used, and a purposeful sample of CAF Veterans was recruited. Inclusion criteria: 18+ years; can read, understand, and speak English or French; and CAF Veterans with chronic pain (> 3 months). Twenty-one online interviews were completed. Interviews were audio-recorded, transcribed, and coded using thematic analysis.

**Results**: The results were categorized into barriers, facilitators, needs, and preferences. Barriers were mental health issues, pain severity and fluctuations, fear of pain, navigating resources, and self-stigma. Facilitators were social support, existing knowledge of exercise benefits, exercise modifications, and pain management. Preferences and needs included tailoring and individualized programs, exercise professionals’ understanding of military culture and service-related pain, and activities that reduced pain.

**Discussion/Conclusions**: This study’s findings provide new knowledge on the barriers, facilitators, needs, and preferences for physical activity/exercise among Canadian Veterans with chronic pain. The results will help inform best practice recommendations and a decision-aid framework for CAF Veterans to choose physical activity/exercise that aligns with their specific needs.

## Fibromyalgia IgG Antibodies Have Elevated Binding to Gut Bacteria

Hannah Cho^1, 2^, Ezrah Issac Roy^1, 2^, Alexander Rosenström^3^, Karolina af Ekenstam^4, 5^, Monika Löfgren^4, 5^, Emilie Linderoth^6^, Katalin Sandor^6^, Carolina B. Meloto^1, 2^, Arkady Khoutorsky^1, 2, 7^, Luda Diatchenko^1, 2, 7^, Camilla Svensson^6^, Eva Kosek^3, 8^, Emerson Krock^1, 2^

^1^Faculty of Dental Medicine and Oral Health Sciences, McGill University, ^2^Alan Edwards Center for Research on Pain, ^3^Department of Surgical Sciences, Clinical Pain Research, Uppsala University, Uppsala, Sweden, ^4^Department of Clinical Sciences, Danderyd Hospital, Karolinska Institutet, Stockholm, Sweden, ^5^Department of Rehabilitation Medicine, Danderyd University Hospital, Stockholm, Sweden, ^6^Department of Physiology and Pharmacology, Karolinska Institutet, Stockholm, Sweden, ^7^Department of Anesthesia, McGill University, ^8^Department of Clinical Neuroscience, Karolinska Institutet, Stockholm, Sweden

**Introduction**: We previously identified a role for autoantibodies in fibromyalgia (FM) pain, but why FM autoantibodies develop remains unclear. The gut microbiome of FM patients is altered, and the abundance of specific bacteria is linked to more severe pain. Our hypothesis is that FM autoantibodies develop through cross-reactivity with gut bacteria. Bacteria are often coated by IgA antibodies, but IgG antibody coated gut bacteria are uncommon. Similarly, circulating IgG in the blood that bind gut bacteria are rare. Increased IgG against gut bacteria is indicative of an abnormal immune response that could give rise to cross-reactive autoantibodies. Here, we investigate changes in IgG coating of gut bacteria and circulating IgG binding to gut bacteria.

**Methods**: Circulating IgG was purified from FM and control serum samples using Protein G columns. IgG coating of stool-isolated gut bacteria and changes in circulating IgG binding to gut bacteria were analyzed by flow cytometry.

**Results**: The percent of gut bacteria coated by IgG was greater in FM patients compared to controls. Similarly, circulating FM IgG bound more FM gut bacteria compared to control IgG and bacteria. Additionally, a moderately-strong correlation was observed between the levels of IgG coated bacteria and Visual Analogue Scale pain scores in FM patients.

**Discussion/Conclusions**: Our results suggest that FM patients have abnormal local and systemic immune responses to FM gut bacteria, which could lead to the generation of cross-reactive autoantibodies. Future studies will identify bacterial taxa bound by IgG in FM patients and controls.

## Calling the Land to Speak to Chronic Pain: Listening to Sexual Assault Survivors

Patricia May Derbyshire^1^, Jon Corbett^1^, Diane Gromala^1^, Sara Khalilipicha^1^, Armin Froozanfar^1^, Efe Erhan^1^, Jacqueline Villeneuve-Ahmed^2^, Chloe Hunt^2^, Hayley Roulstone^3^

^1^Simon Fraser University, ^2^She Matters, ^3^Rise Consulting Ltd.

**Introduction**: Sexual assault is a pervasive issue with significant long-term health consequences. Survivors are at risk of chronic pain conditions related to their assault (Damin, 2023; Zubieta et al., 2023; Cichowski et al., 2017; Sledjeski et al., 2008; Yudenfreund, 2003). The severity, frequency, and type of assault, as well as the victim’s age, increase the likelihood of chronic pain (Damin, 2023; Gómez-Pérez & López-Martinez, 2013; Hellman et al., 2018; Cichowski et al., 2017).

Indigenous women in Canada are disproportionately affected by sexual violence. 60% experience physical assault and almost half experience sexual assault in their lifetime (Heidinger, 2022). Mistrust of the healthcare system, medical trauma, systemic racism, and intergenerational trauma further hinder Indigenous women’s choices to seek care (Public Health Agency of Canada, 2024). Survivors encounter systemic re-traumatization within healthcare settings, inhospitable spaces, limited or no access to Sexual Assault Evidence Kits (SAEK), and a lack of culturally competent care (She Matters, 2021; Wadsworth et al., 2019).

**Methods**: This study employed a genocide-informed, trauma wise approach that integrated Indigenous Storywork and Focusing-Oriented Therapy (Archibald, 2008; Turcotte & Schiffer, 2014; Wilson, 2012). Sixty survivors and service providers, both Indigenous and non-Indigenous from Northern Ontario, Northern British Columbia, and the Yukon, shared their experiences through ‘survivance’ narratives (Vizenor, 1994).

**Results**: The study identified a range of non-pharmacological, land-based, and embodied approaches that survivors desire. By addressing immediate needs the onset of chronic pain conditions may be mitigated. Findings highlight the need for systemic change in healthcare settings driven by Indigenous relational ethics and kinship values.

**Discussion/Conclusions**: Research outcomes offer insights into the relationship between sexual assault and chronic pain. Survivors propose modalities for the pursuit of care following sexual assault disclosure to disrupt physical, intellectual, and emotional experiences of chronic pain longer term. Indigitalized and kinship modalities are outlined.

## Postoperative Pain Intensity in South Asian Females with Breast Cancer Undergoing Mastectomy or Lumpectomy: A Feasibility Study

Jasleen Farwaha^1^, Monakshi Sawhney^1^, Katie Goldie^1^, Shaila Merchant^2^

^1^Queen’s University, ^2^Kingston Health Sciences Center

**Introduction**: South Asian females are diagnosed with breast cancer at a later stage compared to other Canadian females^1^. This may be due to language barriers and fear of stigma in their community^1,2,3^. Unfortunately, this can lead to the need for more extensive surgery. Following surgery, South Asian females experience acute pain that may lead to chronic postoperative pain^4^. Unfortunately, research investigating pain in South Asian females who undergo surgery for breast cancer in Canada is lacking. This study aimed to examine the feasibility of recruiting South Asian females and to examine pain following breast surgery.

**Methods**: This study included South Asian females from a breast cancer clinic in the Greater Toronto Area. Participants were recruited in person or over the telephone. Pain was examined using the BPI-SF on postoperative days (POD) 1 and 7. Analgesic use and side effects were also examined.

**Results**: Overall, 23 out of 29 eligible South Asian females consented to participate. The mean age of participants was 58.8 years old (SD 13.3) and most were from India (60.9%). Participants reported a moderate amount of pain on POD 1 (“worst” pain = 6.3 (SD 2.4)) and POD 7 (4.5 (SD 2.5)). Pain interfered with normal work, general activity, and sleep.

**Discussion/Conclusions**: South Asian females who undergo breast surgery are willing to participate in studies that may help others. Participants reported a moderate amount of pain up to 7 days after surgery. Education regarding how to manage pain, and analgesic protocols may help this population better manage their pain.

## Transgender Implications in Migraine Disease: A Brief Review and Discussion

Karen Ferreira^1^, Ana Miriam Velly^2^

^1^Neurologist, Headache Clinic, Montreal Neurological Clinic, Montreal, Quebec, Canada., ^2^Associate Professor, Faculty of Dental Medicine and Oral Health Sciences, McGill University, Jewish General Hospital, Montreal, Quebec, Canada

**Introduction**: Migraine is one of the most prevalent disorders globally, leading to significant social costs and expensive treatments. Its onset is influenced by hormonal, psychological, and social factors. Although research on migraine in transgender individuals is limited, emerging evidence suggests unique characteristics related to diagnosis, management, and treatment, influenced by hormonal therapy, stigma, and healthcare disparities. This review aims to summarize key points regarding migraine in transgender patients and highlight relevant research in this field.

**Methods**: A systematic review was conducted using databases PUBMED/MEDLINE and Embase, covering studies from inception to January 2025. Eligible studies assessed the implications of transgender status on migraine.

**Results**: A total of 102 studies were identified, of which 9 were ultimately included in the review. Among these, seven (77.8%) were reviews – 6 focused on the epidemiology and underlying mechanisms of migraines in transgender individuals, and 1 addressed treatment. Additionally, 2 (22.2%) were case reports or case series. Overall, transgender individuals undergoing gender-affirming hormone therapy (GAHT) may experience migraine patterns that reflect their affirmed gender. Estrogen therapy in transgender women may lead to an increased frequency of migraines, while the effects of testosterone therapy on transgender men are controversial, with some studies suggesting it may either increase or decrease migraine occurrence. Furthermore, these individuals often experience elevated stress levels due to stigma and discrimination, which can trigger headaches.

**Discussion/Conclusions**: Further research on gender identity minorities is needed to clarify the complexities surrounding migraines in this population, with the goal of delivering the best level of care.

## Pain Beliefs in the Military: A Qualitative Study Exploring the Perspectives of Instructors Within the Canadian Armed Forces on How Pain is Addressed in Basic Training and the Broader Military Culture

Mael Gagnon Mailhot^1, 2^, Peter Stilwell^3, 4^, M. Gabrielle Pagé^1, 2, 5^, Derek Speirs^6^, Hélène Le Scelleur^7, 8^, Timothy Wideman^3, 4^

^1^Department of Psychology, Université de Montréal, Montreal, Quebec, Canada, ^2^Centre de recherche du Center hospitalier de l’Universite de Montréal (CRCHUM), ^3^School of Physical and Occupational Therapy, McGill University, Montreal, Quebec, Canada, ^4^Centre for Interdisciplinary Research in Rehabilitation of Greater Montreal (CRIR), IURDPM, CIUSSS-Center-Sud-de-l’Ile-de-Montréal, Montreal, Quebec, Canada, ^5^Department of Anesthesiology and Pain Medicine, Université de Montréal, Montreal, Quebec, Canada, ^6^Patient partner, ^7^School of Social Services, Ottawa University, Ottawa, Ontario, Canada, ^8^Chronic Pain Center of Excellence, Hamilton, Ontario, Canada

**Introduction**: Pain beliefs play an important role in how people understand and cope with pain, and these are especially relevant for military personnel and Veterans who bear a disproportionately high burden among those living with pain. However, limited research explores what beliefs about pain are central in military training and the broader military culture, and how these beliefs may influence self-management and healthcare utilization.

**Methods**: This participatory qualitative descriptive study explored what beliefs about pain are emphasized in basic military training and the broader military culture related to the Canadian Armed Forces (CAF). We conducted two focus groups with eight CAF instructors and inductively coded the transcripts.

**Results**: Three themes were generated. Theme 1 describes how instructors aim to teach recruits to prioritize mission and team success by pushing through pain and distinguishing it from injury. Theme 2 outlines the culture around pain in training and the broader military. Injuries are often experienced as personal failures and threaten one’s identity. Thus, instructors reported that military personnel often feel pressured to endure painful injuries as seeking care is stigmatized. Theme 3 describes instructors’ critical reflections on how pain is addressed within the military, emphasizing that the military’s relationship with pain is a double-edged sword with both helpful and harmful aspects.

**Discussion/Conclusions**: These findings illustrate a tension between the military’s operational goals and the effective management of pain and injuries. Implications for military training and future research are discussed, including clinical research addressing these beliefs among Veterans suffering with pain.

## Thinking About Pain: The Relationship Between Cognitive Factors and Genitopelvic Pain in Racialized Populations

Melody Garas^1^, Caroline F. Pukall^1^

^1^Queen’s University

**Introduction**: Genitopelvic pain is highly prevalent, and its experience has been associated with negative cognitions. Cognitive factors have been understudied compared to other psychosocial outcomes (e.g., depression), and racial minorities have been historically underrepresented in genitopelvic pain literature.

**Methods**: This study investigated pain cognitions in racialized and White populations with genitopelvic pain. Participants were 72 racialized individuals and 154 White individuals, recruited for a larger online study. Cognitive factors were assessed through validated scales, and results were analyzed with t-tests and regressions.

**Results**: White participants scored significantly higher on the activity engagement subscale of the Chronic Pain Acceptance Questionnaire and reported thinking about their pain symptoms in the past week significantly more than racialized participants. Compared to White participants, racialized participants had significantly higher overall levels of pain coping and pain resilience. Activity engagement, overall pain resilience, and cognitive/affective pain resilience negatively predicted pain intensity in racialized participants; frequency of pain thoughts in the past week positively predicted pain intensity in both groups.

**Discussion/Conclusions**: Cognitive factors may predict pain intensity differentially based on social location. Factors associated with racialized status (e.g., minority stress, discrimination) may shape cognitive responses to pain. For example, the Superwoman Schema describes how Black women feel they must manifest strength and push through their pain, which may partly explain why racialized participants think about their pain symptoms less than White participants and demonstrate more resilience. These results have clinical implications for those treating racialized populations with genitopelvic pain with cognitive-based treatments, in ensuring the development of tailored treatment programs.

## Caregiver Needle Worry Pre- and Post-Toddler vaccination: Caregiver Predictors at 12-, 18-, and 24-months

Nichaela Garvey^1^, Deena Savlov^2^, Eitan Weinberg^2^, Dan Flanders^2^, Hartley Garfield^2^, Rebecca Pillai Riddell^1^

^1^York University, ^2^University of Toronto

**Introduction**: Caregiver needle worry has been linked to greater child pain during vaccination. Thus, it is imperative to understand predictors of caregiver needle worry pre- and postchild vaccination. The current study aimed to explore whether caregiver psychopathology symptomology and parental stress were predictors of pre- and postneedle worry in early-late toddlerhood (12–24 months).

**Methods**: The study included parent-toddler dyads who participated in at least 1 vaccination at 12 (n = 162), 18 (n = 137) or 24-months of age (n = 54). After the appointment, caregivers completed the Brief Symptom Inventory (BSI) and Parent Stress Index (PSI). Caregivers were also asked to rate their pre- and postneedle worry on a scale from 0 to 10.

**Results**: Bivariate relationships between potential predictors and the outcome variables (pre- and postworry) were initially examined. Simple linear regressions were then run for 2 models at each age, including all the predictor variables that had a significant relationship with pre- or postworry. Across the ages, preneedle worry predicted postneedle worry (p < .001). Unique to 12 months, parental distress predicted postneedle worry (b = 0.06, p = .02). Unique to 18 months, total stress predicted preneedle worry (b = 0.11, p = .002). Unique to 24 months, anxiety predicted preneedle worry (b = 0.07, p = .04).

**Discussion/Conclusions**: Caregiver psychopathology symptomology and parental stress related to pre- and postneedle worry during toddler vaccination. Notably earlier in development, aspects of parental stress predicted needle worry, whereas later in development aspects of caregiver psychopathology predicted needle worry.

## Exploring the Impact of Parental Stress and Distress on Infant Pain Responses over Time

Lojain Hamwi^1^, Andrea Lebovic^1^, Deena Savlov^2^, Eitan Weinberg^2^, Dan Flanders^2^, Hartley Garfield^2^, Rebecca Pillai Riddell^1^

^1^York University, ^2^University of Toronto

**Introduction**: Parents and caregivers play a critical role in shaping infant development, including how infants respond to pain. Research suggests parental anxiety and stress can significantly impact a child’s pain experience (Lee et al., 2013; Coric et al., 2014). This study examined the relationship between parental anxiety, stress, and the trajectory of infants’ pain-related responses during routine immunization across three time points.

**Methods**: Parents of infants at 12 months (N = 162), 18 months (N = 148), and 24 months (N = 58) provided self-reports of anxiety (BSI) and parenting stress (PSI-4-SF). Infant pain-related distress was measured using the FLACC scale, which assesses five behaviors (face, legs, activity, cry, consolability). FLACC was coded at baseline (15 seconds preneedle), immediately postneedle (15 seconds), and during recovery (2 minutes). Linear mixed-effects models examined the effects of parental anxiety and stress on FLACC scores across these phases.

**Results**: At baseline, significant interactions were observed between parental stress and time, indicating higher parental stress was associated with greater distress behaviors over time (F(1, 120.408) = 5.680, p = .019). Immediately postneedle, neither parental anxiety nor stress significantly predicted FLACC scores, though trends suggested greater pain-related behaviors. During recovery, no significant associations were observed between parental distress and recovery behaviors, suggesting resilience in infants’ distress reduction.

**Discussion/Conclusions**: These findings highlight the nuanced role of parental psychological factors in shaping infant pain responses. Elevated parental stress influences baseline distress behaviors before the needle but not immediate or recovery phases. Interventions addressing parental well-being may help reduce infants’ heightened distress during vaccinations.

## The Relationship Between Parental Ratings of Infant Temperament and Parental Soothing Behaviors in Response to Infant Pain

Haleh Hashemi^1^, Dan Flanders^2^, Eitan Weinberg^2^, Deena Savlov^2^, Hartley Garfield^2^, Rebecca Pillai Riddell^1^

^1^York University, ^2^University of Toronto

**Introduction**: Parental perceptions of infant temperament may influence caregiving behaviors, particularly in emotional regulation contexts, such as pain. Although previous research has linked parental perceptions of temperament to caregiving styles, less is known about how these perceptions influence soothing behaviors during acute pain. This study examines parent’s soothing behaviors and its relationship with infant’s pain response, after accounting for infant temperament.

**Methods**: Parent-infant dyads were followed during toddlers’ 12- (N = 121), 18- (N = 111), and 24-month vaccination visits (N = 46). FLACC scores (Face, Legs, Activity, Cry, Consolability) and the Measure of Adult and Infant Soothing and Distress (Distraction, Rocking, Verbal Reassurance, Physical Comfort) were coded at baseline, needle, and 1-minute postneedle. Parents completed the Early Childhood Behavior Questionnaire during follow-up. Nine hierarchical multiple linear regressions were conducted.

**Results**: At 12 months, the overall model significantly predicted infant behavior at baseline (F (7,107) = 3.23, p = .004, R^2^ = .174) with parent distraction at baseline as significant predictor (b = .326, t = 3.88, p < .001). At 18 months, model significantly predicted infant behavior at post 1-minute (F (15, 93) = 2.455, p = .005, R^2^ = .284) with distraction (b = .298, t = 3.100, p = .003) and verbal reassurance (b = .340, t = 3.155, p = .002) at post 1-minute being significant predictors. At 24 months model significantly predicted infant behavior at baseline (F (7, 37) = 2.910, p = .016, R^2^ = .355) with distraction at baseline as significant predictor (b = .494, t = 3.51, p = .001).

**Discussion/Conclusions**: The findings indicate that parents’ soothing behavior had an increasing role on infant pain behavior, over age (12, 18 and 24 months), regardless of parent-reported infant temperament.

## Music and Targeted Neonatal Echocardiography: Insights from Neonatal Healthcare Workers

Joshua Hazan Mea^1^, Daniela Villegas Martinez^2^, Stephanie Mardakis^2^, Elissa Remmer^2^, Tíscar Cavallé-Garrido^3^, Gabriel Altit^2^

^1^Division of Neonatology, Faculty of Medicine and Health Sciences, McGill University, Montreal, QC, Canada, ^2^Division of Neonatology, Department of Pediatrics, Montreal Children’s Hospital, Montreal, QC, Canada, ^3^Division of Cardiology, Department of Pediatrics, Montreal Children’s Hospital, Montreal, QC, Canada

**Introduction**: The NICU is stressful for newborns, families, and healthcare teams, with routine procedures exacerbating discomfort and pain. Music has been shown to reduce pain and stress in preterm infants and alleviate maternal anxiety. The Neonatal Hemodynamics (NH) team at the Montreal Children’s Hospital (MCH) uses music and other comfort measures during targeted neonatal echocardiography (TNE) to mitigate patient discomfort and pain. The objective is to assess healthcare professionals’ perspectives on the impact of comfort measures implemented during TNE.

**Methods**: Survey distributed to MCH NICU professionals. Responses were collected for 4 weeks and analyzed using descriptive statistics.

**Results**: In all, 110 surveys were analyzed. Most respondents were women (85%) and nurses (71%), with 48% having >10 years of neonatal care experience. Median age was 37 years (IQR: 31–43). Most believed scans disturbed the infant (71%) by increasing hypothermia risk (75%), lability (67%) and sensitivity to manipulation (57%). Most valued during TNE were warm gel (85%), bundling (80%), and a focused exam (≤30 minutes) (80%). Respondents most often characterized music as being fairly important during TNE (41%). Workers believed music helped calm the neonate (73% agree vs 3% disagree, rest neutral), parent (44% vs 3%) and sonographer (39% vs 5%), with most preferring neoclassical music recordings (53%) over other forms of music delivery.

**Discussion/Conclusions**: Professionals agree that scans disturb newborns, but implementing comfort measures, such as our NICU’s cost-efficient bundle, may diminish discomfort and pain. Music was seen as beneficial for calming babies, parents and sonographers, potentially supporting its integration into routine care.

## Maternal Influences on Infant Physiological Responses During a Heel Lance

Sara Jasim^1^, Carol Cheng^2^, Vibhuti Shah^2^, Rebecca Pillai Riddell^1, 3, 4^

^1^ York University, ^2^Mount Sinai Hospital, ^3^The Hospital for Sick Children, ^4^University of Toronto

**Introduction**: Preterm infants in the neonatal intensive care unit (NICU) depend on maternal regulation during distress (e.g., heel lance procedure), but maternal perceived stress and physiological arousal can impact this process. The goal of the analysis was to determine how maternal self-report of NICU stress impact infant physiological arousal before and after a heel lance while in maternal skin-to-skin care, after controlling for maternal physiological arousal.

**Methods**: Participants were 18 mother-preterm dyads recruited from a large tertiary hospital. Self-reported maternal stress was collected via the Parental Stressor Scale-NICU (PSS-NICU). Infant and maternal heart rate (HR) were collected simultaneously at three phases of a heel lance procedure: baseline (60–29 seconds prelance), lance (0–29 seconds at lance), and recovery (60–89 seconds postlance).

**Results**: Moderate bivariate correlations were found between PSS-NICU and infant HR at baseline, lance, and recovery. Regression analyses revealed that PSS-NICU was not predictive of infant HR at any timepoint after controlling for the influence of maternal HR. Maternal baseline HR was negatively predictive of infant HR at lance (β = −1.38) and recovery (β = −1.65) heart rates. Maternal lance HR was positively associated with infant lance HR (β = 1.44) and recovery HR (β = 2.37).

**Discussion/Conclusions**: Maternal physiological arousal showed stronger links to infant physiological arousal than maternal self-reported stress, reflecting potential underlying physiological regulatory processes. Baseline maternal arousal was associated with lower infant heart rates during lance and recovery, while higher maternal arousal during lance was associated with higher infant heart rates at those timepoints.

## Oral Versus Patient-Controlled Opioids Analgesia for Total Knee and Hip Arthroplasty: A Systematic Review and Meta-Analysis of Randomized Controlled Trials

Harjind Kahlon^1^, Armaanpreet Dhillon^2^, Wenjun Jiang^2^, Jeevan Dhillon^3^, Wahaj Khan^4^, Rachel Couban^4^, James Paul^5^, Li Wang^5^

^1^Temerty Faculty of Medicine, University of Toronto, Toronto, ON, Canada, ^2^Faculty of Health Sciences, McMaster University, Hamilton, ON, Canada, ^3^Faculty of Science, McMaster University, Hamilton, ON, Canada, ^4^Michael DeGroote School of Medicine, McMaster University, Hamilton, ON, Canada, ^5^Department of Anesthesia, McMaster University, Hamilton, ON, Canada

**Introduction**: This systematic review aimed to assess the comparative effectiveness of opioids administered orally vs. in patient-controlled analgesia (PCA) among patients with total knee or hip arthroplasty (TKA/THA).

**Methods**: We searched MEDLINE, Embase, CINAHL, CENTRAL, and Web of Science up to March 2024 for randomized controlled trials (RCTs) examining oral opioids vs. opioid PCA for patients with TKA/THA. We conducted random-effects meta-analysis.

**Results**: Nine RCTs comprising 726 patients proved eligible, including eight RCTs for TKA and one for THA. The median sample size is 73 (IQR: 60–110). Two RCTs were at high risk of bias. Low-to- moderate certainty evidence shows no or little difference between oral opioids and PCA in postoperative pain at rest (24 hours: weighted mean difference [WMD] 0.38 on 0–10 NRS, 95%CI −0.42 to 1.19; 48 hours: WMD −0.12, 95%CI −0.60 to 0.36; and 72 hours: WMD −0.11, 95%CI −0.51 to 0.30), pain at movement (24 hours: WMD 0.33, 95%CI −0.46 to 1.13; 48 hours: WMD −0.08, 95%CI −0.62 to 0.45; and 72 hours: WMD −0.78, 95%CI −1.73 to 0.17), opioid consumption at 24 hours (WMD 0.16 mg, 95%CI −16.25 to 16.58 mg), nausea (relative risk [RR] 0.76, 95% CI 0.52 to 1.10), or vomiting (RR 0.97, 95% CI 0.51 to 1.85). However, opioid PCA may reduce opioid consumption at 48 hours (WMD 13.01 mg, 95%CI 1.08 to 24.94 mg).

**Discussion/Conclusions**: Compared to opioid PCA, oral opioids may have similar effect on postoperative pain management without increase in adverse events although a small increase in opioid consumption at 48 hours.

## Trauma and Post-Hospital Behavior Problems Following a Pediatric Emergency Department Visit

Ayesha Kamran^1, 2^, Sankait Rattu^2, 3^, Jillian Vinall Miller^2, 4, 5^, Jennifer Thull-Freedman^2, 6^, Neta Bar Am^2, 7^

^1^Department of Psychology, ^2^University of Calgary, ^3^Department of Anesthesiology, ^4^Department of Anesthesiology, Perioperative & Pain Medicine, ^5^Cumming School of Medicine, ^6^Department of Pediatrics and Emergency Medicine, ^7^Department of Pediatrics

**Introduction**: Pain and distress can result from medical treatment at the emergency department (ED). Venipunctures are common and may be painful and/or stressful to children and their parents. This study compared parent and child behavioral outcomes after an ED visit between groups of children requiring a venipuncture versus an assessment.

**Methods**: Two groups of parents with children aged 3–9 years were recruited from the pediatric ED. The pain group included parents (n = 46) of children requiring bloodwork or IV insertion. The non-pain group included parents (n = 75) of children who underwent evaluation without needlework. Within 10 days of the ED visit (baseline) and again four months later, parents reported their and their child’s post-traumatic stress symptoms (PTSS), their and their child’s anxiety symptoms in the ED, and whether their child was held down for a procedure. Mean comparisons were applied to examine differences in symptomology between the groups, and linear regression was conducted to explore the baseline factors associated with post-hospital behavior changes at 4-months follow-up.

**Results**: Parent and child PTSS was found to be similar between the pain and no-pain groups. However, children in the pain-exposed group had greater apathy-withdrawal symptoms compared to children in the no-pain group (p = .02, Effect size = 0.12). Linear regression revealed that greater child PTSS at baseline and being held down for procedural pain was associated with greater apathy-withdrawal symptoms at 4-month follow-up (p = .006, R^2^ = 0.48).

**Discussion/Conclusions**: Preliminary evidence suggests that standardization of pain management in the ED is necessary to avoid causing long-term harm to children.

## Public Perspectives on Myalgic Encephalomyelitis/Chronic Fatigue Syndrome: A Twitter Thematic and Sentiment Analysis

Iliya Khakban^1^, Shagun Jain^1^, Joseph Gallab^1^, Blossom Dharmaraj^1^, Cynthia Lokker^1^, Wael Abdelkader^1^, Dena Zeraatkar^1^, Jason Busse^1^

^1^McMaster University

**Introduction**: Myalgic encephalomyelitis (ME), also referred to as chronic fatigue syndrome (CFS), is a complex illness that typically presents with disabling fatigue, cognitive dysfunction, and functional impairment. We explored public discourse on Twitter/X to understand the concerns and priorities of individuals living with ME/CFS.

**Methods**: We used the Twitter application programming interface to collect tweets related to ME/CFS posted between January 1st, 2010, and January 30th, 2024. We sampled 1,000 random tweets from each theme, which were independently reviewed in duplicate to identify subthemes and representative quotes.

**Results**: We retrieved 905,718 tweets, of which 53% were neutral, 38% were negative, and 9% were positive. Tweets mentioning fibromyalgia or long COVID acknowledged the similarities with ME/CFS, stigmatization associated with these disorders, and lack of effective treatments. Physician-related tweets often described frustration with ME/CFS labeled as mental illness, dismissal of complaints by healthcare providers, and the need to seek out “good doctors,” who viewed ME/CFS as a physical disorder. Tweets on research typically praised studies of biomarkers and biomedical therapies, called for greater investment in biomedical research, and expressed frustration with studies that suggested a biopsychosocial etiology for ME/CFS or those supporting management with psychotherapy or graduated activity.

**Discussion/Conclusions**: Our findings suggest that public discourse on Twitter regarding ME/CFS highlights stigmatization and dismissal by physicians; frustration with management approaches focused on activity and psychotherapy; a desire for research that validates a biomedical model of etiology and effective treatments for ME/CFS; and an overlap between fibromyalgia, long COVID, and ME/CFS.

## The Pain in Pregnancy Program: Strengths and Gaps in Peripartum Pain Management

Christine Krupa^1^, Elaine Teh^2^, Erika Cheung^2^, Rebecca Titman^2, 3^

^1^University of Ottawa Faculty of Medicine, ^2^Sinai Health, ^3^University of Toronto

**Introduction**: Pain during pregnancy and postpartum is common, yet few healthcare professionals feel comfortable supporting these patients. The combination of limited interdisciplinary programs, sparse literature and a lack of education of prenatal providers contribute to the under treatment of peripartum pain. This quality improvement project aimed to evaluate the characteristics of patients referred to a dedicated pain in pregnancy (PIP) program at a high-risk obstetrics center and understand patient healthcare experiences.

**Methods**: Anonymized surveys through a secure survey portal were utilized to assess patient perceptions of strengths and gaps of the program. A retrospective chart review was completed to obtain demographic characteristics of patients assessed in the PIP program.

**Results**: The survey was sent to 77 patients with 22 responses (response rate of 28.6%). Almost every patient indicated that the most helpful part of the initial assessment was enhanced support around their pain management, with dedicated time, education and clear communication being most appreciated. The retrospective chart review indicated that common diagnoses included musculoskeletal pain and headaches. Majority of participants identified as white (n = 10) and completed postgraduate education (n = 14). Gaps identified include failing to catch patients with lower socioeconomic status and missed opportunities for patient referrals.

**Discussion/Conclusions**: A dedicated PIP program is addressing a large gap in women’s health by providing support and resources for pregnant individuals experiencing pain. Future directions include strengthening of care pathways and adding to members of the multidisciplinary team (e.g., physiotherapy) to further meet the care needs of pregnant individuals with pain.

## Impacts of the iCanCope Digital Pain Self-Management Intervention Among Adolescents and Young Adults with Chronic Pain: A Randomized Controlled Trial

Chitra Lalloo^1^, Fareha Nishat^1^, Bruce Dick^2^, Lori Montgomery^3^, Fiona Campbell^1, 4^, Tonya Palermo^5^, Tania Di Renna^6^, Jill Chorney^7^, Ayala Gorodzinsky^7^, Karim Mukhida^8^, Mark Simmonds^2^, Krista Baerg^9^, Melanie Noel^10^, Patricia Poulin^11^, Ramesh Zacharias^12^, Joseph Cafazzo^13^, Quynh Pham^13^, Carley Ouellette^12^, Vina Mohabir^1^, Lauren Harris^1^, Cleo Davies- Chalmers^1^, Jennifer Stinson^1^

^1^The Hospital for Sick Children, ^2^University of Alberta, ^3^University of Calgary, ^4^University of Toronto, ^5^Seattle Children’s Research Institute, ^6^Toronto Academic Pain Medicine Institute, ^7^IWK Health Center, ^8^Nova Scotia Health Authority, ^9^University of Saskatchewan, ^10^Alberta Children’s Hospital, ^11^The Ottawa Hospital, ^12^Hamilton Health Sciences, ^13^University Health Network

**Introduction**: iCanCope™ (ICC) is a digital self-management program for 15–25-year-olds with chronic pain. We aimed to evaluate program effectiveness and satisfaction via two-arm randomized controlled trial in Canadian tertiary-care clinics.

**Methods**: Intervention participants received ICC (app and website with symptom tracking, self-management skills, goal-setting, community, and pain education) while active-control participants accessed a similarly-branded app and website with limited features (i.e., symptom tracking, pain education). Outcomes were assessed at baseline, postprogram (2-months; T2), and 6-months (T3). Clinical outcomes were assessed using validated measures. Pain intensity (primary outcome) and satisfaction were assessed using the Brief Pain Inventory and Acceptability e-Scale (score range: 1.0–5.0), respectively. Linear mixed-effects models were applied.

**Results**: N = 302 participants were enrolled and n = 297 (mean age = 17.1 ± 2.4, 83% female) analyzed (ICC = 147, Active-Control = 150). Outcome completion rates were 89% and 71% for T2 and T3, respectively. Program engagement was similarly robust across groups (e.g., ≥ 92% engaged with symptom tracking). Although both groups trended toward improvement, no significant treatment-group effects were identified. A treatment-time effect (β = −0.53, p = .027) indicated improved pain intensity among intervention participants at T3. Both groups were highly satisfied with their proffered app (mean 4.2 ± 0.9 for intervention; 4.2 ± 0.9 for active-control) and website (mean 4.0 ± 1.0 for intervention; 4.0 ± 1.0 for active-control).

**Discussion/Conclusions**: Use of an active-control, while aligned with digital trial recommendations, may have diminished the ability to detect treatment-group effects. Given high satisfaction and data suggesting long-term benefit, ICC has undergone French translation and been integrated into the Power over Pain Portal (https://popyouth.ca) as a free self-management tool for Canadian youth.

## Most Used and Perceived Effectiveness of Non-Pharmacological Interventions for Acute Pain Management of Critically Ill Adults in Quebec

Geneviève Laporte^1, 2^, Amanda Guerin^1^, Émilie Gosselin^3, 4^, Robin Kagie^2^, Caroline Arbour^5, 6^, Francis Bernard^6, 7^, Virginie Williams^8^, David Williamson^6, 9^, Julie Houle^10, 11^, Nathalie Tiffault^11^, Han Ting Wang^7, 12^, Marc Perreault^9, 13^, Andréa Maria Laizner^13, 14^, Céline Gélinas^1, 2^

^1^Ingram School of Nursing, McGill University, ^2^Centre for Nursing Research and Lady Davis Institute, ^3^École des sciences infirmières, Université de Sherbrooke, ^4^Centre de recherche du CHUS, ^5^Faculté des sciences infirmières, Université de Montréal, ^6^Hôpital du Sacré-Coeur de Montréal, ^7^Faculté de médecine, Université de Montréal, ^8^Équipe de recherche en soins infirmiers, Hôpital du Sacré-Coeur de Montréal, ^9^Faculté de pharmacie, Université de Montréal, ^10^Département de sciences infirmières, Université du Québec à Trois-Rivières, ^11^CIUSSS Mauricie-Center-du-Québec, ^12^Centre Hospitalier de l’Université de Montréal, ^13^Centre universitaire de santé McGill, ^14^Institut de recherche du Center universitaire de santé McGill

**Introduction**: Nonpharmacologic interventions (NPI) are integral to a multimodal approach to pain management in the intensive care unit (ICU). NPI play an essential role in preventing unrelieved acute pain, but limited understanding of patient NPI preferences may hinder their implementation in the ICU. Consequently, we aimed to describe the use of NPI and their perceived effectiveness from ICU patients’ perspectives.

**Methods**: In this descriptive study, adult participants were recruited from 5 mixed ICUs in Quebec. Participants reported their pain with the Brief Pain Inventory, recounted the use of NPI in the last 24 hours from a 16-item checklist and rated their perceived effectiveness (1 = ineffective to 4 = very effective).

**Results**: A convenience sample included 350 ICU survivors (34% women), aged from 18 to 98 years old. Participants reported an average pain intensity of moderate level (mean = 4.4/10; SD = 2.2). Patients used an average of 7 different NPI, with significant differences (p = .03) observed between patients reporting low (0–3) and high (4–10) average pain levels (6.3 vs 7.2). Family presence/support (77%), positioning (73%) and emotional support (69%) were the most used NPI. Family presence and emotional support had the highest perceived effectiveness (median = 4, IQR = 3–4) in relieving ICU pain. Positioning was considered less effective (median = 3, IQR = 3–4) than other less employed NPI like information-sharing (median = 4, IQR = 3–4) and massage (median = 4, IQR = 3–4).

**Discussion/Conclusions**: Our findings align with recommendations for ICU family-centered care, with family presence identified as crucial for patient pain management. Patients’ preferences of NPI and their perceived effectiveness should be integrated in ICU multimodal analgesia approaches.

## Examining the Impact of Infant Care Experiences on the Effectiveness of Skin-to-Skin Contact for Acute Pain in Premature Infants in the NICU

Andrea Lebovic^1^, Sara Jasim^1^, Carol Cheng^2^, Vibhuti Shah^2^, Rebecca Pillai Riddell^1^

^1^York University, ^2^Sinai Health

**Introduction**: Skin-to-skin contact (SSC) is a pain intervention for preterm infant in the Neonatal Intensive Care Unit(NICU); however, research on the efficacy has been mixed. Premature infants in NICUs experience frequent painful procedures and with their underdeveloped nervous systems, the recurrent subjection to painful procedures may decrease the infant’s pain thresholds and impede on cognitive development. Considering mother-infant attachment as a primary context for understanding infant pain responses, this study aimed to examine whether prior infant care behaviors related to SSC effectiveness during a painful procedure.

**Methods**: Participants included 21 premature infants held in SSC with mother. Pain responding was measured via heart rate (HR) at three phases of a heel lance: before (baseline), during (reactivity), and after (regulation). Mothers completed a self-report questionnaire regarding duration of time spent engaging in infant care behaviors (SSC, facilitated tucking, infant handling) over 2 weeks prior to the study. Correlation analyses were run.

**Results**: Infant handling and regulation HR were negatively correlated (r = −.26). Facilitated tucking was positively correlated with baseline HR (r = .28) and reactivity HR (r = .18). Lastly, SSC was positively correlated with baseline HR (r = .35) and reactivity HR (r = .30).

**Discussion/Conclusions**: Results suggest that prior duration of infant care behaviors were correlated with infant HR at various stages of a painful procedure and therefore may be related to the effectiveness of SSC for premature infant pain management. The results provide insight into initial mother-infant attachment in relation to acute pain in a hospital setting.

## Exploring Maternal-Infant Regulation Attunement During a Painful Event in the Neonatal Intensive Care Unit: The Role of Maternal Socioeconomic Stressors

Arianna Leguia^1^, Lojain Hamwi^1^, Vibhuti Shah^2^, Carol Cheng^2^, Rebecca Pillai Riddell^1^

^1^York University, ^2^Mount Sinai Hospital

**Introduction**: Stress impedes a caregiver’s ability to attune and regulate their infant (Mueller et al., 2021). This study aims to examine dyadic attunement between a mother and her preterm infant during a distressing event and how maternal socioeconomic stressors impact their attunement patterns.

**Methods**: Mother and infant dyads (n = 42) were observed during a routine heel lance procedure in a Neonatal Intensive Care Unit (NICU). Mother and infant heart rate (HR) were recorded across four different epochs: baseline (1 min pre heel-lance), immediate postlance (1 min following heel-lance), and 2 regulation periods (3 and 5 mins following heel-lance). Mothers were asked to report their socioeconomic stressors via a self-report questionnaire. Line graphs examined HR patterns of mother and infant at different socioeconomic levels. A correlational analysis was conducted to understand the attunement between maternal and infant HR across the different epochs within the heel lance procedure.

**Results**: No significant differences in HR averages were found between mothers with high socioeconomic stress (n = 20) and mothers with low socioeconomic stress (n = 13), across all epochs. Mothers and infants demonstrated a similar average HR pattern during baseline and the postlance regulation periods. However, immediately postlance, infants’ HR was significantly higher than maternal HR. Contrary to previous research, no significant correlations were found between mothers and their preterm infants across all epochs.

**Discussion/Conclusions**: Further research is required to examine the impact of external stressors on dyadic attunement in the NICU to support mothers who experience various distressing events more frequently.

## Exploring the Connection Between Pain Catastrophizing and Pain Disability in a Diverse Sample with Endometriosis

Samantha L. Levang^1^, Caroline F. Pukall^1^

^1^Queen’s University

**Introduction**: Endometriosis is a chronic condition marked by significant pain and disability, yet the interaction between psychological and physical factors remains underexplored. The current study sought to examine the relationship between pain catastrophizing – characterized by rumination, magnification, and helplessness – and pain disability across various life domains in a diverse sample of individuals with endometriosis.

**Methods**: A total of 686 individuals with a self-reported clinician-identified diagnosis of endometriosis participated in this online study. Two-tailed independent sample t-tests were used to examine between-groups differences in pain disability and pain catastrophizing among those below and above clinically relevant moderate pain intensity levels. Between-groups differences in pain disability among those below and above the clinically relevant pain catastrophizing level, as well as between-groups differences in pain catastrophizing among those below and above the clinically relevant moderate pain disability level, were also analyzed.

**Results**: Results demonstrated strong associations between higher pain intensity and increased levels of both pain catastrophizing and disability (p < .001). Clinically relevant pain catastrophizing was significantly linked to greater pain disability across all domains (p < .001). Similarly, higher pain disability was associated with increased catastrophizing (p < .001), suggesting a bidirectional relationship that perpetuates a cycle of chronic pain and psychological distress.

**Discussion/Conclusions**: These findings emphasize the critical role of psychological factors in shaping pain disability, independent of pain intensity. To enhance intervention practices, future research should investigate the temporal connection between catastrophizing and disability. Health care providers are strongly encouraged to assess the impact of endometriosis in patients by adopting a biopsychosocial approach.

## Exploring the Impact of Pain on Canadian Veterans’ Transitions to Civilian Life

Jenny Liu^1^, Dominic Gargala^1^, J. Don Richardson^1^

^1^MacDonald Franklin OSI Research Center

**Introduction**: Pain is a significant barrier to successful transitions from military to civilian life for Canadian Veterans. It affects not only physical health but also psychological resilience, social participation, and access to supports. In a multiyear collaborative project with Veteran Affairs Canada, we sought to explore the prevalence and severity of pain among Veterans, its impact on daily functioning, and how these factors intersect with the challenges of transition and service access.

**Methods**: We surveyed Canadian Armed Forces Veterans to assess health status, pain interference, and quality of life indicators. Data were collected from 233 respondents, focusing on functional limitations, emotional well-being, and social integration.

**Results**: Pain emerged as a pervasive issue, with 47.3% of respondents reporting moderate to extreme interference in normal work activities over the past four weeks. Functional limitations, such as difficulties with moderate activities and climbing stairs, were reported by over 60% of participants. Stratified analyses highlighted profiles of Veterans most impacted by pain.

**Discussion/Conclusions**: Understanding the intersection of pain, transition challenges, and support access is essential to enhancing the quality of life for Canadian Veterans. The findings underscore the critical need for tailored interventions to address the multidimensional impact of pain on Veterans’ lives. Insights gained from this multiyear project offer actionable recommendations for improving service accessibility and resilience-building resources for at-risk Veteran populations.

## Understanding Youth and Caregivers’ Experiences Accessing Community-Based Care for Pediatric Chronic Pain: A Multiple Case Series of Patient Journey Maps

Megan MacNeil^1, 2, 3^, Jaxon Hirtle^2^, Prachi Khanna^3^, Alex Haagaard^3^, Joshua Rash^4^, Abhimanyu Sud^5^, Kate Storey^1^, Kathryn A. Birnie^2, 3^

^1^University of Alberta, ^2^University of Calgary, ^3^Chronic Pain Network, ^4^Memorial University of Newfoundland, ^5^University of Toronto

**Introduction**: Youth with chronic pain face significant barriers to accessing care, including lengthy wait times for specialized pediatric chronic pain clinics. As a result, families often rely on primary/community-based care. This multiple case series study explored the experiences of youth with chronic pain and their caregivers accessing primary care for pediatric chronic pain.

**Methods**: Youth (11–16 year old girls) and caregivers (mothers) participated in separate qualitative interviews using patient journey mapping methodology. Dyads were treated as a “case” and youth and parent interviews were analyzed together. Deductive coding identified (a) patient journey phases (e.g., life before pain, pain symptom onset, accessing healthcare); (b) touchpoints (e.g., health professional visits); (c) facilitators and (d) barriers to care; (e) youth and parent emotions; (f) life factors (e.g., social determinants of health); and (g) chronic pain experience. A visual patient journey map was created for each dyad.

**Results**: Three cases depict unique experiences accessing primary/community-based care in Alberta, Ontario, and Nova Scotia for diverse chronic pain (i.e., CRPS, widespread pain, musculoskeletal pain). They report a wide range in number (1–11) and type of health professionals seen, facilitators and barriers to accessing care (e.g., family/health professional chronic pain knowledge, interaction quality, wait times, care discontinuity), life factors (e.g., rurality, family support), and wide-ranging emotional experience (e.g., hope, frustration).

**Discussion/Conclusions**: Patient journey maps help motivate and inform person-centered health system redesign. This project forms part of the Chronic Pain Network’s knowledge mobilization and implementation science activities to improve chronic pain care for people across Canada.

## New Onset Chronic Pain After Primary Inguinal Hernia Shouldice Repair: A One-Year, Prospective Cohort Study

Marguerite Mainprize^1^, Anton Svendrovski^2^, Ayse Yilbas^1^, Heather Lumsden-Ruegg^3^, Joel Katz^3^

^1^Shouldice Hospital, ^2^UZIK Consulting Inc, ^3^York University

**Introduction**: The purpose was to better understand postsurgical pain development for patients that report no preoperative hernia pain.

**Methods**: REB approval and informed consent were obtained and patients scheduled for primary inguinal hernia repair were recruited and followed for one year. Preoperative measures were collected from surveys administered prior to surgery and included gender, age, stress scores, chronic pain, pain presence (yes or no) and severity (average 0–10 NRS), depression/anxiety symptoms, resilience scores, and pain catastrophizing scores. One-year postoperative surveys captured presence of chronic postoperative pain and severity (average 0–10 NRS). Hernia side, body mass index (BMI) and ASA physical status health classification (ASA) were gathered from patient charts. Descriptive statistics, chi-squared test, Fisher’s Exact test, and bivariate and multivariable analyses were used. P < .05 is considered statistically significant.

**Results**: Of the 1,135 participants recruited, 929 completed the one-year postoperative survey. Of the 346 participants who stated yes to postoperative hernia pain one year after surgery, 71 had been pain-free prior to surgery. Of these 71 participants, 20 reported no pain (NRS = 0), 48 reported mild pain (NRS = 1–3), and 3 reported moderate pain (NRS = 4) one year after surgery. Bivariate binary logistic regression analyses revealed age (p = .003), preoperative stress (p < .001), resilience (p = .006), and pain catastrophizing (p = .025) as significant predictors of the presence of 1-year postsurgical pain. However, multivariate analysis revealed age (p = .033) and preoperative stress (p = .003) as significant predictors.

**Discussion/Conclusions**: Intervention for patients with higher preoperative stress and age may be useful in reducing pain 1 year after primary inguinal hernia Shouldice Repair.

## Treating Osteoarthritis Pain with Green Light Therapy: A One-Way Crossover Clinical Trial

MS O’Brien^1^, S Amey^2^, C DeBow^1^, JJ McDougall^1^, K Mukhida^1^

^1^Dalhousie University, ^2^Nova Scotia Health Authority

**Introduction**: Joint pain is the primary concern for many people living with osteoarthritis (OA). The mainstay of treatment for painful OA is analgesic medications, many of which produce inadequate pain relief and are associated with adverse side effects. Recent clinical evidence suggests that visual exposure to green light therapy (GLT) using dim, narrow-bandwidth green light, reduces pain in migraine and fibromyalgia patients. In preclinical studies, GLT reduces nociception in OA rats, in part through activation of the endocannabinoid system. This study investigated whether GLT could reduce joint pain reported by adults living with knee OA.

**Methods**: A pilot one-way crossover design was used to study the effect of GLT versus control white light therapy in adults with knee OA. The primary outcome was change in arthritis disability score measured using the Western Ontario and McMaster University Osteoarthritis Index (WOMAC). Secondary outcomes were changes in pain measured by the Brief Pain Inventory (BPI), and Numerical Rating Scale (NRS), as well as side effects associated with GLT.

**Results**: Nineteen participants completed both arms of the study. Ten weeks of GLT significantly reduced WOMAC scores (p < .01, paired t-test) and pain measured using the BPI (p < .01, paired t-test). Longitudinal analysis of NRS scores did not reveal a significant difference compared with white light (p > .05, mixed-methods analysis). No adverse side effects were reported.

**Discussion/Conclusions**: This study revealed for the first time that GLT reduced clinical OA pain and disability. Further research is needed to better understand the optimal treatment regime and underlying mechanism of GLT.

## Validity and Reliability of the Pain Resilience Scale in a Complex Postsurgical Sample

Alisha Ratnasekera^1^, Callon Williams^1^, Kristina Axenova^1^, Charly Daley^1^, Amjaad Almohawis^1^, Joel Katz^1^, Hance Clarke^1^, P. Maxwell Slepian^1^

^1^University Health Network

**Introduction**: The Pain Resilience Scale (PRS) is a self-report measure of one’s ability to regulate emotions and cognitions and maintain behavioral engagement despite pain. The PRS has been validated among adults with chronic pain. However, there is limited psychometric information on the PRS among individuals with complex postsurgical pain.

**Methods**: Data are from a retrospective chart review of postsurgical patients (N = 83; 56.8% female) in the Transitional Pain Service (TPS) at Toronto General Hospital, who were seen 1 month postsurgery (M = 32.11 days). After REB approval and informed consent, patients completed the following self-report questionnaires on an electronic platform (Manage My Pain, ManagingLife, Inc.) before their initial TPS visit: pain intensity and interference, psychological responses to pain, and psychological functioning. Internal consistency and concurrent validity were examined using Cronbach’s alpha and correlations.

**Results**: Internal consistency of the PRS total score (α = .92) and two subscales (α = .90 and .93) were excellent. PRS scores were negatively associated with current pain (r = −.31), pain interference (r = −.39), depression (r = −.38), anxiety (r = −.27), pain catastrophizing (r = −.56), and sensitivity to pain traumatization (r = −.49), all ps < .03. PRS scores were positively associated with quality of life (r = .27, p = .031).

**Discussion/Conclusions**: The PRS is a reliable and valid measure of pain resilience among complex postsurgical patients. Longitudinal research should examine if the PRS scores predict pain-related outcomes postoperatively and assess resilience as a modifiable treatment target in postsurgical interventions.

## Exploratory Analysis of Postoperative Outcomes for Children with Autism Following Dental Surgery

Rabia Salman^1^, Julianna Valstar^1^, Christopher Durr^1^, Thierry Lamvohee^1^, Jillian Miller^1^, Tiffany Rice^1^

^1^University of Calgary

**Introduction**: Surgical experiences may be particularly challenging for children with autism spectrum disorder (ASD) due to exposures to new sights, sounds, and people. Pain, fear, and nausea may be experienced postoperatively. Both parent and child preoperative anxiety has been associated with worse postoperative outcomes in non-ASD children. There is minimal information available regarding the perioperative experiences of children with ASD.

**Methods**: In all, 69 children (16 with ASD, 53 without ASD) between the ages of 2 and 13 years requiring dental restoration surgery under general anesthesia were included. Preoperative anxiety was assessed during induction using the modified Yale preoperative anxiety scale. Parents reported on their child’s postoperative pain, fear and nausea using facial rating scales on days 1, 3, 7, and 14. Mean comparisons were applied to examine differences in postoperative outcomes between groups. Linear regression was used to examine the relationship between preoperative anxiety and postoperative outcomes between groups.

**Results**: Just 14 days following surgery, children with ASD (n = 15) had significantly more pain (P = .02) and nausea (P = .01), but not fear compared to children without ASD (n = 44). Greater preoperative anxiety was associated with decreased pain and nausea in children with ASD (n = 9) at 14 days post-surgery (P = .03, R^2^ = 0.85), but not in children without ASD (n = 35).

**Discussion/Conclusions**: Preliminary analysis of the data revealed that greater preoperative anxiety was associated with less postoperative pain and nausea in children with ASD. Data collection for this study is still underway. More research is needed to understand the differences in postoperative outcomes between children with and without ASD.

## Daily Determinants of Cannabis Craving and Cannabis Intake Among Persons With Chronic Pain: Preliminary Insights from an Ecological Momentary Assessment (EMA) Study

Amanda Sirois^1, 2^, Lucas Frankel^3^, Gabriella Spiegler^4^, Jiaqi Bi^5^, Daniel Rosenthal^6^, Maria Verner^2^, Abdulelah Binshihah^2^, Jonathan Hudon^2, 7, 8, 9, 10^, Maayan Ben-Sasson^2^, M. Gabrielle Pagé^2, 11^, Jordi Perez^2, 7^, Mark Ware^2, 3, 8^, Mary-Ann Fitzcharles^2, 12^, Marc O. Martel^1, 2, 7^

^1^Faculty of Dental Medicine and Oral Health Sciences, McGill University, ^2^Alan Edwards Pain Management Unit, McGill University Health Center, ^3^Department of Psychology, McGill University, ^4^Department of Epidemiology, Biostatistics, and Occupational Health, McGill University, ^5^Department of Epidemiology and Biostatistics, Schulich School of Medicine & Dentistry, Western University, ^6^Department of Psychology, Concordia University, ^7^Department of Anesthesia, McGill University, ^8^Department of Family Medicine, McGill University, ^9^Edwards Family Interdisciplinary Center for Complex Pain, Montréal Children’s Hospital, ^10^Division of Supportive and Palliative Care, Jewish General Hospital, ^11^Department of Anesthesiology and Pain Medicine, Université de Montréal, ^12^Division of Rheumatology, McGill University

**Introduction**: Pain relief is the most common reason for using cannabis among patients with chronic pain. The “relaxing” or “calming” effects of cannabis are also commonly reported reasons for using cannabis. To date, the bulk of work that examined the factors contributing to cannabis use in patients with chronic pain was based on cross-sectional (survey) studies. Although useful, these studies were not suited to examine factors contributing to patients’ desires (i.e., cravings) to use cannabis and day-to-day cannabis use patterns. Objectives: To examine the contribution of daily pain intensity and anxiety to cannabis craving. We also examined if the relaxing effects of cannabis contributed to cannabis craving, and the degree to which these factors contributed to the use of THC and CBD products.

**Methods**: In this ecological momentary assessment (EMA) study, patients (n = 95) with chronic pain using cannabis completed electronic diaries, multiple times daily, for 10 consecutive days. Diaries assessed a host of pain, psychological, and cannabis-related variables.

**Results**: Multilevel analyses indicated that intraday elevations in pain intensity and anxiety were associated with heightened cannabis craving (both p’s < .05). Greater relaxing effects of cannabis were also associated with craving (p < .05). Among THC-dominant users, cannabis intake was preceded by significant elevations in pain and craving (both p’s < .05). Among CBD-dominant users, cannabis intake was preceded by significant elevations in pain (p < .05).

**Discussion/Conclusions**: Our findings provide new insights into the factors contributing to cannabis craving and day-to-day cannabis use among patients with chronic pain.

## Living with HIV in Canada: Results of a National Online Bilingual Survey on Chronic Pain Experiences

Sarmitha Sivakumaran^1^, Manon Choinière^2^, Kath Webster^3^, Guy-Henri Godin^4^, Madeleine Durand^5^, Michael Parsons^6^, Claudette Cardinal^7^, Darren Lauscher^4^, M. Gabrielle Pagé^2^, Éric Fortin^8^, Alice Tseng^9^, Kelly O’Brien^10^, Branka Vulesevic^11^, Kieran Cooley^12^, Denise Kreutzwiser^13^, Jaris Swidrovich^14^, Anaïs Lacasse^15^, Kathleen Rice^16^, Jaime Vera^17^, Colleen Price^4^, Francisco Ibáñez-Carrasco^1^

^1^Dalla Lana School of Public Health, University of Toronto, Toronto, Ontario, Canada, ^2^Department of Anesthesiology and Pain Medicine, Université de Montréal, Montreal, Quebec, Canada, ^3^Victoria Persons with AIDS Society, Victoria, British Columbia, Canada, ^4^Community Advisory Committee, CIHR Canadian HIV Trials Network, ^5^Research Center, Center hospitalier de l’Université de Montréal, Montreal, Quebec, Canada, ^6^Dalhousie University, Halifax, Nova Scotia, Canada, ^7^BC Center for Excellence in HIV/AIDS, Epidemiology and Population Health Program, Vancouver, British Columbia, Canada, ^8^COCQ-SIDA, Montreal, Quebec, Canada, ^9^HIV Pharmacotherapy Specialist, Immunodeficiency Clinic, Toronto General Hospital, Toronto, Ontario, Canada, ^10^Department of Physical Therapy, Temerty Faculty of Medicine, University of Toronto, Toronto, Ontario, Canada, ^11^Department of Medicine, Division of Infectious Diseases and Chronic Viral Illness Services, McGill University Health Center, Montreal, Quebec, Canada, ^12^Canadian College of Naturopathic Medicine, Toronto, Ontario, Canada, ^13^St. Joseph’s Health Care London, London, Ontario, Canada, ^14^Leslie Dan Faculty of Pharmacy, University of Toronto, Toronto, Ontario, Canada, ^15^Université du Québec en Abitibi- Témiscamingue, Rouyn-Noranda, Quebec, Canada, ^16^Department of Family Medicine, McGill University, Montreal, Quebec, Canada, ^17^Brighton and Sussex Medical School, Brighton, United Kingdom

**Introduction**: Pain is a common co-occurring condition among the 60,000 individuals living with HIV/AIDS (PLHAs) in Canada, arising from both the HIV infection and the side effects of antiretroviral therapy. It affects physical functioning, sleep, cognition, emotional well-being, social participation, and quality of life. Chronic pain, defined by the International Association for the Study of Pain (2020), persists for over three months. This study involved peer researchers (PRs), clinicians, and educators.

**Methods**: The study employed an exploratory sequential mixed-methods design, starting with a bilingual online survey, followed by Q-sorting workshops to prioritize key statements. We aimed to recruit up to 500 participants who self-identified with chronic pain and consented to complete a self-report questionnaire. The survey included questions regarding chronic pain experiences, healthcare access, and the social determinants affecting the well-being of PLHAs. Qualitative data was analyzed using thematic analysis.

**Results**: Preliminary qualitative analysis with PRs revealed: a) PLHAs may not identify their experiences as chronic pain; b) participants identify systemic issues related to pharmacy prescriptions and barriers to mobility across regions; and c) individuals report their pain as neglected and inadequately managed by healthcare providers, leading them to adopt maladaptive coping strategies.

**Discussion/Conclusions**: This study will improve understanding of chronic pain in PLHAs, highlighting the social determinants of health, access to care, and the impact of pain.

Findings may inform interventions, policies to improve pain management, and future research.

## Estimating the Effects of Social Determinants of Pain in Injured Ontario Workers

Saghar Soltanabadi^1^, Mohammad Bayattork^1^, David Walton^1^

^1^Western University

**Introduction**: Chronic pain following workplace injuries represents considerable cost for employers, insurers, and the workers themselves. Although prior research has found that acute pain intensity and pain-related distress can predict risk of chronic pain, unclear is how risk stratification tools are differentially influenced by intersectional identities like age, sex, education, and racial identity.

**Methods**: Cross-sectional and longitudinal analyses of data collected from acutely injured workers in Ontario. Pain intensity (Numeric Pain Rating Scale) and post-traumatic distress (Traumatic Injuries Distress Scale) were collected alongside information on intersectional identities of age, sex, education, income, experiences of treatment as a person of color, and intersectional discrimination experiences. Rehabilitation outcomes of pain, self-rated disability, and work status were collected after 4 and 8 weeks of rehabilitation care. Bivariate and multivariate regression analyses were used to identify the significant identity and social experience variables that contribute unique significant variance to the NPRS and TIDS tools.

**Results**: Of n = 170, cross-sectional regression indicated that being treated as a person of color and older age explained significant (17.8%) variance in NPRS scores. Education moderated the association between region of injury (spine vs. extremities) and pain. Only prior experiences of intersectional discrimination explained significant (11.2%) variance in TIDS scores. Both TIDS and NPRS differentially predicted 4- and 8-week rehabilitation outcomes.

**Discussion/Conclusions**: Common tools for screening risk of chronic pain functioned as expected in injured workers. However, associations with intersectional identity indicate that cut-scores for determining risk should be adapted based on the worker completing the forms.

## Exploratory Analysis of Placebo Responses in LGBTQ2S+ Youth

Lucy Stuckey^1^, Anita Szabo^2^, Adam Kirton^2^, Helen Carlson^2^, Melanie Noel^2^, Serena Orr^2^, Nivez Rasic^1^, Frank MacMaster^3^, Katie Birnie^2^, Lindsay Craddock^2^, Jillian Miller^2^

^1^University of Alberta, ^2^University of Calgary, ^3^Dalhousie University

**Introduction**: LGBTQ2S+ youth are at higher risk of experiencing trauma compared to heterosexual/cisgender peers. How experiences with trauma impact responses to pain treatments, particularly among LGBTQ2S+, is unknown. We explored how sexuality and trauma interacted with placebo responses among youth.

**Methods**: 17 youth aged 14–18 years self-reported their gender, sexuality (5 LGBTQ2S+ and 12 non-LGBTQ2S+), and number of adverse childhood experiences, and underwent MRI scanning and pain testing. A 3 × 3 grid was drawn on the participants’ forearm. One inert cream was dyed three different colors, and one color was applied to each row of the grid. Participants were made to believe that the pink cream induced hyperalgesia, the blue cream induced analgesia, and the yellow cream had no effect. While undergoing functional MRI, heat pain was applied to the grid and pain intensity was recorded. Brain responses and pain intensity was compared between LGBTQ2S+ and non-LGBTQ2S+ youth with higher and lower traumatic experiences.

**Results**: LGBTQ2S+ youth reported greater trauma compared to non-LGBTQ2S+ youth (P < .001). There was a significant interaction between sexuality and medial prefrontal cortical activity after controlling for trauma, such that greater medial prefrontal cortical activity was significantly associated with a reduced placebo response for LGBTQ2S+ individuals (P < .05), but not non-LGBTQ2S+.

**Discussion/Conclusions**: In an exploratory analysis LGBTQ2S+ youth appeared less responsive to placebo compared to heterosexual/cisgender peers. It is possible that medical mistrust among LGBTQ2S+ members led to reduced placebo responses. More research is needed to determine the relationships between trauma, sexuality, and pain treatment in youth.

## Content on Instagram Regarding Pain with Intrauterine Device Insertion: A Quality Assessment

Aprill Susin^1^, Monakshi Sawhney^1^

^1^Queens University

**Introduction**: Instagram is a social media platform which allows individuals to share stories, personal videos, and pictures with other Instagram users. Many patients turn to friends, family, and social media for contraceptive advice instead of a health care professional. Fear of pain during IUD insertion is one barrier to women choosing this form of contraception, which could be influenced by social media. In Canada, 50% of the population has an Instagram account and spends on average 9 hours a month on the app. Studies examining user content regarding Intrauterine devices (IUDs) and pain have been conducted using the YouTube, X (Twitter), and TikTok platforms. To date there have been no studies exploring the same content on Instagram. Objectives: To understand the content being shared regarding the experience of pain during IUD insertion on the social media platform Instagram.

**Methods**: The first 100 posts related to intrauterine device insertions and pain written in English were reviewed. Two researchers watched each video; categorizing them as negative, positive, or informative. Informative posts were further divided into health professional and recipient.

**Results**: A total of 1,214 Instagram posts were reviewed, with 100 posts meeting the inclusion and exclusion criteria. Thirty-eight percent were categorized as negative, 12% positive, 50% were informative posts. Out of all the informative posts, 85.5% were from health professionals and 14.5% was from recipients. 80.4% of the informative posts contained accurate information.

## An Exploration of the Increasing Prevalence of Chronic Pain among Canadian Veterans: Life After Service Studies 2016 and 2019

Jhalok Talukdara^1^, Dena Zeraatkara^1^, Andrew Thomas^2^, Jason Busse^1^

^1^McMaster University, ^2^Canadian Armed Forces Health Services Center

**Introduction**: The Life After Service Study (LASS) suggests the absolute prevalence of chronic pain among Canadian Veterans increased by 10% from 2016 to 2019. We explored the association of year of survey administration, sociodemographic, military, and health-related factors, with the prevalence of chronic pain among Canadian Veterans.

**Methods**: We analyzed 2016 and 2019 LASS data and built a multivariable regression model to explore factors associated with chronic pain. Measures of association are reported as adjusted odds ratios (ORs) and absolute risk increases (ARIs).

**Results**: The 2016 LASS (73% response rate; 3,002 of 4,121) reported a 41.4% prevalence of chronic pain, and the 2019 LASS (72% response rate; 2,630 of 3,671) reported a 51.5% prevalence of chronic pain among Canadian Veterans. Respondents who completed the 2019 LASS were more likely to endorse anxiety, depression, post-traumatic stress disorder, traumatic brain injury, and mood disorder. In our adjusted regression model, year of survey administration was not associated with chronic pain (OR 1.08, p = .8); however, we found large associations with obesity class 1 (BMI 30.0–34.9) (OR 3.66, 95%CI 1.46 to 9.17; ARI 27%), obesity class 2 (BMI 35.0–39.9) (OR 8.10, 95%CI 1.67 to 39.3; ARI 47%), depression (OR 3.20, 95%CI 1.49 to 6.88; ARI 24%) and anxiety (OR 4.53, 95%CI 1.28 to 16.0; ARI 33%).

**Discussion/Conclusions**: The increase in chronic pain among Canadian Veterans from 2016 to 2019 appears confounded by increased co-morbid mental illnesses associated with chronic pain among responders in 2019.

## Project ECHO: A Model of Care Provider Education to Support Pain Management During The Peripartum Period

Elaine Teh^1, 2^, Lucy Doan^1^, David Flamer^2, 3^, Matthew Shepperd^2, 3^, Erin Lurie^4, 5^, Virginia Fernandes^2, 6^, Rebecca Titman^2, 7^

^1^Lawrence S. Bloomberg Faculty of Nursing, University of Toronto, ^2^Sinai Health, ^3^University of Toronto Department of Anesthesia and Pain Management, ^4^University of Toronto Department of Community and Family Medicine, ^5^Unity Health, ^6^Leslie Dan Faculty of Pharmacy, University of Toronto, Toronto, ^7^University of Toronto Temerty Faculty of Medicine

**Introduction**: Managing pain during the peripartum period is challenging due to limitations in pharmacotherapy, sparse research and a lack of educational resources for prenatal care providers. The Extension for Community Healthcare Outcomes (ECHO) model™ is a virtual network that increases the capacity of community care providers to manage complex cases by providing education and connecting with a specialist team. The objective of this pilot project was to adapt the Project ECHO model to a Pain in Pregnancy curriculum.

**Methods**: A specialist interprofessional group was formed to develop a tailored curriculum consisting of 8 weekly 1-hour virtual sessions focused on key peripartum topics. Each session included a didactic teaching component along with case-based discussion. As a pilot project, data was collected on demographics of registrants, attendance, and feedback from participants to adapt future iterations.

**Results**: Forty-seven individuals registered for the program across Canada, including physicians, nurses, allied health and pharmacists. Attendance was variable weekly, ranging from 19–74% of registrants. Feedback from participants indicated the combination of didactic teaching, case-based discussion and access to specialists in real time was beneficial to their learning. The resources provided were an additional asset. Registrants noted a preference to have access to recorded sessions in the future.

**Discussion/Conclusions**: The ECHO model of education has the potential to be an effective way to empower prenatal care providers and pain practitioners. Future iterations of the program will focus on enhancing active participation, ensuring consistent attendance and compiling resources for ongoing reference.

## Psychometric Evaluation and Validity of the Multidimensional Pain Treatment Planning Questionnaire with a Clinical Sample of Patients Attending Bleeding Disorder Programs in Western Canada

Taylor Teckchandani^1^, Lucas Horvath^2^, Kirstian Gibson^2^, Kelsey Brose^3, 4^, Michael Szafron^2^, Susan Tupper^2, 4^

^1^University of Regina, ^2^University of Saskatchewan, ^3^Saskatchewan Cancer Agency, ^4^Saskatchewan Health Authority

**Introduction**: Pain is common among people living with bleeding disorders. Approximately 60% of adults living with hemophilia report daily pain. To be effective, pain management must be tailored to underlying pain mechanisms and patient capacities. The 28-item Pain Treatment Planning Questionnaire (PTPQ) can guide shared decision-making about pain management for patients attending bleeding disorder clinical programs. Further research is needed to examine psychometric properties of the PTPQ in relation to established pain and coping measures.

**Methods**: Overall, 131 participants were recruited from three bleeding disorder clinics in Saskatchewan, Alberta, and British Columbia to complete the PTPQ, PainDETECT (PD), Central Sensitization Inventory (CSI), and Hemophilia Pain Coping Questionnaire (HPCQ). We examined Cronbach’s alpha (internal consistency), classification accuracy (concurrent validity), and bivariate correlation models of PTPQ numerically graded subscales with PD, CSI, and HPCQ (convergent validity).

**Results**: Measures demonstrated excellent internal consistency (Cronbach’s alpha = 0.76 to 0.92). PTPQ Intensity subscale demonstrated excellent discriminative capacity to detect probable neuropathic pain (ROC = 0.96). PTPQ Intensity and Frequency subscales showed statistically significantly positive associations with PD total scores (r = 0.41 to 0.64), CSI total scores (r = 0.49 to 0.58), and HPCQ subscales (r = 0.22 to 0.46) indicating acceptable convergent validity. PTPQ Treatment Satisfaction and Efficacy subscales showed statistically significantly negative associations with PD total scores (r = −0.44, – 0.45), CSI total scores (r = −0.33, −0.55), and HPCQ Negative Thoughts and Passive Adherence subscales (r = −0.20, −0.46).

**Discussion/Conclusions**: The PTPQ demonstrates excellent internal consistency, concurrent and convergent validity with measures of neuropathic pain, central sensitivity, and pain coping in a Western Canadian bleeding disorder population.

## Canadian Veterans’ Experiences of Living with Chronic Pain: A Descriptive Qualitative Study

Moizza Ul Haq^1^, Vahid Ashoorion^1^, Cheng Xi^2^, Eileen Wang^1^, Natasha Ross^1^, Nandana Parakh^1^, Jason Busse^1^, Andrea Darzi^1^, Elizabeth Alvarez^1^

^1^McMaster, ^2^University of Ottawa

**Introduction**: An estimated 30% of veterans live with chronic pain, compared to 20% of Canadians in the general population. Veterans face health care challenges upon release from the military, increasing difficulties in obtaining chronic pain care. We explored experiences of Canadian Armed Forces veterans living with chronic pain, their transition from military to civilian care, perceived barriers and facilitators to chronic pain care, and impacts of their pain on the domains of well-being.

**Methods**: We conducted a qualitative descriptive study using semi structured interviews. We used a deductive/inductive approach to derive themes and concepts from interview transcripts.

**Results**: Thirty-five veterans living with chronic pain participated. Participants reported that pain affected their lives in numerous ways, including negatively impacting relationships and limiting activities of daily living and leisure. They identified barriers to care, including lack of access to family doctors or health care services, reluctance to ask for help, and challenges in obtaining coverage for services from Veterans Affairs Canada. Facilitators included support from other veterans and online resources. Chronic pain had bidirectional effects on domains of well-being.

**Discussion/Conclusion**: Experiences of pain varied among Canadian veterans, and military culture played a role in perceptions and management of pain. Barriers and facilitators to chronic pain care were highlighted from their time in the military into their transition to civilian care. Participants described the impact of chronic pain on their overall well-being. Determining whether these findings are relevant to a larger population of Canadian veterans will be important for future research.

## Comorbid Chronic Pain and PTSD in Women Canadian Armed Forces Members and Veterans: A Qualitative Inquiry

Gina Vaillancourt^1^, Larah Maunder^1^, Rosemary Wilson^2^, Tim Salomons^1^

^1^Department of Psychology, Queen’s University, ^2^School of Nursing, Queen’s University

**Introduction**: Chronic pain and posttraumatic stress disorder (PTSD) are highly prevalent among service members and Veterans. Chronic pain and PTSD frequently co-occur in military populations, creating a unique clinical interaction that complicates treatment. Evidence shows that women service members and Veterans are more likely to experience comorbid chronic pain and PTSD than their men counterparts. There is a particular need to examine issues (e.g., sexual trauma, social support) that affect military women more acutely that may be uniquely affecting their experience of comorbid chronic pain and PTSD.

**Methods**: Nineteen Canadian Armed Forces service members and Veterans participated in 1- to 2-hour semi-structured interviews about their experiences of comorbid PTSD and chronic pain. This study selected the transcripts of women service members and Veterans (n = 6) and applied interpretive description, a qualitative methodology that aims to produce interpretive insights that enhance clinical understanding. Thematic analysis was used to extract exemplars of interest to identify themes within the data.

**Results**: Preliminary analyses identified four themes. The first theme described mutual maintenance factors between the conditions, and the second captured how participants perceived trauma as existing within their tissues, which manifests physically as chronic pain. The third theme described participants’ struggles with accepting pain and trauma, while the fourth illustrated feelings of betrayal and failure in military and personal contexts.

**Discussion/Conclusions**: By capturing lived experiences, this study provides crucial insight into women Veterans’ unique challenges with chronic pain and PTSD, and offers perspectives to inform integrated interventions that target both conditions.

## Do Nerve Resection, Amputation or Immunotherapy Relieve Causalgia in the Long Term?

Peter Watson^1^

^1^University of Toronto

**Introduction**: We report here the extended follow-up (20 years, 14 years) of two patients with longstanding causalgia initially successfully treated by nerve resection and reported in detail previously. Reports of this duration for this surgical treatment of causalgia are not available.

**Methods**: Two causalgia patients treated by nerve resection were followed yearly for 20 and 15 years.

**Results**: One of these had uncomplicated causalgia (not CRPS) after a traumatic infra-orbital nerve injury and orbital fracture. This pain was relieved by nerve resection with no recurrence over 20 years to date (January 2025).

The other patient had very longstanding and intractable CRPS type 2 after a left leg injury in childhood. This latter patient had 4 years and 4 months relief by nerve resection. At this time pain recurrence and gangrene of the left leg required amputation which saved the proximal leg but did not affect the pain, or a generalized severe skin rash or frequent respiratory infection. The pathology of the amputated limb suggested an autoimmune response and further tests found evidence of autoimmune antibodies and immunoglobulin deficiency. Only after immunotherapy did these problems resolve and this marked improvement has persisted for 5 years and 9 months to date (January 2025).

**Discussion/Conclusions**: There are few if any very long-term assessments of causalgia treated by nerve resection, amputation and immunotherapy. Some short term failures of these treatments for CRPS may be due to an underlying unrecognized and treatable condition such as autoimmunity and immune deficiency.

## Alcohol Use is Associated with Slower Pain Resolution After Surgery – A Retrospective Study

Callon M. Williams^1^, Matthanja Bieze^1^, Stuart A. McCluskey^1^, Hance Clarke^1^, P. Maxwell Slepian^1^

^1^University Health Network

**Introduction**: Pain and alcohol use frequently co-occur. Alcohol use is recognized as a modifiable risk factor for postoperative complications and the transition from acute to chronic pain. This study examined the effects of alcohol use on pain trajectories the first week after surgery.

**Methods**: A retrospective chart review of 12,153 adult surgical patients (48.6% female) who underwent surgery at the University Health Network. Alcohol use was measured in fluid ounces per week. Daily average pain at rest and with activity were measured over the first seven days postoperatively. Separate linear mixed models were used to examine effects of alcohol on pain among those who endorsed alcohol use.

**Results**: Of those asked preoperatively, 1 in 3 individuals reported current drinking (39.3%, n = 2,894). Controlling for sex, age, and number of surgeries, mixed models showed a significant effect of time, such that, on average, pain ratings decreased the first 7 days postoperatively (rest: b = −.47; activity: b = −.49; ps < .001). Results also showed a significant interaction between time and alcohol use (rest: b = .02, p = .021; activity: b = .03, p = .031). Tests of simple slopes indicated pain resolved more slowly at higher amounts of alcohol use, such that pain did not reduce during hospitalization for individuals with high levels of drinking.

**Discussion/Conclusions**: Alcohol use is associated with slower resolution of pain among surgical patients. Clinical efforts should be made to educate patients on the negative impacts of alcohol use on pain and surgical outcomes.

## Associations Between Pain Severity and Substance Use Among Individuals Diagnosed with Ehlers-Danlos Syndrome (EDS) or Generalized Hypermobility Spectrum Disorder (G- HSD)

Callon M. Williams^1^, Molly McCarthy^1^, Rachel Siegal^1^, Michelle Flynn^1^, Stephanie Buryk- Iggers^1^, Daniel Santa Mina^1^, Dmitry Rozenberg^1^, Tania Di Renna^1^, Max Rachinsky^1^, Praveen Ganty^1^, Joel Katz^1^, Laura McGillis^1^, Nimish Mittal^1^, Hance Clarke^1^, P. Maxwell Slepian^1^

^1^University Health Network

**Introduction**: Pain and substance use frequently co-occur. Research indicates pain motivates alcohol, cannabis, and tobacco use. Pain is also highly co-morbid with Ehlers- Danlos Syndromes (EDS) and Generalized Hypermobility Spectrum Disorder (G-HSD), yet there is little research examining associations between pain and substance use in these populations.

**Methods**: A retrospective chart review of 533 patients (88.9% female) seen at the GoodHope EDS Clinic at Toronto General Hospital and diagnosed with EDS/G-HSD. Patients provided self-report data on pain and substance use behaviors before their initial appointment. Analyses included descriptive statistics and bivariate correlations.

**Results**: Over half of patients reported moderate to severe pain (55.0%; >4/10 on BPI- Severity). Alcohol use was reported by 58.5% of patients, with an average of 2.47 drinks consumed per week. Almost half (48.2%) reported cannabis use, with most using four or more times per week (68.6%) and half reporting multiple administration methods (47.4%). Patients who use tobacco (14.3%) reported smoking, on average, half a pack per day (M = 9.74 cigarettes). Pain severity was positively correlated with cannabis use frequency (rs = .23), quantity (rs = .20), and tobacco use (rs = .14), and negatively correlated with alcohol consumption (rs = −.20), all ps < .05.

**Discussions/Conclusions**: Longitudinal research is needed to determine if current findings reflect pain as a motivator for cannabis use or if persistent cannabis use exacerbates pain in this population. Research should also examine whether lower alcohol consumption reflects alcohol-induced analgesia or avoidance of drinking due to exacerbation of other EDS/G-HSD conditions (e.g., dysautonomia).

## Treatment/management/pain programsExploring Factors Associated with Patient-Perceived Success in Chronic Pain Management

Sana Alibhai^1^, Eleni Hapidou^2^, Jennifer Anthonypillai^3, 4^

^1^Faculty of Health Sciences, McMaster University, ^2^Michael G. DeGroote Pain Clinic, Hamilton Health Sciences; Department of Psychiatry and Behavioral Neurosciences; Department of Psychology, Neuroscience & Behavior, McMaster University, ^3^Hamilton Health Sciences, ^4^Michael G. DeGroote Pain Clinic

**Introduction**: The effectiveness of interdisciplinary chronic pain management programs (ICPMP) is well documented, however, identifying factors that differentiate successful patients from less successful ones remains underexplored. Previous literature highlights the importance of cognitive-behavioral factors such as acceptance and coping for patient success. The purpose of this study was to investigate factors associated with patients’ self-evaluated goal accomplishment following attendance of an ICPMP.

**Methods**: Participants (n = 81, 59 male, 56 Veteran) completed a 5-week hospital-based ICPMP. Questionnaire packages at admission and discharge assessed pain intensity, pain- related disability, emotional distress (e.g., depression, anxiety, catastrophizing), kinesiophobia, sensitivity to pain traumatization, readiness to change, and acceptance of pain. Participants were categorized into three groups based on self-evaluated goal accomplishment at discharge: “poorly to fairly,” “well,” and “very well to excellent.” Mixed ANOVAs compared magnitude of change on outcome measures in the three groups.

**Results**: Analyses revealed highly significant improvements from admission to discharge in the above outcome measures (p < .001). Significant between-group differences were observed at discharge, with the “very well to excellent” group demonstrating the greatest improvement in most outcomes. Changes in outcome measures at discharge were not mediated by demographic factors such as age, sex, education, and pain duration. Case manager evaluations also aligned with patient self-ratings, showing better physical, emotional/mental, and social benefits in the “very well to excellent” patient group.

**Discussion/Conclusions**: This study highlights outcomes associated with above-average patient success in ICPMP. We can use this information to further tailor pain management programs for optimal patient success.

## Influence of Positive and Negative Psychological Factors on Patient Reported Pain and Function/Disability in Patients with Upper Extremity Musculoskeletal Disorders

Joline Attalla^1, 2, 3^, Joy MacDermid^1, 2, 3^, Ruby Grewal^1, 2, 3^, Mike Szekeres^1, 3^

^1^Western University, ^2^Roth | McFarlane Hand and Upper Limb Center (HULC), ^3^Lawson Research Institute

**Introduction**: Multiple psychological factors have been proposed to influence pain and disability in musculoskeletal disorders. The overall objective of this paper was to better understand the relative importance of positive and negative psychological factors as determinants of pain and function/disability, reported by patients presenting for care of upper extremity musculoskeletal problems

**Methods**: This was a single-center retrospective cohort design of The Hand and Upper Limb Center (HULC), that provides specialty care to patients with a variety of upper limb extremity disorders. The core outcomes of interest extracted include patient demographics, pain (1 of 3 joint specific PROM) and function/disability (SANE for all patients and 1 of 3 joint specific PROM). All outcome measures were expressed on a common metric of 0–100%. A Pearson correlation analysis and a standard and backwards multivariate linear regression analysis was conducted on SPSS.

**Results**: The association between individual psychological factors and pain was low to moderate (0.07–0.54), and negligible to low with the SANE (−0.04 to −0.28), and a joint specific disability PROM (0.03–0.43) for overall patients.

Baseline Prognostic Negative Sub-scale scores and Baseline Prognostic Positive Sub-scale scores were significantly correlated to Upper Extremity Pain, the SANE, and Upper Extremity Disability.

Our linear regression highlighted that sex (R^2^ = 0.26) had the largest effect on Upper Extremity Pain, while age had the largest effect on Upper Extremity Function (R^2^ = 0.21).

**Discussion/Conclusions**: Patients Negative and Positive Prognostic Sub-scale scores are associated with Upper Extremity Pain, the SANE, and Upper Extremity Disability scales.

## Development and Usability of a Scalable Digital Acceptance and Commitment Therapy Intervention for Chronic Postsurgical Pain

Kristina Axenova^1, 2^, Anna Lomanowska^1, 3^, Tahir Janmohamed^4^, Molly McCarthy^1, 3^, Joel Katz^1, 2, 3, 4, 5^, Hance Clarke^1, 3, 5^, Maxwell Slepian^1, 3, 5^

^1^Transitional Pain Service, Toronto General Hospital, University Health Network, ^2^Faculty of Health, York University, ^3^Department of Anesthesia and Pain Management, University Health Network, ^4^ManagingLife, ^5^Department of Anesthesiology and Pain Medicine, University of Toronto

**Introduction**: The Transitional Pain Service (TPS) at Toronto General Hospital is a multidisciplinary program focused on preventing chronic postsurgical pain (CPSP) and reducing persistent opioid use. While the program is effective in terms of patient outcomes, the scarcity of specialized pain psychologists limits its broader implementation. Acceptance and Commitment Therapy (ACT) is an evidence-based psychology intervention used at the TPS. To increase access to this intervention, we developed a digital self-guided program, Advancing Online Psychology Tools for the Transitional Pain Service (ADOPT-TPS).

**Methods**: The ADOPT-TPS intervention was modeled after a 3-hour ACT group workshop at the TPS. It includes psychoeducation on ACT and pain that revolves around the ACT Matrix, a tool for evaluating coping strategies and enhancing quality of life. The intervention was developed on the digital health application, Manage My Pain (MMP), as an interactive, self-paced program. Development followed an iterative process, with qualitative stakeholder feedback from TPS patients, clinicians, research staff and user experience designers gathered throughout.

**Results**: The online intervention was implemented as four sequential 15-min parts. Each part includes didactic modules interspersed with interactive activities, experiential exercises, and optional learning checks. The MMP platform’s existing navigation features helped streamline development. Feedback from stakeholder “think-aloud” and retrospective interviews helped refine key usability factors, including engagement, navigation, and content clarity.

**Discussion/Conclusions**: The online ADOPT-TPS intervention is scalable and can be easily implemented at clinics without specialized pain psychologists. Further testing through a randomized controlled trial will assess its effectiveness as a substitute for psychologist- led group workshops.

## A Qualitative Analysis of the Recovery Trajectory Following a Pre-Operative Rehabilitation Program For Symptomatic Lumbar Spinal Stenosis Surgery

Nora Bakaa^1^, Luciana Macedo^2^, Lisa Carlesso^2^, Douglas Gross^3^, Joy MacDermid^4^, Alison Rushton^4^, Maxi Miciak^3^

^1^OntarioTech University, ^2^McMaster University, ^3^University of Alberta, ^4^Universty of Western Ontario

**Introduction**: We aimed to understand psychosocial experiences with physical activity and exercise in the recovery trajectory of patients undergoing surgery following a prehabilitation intervention for symptomatic lumbar spinal stenosis.

**Methods**: We conducted a longitudinal qualitative study using interpretive descriptive methodology.

**Results**: We identified four trajectories (n = 10), including 1) Sedentary struggle, 2) Dynamic recovery, 3) Dynamic struggle, and 4) Dynamic resilience. The sedentary struggle trajectory was characterized by low physical activity, negative attitudes and behaviors toward pain and exercise at baseline. During the intervention, they struggled to appreciate the benefits of exercise and described slower recovery following surgery.

The dynamic recovery trajectory had low engagement with physical activity and negative attitudes and behaviors. Participants experienced changes in attitudes and behaviors during the intervention and a faster recovery. The dynamic resilience trajectory was characterized by high engagement in physical activity and positive attitudes and behaviors toward pain and exercise. They experienced further changes in their behaviors and faster recovery. The same factors characterized the dynamic struggle trajectory as the dynamic resilience one; however, participants experienced postoperative complications, leading to a regression in attitudes and behaviors and slow recovery.

**Discussion/Conclusions**: A biopsychosocial approach aiming to alter attitudes and behaviors to enable exercise and physical activity may help improve postoperative outcomes in this population.

## Prioritizing Nursing Interventions for Chronic Pain Management in Primary Care: Insights from a Delphi Study

Andréanne Bernier, inf, PhD student^1^, Marie-Eve Poitras, inf, PhD^2^, Marie-Dominique Poirier, patient partner^2^, Sylvie Beaudoin, patient partner^1^, Anaïs Lacasse, PhD^1^

^1^Université du Québec en Abitibi-Témiscamingue, ^2^Université de Sherbrooke

**Introduction**: Despite chronic pain (CP) frequency (27% of Canadians), nurses involvement in its management remains scarce in primary care. Nurses are positioned to address both the physical and psychosocial aspects of CP, yet barriers such as inadequate training and unclear guidelines limit their role in care. This study aimed to identify priority nursing activities for CP management in primary care, incorporating perspectives from both primary care nurses and patients.

**Methods**: A three-round Delphi study was conducted with 48 primary care nurses and 122 patients with CP (pain for >3 months) in Québec, Canada. In the first round, participants identified important nursing activities for CP management through open-ended questions. In the following two rounds, these activities were rated for importance using a 9-point Likert scale. Activities rated 7, 8, or 9 by ≥75% of participants were considered priority activities.

**Results**: In round one, 47 nursing activities were identified across four domains: global assessment, care management, health promotion, and interprofessional collaboration. Thanks to rounds two and three, 41 activities remained prioritized. For women, the top priorities were establishing a therapeutic alliance, assessing pain, and screening for mood disorders. For men, the top priorities were reviewing patient records, assessing pain, and evaluating the impact of pain on sleep.

**Discussion/Conclusions**: This study identifies essential nursing activities for CP management in primary care, offering a framework to optimize nursing practice and improve patient outcomes. Clinical settings can better target their efforts while advocating for the necessary resources, training, and organizational support to implement interventions effectively.

## A Pyramid Model for Guiding Therapists in Chronic Pain Management: Addressing Central Sensitization, Maladaptive Neuroplasticity and Pain Phenotype

Samuel Bournival^1^, Rodrigo Deamo Assis^1^

^1^Chronic Pain Clinic of Center Integrated Health And Social Services of Abitibi- Témiscamingue

**Introduction**: The IASP recognizes three primary types of pain: nociceptive, neuropathic, and nociplastic. While physicians are generally adept at distinguishing between these pain types for medication prescription, therapists often face challenges in accurately identifying them in a therapeutic context. Further complicating pain assessment are maladaptive neuroplasticity and central sensitization – two factors that can influence the chronic pain experience and present additional challenges for therapists. To address these complexities, we propose a structured, pyramid model aimed at guiding therapists in the effective management of chronic pain in clinical practice.

**Methods**: Our pyramid model is composed of three hierarchical levels: central sensitization (at the base of the pyramid), maladaptive neuroplasticity (middle), and type of pain (top). This structure encourages therapists to start interventions by addressing the foundational level (central sensitization) when it occurs, followed by maladaptive neuroplasticity, if present, and finally, the specific type of pain. Each level is associated with specific types of exercises and treatment prescriptions tailored to the patient’s needs at that stage.

**Results**: Since 2020, this model has been implemented in our pain clinic for patient care. By using this structured approach, we have been able to provide individualized treatment plans that support patient progress and facilitate a more efficient path to discharge. The model enables patients to receive a personalized treatment plan they can continue independently following discharge.

**Discussion/Conclusions**: For our patient population, this pyramid model has proven to be a valuable tool in guiding therapists through the rehabilitation process, enhancing treatment effectiveness and patient outcomes.

## Assessing Exercise And Rehabilitation Needs Of Adults with Ehlers-Danlos Syndromes and Generalized Hypermobility Spectrum Disorder in Canada

Stephanie Buryk-Iggers^1, 2^, Daniel Santa Mina^1, 2, 3^, Nimish Mittal^1, 2, 4^, Laura McGillis^2^, Encarna Camacho Pérez^2^, Dmitry Rozenberg^4, 5^, P. Maxwell Slepian^2, 3, 6^, Hance Clarke^2, 3, 4, 6^

^1^Department of Kinesiology and Physical Education, University of Toronto, Toronto, Ontario, Canada, ^2^GoodHope Ehlers Danlos Syndrome Clinic, Toronto General Hospital, Toronto, Ontario, Canada, ^3^Department of Anesthesia and Pain Management, University Health Network, Toronto, Ontario, Canada, ^4^Faculty of Medicine, Division of Physical Medicine and Rehabilitation, University of Toronto, Toronto, Ontario, Canada, ^5^Department of Medicine, Respirology, Toronto General Hospital Research Institute, Toronto, Ontario, Canada, ^6^Department of Anesthesiology and Pain Medicine, University of Toronto, Toronto, Ontario, Canada

**Introduction**: Exercise and rehabilitation therapy (ERT) has emerged as a potentially critical component of treatment for Ehlers-Danlos Syndromes (EDS) and conditions sharing similar physical sequalae, such as generalized hypermobility spectrum disorder (G-HSD). However, there is only a modest amount of research supporting its efficacy, despite robust benefits observed in other populations with high-musculoskeletal morbidity. It is possible that the underwhelming signal of benefit is due to a lack of research on ERT-related behaviors, preferences and goals in EDS/G-HSD, which may undermine intervention engagement. Identifying these elements is needed to better understand how to optimize ERT programming.

**Methods**: A cross-sectional survey design was used to identify the behaviors, beliefs, facilitators and barriers, preferences and goals related to ERT programming in adults with EDS/G-HSD across Canada. The online survey was developed by clinicians in the GoodHope EDS Clinic (Toronto, Canada) and patient partners, and recruitment occurred via the GoodHope EDS Clinic and Canadian EDS agencies.

**Results**: Forty-five participants completed the survey. Fifty-one percent of respondents met the Canadian physical activity guidelines, and most strongly agreed that exercise improves fitness (78%), is important for long-term management of EDS/G-HSD (69%) and is important for mental health (67%). The leading barriers to exercise were fear of injury (64%), pain (60%), and worsening of symptoms after exercise (56%). Participants most preferred ERT programming to be delivered by a health care professional (100%). The most frequently listed exercise-related goal was to improve or maintain physical capacity (47%).

**Discussion/Conclusions**: These findings inform participant-centered recommendations for future research studies and the optimization of ERT programming, toward improved intervention engagement in EDS/G-HSD.

## The Toronto General Hospital GoodHope Exercise and Rehabilitation (GEAR) program for Ehlers-Danlos Syndromes and Generalized Hypermobility Spectrum Disorder: A Program Evaluation

Stephanie Buryk-Iggers^1, 2^, Daniel Santa Mina^1, 2^, Nimish Mittal^1, 2, 3^, Laura McGillis^2^, Alina Jaglanian^1, 2^, Wing Ting Truong^2^, Alex Bulluzzo^2^, Seyedeh Hashemi^2^, Dmitry Rozenberg^2, 4^, P. Maxwell Slepian^2, 5, 6^, Hance Clarke^2, 3, 5, 6^

^1^Department of Kinesiology and Physical Education, University of Toronto, Toronto, Ontario, Canada, ^2^GoodHope Ehlers Danlos Syndrome Clinic, Toronto General Hospital, Toronto, Ontario, Canada, ^3^Faculty of Medicine, Division of Physical Medicine and Rehabilitation, University of Toronto, Toronto, Ontario, Canada, ^4^Department of Medicine, Respirology, Toronto General Hospital Research Institute, Toronto, Ontario, Canada, ^5^Department of Anesthesia and Pain Management, University Health Network, Toronto, e **With Chronic Pain**

Moustapha Gassama^1^, Mickael Curadeau^1^, Marimée Godbout-Parent^1^, Anaïs Lacasse^1^

^1^Département des sciences de la santé, Université du Québec en Abitibi-Témiscamingue, Rouyn-Noranda

**Introduction**: Disputes to obtain disability benefits in the context of chronic pain present challenges for both patients and healthcare professionals. Patients often face significant difficulties in having the legitimacy of their claims recognized. This study aimed to explore the frequency of disability benefit disputes and identify factors associated with them among persons living with chronic pain.

**Methods**: This cross-sectional analysis was conducted in a sample of 1390 adults living with pain for more than 3 months, residing in Quebec (Canada), and who completed an online questionnaire as part of the COPE Cohort study. Participants were asked: “Are you currently involved in a dispute to obtain disability benefits? For example, with your employer, insurer, Quebec’s workers’ compensation board, public no-fault auto insurance system, or victims of crime compensation progrrelated, treatment, health, and lifestyle variables were measured with validated scales and compared between participants with and without disputes using chi-square and t-tests.

**Results**: 6.4% of participants reported being currently involved in a dispute to obtain disability benefits, 90.4% were not, and 3.2% were unsure. Differences (p < .05) were found on almost all tested factors, with participants involved in disputes being younger, having shorter pain duration, worse pain outcomes, poorer physical and mental health, and greater use of medications and cannabis for pain. Proportion of women was similar between groups.

**Discussion/Conclusions**: A small proportion of participants reported being involved in disability benefit disputes, but these persons differed on several factors, highlighting the need for further investigation into the challenges of this group.

## Adaptive Mentoring Networks: Mentees’ Perceived Barriers to Managing Chronic Pain, Substance Use and Mental Health

Marielle Paule Darnley^1^, Joshua A. Rash^2^, L. Jayne Beselt^3^, Laura M. Visentin^1, 4^, Arun Radhakrishnan^1, 3^

^1^Department of Family Medicine, University of Ottawa, ^2^Department of Psychology, Memorial University of Newfoundland, ^3^Bruyère Health Research Institute, ^4^The Ottawa Hospital Academic Family Health Team

**Introduction**: Adaptive Mentorship Networks (AMNs) are a Canadian innovation to improve primary care capacity and provide compassionate, quality care for those with chronic pain (CP), mental health (MH), and substance use (SU) concerns. We sought to examine associations between perceived barriers to care, and proportion of time spent in practice on CP, MH and SU amongst mentees in AMNs across the country.

**Methods**: Survey data on time spent in practice and perceived barriers to care were collected from mentees from AMNs prior to entering the program. Hierarchical regressions were conducted with proportion of time spent managing CP, SU, and MH concerns as criterion variables. Rurality was entered into STEP1. Perceived barriers were entered into STEP2 using a STEPWISE method with entry p < .05.

**Results**: 169 providers from Atlantic Canada (n = 124) and BC (n = 45) participated. b = −0.128, t(167) = 0.042, p = .967, R^2^ = .001, accessing specialty care, b = 29.646, t(166) = 4.740, p = .000,R^2^ = .084, and insufficient referrals, b = 18.165, t(165) = 2.999, p = .003,R^2^ = .047, were associated with managing SU. b = 3.757, t(167) = 1.299, p = .196, R^2^ = .004, insufficient coverage of nonpharmacological therapies, b = 18.940, t(166) = 3.078, p = .002, R^2^ = .086, funding for alternative pharmacotherapies, b = 13.653,t(165) = 2.503,p = .013,R^2^ = .057, and lack of coordinated care, b = 11.901,t(164) = 2.194,p = .030,R^2^ = .024, were associated with managing MH. Rurality was not associated with time spent in practice across CP, SU, or MH (all p’s > .196).

**Discussion/Conclusions**: The proportion of time that one spends managing CP, MH, and SU concerns in their practice is related to their perceptions of barriers to the provision of such care. Results can guide AMNs, health system funders and decision makers to address barriers to care in different regions of the country.

## Label Accuracy of Legal Oral Cannabis Oil Products in Ontario, Canada

Amanda Doggett^1^, Allan Fein^1^, Tracey Campbell^1^, Nicola Henriquez^1^, Jason Busse^1^, James MacKillop^1^

^1^McMaster University

**Introduction**: In October 2018, Canada legalized cannabis for nonmedical use. One component of the federal system was quality control, including cannabis labeling requirements that specify the allowable variance between labeled and actual amounts of tetrahydrocannabinol (THC) and cannabidiol (CBD) in a commercial product. We examined label accuracy of cannabis products in the legal Canadian market.

**Methods**: We randomly selected 30 products that were available on the Ontario Cannabis Store website. Amounts of CBD and THC in each product were quantified using high-performance liquid chromatography. Federal cannabis regulations indicate that the allowable variability for extracts is 15% above or below the product’s labeled amount.

**Results**: Overall, 12 products (40%) were outside the variability limit for THC and 3 products (10.0%) were outside the variability limit for CBD (due to greater labeled vs laboratory-tested amounts for all but 1 product). Among 16 products that had a label amount of 2.5 mg/g THC or greater, 7 products (44%) had amounts that were lower than what was labeled by more than 15%.

**Discussion/Conclusions**: Our findings suggest that inaccurate labeling of cannabis oil products in the legal Canadian market is common, with most discrepancies due to labeling products with greater THC or CBD content than was present. Given that many medical consumers obtain products from the nonmedical market, one implication is inaccurate dosing. These findings suggest a need for greater quality control in the Canadian legal cannabis market.

## Efficacy of Cognitive Functional Therapy for Pain Intensity and Disability in Patients with Nonspecific Chronic Low Back Pain: A Randomized Sham-Controlled Trial

Mariana Romano de Lira^1^, Ney Meziat-Filho^2^, Gabriela Zuelli Martins Silva^1^, Julia Castro^3^, Jessica Fernandez^3^, Rinaldo Roberto de Jesus Guirro^1^, Roger Berg Rodrigues Pereira^4^, Thaís Cristina Chaves^1^

^1^Postgraduate Program in Rehabilitation and Functional Performance, Ribeirão Preto Medical School, University of São Paulo – USP, São Paulo, Brazil, ^2^School of Rehabilitation Science, Faculty of Health Sciences, Institute for Applied Health Sciences, McMaster University, Hamilton, Ontario, Canada, ^3^Postgraduate Program in Rehabilitation Sciences, Augusto Motta University Center – UNISUAM, Rio de Janeiro, Brazil, ^4^Postgraduate Program in Physical Therapy, Federal University of São Carlos, São Paulo, Brazil.

**Introduction**: Cognitive Functional Therapy (CFT) is a physiotherapy-led intervention that has evolved from an integration of foundational behavioral psychology and neuroscience within the physiotherapist practice directed at the multidimensional nature of chronic low back pain (CLBP). This study investigated the efficacy of CFT versus a sham procedure for disability and pain intensity for patients with nonspecific CLBP.

**Methods**: Randomized sham-controlled trial conducted in a primary care public health service in Brazil. A total of 152 participants were randomly assigned to CFT group (n = 76) and to a sham group (n = 76). CFT group received 6 one-hour individualized sessions; sham procedure group received 6 individual sessions of neutral talking + detuned photobiomodulation. Both groups received an education booklet with information on strategies of CLBP self-management. Primary outcomes were pain intensity (numeric pain rating scale, 0–10) and disability (Oswestry Disability Index, 0–100) at 6 weeks. Participants were assessed pre-intervention, postintervention (at 6 weeks), and 3 and 6 months after randomization.

**Results**: We obtained primary outcome data from 97.37% (n = 74) of participants in the CFT group and 98.68% (n = 75) from the sham group. CFT group showed larger effect sizes in pain intensity (mean difference [MD] = −1.72; 95% CI −2.38 to −1.07; effect size = 0.80) and disability (MD = −9.88; 95% CI −13.08 to −6.68; effect size = 0.95) postintervention compared to sham. The effect remained at the 3-month and 6-month follow-ups.

**Discussion/Conclusions**: CFT showed efficacy when compared with a sham procedure for pain intensity and disability for CLBP, sustained post-treatment, with a meaningful clinical and large effect size.

## Living with Chronic Pain and Excessive Polypharmacy: Insights into the Experience of Taking Multiple Medications

Joséanne Desrosiers^1, 2^, Gabriel Gingras-Lacroix^1^, Andréanne Bernier^1, 2^, Hermine Lore Nguena Nguefack^1^, Gwenaelle De Clifford-Faugère^1^, Nancy Ménard^1^, Sylvie Beaudoin^1^, M. Gabrielle Pagé^3^, Line Guénette^4^, Catherine Hudon^5^, Oumar Mallé Samb^2^, Anaïs Lacasse^1, 2^

^1^Chaire de recherche institutionnelle en épidémiologie de la douleur chronique, Université du Québec en Abitibi-Témiscamingue, Rouyn-Noranda, ^2^Département des sciences de la santé, Université du Québec en Abitibi-Témiscamingue (UQAT), Rouyn- Noranda, ^3^Centre de recherche, Center hospitalier de l’Université de Montréal, Montréal; Département d’anesthésiologie et de médecine de la douleur, Faculté de médecine, Université de Montréal, Montréal, ^4^Axe Santé des populations et pratiques optimales en santé, Center de recherche du CHU de Québec – Université Laval, Québec; Faculté de pharmacie, Université Laval, Québec, ^5^Département de médecine de famille et médecine d’urgence, Faculté de médecine et des sciences de la santé, Université de Sherbrooke, Sherbrooke

**Introduction**: Chronic pain (CP) affects 27% of the population and represents a significant burden on physical and emotional well-being. Excessive polypharmacy is defined as the use of ≥10 medications concurrently. About 26% of people living with CP are in an excessive polypharmacy situation. Despite its frequency, little is known about excessive polypharmacy in the context of CP. This project explored the experience of excessive polypharmacy among people living with CP.

**Methods**: Using a descriptive-interpretive qualitative approach, we explored perceived advantages and disadvantages of taking multiple medications, as well as concerns and needs. We conducted 11 individual semistructured interviews with adults living with CP, who spoke French, and were currently using or had used ≥10 medications concurrently within the past year. Discussions were video-audio-recorded using a virtual platform, and verbatim transcriptions were analyzed using a thematic analysis.

**Results**: Participants shared that taking multiple medications helps them regain control over their lives, enabling participation in family, work, and daily activities. However, they noted disadvantages such as financial costs, side effects, and complexities of pills management. Concerns included, among others, long-term effects, pill availability, and dependence. Despite everything, participants reported finding a balance between the advantages and disadvantages of polypharmacy.

**Discussion/Conclusions**: Our findings provide insight into the experience of excessive polypharmacy among people living with CP. Before considering medication reduction, prescribers should engage in discussions with their patients, viewing them and their experiences as a whole and involving them as partners in decisions.

## Does Opioid Tapering Reduce Long-Term Opioid-Related Harms: A Systematic Review and Meta-Analysis of Observational Studies

Dalraj Dhillon^1^, David Gou^2^, Alex Sy^3^, Jaskarn Ghotra^2^, Matthew Song^2^, Lucas Ilic^2^, Rachel Couban^4^, Jason Busse^5^, Li Wang^5^

^1^Michael G. DeGroote School of Medicine, McMaster Univeristy, ^2^McMaster University, ^3^Department of Medicine, University of Ottawa, ^4^Michael G. DeGroote National Pain Center, McMaster University, ^5^Michael G. DeGroote National Pain Center; Department of Anesthesia, McMaster University

**Introduction**: Opioid guidelines recommend tapering high-dose chronic opioid therapy despite long-term outcomes being unclear. Our systematic review aims to assess the long-term benefits and harms associated with opioid tapering.

**Methods**: We searched MEDLINE, EMBASE, AMED, PsycInfo, CENTRAL, CINAHL, PubMed, Web of Science to November 2023 for observational studies comparing opioid tapering vs stable/escalating dose among chronic opioid users. Reviewers screened literature, assessed risk of bias, and extracted study data in duplicate. We conducted random-effects meta-analysis to pool measures of association.

**Results**: Fifteen studies with 826,726 participants proved eligible, including 14 studies from USA and one from Canada. Most studies (14/15) used administrative data. Nine studies had a representative population; all 15 used valid measures for tapering and outcomes; 10 appropriately adjusted regression models; and 13 studies with loss to follow-up <20%. Meta-analyses showed opioid tapering may increase the risk of suicide (odds ratio [OR] 1.78, 95%CI 1.15 to 2.73), mental health crisis (OR 1.58, 95%CI 1.29 to

1.95), emergency department (ED) visits (OR 1.33, 95%CI 1.05 to 1.70), and hospitalization (OR 1.25, 95%CI 1.05 to 1.50); but not for opioid overdose (OR 1.09, 95%CI 0.79 to 1.50), opioid use disorder (OR 1.90, 95%CI 0.64 to 5.60), or accidents/injuries (OR 0.79, 95%CI 0.48 to 1.29).

**Discussion/Conclusions**: Limited low-quality evidence showed that opioid tapering may be associated with increased risk of suicide, mental health crisis, ED visits, and hospitalization. Future well-designed studies are needed to explore both short- and long- term benefits and harms of opioid tapering and to optimize opioid tapering strategies.

## Advancing Accessibility in Chronic Pain Care: Outcomes of a Virtual Tertiary-Care Rehabilitation Program in Newfoundland and Labrador, Canada

Julie Dwyer^1^, Heather Foley^2^, Josh Rash^1^, Patricia Poulin^3^

^1^Memorial University, ^2^NL Health Services, ^3^Ottawa Hospital

**Introduction**: As healthcare systems evolve, innovative solutions are needed to overcome barriers to equitable care. This study assessed the impact of virtual delivery of the Center for Pain and Disability Management (CPDM), an intensive chronic pain rehabilitation program in Newfoundland and Labrador, Canada.

**Methods**: A retrospective cohort design analyzed data from 229 adults who completed the 5-week CPDM program (Mar 2018-Aug 2022) in virtual (n = 112) or in-person (n = 117) formats. Standardized questionnaires evaluated changes in pain, depression, disability, self-efficacy and acceptance.

**Results**: Mixed-model ANOVAs were used to evaluate equivalence between groups. Results revealed that virtual and in-person formats achieved equivalent clinical improvements across pain, depression, disability, and self-efficacy (all p’s > .43), demonstrating that virtual care does not compromise quality. A significant main effect was found for acceptance, indicating that in-person participants reported slightly higher acceptance scores overall compared to virtual participants (p = .03). Notably, virtual delivery reduced wait times from 20–26 months to 12 months, improving accessibility, particularly for those in rural areas.

**Discussion/Conclusions**: This study highlights the potential of virtual models to transform chronic pain care by improving equity, reducing barriers, and maintaining effectiveness. Future investigations should examine the sustainability of hybrid care models and their long-term impact on health outcomes.

## Unlocking Relief: Stellate Ganglion Block as a Breakthrough PTSD Treatment for Frontline Workers

Dana El-Mughayyar^1^, Christina Saunders^1^, Julia Bennett^1^, Sarah Messer^1^, Dr. Todd Chedore^1^, Dr. Adesanya Tolulope Alugo^1^

^1^Horizon Health Network

**Introduction**: Post-traumatic stress disorder (PTSD) is a concern for frontline workers, triggered by extreme stressors. Stellate Ganglion Block (SGB) has emerged as a potential intervention by modulating the sympathetic nervous system through the stellate ganglion near the C7 vertebra. In 80% of individuals, the stellate ganglion is a fusion of the inferior cervical and first thoracic ganglia, while in 20%, they remain separate. SGB helps reduce sympathetic overactivity, associated with elevated nerve growth factor and norepinephrine levels. This study examines the efficacy of ultrasound-guided SGB in alleviating PTSD symptoms in frontline workers.

**Methods**: The study was an experimental, single-center investigation focusing on frontline workers experiencing PTSD symptoms. The effectiveness of SGB was assessed in managing key PTSD symptoms, including intrusive reexperiencing of traumatic events, avoidance of trauma-related stimuli, negative mood and cognitive changes, and persistent physiological hyperarousal. Six participants received SGB injections following institutional ethics approval. The first injection was administered on the right side, followed by the left side 2 weeks later. Repeat procedures were performed every 3 to 6 months, or as needed. Epinephrine-free 0.25% bupivacaine was used as the local anesthetic. *Outcome Measures*: A 10-question questionnaire was used to evaluate PTSD symptoms, focusing on hypervigilance, nightmares, intrusive thoughts, and the impact on daily functioning. Symptoms were assessed before treatment and again two weeks after the injection to measure the effectiveness of SGB.

**Results**: Findings indicate that SGB significantly reduces PTSD symptom severity in frontline workers. Marked improvements were observed in hypervigilance and intrusive thoughts.

**Discussion/Conclusions**: SGB shows promise as an adjunctive treatment for PTSD, offering symptom relief and improving overall well-being in frontline workers. However, further large-scale studies are necessary to validate these findings.

## Can Tele-Physiotherapy Match Traditional Care? A Study on Shoulder Pain Recovery

Fatemeh Ehteshami^1^, Khadije Otadi^1^, Nastaran Ghotbi^1^

^1^Tehran University of Medical Sciences

**Introduction**: Subacromial Pain Syndrome (SAPS) accounts for many shoulder-related injuries, with muscle weakness playing a key role in its development. Tele-rehabilitation (TR), which incorporates online exercise therapy and myofascial release techniques, has emerged as a potential alternative to traditional clinic-based physiotherapy. This study aimed to compare the effectiveness of tele-rehabilitation with in-clinic therapy, focusing on pain reduction, range of motion (ROM), and functional improvement in SAPS patients.

**Methods**: Forty-five individuals diagnosed with SAPS were randomly assigned to three groups: 1 – videoconferencing group, 2 – prerecorded video-based treatment, and 3 – conventional clinic-based therapy. All participants received structured interventions incorporating exercise therapy and self-release techniques. Assessments were conducted at baseline, mid-treatment, and three weeks post-treatment to evaluate pain, ROM, and functional outcomes.

**Results**: All three groups showed progressive pain reductions and improved shoulder mobility and function over time. By the end of the intervention, most patients experienced a meaningful reduction in pain. Flexion and external rotation improved noticeably, and shoulder function, measured by SPADI, showed significant improvement. Despite slight variations among the groups, the overall differences in treatment outcomes were not statistically significant. The results showed a strong treatment effect for all methods.

**Discussion/Conclusions**: A 10-session tele-rehabilitation program, whether delivered through live videoconferencing or prerecorded video guidance, is as effective as clinic- based therapy in improving pain, mobility, and function in SAPS patients. Self-release techniques and progressive exercises were key contributors to these improvements. Given the comparable benefits, tele-rehabilitation may serve as a feasible alternative to traditional in-person physiotherapy.

## Investigating Chemotherapy-Induced Peripheral Neuropathy-Related Treatment Changes in People with Breast Cancer: A Retrospective Chart Review

Antoine Frasie^1, 2, 3^, Maud Bouffard^1, 2^, Noémie Lavoie^1, 2^, Sarah Béland^1, 2^, Robert H. Dworkin^4, 5^, Jennifer Gewandter^6^, Julie Lemieux^1, 7^, Josée Savard^1, 2, 8^, Philip L. Jackson^8, 9,10^, Sophie Lauzier^11, 12^, Bruno Gagnon^1, 2, 3^, Anne Dionne^1, 7, 11^, Pierre Gagnon^1, 2, 13^, Lucia Gagliese^14, 15^, Lynn R. Gauthier^1, 2, 3^

^1^CHU de Québec-Université Laval Research Center, Oncology Division, Quebec, Canada., ^2^Université Laval Cancer Research Center, Quebec, Canada., ^3^Department of Family and Emergency Medicine, Faculty of Medicine, Université Laval, Quebec, Canada., ^4^Department of Neurology, University of Rochester Medical Center School of Medicine and Dentistry, Rochester, United States of America, ^5^Department of Anesthesiology and Perioperative Medicine, University of Rochester Medical Center School of Medicine and Dentistry, Rochester, United States of America, ^6^Department of Anesthesiology and Perioperative Medicine, ^7^Centre des maladies du sein Deschênes-Fabia, CHU de Québec, Quebec, Canada, ^8^School of Psychology, Faculty of Social Sciences, Université Laval, Quebec, Canada, ^9^Centre Interdisciplinaire de Recherche en Réadaptation et Intégration Sociale, Quebec, Canada, ^10^CERVO Research Center, Canada, ^11^Faculty of Pharmacy, Université Laval, Quebec, Canada, ^12^CHU de Québec-Université Laval Research Center, Population Health and Optimal Health Practices Unit, Quebec, Canada, ^13^Department of Psychiatry and Neurosciences, Université Laval, Quebec, Canada, ^14^School of Kinesiology and Health Science, York University, Toronto, Canada, ^15^Departments of Anesthesia and Psychiatry, University of Toronto, Toronto, Canada

**Introduction**: Chemotherapy-induced peripheral neuropathy (CIPN) impairs quality of life with limited palliative options. The American Society of Clinical Oncology recommends chemotherapy treatment changes (TC) like dose reduction (DR) or premature discontinuation (PD) “in the case of intolerable neuropathy and/or functional nerve impairment” but their impact on survival is unclear and decision-making guidelines are ambiguous. This study examines CIPN-related TC decisions in taxane-based breast cancer chemotherapy.

**Methods**: We conducted a retrospective chart review of 46 patients with CIPN-related DR/PD. Data from a routinely administered non-validated patient self-assessment checklist (e.g., numbness, tingling, weakness, lowered or loss of sensation, pain) and clinicians’ notes were analyzed using content analysis.

**Results**: CIPN description included numbness (71.7%) and tingling (34.8%) from checklists, and paresthesia (76.1%) and neuropathy (39.1%) from clinicians’ notes. Additional features across sources were location (feet:54.3%; hands:56.5%), frequency (54.3%), functional impact (41.3%), and toxicity grade (37.0%). Twenty-four patients (52.2%) had a single TC (DR:83.3%, PD:16.7%). Twenty-two,(47.8%) had sequential TCs (DR(s)+delay:50%; DRs:22.7%; DR(s)+PD:13.6%; DR+delay+PD:4.6%; delay+PD:4.6%; DR+increased dose:4.6%). The only/first TC was DR (69.6%), delay (21.7%), or PD (8.7%). DR notes key elements included one or more of increase/constant numbness/paresthesia, neuropathy grade 1–2, no functional impact. Delay notes included increase/constant paresthesia ± pain, numbness onset, toxicity, walking difficulty. PD notes included increase/constant numbness/paresthesia, sensitivity loss, neuropathy grade 2. Notations about patient discussion were unique to PD. Sequential TCs reflected symptom progression and emergence of functional impact notes.

**Discussion/Conclusions**: Data suggest assessment and decision-making inconsistencies. Additional real-world context research is needed to improve decision-making guidance.

## Is There Any Relationship Between Diaphragm Muscle Release and Shoulder Pain?

Khadijeh Otadi^1^, Fateme Ghaderi Varkani^1^, Siamak Bashardoust Tajali^1^, Kazem Malmir^1^, Fatemeh Ehteshami^2^

^1^Tehran University of Medical Sciences, ^2^University of Western Ontario

**Introduction**: Shoulder pain is the third most common musculoskeletal condition causing pain and disability in the shoulder area and other parts of the body. Release of the diaphragm may affect on pain and function due to physiological and neurological issues in these patients based on the interdependent model.

**Methods**: Twenty-four patients with shoulder pain were divided into intervention and control groups. Both groups received six routine sessions of low-frequency TENS, hot packs, and exercise therapy every other day during two weeks. in addition to traditions, the intervention group also received diaphragm myofascial release. Pain by VAS, disability based on Shoulder Pain and Disability Index (SPADI), and quality of life based on SF-12 was assessed.

**Results**: The average pain reduction during rest and daily activities was 2.62 and 1 mm in the intervention and control groups, respectively. The effect size based on Cohen d was 2.03 for shoulder disability according to SPADI. The quality of life increased in the intervention group in the mental dimension (MCS) and the control group in the physical dimension (PCS) without significance.

**Discussion/Conclusions**: According to the present study results, it seems that the release of diaphragm muscle in addition to the traditional treatment method can lead to a reduction of pain and disability and an increase in the quality of life of patients with chronic shoulder pain.

## Participants’ and Physiotherapists’ Views on the Delivery of a Randomized Controlled Feasibility Trial Investigating a Bespoke Dynamic Elastomeric Fabric Orthosis for Women with Chronic Pelvic Girdle Pain: A Qualitative Exploration

Bradley Halliday^1^, Jennifer Freeman^1^, Sarah Chatfield^1^, Lee Cameron^2^, Kirsty Carter^3^, Joanne Hosking^1, 4^, Jill Shawe^1, 5^

^1^Faculty of Health, University of Plymouth, England, ^2^Aneurin Bevan University Health Board, Wales, ^3^Cornwall Partnership NHS Foundation Trust, ^4^Peninsula Clinical Trials Unit, Plymouth, ^5^Royal Cornwall Hospital NHS Trust

**Introduction**: Chronic pregnancy-related pelvic girdle pain (PPGP) affects approximately 10–30% of postpartum women. PPGP is typically recalcitrant to standard care. A dynamic elastomeric fabric orthosis (DEFO) is one option to manage PPGP. A feasibility RCT (fRCT) was undertaken with participants randomized to control (advice and exercise (standard care)) or intervention (standard care and DEFO). Interventions were delivered remotely with data collection via a web-based app.

**Methods**: An embedded qualitative substudy involved online, semistructured interviews with seven participants and five clinicians to explore the experience of participating in the fRCT. Data were recorded, transcribed verbatim, and analyzed using thematic analysis.

**Results**: Four main themes were identified: “acceptability of trial methods,” “intervention acceptability,” “impact of intervention” and “adherence to exercise.” Data collection methods and remote delivery of the trial were acceptable; however, women and physiotherapists would have preferred at least one face-to-face intervention session. The DEFO was acceptable to women, providing them with a sense of support “holding them together.” They felt it increased awareness of their ability to move, enabling them to be more physically active. Physiotherapists felt the range of available exercises was restrictive. Physiotherapists and participants found the advice acceptable but with room for development. Participants struggled to maintain adherence to the prescribed exercise program over the 24-week period.

**Discussion/Conclusions**: The remote trial procedures and interventions were generally acceptable to both participants and physiotherapists, supporting the overall trial design and implementation. The participant’s experiences and suggestions will be considered in the design and delivery of a future definitive trial.

## Peer Support for Chronic Pain: Unique Needs, Preferences, and Links with Well-being

Susan Holtzman^1^, Sage Wiebe^1^, Carolyn Crawford^1^, Francois Louw^1, 2^, Paul Etheridge^1, 2^

^1^The University of British Columbia, ^2^Bill Nelems Pain and Research Center

**Introduction**: People living with pain often experience loneliness, invalidation, and social isolation, and this can adversely impact mental and physical health. A recent meta-analysis found evidence for a positive impact of peer support interventions for chronic pain. However, a broader understanding of peer support satisfaction, preferences, and engagement among people living with pain remains limited. In a large community-based sample, this study aimed to: (1) assess perceptions of loneliness and peer support, (2) examine associations between peer support and well-being, and (3) characterize preferences for, and barriers to, peer support.

**Methods**: Participants were recruited from an outpatient pain clinic in Western Canada. A total of 993 participants completed a survey that included detailed questions regarding peer support, as well as psychosocial and physical functioning.

**Results**: Almost half (48.6%) of participants reported significant loneliness and less than one-third (30.4%) reported satisfaction with peer support. Satisfaction with peer support was associated with significantly lower depression and anxiety and greater life satisfaction, even after controlling for general feelings of loneliness. Time and logistics were named as the biggest barriers to accessing peer support. Almost half of participants expressed the strongest preference for in-person and one-on-one support from peers, and a high variability in the preferred timing and frequency was noted.

**Discussion/Conclusions**: Our work identifies a significant need for enhancing peer support for people living with chronic pain. A one-size-fits all approach will be insufficient to address peer support needs in the community.

## T-Wo in One: A Novel T-Adapter for Simultaneous Ablation and Saline Delivery in Radiofrequency Ablation for Chronic Pain Management

Yasameen Ihsan^1^, Aamna Naveed^1^, Jialiang (Kevin) Hu^1^, Mohammed Hasan^1^, David Diao^1^, Eugene Maida^1^, Ahilraj Siva^1^

^1^McMaster University

**Introduction**: In Radiofrequency Ablation (RFA), the size of the lesion depends on factors such as tissue conductivity and the surrounding environment. Hypotonic saline infusion has been shown to enhance lesion size by increasing tissue conductivity through ion presence and slowing eschar formation, which acts as an insulator and completely limits lesion growth. Given this, our team of fourth-year undergraduate biomedical engineering students developed a 3D-printed T-adaptor prototype designed to attach to the cannula and provide a secondary port for saline infusion directly near the probe while it remains inserted.

**Methods**: We will assess lesion size in response to variations in the following parameters: timing of injections, temperature, impedance, volume of injection, and content of injection for monopolar RFA. These tests will be conducted using chicken breast tissue, with results compared to lesion size achieved in the current standard of RFA, where injections are absent during ablation. If significant results are observed, the protocol will justify future formal assessments using cadaver models.

**Results**: By providing a secondary port for delivering saline directly at the probe, we can potentially increase the size of lesions compared to standard RFA procedures without secondary access to the lesion site to inject saline. We hypothesize that the T-adapter technique will significantly increase lesion size compared to the current standard of care by enhancing tissue conductivity and minimizing tissue charring.

**Discussion/Conclusion**: The proposed T-adapter presents a feasible and cost-effective enhancement to standard RFA procedures, potentially improving clinical outcomes by increasing lesion size and ablation efficiency.

## Fostering Connections: Desired Changes to a Proposed Group Based Peer Support Program for Adolescents with Chronic Pain

Carter Janssen^1^, Delane Linkiewich^1^, Raissa Amany^2, 3^, Paula Forgeron^4^, Bruce Dick^5, 6^, Sara Ahola Kohut^7, 8^, C. Meghan McMurtry^9, 10^

^1^University of Guelph, ^2^Young Canadians Roundtable on Health, The Sandbox Project, ^3^Interdisciplinary School of Health Sciences, University of Ottawa, ^4^School of Nursing, University of Ottawa, ^5^Department of Anesthesiology and Pain Medicine, Psychiatry & Pediatrics, University of Alberta, ^6^Stollery Children’s Hospital, ^7^Department of Psychology, The Hospital for Sick Children, ^8^Department of Psychiatry, University of Toronto, ^9^Department of Anesthesia, McMaster Children’s Hospital, McMaster University, ^10^Department of Psychology, University of Guelph

**Introduction**: Chronic pain affects 21% of youth, leading to impairments in many domains of quality of life, including social functioning. Group peer support (GPS) is a promising approach to address the needs of adolescents with chronic pain (ACP) who have voiced a desire for meeting others. This study sought to investigate what changes ACP, caregivers of ACP, and healthcare professionals (HCP) recommend for two proposed formats of a GPS intervention (in-person and virtual).

**Methods**: Semistructured virtual interviews were conducted and analyzed using deductive and inductive qualitative content analysis. Demographic data were collected via the Qualtrics online survey platform.

**Results**: Results emphasized conversation facilitation and activities to help foster connections and build rapport (e.g., icebreakers). Age-specific groups (i.e., 12–14 and 15–17 years of age) were suggested to support developmental differences and friendship development. Further, making the atmosphere feel more comfortable, like creating a safety protocol or encouraging ACP to bring comfort items (e.g., blankets, heating pads). Lastly, choosing a youth-oriented name (i.e., GPS for Teens) were voiced as helpful to creating a casual and fun environment.

**Discussion/Conclusions**: This study provides insights into knowledge users’ recommendations for design and content revisions of a GPS intervention. Participants reported these changes may reduce ACPs’ feelings of awkwardness and enhance their comfort and thus engagement, attendance, and connection. Recommendations may facilitate relationship development which is critical as GPS seeks to provide ACP with a network of connections to provide social support aimed at improving quality of life.

## Cost-effectiveness of Cannabis for Medical Purposes Versus Opioids for Chronic Noncancer Pain

Haron Jeddi^1^, Jason Busse^1^, Behnam Sadeghirad^1^, Mitchell Levine^1^, Caroline MacCallum^2^, Li Wang^1^, Rachel Couban^1^, Jean-Eric Tarride^1^

^1^McMaster University, ^2^University of British Columbia

**Introduction**: Chronic noncancer pain affects 1 in 5 Canadians and is commonly managed with long-term opioid therapy. Concerns regarding rare but catastrophic harms associated with opioids, including overdose and death, have generated interest in alternatives including cannabis; however, the comparative cost-effectiveness of these management options is uncertain.

**Methods**: We used findings from a network meta-analysis of 90 randomized trials to develop a 1-year microsimulation model to compare costs and quality adjusted life years (QALY) between oral cannabis for medical purposes and opioids for chronic noncancer pain. We used a payer perspective for our analyses and obtained cost and utility data from publicly available sources. All costs are reported in 2023 Canadian dollars. All analyses were probabilistic, and we conducted sensitivity and scenario analyses to assess robustness

**Results**: Total mean annual cost per patient was $1,466 for oral cannabis for medical purposes and $1,851 for opioids, a difference of -$385 (95% confidence interval [CI] -$1,238 to -$36). QALYs were 0.586 for oral cannabis for medical purposes and 0.582 for opioids, a difference of 0.004 (95% CI: −0.002 to 0.019). Cost-effectiveness acceptability curves showed that oral cannabis was cost-effective in 93% of iterations at willingness- to-pay thresholds up to $50,000/QALY gained.

**Discussion/Conclusions**: Our findings suggest that oral cannabis for medical purposes is more cost-effective than opioids for the management of chronic pain.

## Equitable Care Practices in Pediatric and Adult Clinics Across the Ontario Chronic Pain Network

Abirami Kandasamy^1^, Ardith Baerveldt^2^, Etienne Bisson^3^, Alex Falcigno^4^, Susan Lutfallah^1^, Meaghan McKillip^1^, Michelle Nieuwesteeg^5^, Patricia Poulin^6^, Greg Tippin^7^

^1^Children’s Hospital, London Health Sciences Center, ^2^Holland Bloorview Kids Rehabilitation Hospital, ^3^Kingston Health Sciences Center, ^4^St. Joseph’s Care Group, ^5^Children’s Hospital of Eastern Ontario, ^6^The Ottawa Hospital, ^7^Hamilton Health Sciences

**Introduction**: The purpose of this study was to understand current equity, diversity, and inclusivity (EDI) practices among Ministry of Health funded chronic pain programs in Ontario.

**Methods**: Ontario Chronic Pain Network (OCPN) pediatric and adult pain clinics were surveyed on their current clinic practices related to EDI in relation to care access (referral, intake), implementation (assessment, treatment), and discharge/transition. The survey was developed by the OCPN Research & Evaluation Committee, an interprofessional group of clinicians representing pediatric and adult clinics across Ontario (e.g., Thunder Bay, London, Toronto, Hamilton, Ottawa, Kingston), as well as patient partners (adults and children with chronic pain). Results were analyzed using descriptive statistics.

**Results**: Results capture clinic referral processes, documentation of relevant demographic variables, examples of program modifications for accessibility (e.g., virtual programming, timeline of care), accommodations for rural communities, and clinic self-assessments on gaps in providing equitable care.

**Discussion/Conclusions**: Findings demonstrate the efforts of clinics across the province to incorporate EDI practices across the patient’s journey in the tertiary care setting, and opportunities for continued work and development to best serve patients with chronic pain in Canada.

## Pain Points: Bridging the Gaps in Women’s Chronic Pain Education

Huda Khayyat^1^, Kayla Saul^2^, Ginette Moorse^3, 4^, Rebecca Titman^3, 5^

^1^Division of Anesthesiology & Pain Medicine, Faculty of Medicine, University of Toronto, Toronto, ON, ^2^Temerty Faculty of Medicine, University of Toronto, Toronto, ON, ^3^Department of Medicine, Mount Sinai Hospital, Sinai Health, Toronto, ON, Canada, ^4^Division of Neurology, Temerty Faculty of Medicine, University of Toronto, Toronto ON, ^5^Division of Physical Medicine & Rehabilitation, Temerty Faculty of Medicine, University of Toronto, Toronto ON

**Introduction**: Certain chronic pain disorders, such as migraines, fibromyalgia, and endometriosis, disproportionately affect women. Recent studies reveal that residents feel unprepared to address women’s health, emphasizing the need for better training in residency programs. Our study aims to assess the current state of women’s health education within residency programs across Canada and identify barriers to integrating women’s health curricula.

**Methods**: A survey was sent to all PGME-accredited residency program directors (PDs) in Anesthesia, Pain Medicine, Neurology, and Physical Medicine and Rehabilitation (PM&R) across Canada. Contact information was obtained from publicly available data through the Canadian Resident Matching Service (CaRMS). The invitation was sent to a total of 43 programs.

**Results**: Fifteen PDs participated, with responses from Neurology (26.7%), PM&R (26.7%), and Pain Medicine and Anesthesia (46.7%). Only 40% of the programs offered didactic sessions on chronic pain in women. Despite 53.3% reporting that trainees were unprepared to manage chronic pain in pregnancy, upon graduation, 60% had no plans to expand education on this topic. Clinical exposure was absent or limited in at least 50% of programs. The most frequently cited barrier was lack of time, followed by insufficient expertise, and lack of educational resources.

**Discussion/Conclusions**: These findings suggest significant gaps in education and clinical exposure for trainees in managing chronic pain conditions in women and during pregnancy, highlighting key areas for improvement in residency training programs.

## Existential Therapy for Coping with Chronic Pain: A Scoping Review

Amara Kohlert^1, 2^, Kelsy Dabek^1^, Taylor Hill^3, 4^, Natasha Gallant^1^

^1^University of Regina, ^2^St. Francis Xavier University, ^3^Univeristy of Dundee, ^4^Dalhousie University

**Introduction**: Integrating an existential lens into chronic pain treatment enables a holistic and biopsychosocial approach to treating chronic pain. This scoping review aimed to identify the characteristics, themes, and results of existential therapy for treating chronic pain.

**Methods**: We searched seven databases for empirical papers that described an existentially based intervention for a chronic pain sample. Conducted in May 2023, the search terms were related to chronic pain and truncated versions of existential and humanistic therapies.

**Results**: Six articles met the criteria and were included. Extracted data included study characteristics and common intervention and outcome themes. All studies reported an easing of pain symptoms or severity. Physical functioning was generally not influenced by existential-spiritual therapy; however, it did improve with or in combination with cognitive-behavioral therapy. Meaningfulness was a common theme in all the interventions, and most articles reported increased meaningfulness in the outcomes. Spirituality seems to moderate the effectiveness of existential therapy.

**Discussion/Conclusions**: This study offers an overview of the current state of knowledge of existential therapies for chronic pain treatment and further provides suggestions for advancing the study of existential therapy for treating chronic pain by highlighting both commonalities and inconsistencies in intervention findings.

## Patient Risk Tolerances with Injectable Steroids for Chronic Myofascial Pain

Ajay Kumeta^1^, Rimsha Malik^1^, Jaskarn Bola^1^, Suneel Upadhye^1^

^1^McMaster University

**Introduction**: Interventional pain management of chronic myofascial pain includes use of injectable corticosteroids, with potential clinical adverse effects. This study’s goal was to ascertain patient risk tolerance balancing individual benefits of injectable steroids and their potential risks and subsequent management.

**Methods**: A survey instrument based on European League Against Rheumatism (EULAR) 2013 recommendations for steroid monitoring in clinical practice was created, pilot-tested and revised to optimize patient comprehension. Patients (n = 62) were surveyed to evaluate current benefits (pain relief, functional improvement), and the importance placed on future risks and management, and thresholds for steroid cessation.

**Results**: Average pain and functional improvement across all patients were 42.66% ± 1.83% and 42.50% ± 1.66%, respectively. Onset of coronary artery disease requiring surgical intervention was the strongest predictor of steroid cessation (Patient-Importance (PI): 70.17% ± 2.83%, Cessation (CS): 44.00% ± 6.00%). Steroid sequelae requiring surgery (bariatric, peptic ulcers, glaucoma, vertebral fractures) or insulin injections for new-onset diabetes were associated also with steroid cessation (PI: 48.83% ± 1.34%, CS: 36.00% ± 2.00%). Sequelae requiring new or revised medical therapies (antihypertensives, antacids, oral hypoglycemic agents) were less likely to result in injection steroid cessation (PI: 29.33% ± 1.00 %, CS: 16.00% ± 2.00%). Sequelae requiring lifestyle modifications (diet, exercise, glucose monitoring) were least likely to influence steroid preferences (PI: 26.17% ± 2.83%, CS: 11.00% ± 1.00%).

**Discussion/Conclusions**: Adverse effects requiring surgery or invasive interventions were the primary drivers of steroid cessation in chronic pain patients, while medication changes were moderately influential, and lifestyle management adjustments were least influential on such decisions. Pain physicians should consider individual patient risk tolerance for steroid injection adverse effects during informed consent discussions.

## Cannabis as an Opioid-Sparing Strategy: A Prospective Cohort Study in Chronic Pain (CP) Management

Shehnaz Fatima Lakha^1, 2^, Hadi Shojaei^3, 4^, Jennifer Lake^3^, Claudia Lai^5^, Peter Pennefather^1, 6^

^1^University of Toronto, ^2^Pain and Wellness Center, ^3^St. Joseph’s Care Group, ^4^Thunder Bay Regional Health Sciences Hospital, ^5^University of Victoria, ^6^gDial Inc.

**Introduction**: Prescription opioids significantly contribute to the global overdose crisis. Therapeutic cannabis, with a lower addiction risk, is increasingly used in CP management and may improve pain outcomes and reduce opioid dependence. This study explores the associations between cannabis use, pain management, and opioid tapering within standard care practices.

**Method**: This prospective, longitudinal study enrolled 19 new CP patients referred to the TBRHSC Ambulatory Care Unit between 2022–2023. The study lasted 12 months with follow-ups at baseline, 3, 6, and 12 months. Data was collected through clinic notes, EMR, patient feedback, and validated questionnaires on pain, opioid, and CBD use. The primary outcomes were pain control, functional improvement, and the relationship between cannabis use, pain, and opioid use. Study approved by the TBRHSC Human Ethics Research Board (Protocol #2021516).

**Results**: Of 19 screened patients, 14 enrolled and completed the baseline questionnaire (female/male ratio 2.8:1, mean age 56). Mean pain score (NRS) was 7 ± 1, with pain duration of 17 ± 11 years. Over 50% reported LBP, and 86% had multisite pain. Most were on strong opioids (mean dose 74 ± 60 morphine equivalent). Thirty-six percent (n = 5) tapered opioids and began CBD, while 29% (n = 4) tapered without CBD. At 3 months, CBD use was 20 mg twice/day. At follow-up, 88% reported severe pain, and 60% discontinued CBD due to side effects, cost, or medical reasons.

**Discussion/Conclusions**: One-third of patients showed potential benefits from CBD, but two-thirds discontinued due to side effects and other factors. This highlights the need for a personalized, patient-centered approach to opioid tapering.

## Trajectories of Symptom Change During Group Psychotherapy for Chronic Pain

Elisabeth Lamoureux^1, 2^, Karen Ghoussoub^1, 2^, Yoram Shir^3, 4^, Marc O. Martel^3, 4, 5^, Zakhar Prylutskyy^5^, M. Gabrielle Pagé^1, 2, 6^

^1^University of Montreal’s affiliated Hospital Research Center (CRCHUM), ^2^Department of Psychology, University of Montreal, ^3^Alan Edward Pain management unit, Montreal General Hospital, ^4^Faculty of Dental medicine, McGill University, ^5^Department of Anesthesia, Faculty of Medicine, McGill University, ^6^Department of Anesthesiology, University of Montreal

**Introduction**: It is now well established that multidisciplinary approaches are the gold standard for treating chronic pain and its associated concerns. Therefore, collaboration between physical and mental health providers, including group-based psychotherapy, are frequently offered for treating pain. In this prospective longitudinal observational study, we aimed to identify the trajectories by which changes in pain severity and psychological flexibility occur within group psychotherapy for chronic pain, as well as the baseline characteristics and post-treatment clinical outcomes that are associated with each trajectory.

**Methods**: Participants (N = 71) were recruited from a tertiary care multidisciplinary pain treatment center to partake in an 8-week group psychotherapy program for chronic pain. Pain-related, psychological, and sleep measures were administered at pre- and post-treatment, before each therapy session, and at three months follow-up. Growth Mixture Modeling was used to identify trajectories of symptom change during the 8-week intervention, and their associated predictors.

**Results**: Models providing the best fit to the data were a three-trajectory model for pain severity, a four-trajectory model for psychological flexibility (avoidance), and a three-trajectory model for psychological flexibility (cognitive fusion). Baseline levels of depression and pain unpleasantness significantly predicted trajectory membership for pain severity; baseline levels of depression and sleep disturbances significantly predicted trajectory membership for psychological flexibility (avoidance); and baseline levels of pain catastrophizing and pain intensity significantly predicted trajectory membership for psychological flexibility (cognitive fusion) (p < .05).

**Discussion/Conclusions**: Findings highlight that symptom change varies throughout group psychotherapy chronic pain. Additional research is needed to clarify the mechanisms underlying this variability across patients.

## Peripheral Magnetic Stimulation for the Treatment of Fibromyalgia: A Systematic Review and Meta-analysis

Alvin Leenus^1, 2^, Rayaan Rahman^3^, Elad Dana^4, 5^, Cody Tran^6^, Duncan Westwood^7^, Evgeny E. Osokin^8^, Yasmine Hoydonckx^1, 9^, Massieh Moayedi^7, 8, 10^, Salman Hirani^11, 12^, James S. Khan ^1,13^

^1^Department of Anesthesiology and Pain Medicine, University of Toronto, Toronto, ON, Canada, ^2^Faculty of Medicine, McGill University, Montreal, QC, Canada, ^3^Georgia Institute of Technology, Atlanta, GA, USA, ^4^Department of Anesthesia, Intensive Care and Pain Medicine, Meir Medical Center, Kfar Saba, Israel, ^5^Sackler School of Medicine, Tel Aviv University, Tel Aviv, Israel, ^6^Department of Anesthesia, McMaster University, Hamilton, ON, Canada, ^7^University of Toronto Center for the Study of Pain, University of Toronto, Toronto, Ontario, Canada, ^8^Centre for Multimodal Sensorimotor and Pain Research, Faculty of Dentistry, University of Toronto, ON, Canada, ^9^Toronto Western Hospital, Toronto, Ontario, Canada, ^10^Division of Clinical & Computational Neuroscience, Krembil Brain Institute, University Health Network, ^11^Department of Anesthesiology and Perioperative Medicine, Division of Pain Medicine, ^12^Oregon Health & Science University, Portland, OR, USA, ^13^Mount Sinai Hospital, Toronto, ON, Canada

**Introduction**: Fibromyalgia (FM), a chronic condition causing widespread pain and emotional challenges, predominantly affects women (70.7%) and costs the US $12–14 billion annually. Current treatments provide modest relief. Peripheral magnetic stimulation (PMS), a type of pulsed electromagnetic therapy, offers a novel approach by targeting inflammatory and neurological processes. This systematic review evaluates PMS’s benefits and risks for FM management, hypothesizing moderate pain relief with minimal adverse effects.

**Methods**: This systematic review and meta-analysis followed PRISMA guidelines and Cochrane methodology (PROSPERO CRD42021235164). A search of six databases (July 2023) targeted studies involving adult FM patients treated with PMS, assessing pain and functional outcomes. Screening and data extraction were performed independently by reviewers using a standardized form. Risk of bias was evaluated using Cochrane criteria, and evidence quality was assessed with GRADE. Meta-analysis employed a random-effects model, with heterogeneity assessed using the I^2^ statistic.

**Results**: From 9,578 citations, six RCTs involving 279 patients were included. PMS device parameters varied (400 nT-400 μT, 3–84 days). Meta-analysis showed significant short-term pain relief within 1–3 months (mean difference −1.86, p = .0002), though benefits diminished beyond 3 months. Functional outcomes were inconsistent, and no major adverse events were reported.

**Discussion/Conclusions**: PMS demonstrated short-term analgesic effects with minimal adverse events but inconsistent results across trials. Heterogeneity in PMS parameters and small sample sizes limit generalizability. Although PMS shows promise as a noninvasive pain management option, higher-quality RCTs are needed to establish its long-term clinical value and optimize its role in FM treatment.

## Development of a Web-based Registry to Track Outcomes of Advanced Procedural Interventions for Treating Chronic Pain Syndromes

Johanna Marie Liu^1^, Anuj Bhatia^1^, Victoria Bains^1^, Emma Robertson^1^, Anna Kalleitner^1^, Neilesh Soneji^1^, Pranab Kumar^1^, Ehtesham Baig^1^, Yasmine Hoydonckx^1^, Abeer Alomari^1^, Danielle Alvares^1^

^1^Toronto Western Hospital

**Introduction**: In Canada, 20% of the population lives with chronic pain (CP) and Canadians report it adversely affecting daily activities. Advanced procedural interventions (API) such as spinal cord stimulation (SCS), and intravenous ketamine infusions (KI) are offered for patients with refractory pain. Longitudinal evaluation of these treatments for pain and related domains via validated instruments is essential to fill in gaps in building real-world evidence.

**Methods**: This study reports the development of a registry to track long-term outcomes of patients with CP who receive API. The Canadian minimum pain dataset^1^ is integrated into the registry along with validated instruments for assessing the impact of interventions on pain-related domains^2^ via web-based questionnaires. Datasets at three months post-ketamine infusions and at 12 months post-SCS implant were collected.

**Results**: Data was collected from 292 patients (171 SCS and 121 KI). Results indicate 51% of patients following SCS and 29% of patients following KI achieved the minimal clinically important difference (MCID) of 30% or greater reduction in pain intensity at 12 and 3 months, respectively, along with improvement in pain-related domains (Table). Additionally, 81% of patients following SCS and 90% of patients following KI achieved the MCID improvement of 0.03 or greater increase in EQ-5D index at 12 and 3 months, respectively.

**Discussion/Conclusions**: This registry has the potential to fill in knowledge gaps regarding impact of API for patients with CP. The findings will lead to a better understanding of the disease process and hence deliver the best treatment options for patients with CP.

**References**

1. Lacasse A, et al. The Canadian minimum dataset for chronic low back pain research: a cross-cultural adaptation of the National Institutes of Health Task Force Research Standards. CMAJ Open. 2017;5(1):E237-E248.

2. Dworkin R, et al. Core outcome measures for chronic pain clinical trials: IMMPACT recommendations. Pain 2005;113:9–19.

## Attendance Patterns in the Group-Based Pain Education and Self-Management Programming of the Alberta Virtual Pain Program

Elena Lopatina^1, 2^, Susan Sobey-Fawcett^2^, Tina Hoang^1, 2^, Magali Robert^1, 2, 3^

^1^University of Calgary, ^2^Primary Care Alberta, ^3^Alberta Health Services

**Introduction**: The Alberta Virtual Pain Program (AVPP) is Canada’s first publicly funded, province-wide chronic pain program. One of the AVPP’s offerings is a group-based pain education and self-management program. The program is delivered via Zoom and includes 6 weeks with 2 sessions per week – one led by clinician facilitators and the other by peer support workers (PSWs). PSW-led sessions continue for up to one year provide continuous support and achieve better outcomes long-term.

**Methods**: Attendance data for the first 6 weeks of the group-based program was retrieved from electronic medical records for the first 13 cohorts. The total number of participants was estimated, and mean attendance and attrition rates were analyzed separately for facilitator-led and PSW-led sessions. Attendance was expressed as a percentage of participants from the first session, while attrition was calculated as the percentage decrease in attendance between the first and final sessions.

**Results**: A total of 101 patients participated across 13 cohorts. Facilitator-led sessions maintained higher attendance, with an average of 72% by Week 6 and an attrition rate of 43%. PSW-led sessions showed greater variability, with an average attendance of 37% by Week 6 and a higher attrition rate of 68%. Yet, two cohorts stood out with increased attendance in PSW-led sessions over time, reflected in negative attrition rates of −12% and −32%.

**Discussion/Conclusion**: Variability between cohorts was evident, with factors such as cohort-specific dynamics and scheduling differences potentially influencing attrition. Further investigation is needed to ensure the program meets patients’ needs effectively.

## Oral Delivery of Delta-9-tetrahydrocannabinol Has Pain-modifying Effects in Mouse Models of Knee Osteoarthritis

Anca Maglaviceanu^1, 2, 3^, Jason Rockel^1, 2^, Helena Fetter Filippini^4, 5^, Ewa Wasilewski^2, 6^, Melissa Lewis-Bakker^2, 6^, Shabana Vohra^1, 2^, Katrina Hueniken^1, 2^, Osvaldo Espin-Garcia^1, 2^, Chiara Pastrello^1, 2^, Pratibha Potla^1, 2^, Yangqing Deng^1, 2^, Keemo Delos Santos^1, 2^, Starlee Lively^1, 2^, Nikita Looby^1, 2^, Johana Garcia^1, 2^, Evan Pollock Tahiri^1, 2^, Behdad Ravarian^1, 2^, Laura Bennett^4^, Jian Wang^2^, Michael Fehlings^2^, Rajiv Gandhi^1, 2, 6^, Nizar Mahomed^1, 2, 6^, Timothy Leroux^1, 2, 6^, Rachel Miller^7^, Igor Jurisica^1, 2^, Lakshmi Kotra^2, 4^, Robert Bonin^4^, Hance Clarke^2, 8^, Mohit Kapoor^1, 2^

^1^Division of Orthopedics, Osteoarthritis Research Program, Schroeder Arthritis Institute, University Health Network; Toronto, ^2^Krembil Research Institute, University Health Network; Toronto, ^3^Department of Laboratory Medicine and Pathobiology, University of Toronto; Toronto, ^4^Leslie Dan Faculty of Pharmacy, University of Toronto; Toronto, ^5^School of Dentistry, Virginia Commonwealth University; Richmond, VA, ^6^Toronto Western Hospital, University Health Network; Toronto, ^7^Division of Rheumatology, Department of Internal Medicine, Rush University Medical College; Chicago, IL., ^8^Department of Anesthesia and Pain Management, Toronto General Hospital, University Health Network; Toronto.

**Introduction**: Osteoarthritis (OA) involves pathological changes in the joint and dorsal root ganglia (DRG) that contribute to chronic pain. Some OA patients use cannabis for symptom relief. We found oral administration of delta-9-tetrahydrocannabinol (THC) had disease-attenuating effects in the destabilization of the medial meniscus (DMM) mouse model of knee (K)OA. Here, we investigated THC’s effects and signaling mechanisms in KOA pain.

**Methods**: DMM and monosodium iodoacetate (MIA) mice were administered THC orally.

Pain was assessed using Von Frey tests. Plasma from THC-treated DMM mice 10-weeks postsurgery was analyzed by targeted metabolomics. Ipsilateral L3-L5 DRG were collected 3-weeks post-MIA injection and single nucleus RNA sequencing (snRNAseq) was performed to determine THC-induced transcriptomic changes in distinct cell populations. Computational and bioinformatics analyses were used to identify enriched pathways and infer intercellular communications.

**Results**: THC reduced pain in DMM and MIA mice. Metabolomics analyses revealed that serotonin, carnosine, and 5-oxoproline were reduced upon DMM surgery and rescued with THC administration. snRNAseq analyses identified transient receptor potential melastatin 8 (Trpm8)-expressing neurons, peptidergic nociceptors (Pep), and neurofilament (NF)-expressing neurons as having the highest DEGs in response to THC of all cells in the DRG. DEGs of Trpm8 neurons were enriched in neuronal transmission pathways, immune system- and lipid-related pathways. Cell communication analyses determined a putative decrease in ligand-receptor signaling between Trpm8 neurons and Pep/NF neurons with THC.

**Discussion/Conclusions**: In mouse models of KOA, THC reduced pain, and induced systemic metabolic changes and gene expression/pathway/communication changes in DRG Trpm8 neurons, suggesting a role in KOA pain modulation.

## Day-to-day Opioid Intake Among Patients with Chronic Pain Using Short-Acting Opioids: Exploring the Role of Pain Intensity, Psychological States, and Physical Dependence

Marc Martel^1, 2, 3^, Alice Bruneau^4^, Amanda Sirois^1, 3^, Jiaqi Bi^5^, Gabriella Spiegler^6^, Juliet Ware^7^, Reem Alghamdi^3^, Nuzhat Nipa^3^, M. Gabrielle Pagé^8^, Mark Ware^3, 9^, Jordi Perez^2, 3^

^1^Faculty of Dental Medicine, McGill University, ^2^Department of Anesthesia, McGill University, ^3^Alan Edwards Pain Management Unit, McGill University Health Center, ^4^Division of Experimental Medicine, McGill University, ^5^Department of Epidemiology and Biostatistics, Schulich School of Medicine & Dentistry, Western University, ^6^Department of Epidemiology, Biostatistics, and Occupational Health, McGill University, ^7^Department of Psychology, University of British Columbia, ^8^Department of Anesthesiology and Pain Medicine, Université de Montréal, ^9^Department of Family Medicine, McGill University

**Introduction**: Many patients with chronic non-cancer pain (CNCP) use opioid medications. The amount of opioids used by patients can fluctuate on a day-to-day basis, especially among short-acting opioid users. Given recommendations to use the “lowest effective doses” of opioids in patients with CNCP, research is needed to examine why certain days are characterized by greater opioid intake. Exacerbations in pain are commonly invoked to explain opioid intake, but patients’ psychological states and physical dependence could also contribute to greater daily opioid intake. Objectives: To explore the contribution of pain intensity, psychological factors, and physical dependence symptoms to daily opioid intake in patients with CNCP.

**Methods**: In this ecological momentary assessment (EMA) study, patients (n = 78) with CNCP prescribed short-acting opioids completed diaries, in between opioid doses, for 10 consecutive days. Diaries assessed a host of pain, psychological, and opioid-related variables. Diaries also assessed total daily morphine equivalent doses (MED) used by patients.

**Results**: Multilevel analyses indicated that day-to-day elevations in pain intensity, catastrophic thinking, and opioid craving were associated with greater daily opioid intake (i.e., MED) (all p’s < .05). Physical dependence symptoms were not associated with daily MED but were associated with opioid craving (p < .05). In adjusted models, craving was the only factor significantly associated with daily intake (p < .05).

**Discussion/Conclusions**: Our findings provide new insights into the factors contributing to daily opioid intake among patients with CNCP. Interventions targeting these factors could potentially prevent opioid dose escalations among patients maintained on opioid therapy.

## Finding our NICHE: Lessons Learned from the Pediatric Pain Pilot in Northwestern Ontario

Virginia McEwen^1^, Jennifer Cano^2^, Tara Kydd^3^

^1^Northern Ontario School of Medicine University, ^2^St. Joseph’s Care Group, Thunder Bay, ^3^George Jeffrey Children’s Center

**Introduction**: Children in Northwestern Ontario living with chronic pain typically must seek care in southern Ontario, posing significant access challenges and long-term impacts on health, academics, and future aspirations. This quality improvement project, funded by a Northern Ontario Academic Medicine Association grant, piloted a multidisciplinary pediatric pain program from November 2023 to December 2024. The goal was to assess local needs, feasibility, and to guide potential future programming.

**Methods**: The program was developed with input from other existing pediatric pain programs. The locally assembled team included a pain physician, physiotherapist, occupational therapist, psychology associate, and psychotherapy-trained social worker. Referral packages were distributed to pediatricians and primary care providers across Northwestern Ontario. Evaluated by the physician, patients were offered relevant allied health services. A virtual facilitated group pain psychoeducation series, based on the WebMap app and insights from other pain programs, was developed and offered to eligible patients.

**Results**: Over one year, 42 referrals for various pain conditions were received from more than 20 physicians. The pilot effectively provided local, specialized care with most participants reporting improved symptoms, management strategies, function, and satisfaction.

**Discussion/Conclusions**: The high referral rate demonstrates local demand and interest. The pilot successfully provided pediatric specialty care locally with positive feedback from families. A key strength that emerged was the team members’ ability to support patients across the lifespan. A weakness identified was the limitations of space, preventing stronger interdisciplinary collaborative work. The pilot demonstrated feasibility, clear community support for this program, and patient and family satisfaction.

## Real World Effectiveness and Tolerability of Low-dose Naltrexone to Treat Chronic Pain: A Retrospective Cohort Study of One Pain Physician’s Practice

Virginia Anne McEwen^1^, Hannah Aalto^1^, Sara Paul^1^

^1^Northern Ontario School of Medicine University

**Introduction**: Low-dose naltrexone (LDN) as an off-label treatment for chronic pain has shown promising results with a minimal side effect profile previously in the literature. Published data has found an inverse relationship of the prescription of LDN and opioid utilization. This retrospective cohort study looks to add to the growing body of literature by examining LDN prescriptions in one pain physician’s practice for patient-reported outcomes of both efficacy in chronic pain and documented adverse effects.

**Methods**: Retrospective cohort study methods were used to evaluate efficacy of LDN on pain symptoms in a pain physician’s practice in Thunder Bay, Ontario over a 3-year period. Two reviewers independent of the prescribing physician audited charts to extract pain diagnoses, reported benefits, and adverse effects. Using descriptive analysis, patients were categorized by primary pain diagnosis and considered responders if they self-reported sufficient clinical benefit to continue with LDN long-term.

**Results**: A total of 128 patients prescribed LDN underwent chart review, and 32 were excluded. Of the 96 patients included in final review, 54% of patients reported clinically meaningful benefit for pain. Subanalysis of pain diagnoses reporting higher response rates than the overall group included patients with fibromyalgia, arthritis, hypermobility spectrum disorder, mast cell activation syndrome, and pelvic pain. Adverse effects were consistent with previously reported studies.

**Discussion/Conclusions**: This study reports benefits and an adverse effect profile of LDN for chronic pain consistent with other published studies. LDN is suggestive as a reasonable pharmacological tool to include in the management of multiple chronic pain conditions.

## Making Sense of Pain: Five Years Supporting People Living with Chronic Pain

Gregg Moor^1^, Majd Khodari^1^, Helena Daudt^1^

^1^PainBC

**Introduction**: People who experience marginalization are more likely to have chronic pain than the general population and are more likely to experience barriers to accessing care and services. Making Sense of Pain (MSOP) is a self-management program designed for people with pain who experience marginalization and face barriers to accessing care. It is low-barrier, adaptable, and free for participants.

**Methods**: MSOP is currently held over a span of nine to ten weeks and is led by a trained facilitator. Facilitators provide participants with opportunities to learn about pain and simple strategies that can help them better manage pain in everyday life. Since its inception in BC in 2019, MSOP has reached more than 800 people. It has been culturally tailored to six communities (Punjabi-speaking, Arabic-speaking, Cantonese-speaking, Mandarin-speaking, 2SLGBTQ+ adults, and gender-diverse youth), translated into French, and spread beyond BC.

**Results**: In 2023, we offered 25 cohorts of MSOP in BC, 5 in ON, 4 in AB, 2 in YT, and 1 in each of QB, NS, and NL. On average, seven people attended each session. Overall, the feedback has been consistently positive. The social aspect – feeling validated and not alone – has turned out to be an important benefit of the program.

**Discussion/Conclusions**: Most participants report increased knowledge and are practicing the skills they’ve learned outside of the sessions. Collecting feedback from participants, however, has been a challenge. We are currently revising our facilitator training to highlight the MSOP principles and emphasize the importance of evaluation.

## High-dose Ketamine Infusion under the Magnifying Glass: Does Pain Catastrophizing Predict Patient Outcome?

Abi Muere^1^, Kathryn Curtis^1^, Emma Robertson^1^, Danielle Alvares^1^, Victoria Bains^1^, Anna Kalleitner^1^, Anuj Bhatia^1^, Neilesh Soneji^1^

^1^Ketamine Infusion Pain Program, Comprehensive Interventional Pain Program – Interventional Pain Service, Toronto Western Hospital and University of Toronto, Toronto, Ontario

**Introduction**: The multidisciplinary Ketamine Infusion Pain Program (KIPP) provides high- dose Ketamine infusion (KI) to patients with refractory chronic pain. KIPP has presented data demonstrating baseline differences in pain catastrophizing between KI responders versus nonresponders. A logical next step and the aim of this study was to determine if psychological factors predict KI response for pain reduction.

**Methods**: The study received REB approval (#2021-0046-P). Participants were KIPP patients who consented to provide clinical data (N = 139; mean years of pain = 8.8 ± 7.7, female (64.0%), mean pain score (0–10) = 6.8 ± 1.6; common diagnoses: neuropathic pain). Pain, pain catastrophizing (PCS), depressive symptoms, and anxiety scores were collected at baseline and one-month post-KI. Responders were participants reporting ≥30% pain reduction one-month post-KI.

**Results**: In all, 81 participants received a single-day KI (SDKI) and 58 participants received a multiday KI (MDKI). Of participants, 32.4% were responders (21% SDKI, 48% MDKI). A multivariate logistic regression using the total sample was conducted to determine if pain catastrophizing, depressive symptoms, and anxiety predict response to KI, but this model had overall poor discrimination with area under the Receiver Operating Characteristic curve (AUC) = 0.67. Secondary analyses revealed that the predictor model had acceptable discrimination for the MDKI cohort, AUC = 0.73. For each unit increase on the PCS, the odds of responding to a MDKI decreased by 8% (p < .05).

**Discussion/Conclusions**: Pain catastrophizing was identified as a novel predictor of MDKI response. Participants with a greater tendency to catastrophize had lower odds of responding to MDKI. Future clinical directions include providing targeted psychological intervention to reduce pain catastrophizing.

## Leveraging Patient Data to Enhance Tailored Pain Care: Insights from TAPMI’s Orientation Program

Adriano Nella^1^, Rachael Bosma^1^, Brittany Rosenbloom^1^, Andrew Smith^2^, Anuj Bhatia^3^, David Sussman^4^, David Flamer^5^, Hance Clarke^3^, Yasmine Hoydonckx^3^, Harikrishnan Gopalakrishnan^1^, Karen Ng^1^, Cara Stanley^1^, Kimberly Coombs^1^, Christian Aquino^1^, Sylvia Gomes^1^, Tania Di Renna^1^

^1^Women’s College Hospital, ^2^Centre for Addiction and Mental Health, ^3^University Health Network, ^4^St Michael’s Hospital, ^5^Sinai Health

**Introduction**: TAPMI is a partnership of five specialized hospitals managing pain care in Toronto, processing approximately 6,000 new referrals annually through centralized triage at Women’s College Hospital. To enhance patient-tailored care, an orientation session was introduced in 2024, designed to support patients while they await care and generate a continuously updated database with patient characteristics. This data helps identify gaps in care and informs clinical programming to meet patient needs.

**Methods**: Data from TAPMI patients who attended the orientation session between January 1 and November 11, 2024, and consented for research use were included. Variables collected included demographics (age, ethnicity, gender), pain characteristics (location, intensity, disability), trauma history, and mental health.

**Results**: A total of 1,767 patients attended the orientation session, with 1,083 (61.3%) consenting to research use. The average age of patients was 50 years (SD = 15.67). The majority identified as women (68.3%), White (59.5%), and spoke English as a primary language (87.8%). Nearly half (49.0%) had lived with pain for over five years, and 56% reported experiencing emotional distress related to trauma. Symptoms of anxiety and depression were present in 49.2% and 46.6% of the population, respectively, with 26.9% of patients reporting severe psychological distress.

**Discussion/Conclusions**: These findings enable tailoring of personalized care plans, ensuring the right care is provided at the right time. It also informs program planning and resource allocation, guiding the development of interventions for trauma and pain, and supports the creation of equitable, patient-centered care models through strategic partnerships and advocacy.

## Knowledge Translation in Primary Care: Cognitive Behavioral Therapy for Insomnia in People Living with Chronic Pain

Samuel Neumark^1^, Janine Noorloos^1^, Fairuz Karim^1^, Lisa Caldana^1^, Cristina Bostan^1^

^1^Temerty Faculty of Medicine, University of Toronto

**Introduction**: There is a need to increase the use of Cognitive Behavioral Therapy for insomnia (CBT-i) as a first-line treatment option for patients living with chronic pain. Primary care clinicians report insufficient knowledge about CBT-i mechanism of action, efficacy, benefits, and referral pathways. This project aims to develop an information tool for primary care physicians to increase the use of CBT-i as a first-line treatment option for patients living with insomnia and chronic pain.

**Methods**: We recruited five physicians from academic and community-based primary care practices in the Greater Toronto Area and applied the Translational Thinking Framework to guide the project. We conducted semi-structured interviews to verify the problem, confirm the unmet need, and establish co-design partnerships. We then performed a thematic analysis of interviews to generate key themes for ideation workshops and create a prototype patient handout.

**Results**: Physicians highlighted unfamiliarity with strategies to promote non-pharmacological therapies that manage insomnia and mental health challenges associated with chronic pain. In collaboration with the physicians, we refined the design, appearance, accessibility, and scientific content of our prototype to develop a two-page digital handout for integration into primary care electronic health records. The main topics within the handout include an explanation of CBT-i, its effectiveness, a comparison to pharmacotherapy, and accessible local CBT-i services for chronic pain.

**Discussion/Conclusions**: Educating primary care physicians about CBT-i and involving users in the codesign of knowledge translation materials is important to enhance the quality of life for people living with insomnia and chronic pain.

## Comprendre l’interaction entre la douleur et le contrôle moteur: la manipulation de la kinésiophobie chez des volontaires sains

Adrien Nourry^1, 2^, Pierre Morel^3^, Hervé Devanne^3^, Said Ahmaidi^1^, Guillaume Léonard^2^, Thierry Lelard^1^

^1^Laboratoire APERE, Université de Picardie Jules Verne, Amiens, ^2^Laboratoire GRAND, Université de Sherbrooke, Sherbrooke, Canada., ^3^Laboratoire URePSSS, Université du Littoral Côte d’Opale, Université de Lille, Université d’Artois, Calais, France.

**Introduction**: Les personnes souffrant de douleurs chroniques développent souvent une peur irrationnelle du mouvement, appelée kinésiophobie. Cette kinésiophobie influence la marche, mais ces adaptations comportementales observées chez les douloureux ont pour facteurs confondants la douleur et l’appréhension de la douleur. Cette étude examine comment la manipulation de la kinésiophobie affecte la motricité, sans douleur induite ni conditionnée, lors d’un mouvement automatique et lors d’un mouvement volontaire.

**Méthodes**: Vingt-six participants sains (âge moyen: 34 ± 14 ans) ont réalisé l’étude, avec un score de kinésiophobie de 34.3 ± 6.9 mesuré à l’aide du TSK-17. Ils ont effectué une tâche de marche et une tâche de pointage dans une condition contrôle et une condition où la kinésiophobie était accentuée par la menace d’effets indésirables au niveau lombaire (K+). Les paramètres de marche mesurés incluaient la vitesse, la cadence, la longueur d’enjambée et le temps de mouvement pour atteindre les cibles lors du pointage.

**Résultats**: Les modèles linéaires mixtes ont révélé en condition K+ que la vitesse (p = 0.002), la cadence (p = 0.009) et la longueur de pas (p = 0.002) augmentaient, indépendamment du score TSK. Pour la tâche de pointage, une augmentation significative du temps de mouvement a été observée sous la condition K+ pour une cible (p = 0.042).

**Discussion/Conclusion**: Les résultats indiquent que la manipulation de la kinésiophobie influence de façon indépendante les tâches de marche vs de pointage. Tandis que l’accélération de la marche pourrait refléter une stratégie de fuite automatique pour échapper à la menace, le ralentissement du pointage pourrait refléter une conscientisation de la menace. Néanmoins, le score de kinésiophobie ne semble pas expliquer les variations observées pour ces 2 tâches. Ces résultats suggèrent qu’au-delà du score de kinésiophobie, les stratégies motrices dépendraient de la perception de la menace associée à la tâche et de son objectif spécifique.

## Migraine Prescribing Patterns in Nova Scotia Primary Care Clinics: A Retrospective Analysis of the Maritime Research Network for Family Practice Database

MS O’Brien, M Grandy, JA Dawe

Dalhousie University

**Introduction**: Migraine is a common neurological disorder affecting approximately 8% of the population. People living with migraine report significant disability and pharmacological management of migraine is often warranted. There is currently limited data describing prescribing patterns among clinicians caring for patients with migraine in Canada. The goal of this study was to examine the prescribing patterns for migraine in primary care clinics in Nova Scotia.

**Methods**: Deidentified electronic medical record data from 85,000 patients at 67 primary care clinics in the Maritime Research Network for Family Practice (MaRNet-FP) database were analyzed for migraine prescribing from January 2019 to December 2024. Using ICD-11 and Anatomical Therapeutic Chemical medication codes, patients with a diagnosis of migraine were identified and prescribing of migraine and pain medications were extracted.

**Results**: In total 12,079 patients with a diagnosis of migraine (migraine, episodic, or chronic) were seen by a primary care provider over the five-year extraction period. Over 80% of these patients were identified at women. 26.5% of migraine patients were prescribed an acute migraine medication, the most common of which were triptans (15.7%) and anti-inflammatory drugs (10.2%). 47.2% were prescribed preventative agents, most commonly an antidepressant medication (29.7%).

**Discussion/Conclusions**: Canadian guidelines for the management of migraine recommend acute and/or preventative medications depending on the severity and frequency of attacks. This study identified prescribing patterns for migraine in primary care clinics in Nova Scotia. These results will help inform future educational programs for the treatment of migraine.

## Patient perceptions toward reduction or avoidance of opioids after knee and hip arthroplasty: A cross-sectional survey

Mansi Patel^1^, Darren Shing^2^, Annie George^1^, Dhivya Bhaskaran^1^, Anthony Atalian^1^, Manpreet Walia^1^, Milin Patel^1^, Daniel Tushinski^1^, Anthony Adili^1^, Kamal Bali^1^, Vickas Khanna^1^, Sheila Sprague^1^, Kim Madden^1^, Jason Busse^1^

^1^McMaster University, ^2^University of Ottawa

**Introduction**: Opioid analgesics are routinely prescribed to manage pain after total joint arthroplasty but are associated with important harms. We explored the proportion of patients who would be open to receiving opioid-free or opioid-reduced postoperative care.

**Methods**: We administered a cross-sectional survey to patients scheduled to undergo, or who had undergone, total knee or hip arthroplasty. We constructed multivariable logistic regression models to explore features associated with patients’ receptivity to opioid reduction or avoidance.

**Results**: We approached 200 patients, and 190 provided a completed survey. A quarter of respondents believed that other analgesics were similarly effective or superior to opioids, and 68% perceived that opioids were associated with more side effects than alternatives. Fifty percent advised they would be receptive to reduced opioid use post- operatively. Endorsement was associated with not using opioids at the time of survey completion (OR 2.5, 95%CI 1.04 to 6.4), and the belief that opioids had more side effects than alternatives (OR 3.4, 95%CI 1.5 to 7.9). Forty percent of respondents advised they would be willing to avoid opioid use after surgery, and endorsement was associated with the belief that opioids cause more side effects than alternatives (OR 4.3, 95%CI 1.8 to 11.9) and that non-opioid analgesics are similarly or more effective (OR 3.4, 95%CI 1.4 to 8.3).

**Discussion/Conclusions**: Many participants were willing to reduce or avoid the use of postoperative opioids, and receptivity was strongly associated with beliefs regarding the comparative benefits and harms of alternatives.

## Generalized Negative Expectancies Associated with Pain Catastrophizing

Antonina D. S. Pavilanis^1^, Christiane Konstantopoulos^2^, Mathieu Roy^1^, Michael J. L. Sullivan^1^

^1^McGill University, ^2^Stanford University

**Introduction**: Pain catastrophizing, broadly conceptualized as an appraisal process where individuals expect the worst with respect to their pain experience, has emerged as one of the most robust psychological predictors of adverse pain outcomes. A number of studies have shown that individuals with high catastrophizing scores expect to experience more pain. It is not clear whether this is associated with pain-specific negative expectancies, or with a wider range of negative expectancies. The present study examined the association between pain catastrophizing and generalized negative expectancies.

**Methods**: Participants (N = 388) with whiplash-associated disorder were recruited from six multidisciplinary pain clinics in Greater Montreal (2012–2015). They underwent a seven-week rehabilitation program including medication management, education, exercise, and self-management training. Self-report measures, including the Pain Catastrophizing Scale (PCS) and Medical Expectancy Inventory (MEI), were completed at treatment admission. Pearson correlations examined associations between PCS scores and 15 MEI expectancy items.

**Results**: Higher pain catastrophizing was significantly associated with more negative health and recovery expectancies. After Bonferroni correction, eight MEI items remained significant. Greater catastrophizing correlated with lower expectations of pain reduction (r = −0.454, p < .001), physical recovery (e.g., 30-minute walk, r = −0.313, p < .001), and functional independence (e.g., household tasks, r = −0.390, p < .001). It also correlated with expectations of worsening pain (r = 0.250, p < .001) and health deterioration (r = 0.312, p < .001). Expectancies regarding lifting ability, return to full-time work, and complete recovery were nonsignificant after correction.

**Discussion/Conclusions**: Pain catastrophizing extends beyond pain-specific concerns to general recovery expectancies, leading to worse health, lower functional capacity, and continued medication reliance. These findings highlight expectancy-based interventions as potential targets for mitigating the impact of pain catastrophizing. Future research will investigate these associations while accounting for pain severity and depressive symptoms as potential confounding factors.

## Clinical Evidence with the Combination of Ibuprofen and Diphenhydramine for the Management of Pain and Sleeplessness

Richard Petruschke^1^, Peter Gao^1^, Connor Geddis^1^

^1^Haleon

**Introduction**: Pain can contribute to sleeplessness. Sleep helps your body to store energy and information and recover from activities and injuries. Inadequate sleep can have a negative impact on health. To manage pain and sleeplessness, there are a number of combination analgesic and sleep products, including ibuprofen/diphenhydramine (IBU/DIPH).

**Methods**: Four placebo (PBO)-controlled clinical studies were conducted with solubilized IBU/DIPH 400/50 mg per dose. Subjects were post-third molar extraction with moderate to severe pain experiencing sleeplessness. We evaluated common study endpoints including duration of sleep, onset of sleep, global rating of sleep, and use of rescue medicine.

**Results**: In four studies, the range of subjects with duration of sleep >6 hours with IBU/DIPH was 57%-70% versus PBO 3%-15%. Average minutes to sleep was 30.8–45 minutes with IBU/DIPH versus 63.8->180 minutes with PBO. Global assessment of sleep was rated as good, very good, or excellent for 51.6–79.3% with IBU/DIPH versus 2.5–14.6% with PBO. Average time to use of rescue medication was >12 hours with IBU/DIPH versus ≤2.1 hours with PBO, and rescue medication was 33.6%-45.8% with IBU/DIPH versus 81%-95% with PBO.

**Discussion/Conclusions**: Pain and sleeplessness are commonly experienced and can have long term impacts on health. Solubilized IBU/DIPH is a well-established treatment for pain with sleeplessness. In the studies evaluated, the product consistently provided longer duration of sleep, faster onset of sleep, a better global sleep experience, and less rescue medication use. The improved total sleep experience allows the user to optimize the benefits of sleep on overall health.

## Development of an Outpatient City-Wide Transitional Pain Service in Calgary

Magali Robert,^1^ Diane Colley-Urquhart,^1^ Arlene Cox,^1^ Angie Yang,^1^ Becky Job,^1^ Jeff Krahn,^1^ Tina Samuel^1^

^1^Calgary Chronic Pain Program

**Introduction**: Approximately 10% of surgical patients will develop chronic pain.^1^ Early intervention through development of a transitional pain service provides an opportunity to change the chronic pain trajectory, decrease opioid use and improve patient outcomes.

**Methods**: The development challenge was to incur no additional costs by utilizing existing infrastructure and resources and serve all four hospital sites and contracted surgical services within the Calgary zone which performed over 110,000 surgeries in 2023. The program was designed with engagement of all major stakeholders ranging from people with lived experiences to policymakers; following a thorough environmental scan of pre- existing programs.

The six-month program delivers an outpatient service, in the Chronic Pain Center, using a hybrid in-person and virtual group-based approach delivered by a core team (nurse coordinator, psychologist, occupational therapist and physician). Both preoperative and postoperative programing use pain science education principles, acceptance commitment and cognitive behavioral therapy approaches, medical and interventional management. To minimize overlap, the program leverages preexisting resources such as the Alberta Prehab Program, Alberta Virtual Pain Program, Alberta Virtual Rehab Line and Alberta Healthy Living Programs.

**Results**: Evidence-based inclusion criteria help streamline referrals (with wait-times under a month). Since full launch Sept 15, 2024, 40 people have been referred to the program. Most patients are discharged back to their medical home, some directly enter the Chronic Pain Center if required.

**Discussion/Conclusions**: Results indicate that similar programs can be implemented with the use of minimal infrastructure and financial support. The Calgary Transitional Pain Service offers a scalable model for other regions.

## Bridging The Gap: Impact of Prolonged Wait Times on Pain and General Health

Magali Robert,^1, 2, 3^ Andrew Walker,^2, 3^ Tina Samuel^3^

^1^Department of Obstetrics & Gynecology – University of Calgary, ^2^Department of Anesthesiology, Perioperative & Pain Medicine – University of Calgary, ^3^Department of Anesthesia (Pain Medicine) – Alberta Health Services

**Introduction**: Prolonged wait times for neuromusculoskeletal pain care pose a growing global challenge, with significant impacts on well-being, human suffering, and the economy.^1,2^

**Methods**: This cross-sectional study analyzed 90 survey respondents stratified by waitlist duration (< 3 months, 3–6 months, and > 6 months) for a multidisciplinary chronic pain program in Calgary, Alberta. Patient demographics, health confidence and pain-related characteristics were compared across groups using Chi-Square, One-way ANOVA, Kruskal-Wallis, and Fisher’s Exact tests. Significant findings (p < .050) prompted between-group comparisons with an adjusted p-value of <0.017 to account for multiple comparisons.

**Results**: No significant associations were found between wait times and patient demographics, health confidence and most pain-related characteristics. Patients waiting 3–6 months or > 6 months reported significantly worsened pain compared to those waiting < 3 months (< 3 months: 24.1%, 3–6 months: 62.1%, > 6 months: 58.6%, p = .003). Similarly, general health showed a significant worsening compared to < 3 months (< 3 months: 26.7%, 3–6 months: 69.0%, > 6 months: 62.1%; p = .005). No significant differences in pain status or general health were found between the 3–6 months and > 6 months groups.

**Discussion/Conclusions**: Longer wait times are linked to worsening pain and general health. This deterioration is seen after 3 months on a waitlist and then stabilizes. This highlights the need to see referred patients expeditiously or consider interim interventions to improve overall quality of life for people living with pain.

**References**: (1) Lynch ME, et al. Pain. 2008 May;136(1–2):97–116. doi: http://10.1016/j.pain.2007.06.018. Epub 2007 Aug 17. PMID: 17707589.

(2) Burke AL,et al. J Health Psychol. 2020 Aug;25(9):1198–1212. doi: http://10.1177/1359105317752828. Epub 2018 Jan 11. PMID: 29322830.

## Treating Nociplastic Pain is Possible: Initiating a Pain Reprocessing Therapy Program at an Interdisciplinary Tertiary Pain Clinic

Lori Selkirk^1^, Suzanne Deutsch^1^, Emily Woodburn^1^, Etienne J. Bisson^1, 2^

^1^Kingston Health Sciences Center, ^2^Queen’s University

**Introduction**: The aim of this study is to describe the development and preliminary results of a new Pain Reprocessing Therapy (PRT) program for adults with nociplastic pain receiving care at an interdisciplinary tertiary pain clinic.

**Methods**: The PRT program was developed at the Chronic Pain Clinic of Kingston Health Sciences Center (KHSC) by Allied Health providers with PRT training and certification. The program includes a 2-hr virtual session per week for 8 weeks covering topics of pain neuroscience, mind-body concept, pain-fear cycle, somatic tracking, overcoming setbacks, addressing other psychological factors, overcoming fear-inducing stimuli, and developing a toolkit. To determine program eligibility, patients undergo a chart review and a clinical interview that assess the presence of nociplastic pain, their pain-related beliefs, and readiness to engage in a non-tissue-based approach. Two groups were completed between January and November 2024. Patients were asked to complete pre- and post-outcome measures and had the opportunity to provide feedback.

**Results**: Of 19 adults with nociplastic pain referred to PRT, 17 completed the program (88.2% female, mean age = 52 ± 12 years). The majority of patients (85.7%) reported noticeable changes after completing the program, with improvements in all outcome measures. All patients were highly satisfied with the program and provided rich feedback for further program improvement.

**Discussion/Conclusions**: The preliminary results suggest that adults with nociplastic pain receiving care at the KHSC Chronic Pain Clinic benefited from the PRT program. The ongoing PRT program will continue to support adults with nociplastic pain and provide data to further evaluate effectiveness.

## Ultrasound-guided Supine Popliteal Sciatic Nerve Block: Crosswise Approach to Popliteal Sciatic (CAPS) versus Posterior Approach

Varun Singla^1^, Nidhi Bhatia^1^, Kajal Jain^1^, Revathi Nair^1^, Bisman Khurana^2^

^1^PGIMER Chandigarh, ^2^PILBS Mohali Punjab

**Introduction**: Popliteal sciatic nerve block (PSNB) is used alone or combined with a femoral or saphenous nerve block for below-knee surgeries. The supine posterior out-of- plane approach offers less chance of puncturing the popliteal vessels and a shorter needle path but requires hip and knee flexion. Recently described USG (ultrasound) guided CAPS (Crosswise approach to popliteal sciatic) block offers the advantage of better patient comfort as there is no need for patient positioning, flexion of hip and knees and external assistance. We aim to compare the USG-guided posterior approach of PSNB and CAPS block.

**Methods**: A total of 60 patients were included in the study and were randomized into two groups: 1. Classical PSNB group (posterior approach), 2. CAPS group. The primary outcome was time to perform the block. Secondary outcomes were block onset time, number of needle passes, level of procedure-related pain, sciatic nerve visibility score, depth from skin, VAS in 48-hour postoperative period, time to first rescue analgesia, 48- hour rescue analgesic consumption, and any adverse events/complications.

**Results**: We report here the preliminary results of 25 patients. Time to perform the block was not statistically lower (p = .086%) in the classical group [2.04 (1.50 to 2.23)] compared to the CAPS group [3.12 (2.12 to 4.21)]. Onset time, number of needle passes, sciatic nerve visibility score, depth of sciatic nerve from the skin, and success of block were comparable in both groups (p > .05%). 48-hour rescue analgesic consumption and VAS were also comparable in both groups (p > .05%). No adverse events or complications were reported in the groups.

**Discussion/Conclusions**: CAPS provides a similar technical and clinical profile as compared to classical PSNB.

## Primary Care’s Perspectives on Chronic Pain Management in Language-Discordant Contexts

Camilia Thieba^1^, Taraneh Tabatabaei^1^, Krystal Kehoe MacLeod^2^, Maya Gibb^2^, Lina Shoppoff^1^, Tracey O’Sullivan^1^, Sathya Karunananthan^1^

^1^University of Ottawa, ^2^Ottawa Research Institute

**Introduction**: Chronic pain affects nearly 8 million Canadians, disproportionately burdening seniors, low-income individuals, those with mental health or substance use disorders, and marginalized racial and ethnic communities. These populations face significant barriers to adequate pain management, which are exacerbated in language- discordant care settings where patients and providers do not share a common language. Despite this, limited research examines the impact of language barriers on chronic pain care. This study explores primary care providers’ perspectives on managing chronic pain in such contexts.

**Methods**: We conducted virtual, semi-structured interviews with 12 healthcare professionals, including family physicians, nurses, and allied health providers, from a primary care clinic in Ottawa. Participants discussed their experiences with language-discordant care and evaluated tools such as interpreters and translated questionnaires. Reflexive thematic analysis was used to identify challenges and opportunities for improvement.

**Results**: Preliminary findings reveal significant challenges, including fears of miscommunication, cultural and mental health complexities, and difficulties accessing non-pharmacological treatments like physiotherapy, especially for low-income patients. Providers noted a lack of patient-centered multilingual resources and culturally informed pain management strategies.

**Discussion/Conclusions**: This study highlights the need for structural reforms to improve chronic pain care for linguistic minorities. Recommendations include training healthcare providers, developing inclusive multilingual resources, and implementing culturally competent care practices. Addressing these gaps can improve access, equity, and outcomes for marginalized communities facing chronic pain

## Evaluation of Group Transdiagnostic CBT for Psychological Distress in Chronic Pain

Greg Tippin^1^, Abi Muere^2^

^1^Hamilton Health Sciences, ^2^Toronto Western Hospital

**Introduction**: Chronic pain is highly comorbid with emotional disorders (e.g., depressive and anxiety disorders). Patients with these comorbid conditions have worsened physical, emotional, and social functioning. Transdiagnostic cognitive behavior therapy (TD-CBT) is an effective intervention for emotional disorders and may have benefit for patients with comorbid chronic pain. The aim of the study was to examine the effectiveness of a TD-CBT group in treating psychological distress in patients with chronic pain receiving services at the Michael G. DeGroote Pain Clinic.

**Methods**: Forty-one participants completed an 8-session TD-CBT group between September 2021 and May 2024. Participants completed baseline and post-intervention measures of pain intensity, depression/anxiety symptoms, general psychological distress, pain catastrophizing, kinesiophobia, and pain-related disability. One-tailed paired sample t-tests were conducted to test for significant decreases in post-intervention scores compared to baseline.

**Results**: There were significant decreases in pain intensity scores, depression/anxiety symptoms, and general psychological distress following completion of the 8-session TD-CBT group, ts(40) > 2.58, ps < .007, after applying the Bonferroni correction.

**Discussion/Conclusions**: Findings demonstrate the potential effectiveness of a TD-CBT group in reducing psychological distress, depression/anxiety symptoms, and average pain intensity in patients with chronic pain, though results suggest that this intervention was not effective in reducing pain-related factors (catastrophizing, kinesiophobia, disability). TD-CBT may serve as a useful intervention for reducing psychological distress in preparation for directed pain management programming.

## Exploring the Needs of Chronic Pain Patients and Their Informal Caregivers for Diverse Pain Education Tools

Yuemei Wu^1^, Armin Froozanfar^1^, Tim Kagiri^1^, Chris Shaw^1^, Diane Gromala^1^

^1^Simon Fraser University

**Introduction**: To conduct an initial needs analysis for building a pain education application, we developed a hands-on participatory design approach from longstanding user-centered practices in Human-Computer Interaction (HCI) as they overlap with patient-research methods. This study included six patients, each accompanied by their nonprofessionally trained caregiver (partner, family member, friend). Few studies

include caregivers despite their importance in helping patients grapple with chronic pain.

**Methods**: In this 3.5-hour research study, each patient-caregiver pair explored their needs from multiple perspectives: the affordances and limits of common clinical pain measures; their emotional states evoked with affective visuals (“mood boards” from a specialized library); in-depth questionnaires assessing a short patient education video, patient education physical cards and a patient education app. Finally, open-ended

interviews were thematically coded.

**Results**: Both kinds of participants strongly preferred practical, actionable pain management strategies and trustworthy tools that can be personalizable and frequently accessible. Caregivers sought education specifically to help and more deeply understand the lived experiences of people with pain they care for. A fundamental finding of the study is how much patient education depends on the variable capacity of the patient. The study also underscored considerations for HCI developers-researchers: pacing information, the importance of social stigma, any-time encyclopedia-like accessibility, and “serious ease-of-use” – no matter the media.

**Discussion/Conclusions**: This work advances pain education by centering on lived experiences and the social context of caregiver perspectives, informing more effective, inclusive tools for chronic pain management.

## Implementation of the Power Over Pain Portal to Empower Those Waiting for Spinal Care at The Ottawa Hospital with Stepped Care Resources for the Management of Pain, Mental Health, and Substance Use

Amin Zahrai^1^, Etienne J Bisson^2, 3^, Yaadwinder Shergill^1^, Rachael Bosma^4, 5^, Joshua A Rash^6^, Lynn Cooper^1^, Natalie Zur Nedden^1^, Jenny Olson^1^, Megan MacNeil^7^, Eugene K Wai^8^, Patricia Poulin^1, 8^

^1^The Ottawa Hospital Research Institute, ^2^Kingston Health Sciences Center, ^3^Queen’s University, ^4^University of Toronto, ^5^Women’s College Hospital, ^6^Memorial University of Newfoundland, ^7^University of Calgary, ^8^The Ottawa Hospital

**Introduction**: The Power Over Pain (POP) Portal provides free access to pain self-management resources including education, self-directed courses, peer support, and interactive workshops. We integrated the POP Portal within a spinal care program and evaluated acceptability and usability among referred patients.

**Methods**: Patients from The Ottawa Hospital’s Spine Central Intake program, deemed nonurgent or unsuitable for surgery, were referred to the POP team by administrative staff. They were introduced to the Portal and its resources through virtual orientation sessions and then encouraged to engage with the Portal over the following 4 weeks. We conducted semistructured interviews to assess patient acceptability, usability, and intentions for continued use. A thematic analysis was performed to evaluate patient feedback.

**Results**: Of the 67 patients referred (50.7% female; age = 56.5 ± 18.4 years), 35 (52.2%) completed the orientation and all intended to use the Portal. Among the 23 patients interviewed at 4-week follow-up, 18 (78.2%) actively engaged with the Portal over the 4-week period with all recommending it to other spinal care patients and 17 (73.8%) expressing intentions to continue using it. Patients accessed various resources, most commonly the back pain webinar, sleep and mindfulness tools. Ten (55.6%) created a Portal account and 6 (33.3%) completed health self-assessments. The Portal’s acceptability (24/30) and usability (83/100) scores met satisfactory threshold. Patients valued it for pain education, guidance while awaiting clinic appointments, and validation of their experiences.

**Discussion/Conclusions**: Most spinal care patients referred to the POP Portal found it acceptable and useful in addressing their pain management needs.

